# Absorption Intensities of Organic Molecules from Electronic Structure Calculations versus Experiments: the Effect of Solvation, Method, Basis Set, and Transition Moment Gauge

**DOI:** 10.1021/acs.jctc.4c00642

**Published:** 2024-08-14

**Authors:** Jorge C. Garcia-Alvarez, Samer Gozem

**Affiliations:** Department of Chemistry, Georgia State University, Atlanta, Georgia 30302, United States

## Abstract

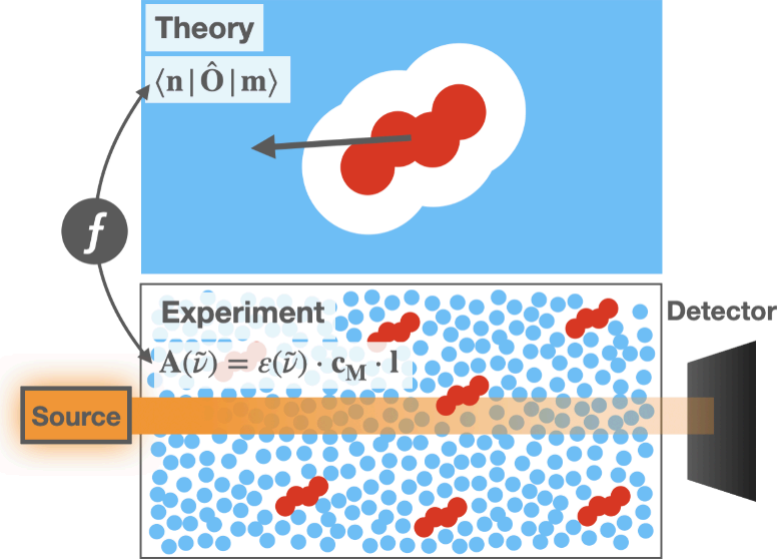

Recently, we derived experimental oscillator strengths (OSs) from well-defined UV–visible absorption spectral peaks of 100 molecules in solution. Here, we focus on a subset of transitions with the highest reliability to further benchmark the OSs from several wave function methods and density functionals. We consider multiple basis sets, transition moment gauges (length, velocity, and mixed), and solvent corrections. Most transitions in the comparison set come from conjugated molecules and have π → π* character. We use an automated algorithm to assign computed transitions to experimental bands. OSs computed using the Tamm–Dancoff approximation (TDA), CIS, or EOM-CCSD exhibited a strong gauge dependence, which is diminished in linear response theories (TD-DFT, TD-HF, and to a smaller degree LR-CCSD). OSs calculated from TD-DFT with PCM solvent models are systematically larger than apparent OSs derived from experimental spectra. For example, *f*_comp_ from hybrid functionals and PCM have mean absolute errors that are ∼10% of *n*·*f*_exp_, where *n* is a solvent refractive index factor that arises from the energy flux of the radiation field in a dielectric (solvent). Theoretical cavity field corrections considering spherical cavities do not improve the agreement between computed and experimental data. Corrections that account for the molecular shape and the direction of transition dipole moments, or that explicitly account for the effect of solvent molecules on the local field, should be more appropriate.

## 1 Introduction

Among quantum mechanics’ earliest successes was its ability to explain and predict spectra, from blackbody radiation to atomic emissions. The agreement between calculations and spectroscopic quantities remains an important and widely used metric for assessing the accuracy of quantum chemical theories and models. Many benchmark studies have provided data on the accuracy of computed electronic transition energies in molecules. For instance, vertical energies computed with time-dependent density functional theory (TD-DFT) can lie within a fraction of an electronvolt from experimental λ_max_.^1^ Specifically for π → π* transitions in organic dyes, a review of TD-DFT computations reports typical deviations in the 0.15–0.25 eV range.^2^ Protocols aimed at reproducing experimental adiabatic excitation energies and that take into account vibrational zero-point energies have achieved chemical accuracy (errors under 1 kcal·mol^–1^ or 0.043 eV) on small molecules using systematically improvable but more time-consuming *ab initio* excited-state methods.^3^

Compared to electronic transition energies, far fewer studies have looked into the ability of computational methods to accurately reproduce absorption intensities. This is the focus of the present work. We start by briefly reviewing some of the benchmark studies for oscillator strengths (OS, or *f* values) in the literature.

Most studies have focused on comparing *f* values computed using one method to another suitable computational reference. For instance, Silva-Junior et al.^4^ compared TD-DFT (BP86, B3LYP, and BHLYP) and DFT-based multireference configuration interaction (DFT/MRCI) *f* values to the best theoretical estimates from *ab initio* methods such as MS-CASPT2, CC2, and CCSD.^4,5^ They focused on optically allowed transitions from 28 medium-sized organic molecules and found that TD-DFT generally underestimates the *ab initio* OSs, while DFT/MRCI *f* values were comparable, with a mean absolute deviation in the range 0.06–0.08.^4^

Similarly, Caricato et al.^6^ assessed the performance of TD-DFT functionals, RPA, CIS, and CIS(D) relative to EOM-CCSD calculations in a set of 11 small organic molecules containing alkenes, carbonyls, and azobenzenes. They analyzed a total of 69 states: 30 valence and 39 Rydberg (Ry) in nature. They found significant variations between functionals and a marked dependence of the error magnitude on the molecule.^6^

A few studies have directly compared computed and experimental *f* values. Chrayteh et al.^7^ studied the excited state properties of 13 small molecules in the gas phase using the CC-expansion and extrapolating to the complete basis set limit. They found that their computations fall within experimental errors for transitions with experimentally reproducible *f* values.^7^ Jacquemin et al.^8^ compared *f* values computed with the Bethe–Salpeter equation (BSE) formalism (combined with the GW approximation) to experimentally derived *f* values of 30 anthraquinones in dichloromethane.^9^ The BSE/GW calculations reproduced the experimental trend. They report a *R*^2^ value of 0.819 for the linear regression between the computed and experimental data, even though the calculation did not include solvation effects.^8^

In a study that focused on N_2_, CO, formaldehyde, ethylene, and benzene, Tawada et al.^10^ found that LC-functionals (LC-BOP, LC-BLYP, and LC-PBEOP) were able to correctly reproduce the order of magnitude of the experimental *f* values in N_2_ Ry transitions. On the other hand, pure functionals (BOP, BLYP, and PBEOP) underestimated *f* values by 2 orders of magnitude, and B3LYP underestimated them by 1 order of magnitude. With a less marked difference in CO’s σ → π* transition, LC-TDDFT still outperformed B3LYP and pure functionals (the worst performing again). In formaldehyde, ethylene, and benzene, results became more mixed, with LC-TDDFT greatly overestimating C2H4’s π → π* transition.^10^

Miura, Aoki, and Champagne^11^ focused on lowest-energy dipole-allowed (mainly π → π*) transitions in benzene, phenol, aniline, and fluorobenzene. They compared seven functionals (SVWN, BLYP, PBE, TPSS, B3LYP, PBE0, and BHandHLYP) to available gas-phase experimental *f* values, and to RPA, CIS, CCS, CC2, and CCSD calculations. They investigated the effect of basis set (Pople’s 6-31G*, 6-311G*, 6-311G**, and 6-311++G**; and Dunning’s cc-pVDZ to cc-pV5Z, aug-cc-pVDZ to aug-cc-pVQZ, and d-aug-cc-pVDZ to d-aug-cc-pVTZ) on the energies, *f* values, and character of the transition computed with B3LYP and PBE0. An important conclusion from their work is that diffuse basis functions are important for both energies and OSs. For example, the decrease in excitation energy (in agreement with experiment) is more pronounced going up in the cc-pVXZ than in the aug-cc-pVXZ series of basis sets. The corresponding *f* values increase with the former and decrease (in agreement with experiment) with the latter series. The bulk of their calculations were carried out with the 6-311++G** basis set. When expanding their benchmark to include chlorobenzene, anisole, and phenetole and comparing their calculations to experiments, they found that the computations correlated well with the experimental data but were not in quantitative agreement. For example, TD-B3LYP calculations yielded a slope of 1.48 and a y-intercept of −0.26 when plotted against experimental data, but the correlation coefficient (R) value was 0.94. Other functionals gave comparable results.^11^

For more information about oscillator strength benchmark studies in the literature published before 2013, we refer the reader to ref (1).

Recently, we generated a collection of *f* values from experimental UV–visible absorption spectra of 100 organic molecules in solution.^12^ A total of 164 OSs were obtained by integrating the attenuation coefficient  over the limit of well-defined bands in the spectra. Transitions were categorized as either very high (VH), high (H), medium (M), low (L), or very low (VL) confidence on the basis of the reproducibility and quality of the fitting. We refer the reader to ref (12). for more details on the fitting, integration, categorization, and a discussion of the sources of error of these *f* values. While errors in experimental OSs are difficult to quantify, we expect that experimental errors in the condensed phase should be smaller than in the gas phase due to wider peaks relative to the spectrophotometer resolution and better integrability of the spectra.^12,13^

Here, we employ this benchmark set to compare OSs computed using several TD-DFT functionals and wave function methods. Aspects that affect the comparison between theory and experiments, such as experimental deviations from the Beer–Lambert Law, solvent effects, and the *f* value dependence on the energy of the electric transition, are discussed.

The manuscript is structured in four sections. To provide a framework for how computed and experimental OSs can be compared, the first section presents a concise background explaining how *f* is obtained from absorption experiment observables (Experimental oscillator strength) and from quantum theory (Theoretical oscillator strength). A third subsection discusses cavity field corrections in the literature. The next section details the approach used to compute *f* in this work (Computed absorption transitions) and outlines how the computed OSs are compared with the experimental data (Statistical analysis). The last two sections present the results of the benchmark and conclusions, respectively.

Modern quantum chemical methods have become valuable tools for early stage screening of novel dyes.^14^ Chromophores with strong absorption and emission have applications in areas spanning solar energy and light-emitting devices to bioimaging. For such applications, we emphasize the importance of selecting computational methods that accurately predict relative transition strengths as well as transition energies.

## 2 Theoretical Background

### 2.1 Experimental Oscillator Strength

A detailed discussion of the experimental aspects and theory of UV–visible absorption spectroscopy can be found, for example, in refs (15 and 16). Here we briefly summarize some main points that connect the experimental observables to the oscillator strength and provide context for later discussion on the validity of the expressions used.

**Figure 1:**
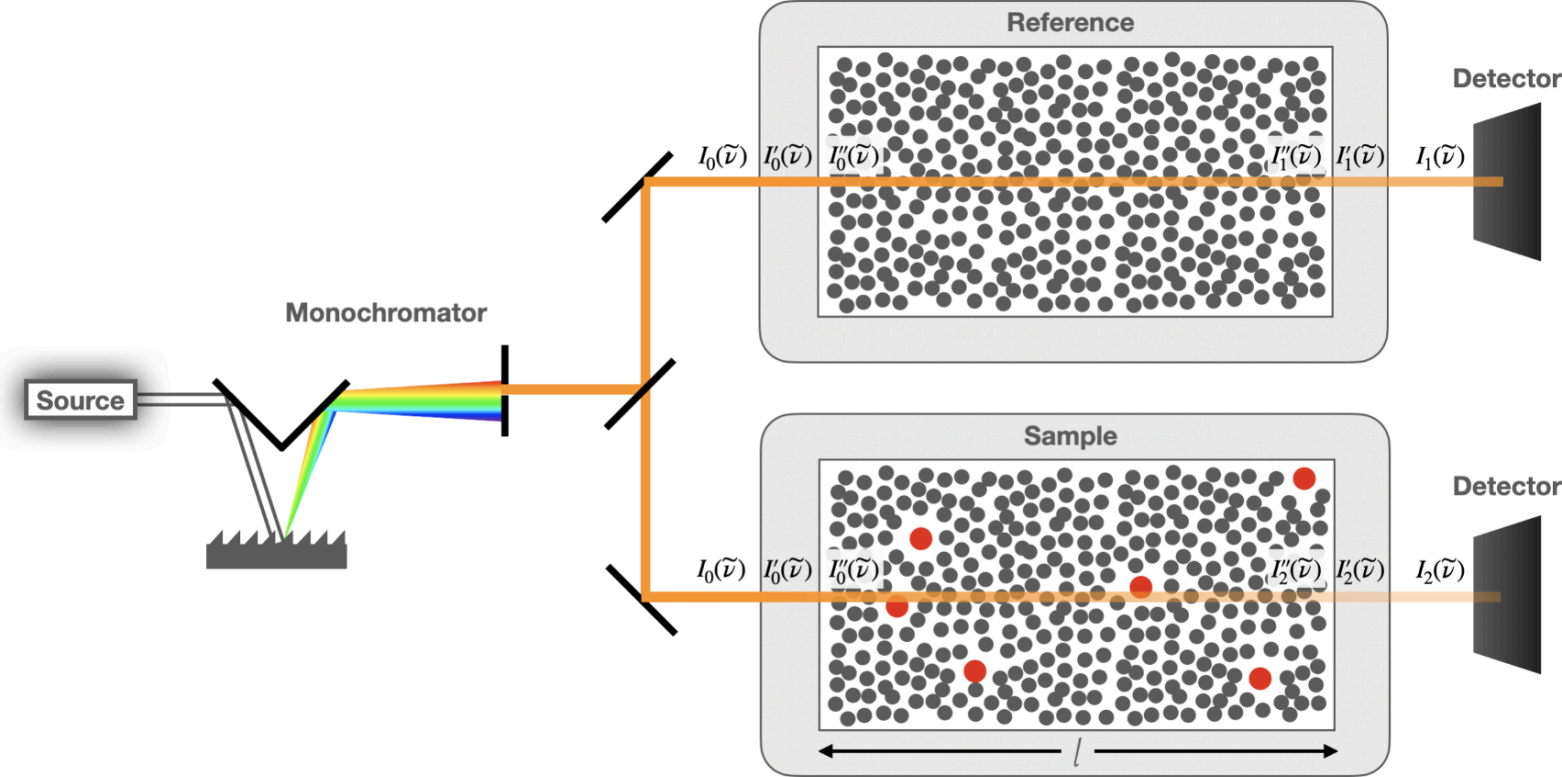
A scheme of the typical absorption experiment setup. Here, *I*_0_(ν̃) is the intensity of the incident light in air entering the cuvettes, *I*_1_(ν̃) is the intensity of light leaving the reference cuvette and reaching the detector, and *I*_2_(ν̃) is the intensity of light leaving the sample cuvette and reaching the detector. The terms with primes indicate changes in the intensity of light as it enters and exits the cuvette walls.

A representative UV–visible absorption experimental setup in the condensed phase (Figure 1) involves nearly monochromatic, collimated electromagnetic radiation traveling through a cuvette containing the molecule of interest dissolved in a solvent. A sensor measures the intensity of light after it traverses the cuvette. This intensity is compared to a reference, typically light that has traveled through an identical cuvette filled only with the solvent, to account for the intensity reduction resulting from reflection, scattering, or absorption by molecules other than the solute.^16^ [Depending on the spectrophotometer used and the wavelength of interest, the light reaching the cuvette will exhibit a certain degree of polarization arising from reflection and refraction in the optical elements of the monochromator. For example, one of the spectrophotometers used for some of the molecules in the benchmark, the Cary model 14, was found to have varying degrees of polarization, going from fairly constant in the UV, to more varying in the visible, to sharp maxima in the IR.^17^ This effect should not be important for an isotropic distribution of molecules (such as molecules in solution) but becomes relevant for an anisotropic or partially oriented sample.^15^]

The intensity of light of wavenumber ν̃ reaching the sample, *I*_0_(ν̃), and the intensity leaving it, *I*(ν̃), will determine the spectral absorbance *A*(ν̃), a magnitude commonly used to describe the reduction in the light intensity:
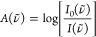
1

The absorbance of a solute of interest is then found from the difference between the absorbance obtained for the solution (sample) and the pure solvent (reference):^16^
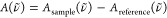
2

Assuming the solution and solvent reference are measured under identical conditions, effects such as scattering and reflection by the solvent and cuvette walls that reduce light intensity cancel out, and the expression for the absorbance of the solute simplifies to
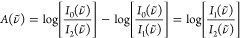
3where *I*_0_(ν̃) is the intensity of light reaching the cuvettes, *I*_2_(ν̃) is the intensity leaving the cuvette with the sample, and *I*_1_(ν̃) is the intensity of light leaving the cuvette with the reference. [Upon normal incidence on a surface separating two media, the transmitted electric field vector is **E**_**t**_ = 2η_*t*_/(η_*t*_ + η_*i*_)**E**_**i**_, where **E**_**i**_ is the incident electric field vector,  is the intrinsic impedance of the medium of the incident waves,  is the intrinsic impedance of the medium of the reflected waves, μ_*i*_ and μ_*t*_ are the relative permeabilities, and ϵ_*i*_ and ϵ_*t*_ are the dielectric constant of these media. Therefore, the intensities in air are related to those in solution by the same multiplicative constant. That is, *I*_1_″(ν̃) = *cI*_1_′(ν̃) and *I*_2_″(ν̃) = *cI*_2_′(ν̃), where *c* is a common constant.] The absorbance measured in this way will correspond to a reduction in intensity given by the Beer–Lambert law,
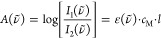
4where ε(ν̃) is the decadic molar extinction coefficient (also called absorption or attenuation coefficient), *c*_*M*_ is the molar concentration of the solute in the solution, and *l* is the path length of the light through the solution. The linear correlation between absorbance *A*(ν̃) and concentration *c*_*M*_ in the Beer–Lambert law requires that^15,18^ (i) the solute molecules do not aggregate, (ii) light scattering by the solute molecules is negligible, (iii) *I*_0_(ν̃) is small and multiphoton processes, excited-state populations, and photochemical reactions are negligible, and (iv) the spectral bandwidth of the light used is narrow compared to the transition bandwidth, i.e., the light leaving the monochromator spans only a narrow range of frequencies. [A narrow spectral bandwidth, i.e., being as close as possible to monochromatic, can be critical when obtaining atomic or highly resolved vibronic spectra. The monochromator spectral bandwidth should be smaller than the absorption bandwidth of the sample to resolve it properly. This is less of a concern for broad-band absorptions such as the ones in the experimental reference spectra used in this work.]

In molecules, the spectral band for a given electronic transition is spread over a wide range of frequencies by each electronic level’s vibrational and rotational substructures. Solvent effects further broaden the line shape of transitions. The transition probability is obtained by measuring the full range of ε(ν̃) as a function of ν̃ for that specific excitation.

Since *A* is dimensionless, the units for ε are determined by the units of *c*_*M*_ and *l*, typically moles per liter (mol/L) and centimeters (cm), respectively. Therefore, ε is usually reported in units of *L*/(mol ·cm) or equivalently in *M*^–1^*cm*^–1^.

The reduction in intensity can be equivalently expressed in terms of the absorption cross section of the solute in the specific solvent σ(ν̃) as
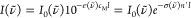
5where *n*′ is the number density (number of molecules per unit volume). The following expression can be used to convert from cross sections expressed in *cm*^–2^ and attenuation coefficients in *cm*^–1^*M*^–1^:
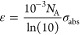
6where *N*_*A*_ is Avogadro’s number.

Delving into the derivation of the Beer–Lambert law in terms of the absorption cross-section offers valuable molecular-level insights into the approximations inherent in the law. A detailed discussion can be found in ref (19)., where the law is derived by equating the probability of a photon traversing the sample length *l* (without being absorbed) to the probability of encountering no molecules within a cylinder of base equal to the molecular absorption cross-section σ(ν̃) and of height *l*. Although we will focus on OSs as our primary metric, we will revisit the cross-section perspective later as we describe solvent effects theoretically.

The connection between oscillator strength *f* and the experimental metrics of attenuation is given by *f*’s historical origin as a link between classical electromagnetic dispersion theory and quantum theory. Classically, the solutions in the absorption experiments can be described as isotropic nonmagnetic media with the constitutive relations D = ϵ̃E and **B** = **H**, since the magnetic permeability of typical solvents μ is practically equal to vacuum’s μ_0_^15^ (we are using Gaussian units^20^ so that μ ≈ μ_0_ = 1 and |*H*_0_| = |*E*_0_|). The propagation of a plane, monochromatic, linearly polarized wave in such media is given by
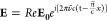
7where *c* is the speed of light in vacuum, and *ñ* is a complex index of refraction:
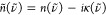
8The real part of (8), *n*(ν̃), quantifies the phase velocity (the usual refractive index) while the imaginary part, κ(ν̃), quantifies absorption in the medium. The complex refractive index is related to the complex dielectric constant by .^21^ [Rigorously, all magnitudes in eq 8 depend also on temperature *T* and the (number) concentration of molecules (*n* = *n*(ν̃,*T*,*n*′), κ = κ(ν̃,*T*,*n*′))]. The average light intensity at a depth *x* is given by
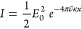
9or in terms of the intensity at *x* = 0, *I*_0_:
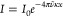
10

Comparing (10) to (5) then:
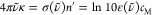
11

In classical dispersion theory, the interaction of electromagnetic radiation with the medium is described by a model that has electrons harmonically bound to positive charges (e.g., nuclei). These oscillating electrons have characteristic frequencies and damping constants, resulting in distinctive oscillations when the frequency of the incident field is close to the characteristic frequency (in analogy to the resonant character of atomic electronic transitions). The polarization induced in the medium by the time-varying external field, without accounting for any ordering of the permanent molecular dipoles, is described in terms of the displacements induced in the harmonically bound electrons.^21−25^ In this way, the model accounts for the dielectric constant ϵ̃ and determines both the real and imaginary parts of the refractive index *ñ*.

In 1921, in the context of the development of quantum mechanics, Ladenburg introduced the oscillator strength^26^ as a quantity that represents the fraction of the total number of atoms/molecules that have a “dispersion electron,” i.e., those electrons oscillating with the characteristic frequency of a given electronic transition.

From the relations above, *f* values can be expressed in terms of the integrated absorption intensity of a band as^15,18^
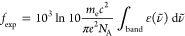
12where ε(ν̃) is expressed in *M*^–1^*cm*^–1^ and dν̃ in *cm*^–1^.

Two comments regarding eq 12 must be made. First, it is common to include the (real) refractive index of the medium in the denominator of (12) to account for the effect of the solvent on the electric field “felt” by the solute.^15,18^ This correction is expected to make *f* values obtained in different solvents directly comparable. The factor 1/*n* is recognized to be a rough approximation^15^ for a complicated problem with several authors proposing different corrections. This is discussed further at the end of the section.

Second, while the OS is a well-defined magnitude for electronic transitions in atoms or even for a line corresponding to a vibronic transition in a molecule, it does not have the same clear meaning for a molecular spectral band.^23,27,28^ The main obstacles are the temperature-dependent population of energy sublevels, and the spread of frequencies over which molecular transitions are possible.

Nonetheless, *f* values obtained according to eq 12, as a function of the integrated intensity if nothing else, quantify the probability of an electronic transition in a way suitable to compare to theoretical strength computations.

### 2.2 Theoretical Oscillator Strength

In the linear regime (where the Beer–Lambert law is valid), the reduction in light’s intensity as it goes through a sample is attributed exclusively to one-photon processes. At these intensities, light can be treated classically while the light–molecule interaction can be described using time-dependent perturbation theory. Such a semiclassical treatment of the interaction is presented at length in several quantum mechanics textbooks such as refs (29−32). as well as in books focused on spectroscopy such as refs (15 and 28). To frame our comparison with experimental strengths, a minimal discussion of the key points in the derivation is provided below.

A linearly polarized plane wave, such as (7), can be expressed in terms of the vector potential **A**. Under the Coulomb gauge, and in the absence of charges, **A** relates to **E** and **H** via **E** = −(1/*c*)∂**A**/*∂t* and **H** = ∇ × **A**. Expressed as a function of time (*t*) and position (**r**), **A** is given by
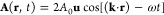
13
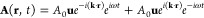
14where **u** is the direction of polarization of the wave, **k** is the wave vector that points in the direction of propagation of the wave, and ω is the angular frequency. The magnitude of the wave vector, |**k**|, is related to the wavelength of light λ by |**k**| = 2π/λ and to ω and the speed of light in vacuum *c* as |**k**| = ω/*c*, while the angular frequency ω = 2*πν* is related to the wavenumber in vacuum as ω = 2πν̃c. The constant *A*_0_ represents the intensity of light and can be related to the average number of monochromatic photons of energy *ℏω* per unit volume, *N*_*photons*_:^30^
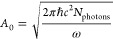
15

The probability per unit time *P*_*nm*_ of a molecule absorbing a photon of the incident field, described by (14), and undergoing a transition from an initial state *m* to a final state *n* is given by:
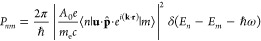
16where *e* and *m*_*e*_ are the charge and mass of an electron, respectively, **p̂** is the linear momentum operator, *E*_*m*_ and *E*_*n*_ are the energies of the initial and final states, respectively, δ(*x*) is Dirac’s delta function, and ⟨*n*|**u**·**p̂**·*e*^*i*(**k**·**r**)^|*m*⟩ is the transition moment integral. [From eqs 16–23, *n* represents the final state of a system, while in the rest of the manuscript *n* refers to a solvent refractive index.]

Expression (16) is obtained as a first-order approximate solution in a time-dependent perturbation-theory treatment of the interaction. The perturbation used,  is the most relevant term from the classical Hamiltonian for the interaction of an electron with an electromagnetic field characterized by the vector potential **A** (and the scalar potential ϕ). Dirac’s δ(*x*) in (16) represents the resonant character of the transition and conservation of energy.

The position **r** in (14), and consequently in (16), is measured from an arbitrary origin. Choosing it to be located on the molecule is convenient to carry out the integration in (16) since the integrand will be nonvanishing only in the proximity of the molecule, where the wave functions are different from zero. Given the relatively small dimensions of a molecule compared to the wavelength of the electromagnetic radiation, **k** ·**r** ≪ 1 in the relevant volume. It is convenient then, to expand the exponential term *e*^*i*(**k**·**r**)^ in (16) using the definition of an exponential function in the complex plane:
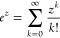
17where . This gives the infinite series:
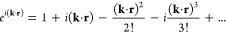
18

Taking *e*^*i*(**k**·**r**)^ ≈ 1 results in what is known as the dipole approximation:
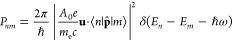
19

Expression (19) provides the probability per unit time of an induced transition on a specific molecule. Such a probability will depend on the orientation of the molecule relative to the polarization of the incident wave, as indicated by the product u·⟨n|**p̂**|m⟩. Therefore, *P*_*nm*_ will be maximal when **u** and ⟨n|**p̂**|m⟩ are aligned in the same direction. On the other hand, for a perpendicular orientation of these vectors, *P*_*nm*_ will be 0. For any given molecule, 0 ≤ **u**·⟨*n*|**p̂**|*m*⟩ ≤ |⟨*n*|**p̂**|*m*⟩| (since |**u**| = 1). The different orientation of the molecules relative to the polarization of the field results in an average value of |⟨*n*|**p̂**|*m*⟩|^2^/3 for the product |**u**·⟨*n*|**p̂**|*m*⟩|^2^ when considering all the molecules in an isotropic sample.^33^

The commutation relation between the individual components of the position **r̂** and momentum **p̂** operators can be used to relate the transition moments associated with each:
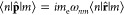
20

The equality in (20) only holds for exact wave functions. The approximate nature of ⟨*n*| and |*m*⟩ makes the transition probabilities computed from ⟨n|**p̂**|m⟩ (referred to as dipole velocity formulation) or ⟨n|**r̂**|m⟩ (dipole length formulation) differ. These two formulations are perhaps the most widely used, though in general the transition dipole moments can be expressed in terms of other operators.^34,35^

The oscillator strength *f*_*nm*_ for a transition from an initial state *m* to a final state *n* is obtained from *P*_*nm*_ and can be expressed in either the length formulation (superscript lg), the velocity formulation (vg), or a mixed formulation (mx), as follows [Several linear combinations of ⟨n|**p̂**|m⟩ and ⟨n|**x̂**|m⟩ multiplied by each other’s complex conjugates are suitable for an *f*_*nm*_^*mx*^ expression independent from the energy of the transition. The notation  in eq 23 is meant to represent any of them. For example, Gaussian^37^ outputs report the expression .]:^35,36^
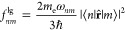
21
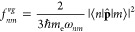
22
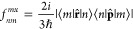
23

The expressions above do not explicitly account for solvent effects. The presence of a solvent influences transition probabilities in three ways: (i) the solvent may chemically alter the solute (e.g., tautomerization, acid–base reactions, complexation, etc.), (ii) the solvent’s electrostatic potential acts on the solute, and (iii) the solvent affects the incident electromagnetic field that drives the electronic transition on the solute.^38,39^

Effects (i) and (ii) would affect the probability (per unit time) of an induced electronic transition, as given by eq 19, by altering the wave functions of the absorbing molecule in the ground and excited state, therefore affecting the transition dipole moment ⟨n|**p̂**|m⟩. On the other hand, effect (iii) would modify the intensity of the perturbation along the direction of the transition dipole moment which is represented in eq 19 by the projection of the term *A*_0_**u** along the direction of ⟨n|**p̂**|m⟩.

If considered in the context of the Beer–Lambert law derivation, where the decrease in light intensity *dI* as it travels a distance *dx* is given by *dI* = −*Iσn*′*dx*, effects (i) and (ii) would affect the absorption cross-section σ, while effect (iii) would result in a replacement of the light intensity *I*, proportional to the square of the electric field (*E*^2^), with a more complicated function of the incident field: *dI* = −*f*(*E*)σ_solution_*n*′*dx* (where *n*′ is the number of molecules per unit volume).

The first two interactions (i) and (ii) exist in the absence of the incident field. For absorption measurements, solvents and experimental conditions are chosen to prevent as much as possible chemical alteration of the solute, and effect (i) will not be discussed here. The description of the second effect, dating back to Onsager’s “reaction field,”^40^ is historically linked to the description of effect (iii). The reaction field that acts on the solute molecule originates from the polarization of the dielectric medium caused by the solute molecule itself. The reaction field has been the subject of intense research resulting in several *continuum* solvation models that treat the solute–solvent interaction representing the latter with a continuum dielectric material. These models are widely used and implemented in many electronic structure software packages.^41,42^ Effect (ii) can also be treated with calculations including *explicit* solvent molecules around the solute of interest. In this manuscript, we assume that effect (ii) is adequately described by the *polarizable continuum model (PCM)* used in our computations (see the methods section for further details). By default, those methods do not account for effect (iii) in most widely used implementations. We describe that effect in some more detail next to understand how it affects experimentally measured and computed absorption intensities.

### 2.3 Cavity Field Corrections

The effect of the solvent on the incident electromagnetic field “felt” by a solute molecule has been traditionally described by considering a cavity that contains the solute inside a macroscopic dielectric medium that represents the solvent. Considering a dielectric medium upon which an external static (constant-in-time) electric field acts, and a hypothetical spherical cavity large enough that its inner region still can be described by the macroscopic constants of the surrounding medium, Lorentz obtained, for the local field *F*_*L*_ acting upon a charge inside the cavity:^43^
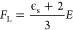
24where *E* is the macroscopic electric field in the dielectric, and ϵ_*s*_ is the static dielectric constant which is assumed to be independent of *E* when the latter is small enough to prevent saturation effects.^44^

Onsager proposed the division of the local field into two components, a “cavity field” proportional to the external field as well as to the polarization induced by this field, and the aforementioned “reaction field” proportional to the dipole moment of the solute molecule. Considering a spherical cavity containing only a dipolar molecule, he obtained for the cavity field *F*_*C*,*O*_:^40^
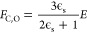
25

Onsager’s theory of polarization has further generalizations. For example, Kirkwood^45^ considered explicitly the electric moments of the first shell of solvent molecules around a solute. His corrections are particularly relevant for polar solvents, since in the vicinity of a given molecule, the surrounding molecules tend to maintain definite (either parallel or antiparallel) orientations of their dipoles.^46^ Detailed derivations and discussion of these effects are provided in ref (44).

Other authors have generalized the local field corrections by considering nonspherical cavities. For example, Scholte^47^ and Shibuya^48^ considered ellipsoidal cavities and the fields along the principal axes of the ellipsoid.

Using the Maxwell relation connecting the refractive index of a medium to its dielectric constant (*n*^2^ = ϵ), the different cavity field corrections have been adapted to time-varying fields for which the permanent dipoles of solvent molecules have no time to reorient. Using these local field expressions, several authors have proposed corrections that relate the “apparent” OS of a molecule when experiments are carried out in solvents with different refractive indexes. We will employ a common notation to summarize some of those corrections: we call *f*″ the OS measured (according to eq 12) for a molecule in a solvent of refractive index *n*, and *f* the OS of the same molecule when the refractive index of the medium is 1.

The first correction dates back to 1934. Chako, using a Lorentz field obtained:^49^
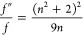
26

A limitation of this expression is that it predicts that *f*″ will always increase, at a fixed rate, when absorption experiments are carried out in solvents of higher refractive index. This is known not to be the case, with notable exceptions such as the π → π* transition of β-carotene.^50^ To correct this issue, Böttcher^51^ and Schuyer^52^ included the polarizability and radii of the solute molecules in their correction. Myers and Birge^50^ considered a cylindrical cavity and the orientation of the transition moment relative to the cavity. Shibuya,^48^ considering the local field along the principal axis of the ellipsoidal cavity he used, obtained for a transition oriented along the principal axis *k* the correction:
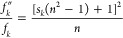
27where *s*_*k*_ is a shape parameter equal to the depolarization factor along the corresponding axis. The value of *s*_*k*_ ranges from 0–1, and ∑_*k*_^3^*s*_*k*_ = 1.^53^ Therefore, Shibuya’s correction *f*″/*f* ranges from 1/*n* to *n*^3^ depending on the molecular shape and relative orientation of the transition moment, and reduces to Chako’s in the limit where the ellipsoid becomes a sphere. Ref (48) contains a more extensive survey of the work in this area prior to 1983.

Other corrections depending only on the refractive index are those by Abe.^54^ Using a Lorentz field Abe obtained the expression:^54^
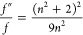
28while when using an Onsager cavity field he obtained:
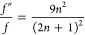
29

In the same paper Abe approximated Schuyer’s expression as
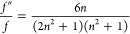
30

For this last expression, it is worth mentioning that instead of eq 12 he considered a modified relation of the OS to the integral of the attenuation coefficient that included the factor *n*^2^ in the denominator.

The value of *n* to use in the corrections above is also subject to debate. Some authors recommend using the refractive index of the solvent for the frequency of the transition, *n*(ν̃), while other authors recommend *n*(*∞*) (the refractive index of a material for a field in the limit of infinite frequency). This latter value has been said to be well approximated by the refractive index at the sodium *D* line (*n*_*D*_).^41^ Warner and Wolfsberg,^55^ in their study of spectra in condensed phases, used the Lorentz field to derive the correction factor
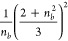
where *n*_*b*_ is the “slowly varying background contribution to the refractive index” that depends on the off-resonance polarizability of the solute molecules. They obtained *n*_*b*_ values by fitting a model for *n*(ν̃) presented in their paper to data from reflection experiments. For benzene, chloroform, and methyl iodide, they found *n*_*b*_ values close to, but consistently lower than, the corresponding *n*_*D*_ values.^55^

It is worth mentioning that the dependence on *n* in the *f*″/*f* correction factors comes not only from the local/cavity field correction. This may be best understood in terms of the absorption cross section (see, for example, the derivations in ref (56).), which is defined as *energy (per unit time) absorbed by the molecule* divided by the *energy flux of the radiation field*. Macroscopically, the flux of energy of the incident electromagnetic wave in a nonmagnetic medium of refractive index *n* is given by^25,57^
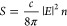
31

While the local/cavity field is considered for the *energy (per unit time) absorbed by the molecule*, eq 31 must be taken into account for the *energy flux of the radiation field*. Comparison of Chako’s correction (eq 26) to the Lorentz local field (eq 24) shows that *n* in the denominator of eq 26 comes from eq 31. The same applies to Shibuya’s correction (expression 27).

The computational description of the reaction field [responsible for effect (ii)] using implicit solvation models typically accounts for solute–solvent electrostatic interactions using apparent charges at the surface of a cavity constructed around the molecule. Details of how the cavity is constructed, how the apparent charge on the cavity’s surface is discretized, or how to treat nonequilibrium effects, have been extensively researched. More recent efforts have been made to describe the cavity field in terms of additional surface charges. See, for example, refs (56, 58, and 59). There have also been efforts to describe the cavity field with polarizable embedding models.^112,113^

## 3 Methods

### 3.1 Computed Absorption Transitions

The coordinates of the 100 molecules optimized at the B3LYP/6-31+G* level of theory were obtained from the Supporting Information of ref (12). Here, we refined the structures at the B3LYP/6-311++G** level of theory.^60−62^ Frequency calculations were carried out at the same level of theory to ensure that all positive frequencies were obtained. The updated geometries are provided in the Supporting Information (SI) as xyz coordinate files.

In this study, we will focus our analysis on 85 transitions categorized as very high, high, or medium confidence in ref (12). This subset of data will be referred to as VHHM throughout this work (where VHHM = VH ∪ H ∪ M). Since more than one transition per molecule is sometimes included, the 85 transitions come from 69 molecules. Table S1 of the SI document lists the molecules and transitions included in the subset.

From the optimized geometries, the energies and OSs of the lowest 30 singlet exited states were computed using single-point TD-DFT calculations. Nine different functionals were tested: One pure functional (SVWN^63,64^), five hybrid functionals (B3P86,^60,65^ O3LYP,^61,66^ mPW1PW91,^67^ M05,^68^ and B3LYP^60,61^), and three long-range corrected hybrid functionals (CAM-B3LYP,^69^ LC-wHPBE,^70,71^ and wB97XD^72^). The 6-311++G** basis set^62^ was also used for all TD-DFT calculations. The solvent effect was included, in both geometry optimizations and single-point calculations, through PCM using the integral equation formalism (IEFPCM).^73^

Excitation energies and OSs for all nine functionals were computed both with and without the Tamm-Dancoff approximation (TDA)^74^ using the same basis set and solvation method. We also recomputed the transition energies and OSs for TD-B3LYP with multiple basis sets: STO-3G, 3-21G, 6-31G*, 6-31++G**, cc-pVDZ, aug-cc-pVDZ, and aug-cc-pVTZ.^62,75−77^

The calculations above were all performed using Gaussian 16 version C.01.^37^ In addition, using the PCM/6-31+G* optimized geometries reported by Tarleton et al.,^12^ we carried out additional calculations using the 6-31+G* basis set with Q-Chem 5.3.^78^ Specifically, we ran the calculation *in vacuo* and also using two additional solvation models; the conductor-like PCM (CPCM) model and COSMO. Those were compared to the B3LYP/6-31+G* calculations using IEFPCM solvation from Gaussian. Differences in the computed OSs using the three solvation models and two different software were negligible, indicating that the strengths are relatively insensitive to the details of the solvent model implementations tested. The gas phase calculations are discussed further in the Results and Discussion Section.

In addition to TD-DFT calculations, we carried out single-point excited state energy calculations using three *ab initio* methods in Gaussian 16:^37^ time-dependent Hartree–Fock (TD-HF), configuration interaction singles (CIS) and equation of motion coupled cluster with singles and doubles (EOM-CCSD). In the case of EOM-CCSD, we compute OSs from linear response transition densities (LR-CCSD) in addition to the unrelaxed EOM ones.^79−81^ EOM-CCSD applies excitation operators to a CCSD ground state reference and includes doubly excited configurations.^82,83^ PCM effects for EOM-CCSD are calculated with a “zeroth-order” approximation that employs solvent-polarized molecular orbitals but misses a complete EOM-CCSD electronic response to the solvent.^84^ More complete models are available but have not been used here.^85−88^

These wave function method calculations were carried out for a smaller subset of 35 transitions from 26 molecules for which EOM-CCSD calculations were tractable. Those molecules and transitions are listed in Table S1 of the SI document. Furthermore, for the EOM-CCSD calculations, only 15 excited states were requested instead of 30. The EOM-CCSD calculations were carried out using the double-ζ aug-cc-pVDZ basis set.^77^

### 3.2 Statistical Analysis

When running, for instance, a TD-DFT calculation for 30 excited states of a given molecule, 30 OSs are obtained in each gauge (i.e., 30 of each of *f*_*nm*_^*lg*^, *f*_*nm*_^*vg*^, and *f*_*nm*_^*mx*^). Here, we will drop the superscripts related to the gauge, as the same discussion will apply to all three gauges. The individual state-specific OSs are denoted *f*_*n*0_, where 0 is the index for the ground state and *n* represents the excited state index (e.g., 1 for the first singlet excited state, 2 for the second singlet excited state, etc.). As a first approximation, a code assigns the computed *f*_*n*0_ to an experimental band of the molecule if its corresponding energy is within the energy limits of the band (ε(ν̃) minima in the experimental spectra). Often, more than one transition contributes to a band. When that is the case, we use the sum of the corresponding *f*_*n*0_ values to find the total OS of the band. The OS computed in this way for a specific band, *k*, is referred to as *f*_*comp*,*k*_ and can be compared to the corresponding *f*_exp ,*k*_.

Our benchmark for OSs faces the inconvenience that not only are the individual *f*_*n*0_ values dependent on energy, but also that the set of *f*_*n*0_ values that contribute to a given *f*_*comp*,*k*_ is affected by the accuracy of the computed (vertical) electronic transition energies. Judging whether or not a computed transition belongs in a band may not be straightforward, even more so if we are not sure whether *f*_comp_ values should reproduce *f*_exp_ values as given by eq 12, or *f*_exp_/*n* as proposed in refs (15 and 18), or one of the other solvent effect corrections proposed. Therefore, an algorithm has been used that actively maximizes the agreement between *f*_comp_ and *f*_exp_ (or *f*_exp_/*n*, *nf*_exp_, etc..) by modifying which *f*_*n*0_s contribute to a band. The algorithm does this by shifting the band limits to include or exclude computed transitions to minimize |*f*_comp_ – *f*_exp_| (or equivalently |*f*_comp_ – *f*_exp_/*n*|, |*f*_comp_ – *nf*_exp_|, etc.). As an initial guess, the band limits are given by the experimental ε(ν̃) minima.

The implementation avoids having a specific transition *f*_*n*0_ double-counted toward two different bands. This should give a “best-case scenario” where the computed OSs are as close as possible to the experimental ones. Note that in case of an incorrect energy ordering of excited states, the algorithm will not be able to repair the issue. While we recognize the shortcomings of this approach, automation was necessary given the large number of computations in the present benchmark.

For each set of computations, two comparisons with experimental data are presented. These are labeled “Exact Band Limits” and “Improved Fit”. In the first case computed *f*_*n*0_ are assigned to an experimental band if the computed energy lies strictly within the energy limits of the band. The second case corresponds to the application of the algorithm described above (labeled “Improved Fit Algorithm” as well). We emphasize that with the use of the Improved Fit algorithm, it becomes only possible to discuss an upper limit to the accuracy of a method’s *f* values. On the other hand, within the Exact band limits framework, large computed energy discrepancies with experimental energies will severely affect the OS comparisons.

To quantify the agreement of a method with the experimental data, two sets of metrics are employed: The first metric is the mean absolute error, MAE, calculated as
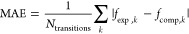
32where *N*_transitions_ is the total number of transitions. The second set of metrics is obtained from linear regression analysis of the (*f*_exp_, *f*_comp_) pairs. A small MAE, a linear fit close to *y* = *x* + 0, and an *R*^2^ value close to one are indicators of a good agreement between the set of *f*_comp_ obtained with a given method and the corresponding set of experimental values *f*_exp_.

Since *f*_*comp*,*k*_ values depend on the computed energy (with *f*_*n*0_ explicitly dependent in the position and momentum gauges) we must also pay attention to how the computed transition energies compare to the experimental ones. To describe the experimental transition energies, an average transition energy is obtained for a band *k* as
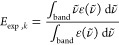
33using the data from ref (89) and digitized in ref (12) for ε(ν̃).

Analogously, computed energies are obtained as oscillator strength-weighted transition energy averages:
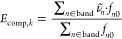
34

Three metrics are employed to monitor how computations reproduce experimental energies:

i) The mean absolute error (in energy) computed as
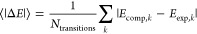
35

ii) The mean error (in energy) obtained as
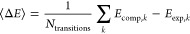
36

iii) The mean ratio of computed to experimental energies, given by
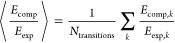
37

As mentioned earlier, solvent effects need to be accounted for when comparing computed vs experimental OSs. In the experimental spectra, the solvent effects are intrinsic to the measured attenuation coefficient leading to an apparent *f*_exp_ obtained from eq 12. On the other hand *f*_comp_ values computed with PCM only account for the reaction field component of the local field while computations without any solvent model make no account at all. That is why, initially, we start by comparing *f*_comp_ not only to *f*_exp_, but also to *f*_exp_/*n* and *nf*_exp_. We also test some of the cavity field corrections mentioned before, noticing the equivalence of *f*_exp_ and *f*_comp_ to the notation used in the cavity field corrections subsection: *f*″ → *f*_exp_ and *f* → *f*_comp_. Making that substitution, for example, on Chako’s correction (eq 26) we obtain
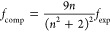
38
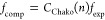
39where *C*_Chako_(*n*) = 9*n*/(*n*^2^ + 2)^2^. We can obtain equivalently *C*_Shibuya,*k*_(*n*) = *n*/[*s*_*k*_(*n*^2^ – 1) +1]^2^, *C*_AbeL_(*n*) = 9*n*^2^/(*n*^2^ + 2)^2^, *C*_AbeO_(*n*) = 9*n*^2^/(2*n* + 1)^2^, and *C*_Schuyer_(*n*) = [(2*n*^2^ + 1)(*n*^2^ + 1)]/(6*n*), from eqs 27, 28, 29, and 30, respectively. The agreement of *f*_comp_ to *C*_Chako_ · *f*_exp_, *C*_AbeL_ · *f*_exp_, *C*_AbeO_ · *f*_exp_, and *C*_Schuyer_ · *f*_exp_ is also tested in this work.

We calculate the expressions above using *n*(ν̃) evaluated at the corresponding frequency of each transition. We used the dispersion formulas reported in the literature for water,^90^ ethanol,^91^ CCl_4_,^92^ dioxane,^92^ acetonitrile,^93^ methanol,^93^ cyclohexane,^93^ hexane,^94^ and heptane.^94^ Those expressions are collected in refs (95 and 96). We also evaluated the correction factors using the value at the sodium *D*-line (*n*_*D*_) obtained from ref (97).

An alternative approach to using these cavity field corrections is to find the optimal scaling factor that relates the computed and experimental OSs. Consider Algorithm 1, shown below. It computes *C* constants that reflect the slope between (*f*_exp_ and *f*_comp_) accounting for the Improved Fit algorithm. The value of *C* often converges after a few iterations. However, in a few cases, the algorithm is sensitive to the initial guess for *C*. This is discussed further in the Results and Discussion Section.

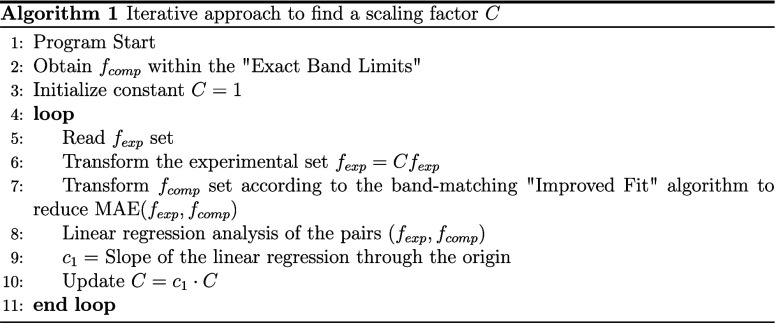



As discussed in the computed absorption transitions subsection, the majority of the statistical analysis focuses on the subset of VHHM transitions. A smaller subset of 35 transitions is used for comparison between wave function methods. We carry out further analysis by looking at subsets of VHHM prepared based on: 1) transition character (π → π*, charge transfer, or mixed character), 2) point group symmetry (*C*_1_, *C*_*s*_, or higher symmetry), 3) solvent (water, electrolyte solution, ethanol, methanol, heptane, hexane, and cyclohexane), and 4) Spectrophotometer used to measure the experimental UV–visible spectra (Zeiss PMQ II, MM12, PerkinElmer 4000 A, Unicam SP 500, or other). In the case of solvents, we also prepared a subset that is a union of protic solvents (ethanol, methanol, water, electrolytes) and aprotic nonpolar solvents (heptane, hexane, CCl_4_, petroleum ether, cyclohexane, dioxane).

## 4 Results and Discussion

**Figure 2:**
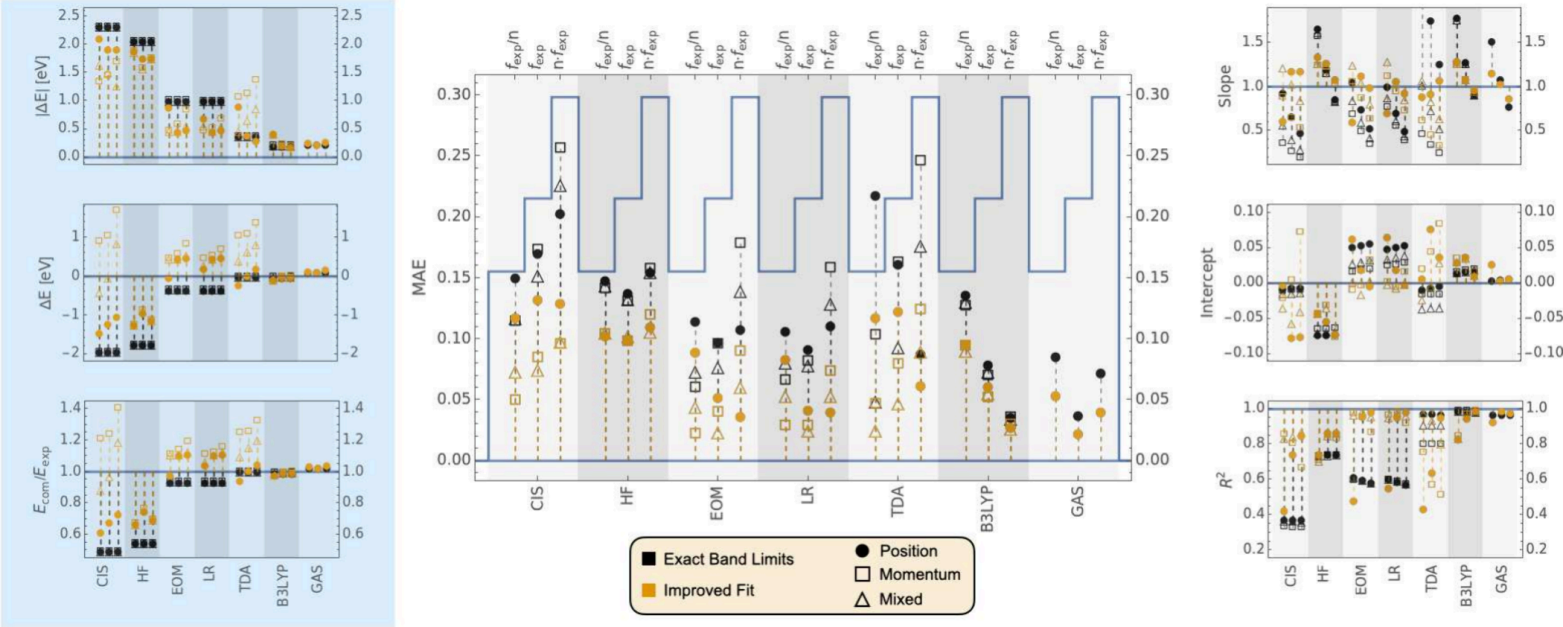
Comparison of *f* values computed using CIS/6-311++G**, TD-HF/6-311++G**, EOM-EE-CCSD/aug-cc-pVDZ, LR-CCSD/aug-cc-pVDZ, TDA-B3LYP/6-311++G**, RPA TD-B3LYP/6-311++G**, and GAS phase-RPA-TD-B3LYP/6-31+G* for a subset of 35 experimental transitions. For each method, the *f*_comp_ values are compared to *f*_exp_/*n* (left), *f*_exp_ (center), and *n*·*f*_exp_ (right) as indicated by the labels on top of the central plot. The blue bar outline indicates the average values of *f*_exp_ multiplied by the respective refractive index prefactor (⟨*f*_exp_/*n*⟩ = 0.155384, ⟨*f*_exp_⟩ = 0.215319, and ⟨*n*·*f*_exp_⟩ = 0.298506). A full circle corresponds to the data obtained with the length gauge. An empty square corresponds to the velocity gauge, and an empty triangle corresponds to the mixed gauge. Markers in black correspond to transitions assigned using the Exact Band Limits, while markers in yellow correspond to transitions assigned using the Improved Fit algorithm. The data displayed can be found in Tables S2 to S19 of the SI.

In ref (12), we focused on benchmarking a single method, TD-B3LYP. In Figure 2, we extend the comparison to include wave function method calculations for 35 transitions that belong to the VHHM subset. Large molecules from that subset are excluded due to computational cost. These transitions are listed in Table S1. Six methods are tested: CIS, TD-HF, EOM-CCSD, LR-CCSD, Tamm-Dancoff approximated (TDA) DFT and TD-DFT.

The B3LYP functional is used for this plot. For the sake of simplicity, at this stage, we consider only three possible approximate solvent effect corrections: *f*_exp_/*n*, *f*_exp_, and *n* · *f*_exp_. We present the other prefactors later in this Section.

In the center of Figure 2, a plot shows the MAE between computed and experimental OSs, calculated using eq 32. The MAEs are shown for different methods (labeled at the bottom of the plot), for different refractive index prefactors for the experimental strengths (*f*_exp_/*n*, *f*_exp_, and *n* · *f*_exp_, shown on the top of the plot), different gauges (represented using different symbols), and before and after the application of the Improved Fit band matching algorithm (black and yellow, respectively). The blue bar outline indicates the average values of *f*_exp_ multiplied by the respective refractive index prefactor, and serves as a reference to allow comparison of the magnitudes of the MAEs relative to the average values of the experimental OSs.

Six additional panels are shown in all figures presented in this section. The panels on the left and right sides are set up in the same way as the central one but with most *x*-axis labels excluded. The results for different methods are shown with alternating background shades to help correlate with the labels in the central panel.

The three panels on the left indicate the agreement between experimental and computed excitation energies, obtained from eqs 33 and 34, respectively. |Δ*E*|, Δ*E*, and *E*_comp_/*E*_exp_ are calculated using eqs 35, 36, and 37, respectively. Notably, if no state was found within the band limits, *E*_comp,*k*_ was assigned a value of zero. Therefore, a negative value of Δ*E* does not necessarily mean that computed transitions are systematically red-shifted with respect to computed ones but instead indicates that many transitions may fall outside the band limits. When this occurs, the associated |Δ*E*| will be large and *E*_comp_/*E*_exp_ will be smaller than 1.

The comparison between computed and experimental energies before (black markers) and after (yellow markers) the Improved Fit algorithm reflects what the algorithm did. The algorithm does not alter the computed energy associated with a given *f*_*n*0_; it just assigns or unassigns computed transitions to each band. Therefore, when the black and yellow markers differ, it means that the algorithm made changes to the band assignments. The values of |Δ*E*|, Δ*E*, and *E*_comp_/*E*_exp_ after the Improved Fit algorithm are more representative of the errors stemming from the electronic structure method used, as it is assumed the algorithm assigned computed transitions reasonably well to the experimental bands.

Figure 2 indicates that CIS and TD-HF computed transitions often fall completely outside of the experimental absorption band limits. This is consistent with previously reported errors associated with CIS, often in the 0.5–2.0 eV range.^98^ The Improved Fit algorithm partly resolves this issue, reducing the errors in the excitation energies, but CIS and TD-HF transitions may still not have been assigned correctly in all cases to the experimental bands. Therefore, those two methods will not be discussed extensively in this section.

Several EOM (or LR) CCSD transitions also fall outside of the experimental band limits. However, the center-left panel shows that the Improved Fit algorithm largely resolves the issue and that EOM-CCSD transitions are typically overestimating rather than underestimating relative to the experimental band energies. This is largely consistent with what is expected of EOM-CCSD with a double-ζ basis set;^99,100^ better agreement between computed and experimental excitation energies would require the triples correction and/or a larger basis set, but those would not be tractable for the systems studied here. It is also known in some cases that including explicit solvent molecules close to the solute can shift the position of transitions with respect to PCM-only calculations, potentially improving the agreement between computed and experimental energies, especially if molecular dynamics is used to sample the position of solvent molecules and/or counterions.^101−105^

TD (or TDA) DFT transitions mostly fall within the band limits, as reported also in the Supporting Information of ref (12). The Improved Fit algorithm makes a few changes in the band assignments, but those changes do not significantly affect the energetic error metrics.

The three panels on the right of Figure 2 indicate the values of the slope, y-intercept, and *R*^2^ for the linear regression between computed and experimental OSs. An excellent agreement would yield values of 1, 0, and 1, respectively, so any deviations from those values indicate differences between the computed and experimental strengths. Together with the center panel, which presents the MAE, those metrics aid in quantifying the differences in the computed and experimental OSs.

CIS and TDA DFT exhibit a strong dependence of the OS on the gauge used; the length gauge (full circle) and velocity gauge (empty square) often give OSs that can vary significantly. The mixed gauge (empty triangle) is typically in between the other two gauges. This gauge dependence is almost eliminated when using TD-HF or TD-DFT, which follow the Thomas–Reiche–Kuhn sum rule (∑_*i*_^*N*^*f*_*i*_=*N*, where N is the number of electrons in the system)^106−108^ unlike CIS and TDA.^98,109^ Similarly, EOM-CCSD has a larger gauge-dependence than LR-CCSD, but the difference is not as pronounced.

For the remainder of this section, we focus our discussion on TD-DFT (without the Tamm-Dancoff approximation) and LR-CCSD.

In ref (12), we verified that (RPA) TD-B3LYP OSs with PCM solvation improved by almost all metrics when compared against *n* · *f*_exp_ instead of just *f*_exp_. Here, we revisit this comparison focusing only on the subset of 35 VHHM transitions and applying the Improved Fit algorithm. We find, consistently with Tarleton et al.,^12^ that TD-B3LYP *f*_comp_ are overestimated relative to *f*_exp_, and are in much better agreement with *n* · *f*_exp_. This is reflected in each of the MAE, slope, y-intercept, and *R*^2^ plots in Figure 2. We note also that the relative error (compared to the average value of experimental OS) is significantly lower for *n* · *f*_exp_, as shown in Table 1.

**Table 1:** Relative MAE for TD-B3LYP Compared to the Average of the Experimental Reference^a^

framework	*f*_exp_**/***n*	*f*_exp_	*n*·*f*_exp_
Exact Band Limits	84.3%	34.4%	11.8%
Improved Fit	60.1%	26.1%	9.3%

a We use the average of the three gauges since TD-B3LYP does not exhibit strong gauge dependence.

Within the Exact Band Limits framework, the metrics for MAE and slope are best for *n* · *f*_exp_ and worst for *f*_exp_/*n*. *R*^2^ and *y*-intercept are instead comparable for *f*_exp_/*n*, *f*_exp_, and *n* · *f*_exp_. However, application of the Improved Fit algorithm, which reduces the MAE, improves the agreements with *n* · *f*_exp_ by almost all metrics; it results in a slight improvement in *R*^2^, minimizes the y-intercept, and reduces the absolute error in energy |Δ*E*|. Meanwhile, applying the same Improved Fit algorithm when comparing to *f*_exp_ and *f*_exp_/*n* yields a limited improvement or even a worst agreement (in terms of *R*^2^, y-intercept, and |Δ*E*|) compared to the Exact Band Limits framework.

Due to the stronger gauge-dependence of EOM-CCSD (which is only partially but not fully resolved with LR-CCSD), it is more difficult to draw conclusions about which solvent correction (*f*_exp_/*n*, *f*_exp_, or *n* · *f*_exp_) is in best agreement with the EOM-CCSD results. The MAE and other metrics in the length gauge are in best agreement with *n* · *f*_exp_. However, velocity gauge calculations give a better agreement with *f*_exp_/*n*. The mixed gauge calculations appear to have a similar error with all three gauges but agree best with *f*_exp_. Overall, we expect the results of the length gauge to be more reliable for the double-ζ basis set used.^110^

**Figure 3:**
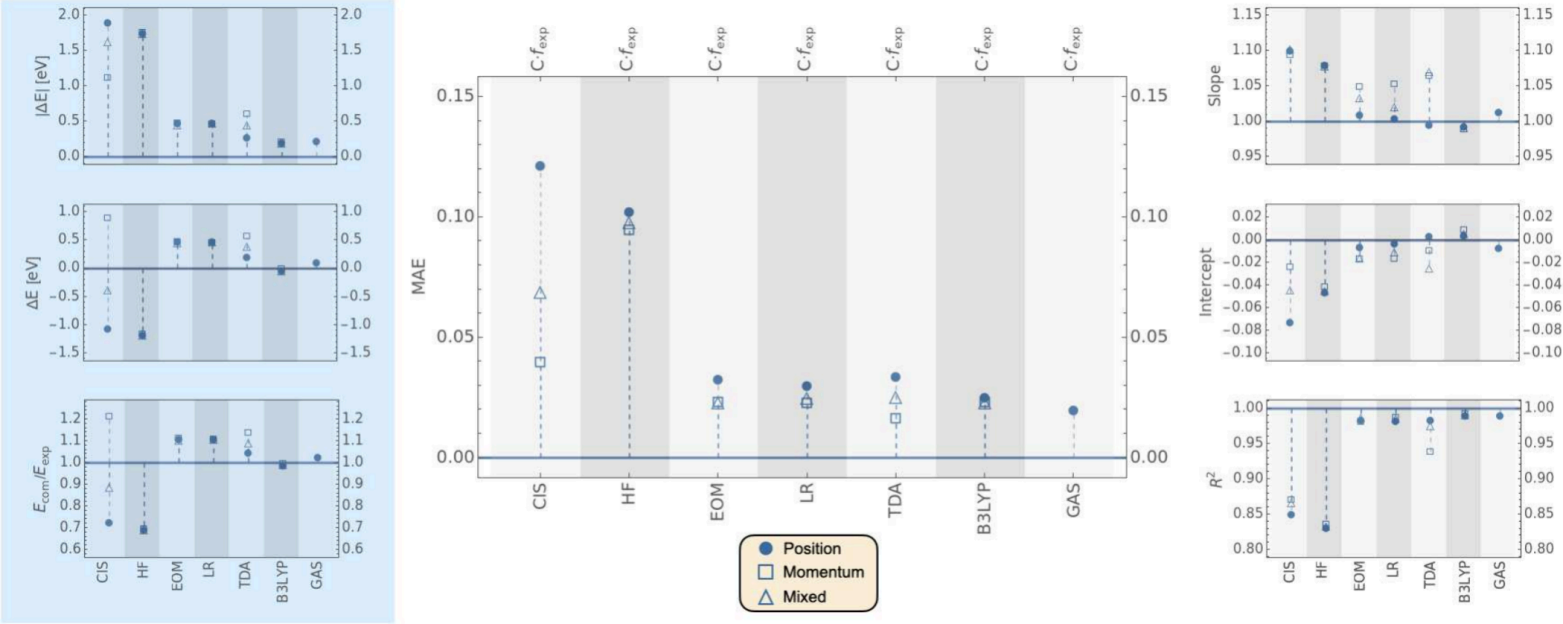
Comparison of *f* values computed using CIS/6-311++G**, TD-HF/6-311++G**, EOM-EE-CCSD/aug-cc-pVDZ, LR-CCSD/aug-cc-pVDZ, TDA-B3LYP/6-311++G**, RPA TD-B3LYP/6-311++G**, and GAS phase-RPA-TD-B3LYP/6-31+G* for a subset of 35 experimental transitions. For each method, the *f*_comp_ values are compared to *C*·*f*_exp_, where the constants *C* were obtained according to Algorithm 1 and are displayed in Table 2. A full circle corresponds to the data obtained with the length gauge. An empty square corresponds to the velocity gauge, and an empty triangle corresponds to the mixed gauge. The data displayed can be found in Tables S20 to S28 of the SI.

The fact that the length gauge OSs overestimate *f*_exp_, while the momentum gauge OSs underestimate *f*_exp_ could be partially explained by the computed transition energies being systematically larger than the experimental energies (*f*_*nm*_^*lg*^ is proportional to the transition energy while *f*_*nm*_^*vg*^ is inversely proportional to it; see eq 21 and 22.) From the *E*_comp_/*E*_exp_ plot in Figure 2 (see Figure 3 as well) EOM(LR)-CCSD computed energies appear to be ∼10% larger than the experimental energies. That said, the difference between OSs computed in different gauges seems to be larger than what can be explained by the energy overestimation, as will also become apparent during the discussion of Table 2 below.

**Table 2:**
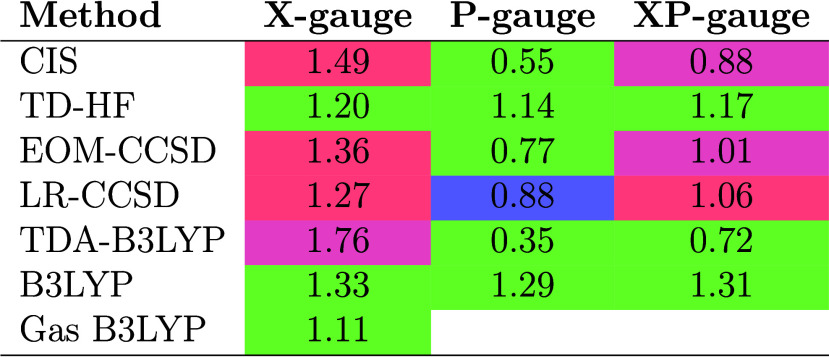
*C* Values Obtained According to Algorithm 1^a^

a The background color indicates the convergence from three different starting points for *C*. Green indicates that the same *C* value is obtained from either *C*_0_ = 0.7, 1.0, or 1.4. Red or blue indicate that the *C* value with the highest *R*^2^ comes from *C*_0_ = 1.4 or 1.0, respectively. Purple indicates that the same *C* value with the highest *R*^2^ is obtained from either *C*_0_ = 1.4 or 1.0.

The gas phase OS calculations agree better with *f*_exp_ rather than *n* · *f*_exp_, even though the experiments are carried out in solution. In other words, the reaction field effect introduced by using PCM significantly increases the computed OS. This can be traced to the individual *f*_*n*0_. For most transitions computed in this set, the *f*_*n*0_ were larger when computed using PCM. We believe that the agreement of gas-phase calculations to *f*_exp_ result from a cancellation of errors.

Next, we optimize the scaling factor “C” that relates the computed and experimental OSs by following the approach in Algorithm 1. For the electronic structure methods presented in Table 2, three initial guesses were tested: *C*_0_ = 0.7, 1.0, and 1.4. The converged *C* values resulting in the highest *R*^2^ are presented in Table 2.

Most of the data in Table 2 has a green background which indicates that the algorithm converged to the same C value independent of the starting point used (0.7, 1.0, or 1.4). Even among the values colored with a red, blue, or purple background, most converged to similar values from the different starting points. For the few exceptions to this, there usually is a poor quality fit (e.g., for CIS) and the *R*^2^ value between *C* · *f*_exp_ and *f*_comp_ gives a good indication for which value of C to trust.

The results in Table 2 again highlight the strong gauge dependence of CIS, EOM-CCSD, and TDA-B3LYP, and the slightly reduced gauge dependence of LR-CCSD. In all those cases, the length gauge gives a scaling factor larger than one and the velocity gauge gives a scaling factor smaller than one. On the other hand, the more gauge-independent TD-HF and TD-B3LYP methods systematically overestimate *f*_exp_ regardless of the gauge. It is worth noting that for any one given gauge, LR and EOM-CCSD give results that are in reasonably good agreement, as also discussed in ref (111).

The statistics for the pairs (*C* · *f*_exp_, *f*_comp_) are displayed in Figure 3. These results represent the best possible fit using a simple scaling factor, C, in combination with the Improved Fit algorithm. We find that all of EOM-CCSD, LR-CCSD, TD-DFT, and TDA-DFT can be in good agreement (MAE lower than 0.04) with experimental OS through selection of band assignments and appropriate scaling factor C. This algorithm also reduces the gauge dependence for EOM-CCSD and LR-CCSD by using a different scaling factor C for each gauge to better fit the experimental data. This is also true, to a lesser degree, for TDA-DFT, but not CIS, where the MAE still varies strongly with the gauge. Among these methods, TD-DFT with the B3LYP functional still results in the best metrics overall for the slope, y-intercept, *R*^2^, and energy errors, compared to the other methods. Meanwhile, other methods can be fit to reproduce OSs with a similarly low MAE but they either result in a worse linear regression slope and intercept or require using transitions outside of the band limits, which manifests in larger errors in the energy metrics.

**Figure 4:**
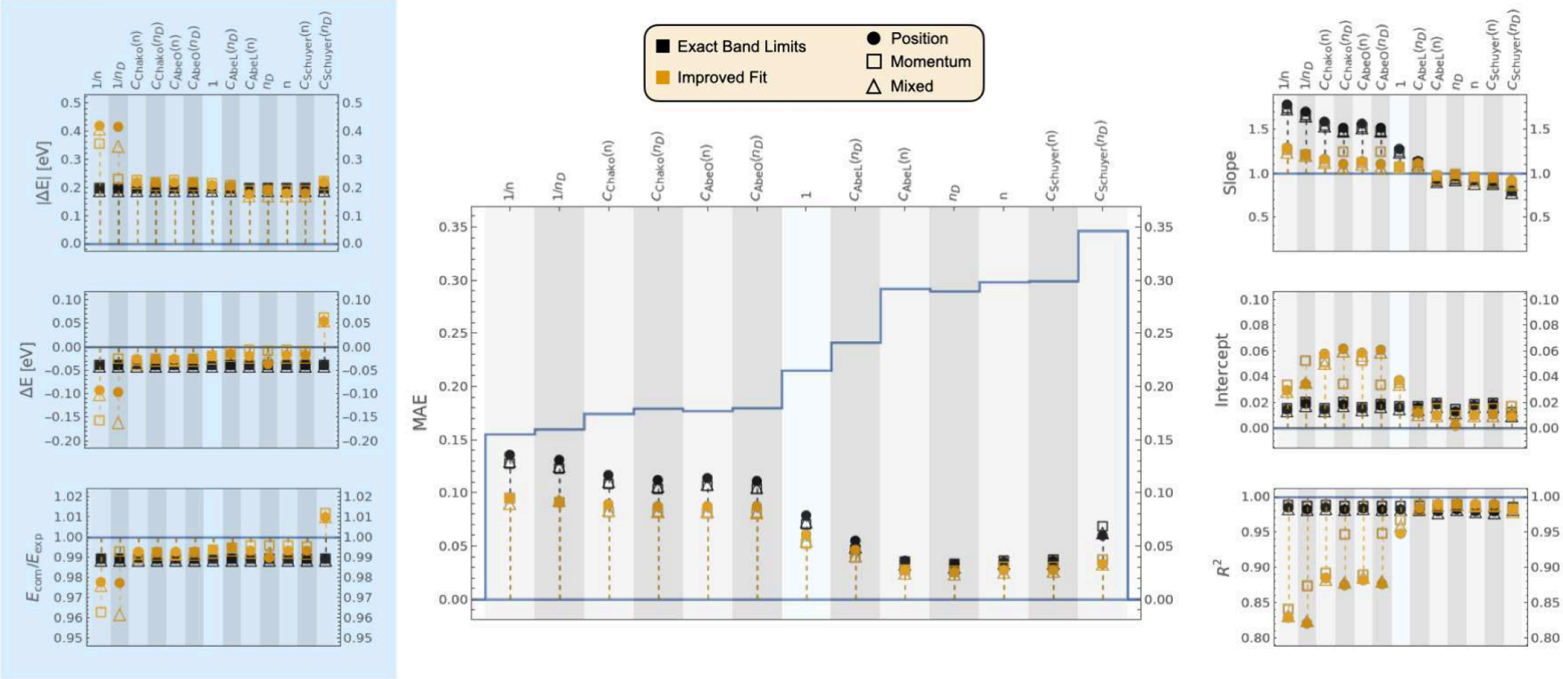
Comparison of *f* values computed using TD-B3LYP/6-311++G**/PCM for a subset of 35 experimental transitions multiplied by different cavity field corrections (listed at the top). The blue bar outline indicates the average values of *f*_exp_ multiplied by the respective cavity field factor. A full circle corresponds to the data obtained with the length gauge. An empty square corresponds to the velocity gauge, and an empty triangle corresponds to the mixed gauge. Markers in black correspond to transitions assigned using the Exact Band Limits, while markers in yellow correspond to transitions assigned using the Improved Fit algorithm. The data displayed can be found in Tables S29 to S106 of the SI.

For gauge dependent methods, the scaling factor C is a complicated function that captures multiple effects, including a correction for the gauge used. However, for gauge-independent methods like (RPA) TD-DFT, the origin of the scaling factor C may be largely attributed to the solvent effect. As discussed in the theoretical background section, the solvent impacts the absorption intensity in several ways. The reaction field [effect (ii)] is accounted for through PCM. However, the cavity field [effect (iii)] is missing. In Figure 4, we test some of the simple cavity field corrections proposed in the literature (see eq 26 to 30) using both the frequency-specific refractive index of the solvent (*n*) and the refractive index at the sodium *D*-line (*n*_*D*_). We focus at this point on TD-B3LYP calculations. Similar figures for the gas phase and for LR-CCSD are shown in SI Figure S1.

All the cavity field corrections used in Figure 4 assume a spherical cavity. For the molecules in this benchmark, we find that none of the corrections performed better than a simple multiplication by the refractive index (either *n* or *n*_*D*_). The corrections displayed to the left side of *f*_exp_ give considerably worse agreement by almost all metrics.

As shown earlier in eq 31, the factor *n* that appears in *n* · *f*_exp_ arises from the energy flux of the radiation field in a dielectric and appears in early cavity field literature corrections.^48,49^ It has been shown that more accurate cavity field corrections would need to account for the cavity shape beyond using a simple spherical approximation.^48,56^ Such corrections will be tested in future work.

A similar analysis of different cavity field corrections was carried out for LR-CCSD calculations (see SI Figure S1). The results in the length gauge, which also give an overestimation of the computed OS relative to the experimental one, largely follow the same trend as observed for TD-B3LYP.

Next, we expand the benchmark set to include all 85 VHHM transitions to compare *f* values computed using different TDDFT functionals and basis sets. Hereon, we focus on comparing the computed transitions relative to only *f*_exp_ and *n* · *f*_exp_, and no longer consider other cavity field correction terms. For reference, the average experimental OS in the set, as given by eq 12, is ⟨*f*_exp_⟩=0.3077, while ⟨*n* · *f*_exp_⟩ is 0.4333.

**Figure 5:**
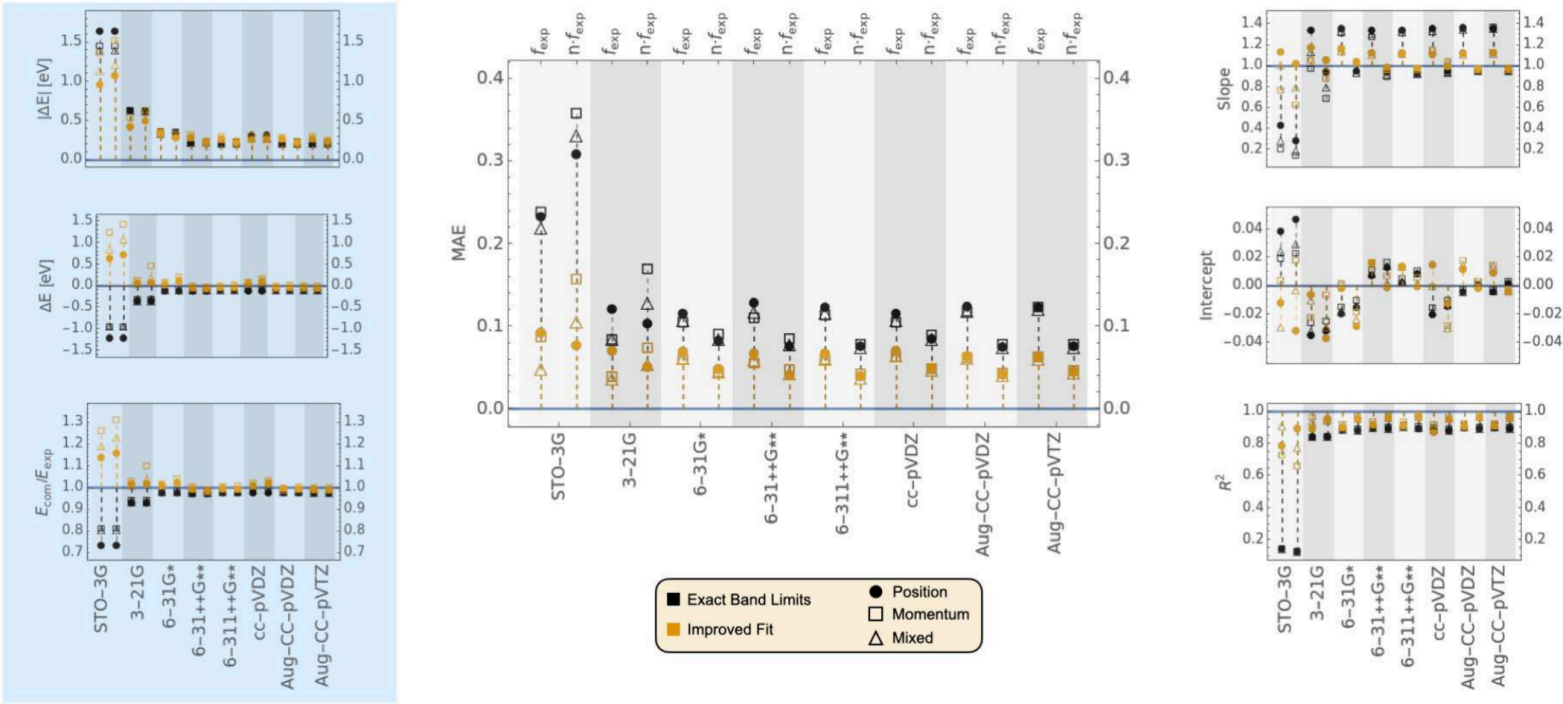
Comparison of *f* values computed using B3LYP with different basis sets to a subset of 85 experimental transitions. For each basis set the *f*_comp_ values are compared to *f*_exp_ (left) and *n*·*f*_exp_ (right). For reference, the average value of the experimental *f* values are ⟨*f*_exp_⟩ = 0.307655, and ⟨*n*·*f*_exp_⟩ = 0.433273. A full circle corresponds to the data obtained with the length gauge. An empty square corresponds to the velocity gauge, and an empty triangle corresponds to the mixed gauge. Markers in black correspond to transitions assigned using the Exact Band Limits, while markers in yellow correspond to transitions assigned using the Improved Fit algorithm. The data displayed can be found in Tables S107 to S118 of the SI.

Figure 5 presents the data for the OS calculations carried out using TD-B3LYP and different basis sets. Small basis sets exhibit a strong gauge-dependence, especially for STO-3G and 3-21G*, that is significantly reduced for larger basis sets. In general, the MAE and gauge-dependence continue to decrease with increasing basis set size above 6-31G* (see SI Figure S2 for a more detailed figure). For example, the range of MAEs for the different gauges decreases from 0.083(length)-0.089(velocity) for 6-31G* to 0.076(length)-0.083(velocity) for 6-31++G** to 0.076(length)-0.077(velocity) for 6-311++G** when not using the Improved Fit algorithm. Similarly, across the Dunning basis set series, 0.085(length)-0.088(velocity) for cc-pVDZ to 0.075(length)-0.077(velocity) for aug-cc-pVDZ to 0.076(length)-0.077(velocity) for aug-cc-pVTZ. The same trends are largely conserved when using the Improved Fit algorithm. Overall, the error introduced by the basis set is not large; the difference between *f*_comp_ values obtained with different basis sets are significantly smaller than the MAEs relative to experimental data. For example, for the length gauge and within the Exact Band Limits, ⟨|*f*_*Aug*-*cc*-*pVDZ*_ – *f*_*Aug*-*cc*-*pVTZ*_|⟩ = 0.003 while ⟨|*f*_6-311++G**_ – *f*_*Aug*-*cc*-*pVTZ*_|⟩ = 0.009.

**Figure 6:**
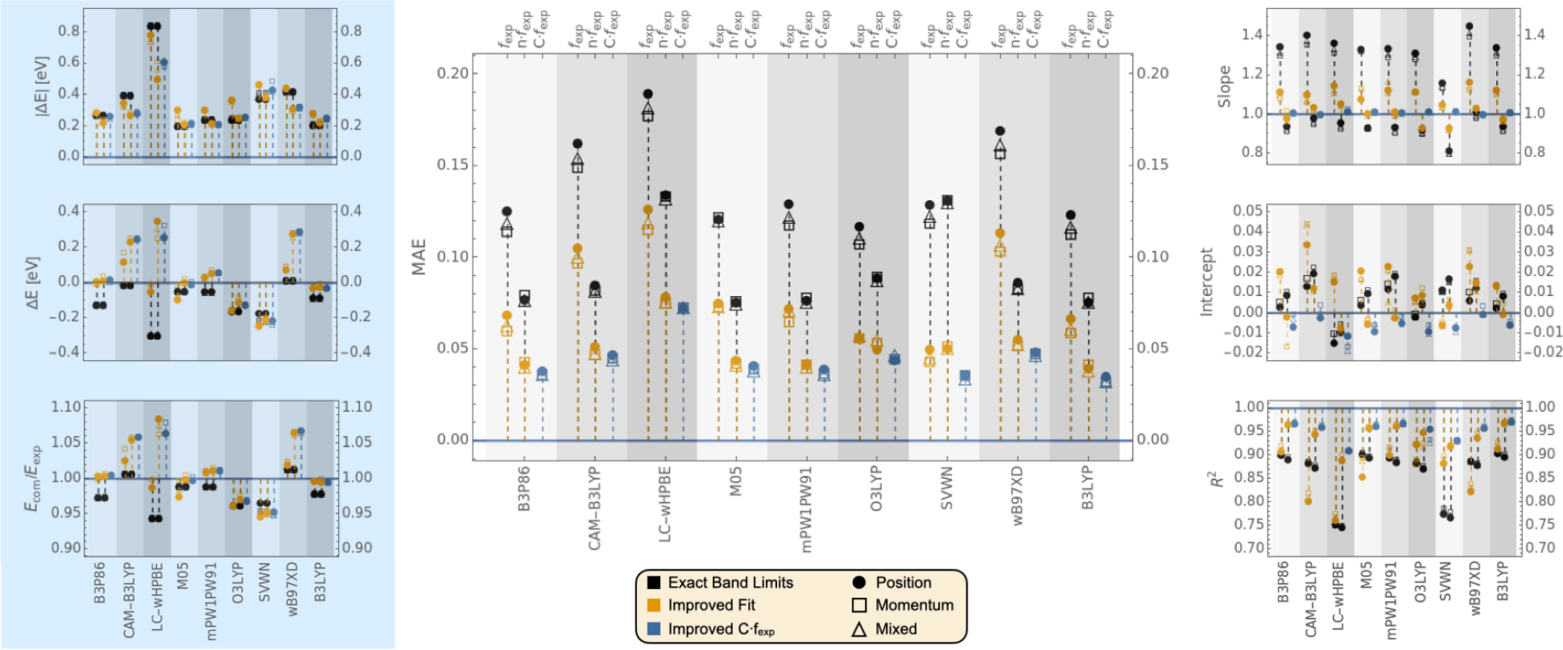
Comparison of *f* values computed using different density functionals to a subset of 85 experimental transitions. For each method, the *f*_comp_ values are compared to *f*_exp_ (left), *n*·*f*_exp_ (center), and *C*·*f*_exp_ (right). For reference, the average values of the experimental *f* values are ⟨*f*_exp_⟩ = 0.307655 and ⟨*n*·*f*_exp_⟩ = 0.433273. A full circle corresponds to the data obtained with the length gauge. An empty square corresponds to the velocity gauge, and an empty triangle corresponds to the mixed gauge. Markers in black correspond to transitions assigned using the Exact Band Limits, while markers in yellow correspond to transitions assigned using the Improved Fit algorithm. Blue markers are used for the iterative comparison to *C*·*f*_exp_ The data displayed can be found in Tables S119 to S133 of the SI.

Figure 6 compares the OS errors relative to *f*_exp_, *n* · *f*_exp_, and *C* · *f*_exp_ for a series of 9 TD-DFT functionals (one pure, five hybrid, and three long-range corrected functionals). The values of C for each gauge and each functional are shown in Table 3. The green background in Table 3 indicates that in all cases, convergence was achieved regardless of the starting value of C (0.7, 1.0, or 1.4). All functionals behave consistently with TD-B3LYP and overestimate the OS relative to *f*_exp_. A weak gauge-dependence is observed in all cases. On the other hand, the TDA equivalents, shown in Table S275 to S292 and Figure S5 of the Supporting Information, display much stronger gauge-dependence consistent with TDA-B3LYP.

**Table 3:**
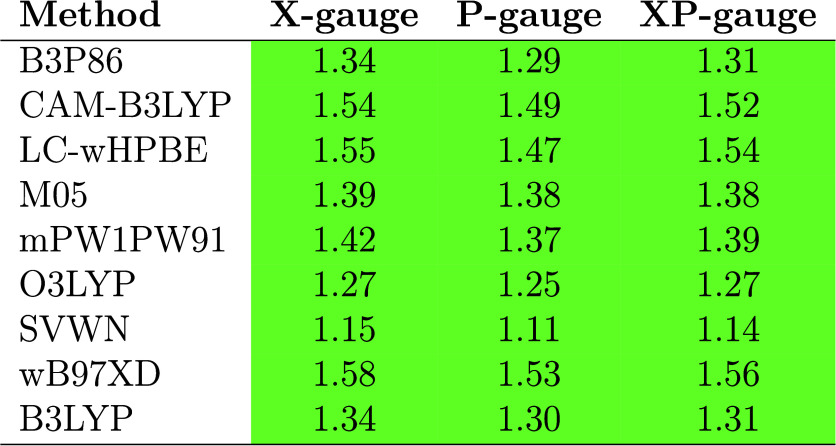
*C* values Obtained According to Algorithm 1 for the Nine Functionals Considered

Hybrid functionals without long-range corrections give an optimal *C* value in the range of 1.25–1.42. the pure functional SVWN gives an optimal *C* value in the range of 1.11–1.15. Long-range functionals give optimal C values in the range 1.47–1.58. Most of those factors, especially for the hybrid functionals, are close to the refractive index of solvents, so the agreement with *n* · *f*_exp_ is usually better than the agreement with just *f*_exp_, with the exception of SVWN. B3LYP still gives the best agreement with *n* · *f*_exp_, although a few other hybrid functionals are close.

Out of the 85 transitions that belong to the VHHM subset, 43 come from molecules whose ground state symmetry point group is *C*_1_, 30 come from molecules of point group *C*_*s*_, and 12 come from molecules of point groups of higher symmetry (one from *D*_2*h*_, two from *C*_2*h*_, two from *D*_2_, four from *C*_2*v*_, and three from *C*_2_). Of the 85 B3LYP transitions, 54 have ππ* character, 7 are ππ* with significant charge transfer character, and 21 are mixed, containing Ry or diffuse character in addition to ππ*. In the reference experimental data, 32 of the 85 transitions were measured in nonpolar solvents, 50 in protic solvents, and 3 in polar aprotic solvents. We carry out further statistical analyses on these subsets of data in Figures S3 and S4 of the Supporting Information. We find that the trends observed overall for the 85 transitions are largely reproduced by the subsets if they have a large enough sample size. In other words, we do not identify significantly different trends for molecules of different symmetry, excitation character, or solvent polarity.

## 5 Conclusions

In a previous study, Tarleton et al. derived experimental oscillator strengths from well-defined UV–visible absorption spectral peaks of 100 molecules in solution.^12^ Here, we use a subset of transitions identified as having reliable experimental strengths, based on the reproducibility and quality of their deconvolution and having little overlap with other peaks, to further benchmark several wave function methods, density functionals, basis sets, transition dipole gauges (length, velocity, and mixed), and solvent corrections. A band-matching algorithm is used to assign computed transitions to experimental peaks.

Large errors and gauge-dependence were observed and quantified in oscillator strengths computed with CIS or TD-DFT paired with the Tamm-Dancoff approximation (TDA). These theories, which do not satisfy the Thomas–Reiche–Kuhn sum rule, gave oscillator strengths that do not match well with the experimental data. Linear response methods like TD-DFT and TD-HF (RPA) showed much smaller gauge dependence. TD-DFT calculations resulted in mean errors that are less than half of those observed in the best TDA cases.

The size of the molecules (average molecule weight = 160 g/mol for the molecules included in the 85 VHHM transitions) made systematic calculations using high-level wave function methods and large basis sets intractable. Instead, we opted to run EOM-CCSD and LR-CCSD calculations with the aug-cc-pVDZ basis set on a subset of 35 transitions. Overall, EOM-CCSD calculations also exhibited a strong gauge-dependence which was only slightly reduced with LR-CCSD.

In general, we find that an increase in the size of the basis set resulted both in smaller gauge-dependence and smaller mean errors relative to the experimental data.

Several functionals were benchmarked in addition to TD-B3LYP. In all cases, the oscillator strengths were overestimated relative to the experiments, but the degree of this overestimation depends on the class of functional used. A pure functional only overestimated *f*_exp_ by a factor of around 1.1, while hybrid functionals had a larger factor ranging from 1.25 to 1.4. Long range corrected functionals gave the largest factor relative to *f*_exp_, up to 1.58. The EOM-CCSD and LR-CCSD in the length gauge also overestimate the data by a similar factor as hybrid functionals, close to 1.3.

The systematic overestimation of most computational methods compared to *f*_exp_ is consistent with the refractive index factor that appears in the denominator of several theoretical solvent effect corrections. This factor arises from the energy flux of the radiation field in a dielectric (eq 31). Through testing several simple cavity field corrections, we find that factors derived using a spherical cavity do not improve the agreement between computations and experiments. As highlighted by several studies,^48,56^ a suitable cavity field correction can be obtained by using a cavity shaped to the dimensions of the molecule and that considers the direction of the transition dipole moment relative to that cavity. While these effects can be explored further, in the meantime, we find that simply multiplying the experimental oscillator strength by the solvent refractive index, which is equivalent to assuming that the cavity field acting on the molecules is equal to the macroscopic (averaged) field, gives a reasonably good agreement with computed oscillator strengths for TD-DFT/PCM methods, especially when using a hybrid functional. For example, in the case of TD-B3LYP, the error when comparing *f*_comp_ and *n* · *f*_exp_ is on the order of 0.02, which represents 9–12% of the actual magnitude of the experimental strength, depending on how band assignments are made.

## Supporting Information Available

Structures of the 100 molecules from Tarleton et al. reoptimized with the 6-311++G** basis set (ZIP)
Tables with the numerical values for the figures shown in the Results and Discussion section of this work; tables and figures of cavity field correction results; tables of results for all 164 transitions with nine functionals and 6-311++G**; tables and figures with TDA-DFT calculations for nine functionals with the 6-311++G** basis set; tables and figures summarizing the results of oscillator strength calculations for different subsets of the data divided according to symmetry, transition character, solvent, and spectrophotometer used (PDF)
