# Supplementary material for: Absorption Intensities of Organic Molecules from Electronic Structure Calculations versus Experiments: the Effect of Solvation, Method, Basis Set, and Transition Moment Gauge
Source: J Chem Theory Comput. 2024 Aug 14;20(16):7227–43. doi: 10.1021/acs.jctc.4c00642 (PMC11360136; doi:10.1021/acs.jctc.4c00642)
Supplement: Supplementary file 2 — ct4c00642_si_002.pdf [file ct4c00642_si_002.pdf]

Supporting Information for manuscript: Absorption Intensities of Organic Molecules from Electronic Structure Calculations versus Experiments: The Effect of Solvation, Method, Basis Set, and Transition Moment Gauge

Jorge C. Garcia-Alvarez* and Samer Gozem*

Department of Chemistry, Georgia State University, Atlanta, Georgia 30302, United States

E-mail: jgarciaalvarez1@student.gsu.edu; sgozem@gsu.edu

Contents

S1 Subsets of transitions to be analyzed **S5**

S2 Tables with the numerical values of the statistics depicted in section: Results and Discussion **S5**

S2.1 Wave function method comparisons for subset of 35 transitions S5

S2.1.1 Wave function methods vs. f_{exp} S5

S2.1.2 Wave function methods vs. $(n \cdot f_{exp})$ S6

S2.1.3 Wave function methods vs. (f_{exp}/n) S8

S2.1.4 Wave function methods vs. Cf_{exp} calculated from $C_0 = 1$ S9

S2.1.5 Wave function methods vs. Cf_{exp} calculated from $C_0 = 1.4$ S10

S2.1.6 Wave function methods vs. Cf_{exp} calculated from $C_0 = 0.7$ S10

S2.2 Cavity Field Corrections S11

S2.2.1 $f_{exp}/n(\tilde{\nu})$ S11

S2.2.2 f_{exp}/n_D S12

S2.2.3 $C_{Chako}(n)f_{exp}$ S13

S2.2.4 $C_{Chako}(n_D)f_{exp}$ S14

S2.2.5 $C_{AbeO}(n)f_{exp}$ S15

S2.2.6 $C_{AbeO}(n_D)f_{exp}$ S17

S2.2.7 f_{exp} S18

S2.2.8 $C_{AbeL}(n_D)f_{exp}$ S19

S2.2.9 $C_{AbeL}(n)f_{exp}$ S20

S2.2.10 n_Df_{exp} S21

S2.2.11 $n(\tilde{\nu})f_{exp}$ S23

S2.2.12 $C_{Schuyer}(n)f_{exp}$ S24

S2.2.13 $C_{Schuyer}(n_D)f_{exp}$ S25

S2.3 VHHM transitions for TD-B3LYP with different basis sets S28

S2.3.1 f_{exp} S28

S2.3.2 nf_{exp} S30

S2.4 VHHM transitions with the 9 functionals and 6-311++G** S33

S2.4.1 f_{exp} S33

S2.4.2 nf_{exp} S34

S2.4.3 Cf_{exp} S36

S3 Other Results not presented in the manuscript **S37**

S3.1 All 164 Transitions with 9 functionals and 6-311++G** S37

S3.1.1 f_{exp} S37

S3.1.2 nf_{exp} S38

S3.2 VHHM ∩ pi-pi* S40

S3.2.1 f_{exp} S40

S3.2.2 nf_{exp} S41

S3.2.3 Cf_{exp} S42

S3.3 VHHM ∩ Mixed S43

S3.3.1 f_{exp} S43

S3.3.2 nf_{exp} S44

S3.4 VHHM ∩ pi-pi* (CT) S45

S3.4.1 f_{exp} S45

S3.4.2 nf_{exp} S47

S3.5 VH S48

S3.5.1 f_{exp} S48

S3.5.2 nf_{exp} S50

S3.6 Spectrophotometers S52

S3.6.1 f_{exp} S52

S3.7 Different Solvents S53

S3.7.1 f_{exp} S53

S3.7.2 Solvent: Non-polar S56

S3.7.3 f_{exp} S56

S3.7.4 nf_{exp} S57

S3.7.5 Solvent: Polar S58

S3.7.6 f_{exp} S58

S3.7.7 nf_{exp} S59

S3.8 Different Point Groups of symmetry S60

S3.8.1 Point Group: C_1 ∩ VHHM S60

S3.8.2 f_{exp} S60

S3.8.3 nf_{exp} S62

S3.8.4 Point Group: $C_s \cap$ VHHM S63

S3.8.5 f_{exp} S63

S3.8.6 nf_{exp} S64

S3.8.7 Point Group: Higher than C_s (D_{2h}, C_{2h}, D_2, C_{2v}, and C_2) $\cap$ VHHM . S65

S3.8.8 f_{exp} S65

S3.8.9 nf_{exp} S67

S3.9 TDA/TD-DFT/PCM: VHHM transitions S69

S3.9.1 f_{exp} VHHM transitions S69

S3.9.2 nf_{exp} VHHM transitions S70

S3.9.3 f_{exp}/n VHHM transitions S71

S3.9.4 $C_0 = 1.0\ Cf_{exp}$ VHHM transitions S73

S3.9.5 $C_0 = 1.4\ Cf_{exp}$ VHHM transitions S74

S3.9.6 $C_0 = 0.7\ Cf_{exp}$ VHHM transitions S74

S1 Subsets of transitions to be analyzed

Table S1: Subset of experimental transition ID numbers and molecule ID numbers used in this work. The full set and corresponding ID numbers can be found in Tarleton et al., J. Phys. Chem. A 2022, 126, 3, 435–443.

Category	Molecule IDs	Transition IDs
VHHM	11, 12, 13, 16, 2, 20, 22, 23, 24, 26, 27, 28, 29, 30, 31, 32, 33, 34, 36, 37, 39, 4, 42, 44, 45, 46, 47, 48, 49, 50, 53, 54, 55, 56, 57, 58, 59, 60, 61, 62, 63, 64, 66, 67, 68, 69, 70, 71, 72, 73, 74, 75, 76, 77, 78, 80, 82, 83, 87, 88, 89, 90, 91, 92, 93, 94, 96, 97, 98 (N = 69)	3, 5, 12, 13, 15, 16, 21, 25, 27, 29, 30, 32, 33, 34, 35, 36, 38, 39, 41, 42, 43, 44, 45, 47, 49, 51, 55, 59, 60, 61, 62, 63, 65, 68, 70, 71, 78, 79, 81, 84, 86, 87, 88, 89, 90, 91, 92, 93, 94, 95, 98, 100, 101, 103, 106, 109, 111, 113, 114, 115, 117, 118, 119, 121, 122, 123, 124, 125, 127, 130, 131, 134, 137, 144, 145, 147, 149, 150, 151, 152, 153, 155, 158, 159, 161 (N = 85)
Subset used to benchmark wavefunction methods	12, 13, 34, 46, 47, 49, 53, 55, 56, 57, 58, 59, 60, 61, 62, 66, 68, 69, 70, 80, 87, 88, 89, 90, 92, 93 (N = 26)	13, 15, 16, 45, 63, 65, 70, 78, 79, 84, 86, 87, 88, 89, 90, 91, 92, 93, 94, 95, 98, 106, 111, 113, 114, 115, 130, 131, 144, 145, 147, 149, 150, 152, 153 (N = 35)

S2 Tables with the numerical values of the statistics depicted in section: Results and Discussion

S2.1 Wave function method comparisons for subset of 35 transitions

S2.1.1 Wave function methods vs. f_{exp}

Avg $f_{exp} = 0.215319$

Table S2: f_{comp} vs f_{exp} /x-gauge/Exact Band Limits/

	CIS	HF	EOM-CCSD	LR	TDA-B3LYP	B3LYP	GAS
MAE	0.169904	0.13687	0.0968929	0.09107	0.160716	0.07839	0.0367631
Slope	0.662698	1.18924	0.740419	0.703667	1.74979	1.27771	1.08301
Intercept	-0.00745127	-0.0731274	0.0529283	0.0502075	-0.00725006	0.0157681	0.00478047
R^2	0.372657	0.738129	0.598033	0.588269	0.968795	0.982224	0.966718
$\|\Delta E\|$	2.29697	2.04943	0.987765	0.987842	0.366594	0.194237	0.226343
ΔE	-1.96982	-1.77584	-0.369483	-0.36936	-0.0226967	-0.0383761	0.0994301
E_{comp}/E_{exp}	0.490587	0.541302	0.930338	0.930367	0.998425	0.989187	1.02293

Table S3: f_{comp} vs f_{exp} /p-gauge/Exact Band Limits/

	CIS	HF	EOM-CCSD	LR	TDA-B3LYP	B3LYP
MAE	0.173013	0.13073	0.0956643	0.0814786	0.162653	0.0696729
Slope	0.251073	1.12217	0.480595	0.537328	0.326803	1.2339
Intercept	-0.011755	-0.0660497	0.0166304	0.0251118	-0.0177009	0.016348
R^2	0.324247	0.733311	0.57832	0.583212	0.796121	0.984933
$\|\Delta E\|$	2.29919	2.04936	0.991367	0.990235	0.357805	0.193666
ΔE	-1.96759	-1.77598	-0.366188	-0.367438	-0.0293323	-0.0396048
E_{comp}/E_{exp}	0.491051	0.541269	0.931054	0.930782	0.997096	0.988917

Table S4: f_{comp} vs f_{exp} /xp-gauge/Exact Band Limits/

	CIS	HF	EOM-CCSD	LR	TDA-B3LYP	B3LYP
MAE	0.153044	0.133719	0.0777414	0.0789586	0.0940329	0.0739043
Slope	0.401892	1.15521	0.596806	0.617186	0.730062	1.2555
Intercept	-0.0149434	-0.0696029	0.0297279	0.0363542	-0.0359101	0.0159667
R^2	0.362759	0.73596	0.594933	0.587986	0.906666	0.983751
$\|\Delta E\|$	2.29856	2.04941	0.989803	0.989014	0.361076	0.193965
ΔE	-1.96823	-1.77592	-0.367502	-0.368387	-0.0231371	-0.0390178
E_{comp}/E_{exp}	0.490931	0.541283	0.93077	0.930576	0.998372	0.989044

Improving fit:

Table S5: f_{comp} vs f_{exp} /x-gauge/Improved Fit/

	CIS	HF	EOM-CCSD	LR	TDA-B3LYP	B3LYP	GAS
MAE	0.132056	0.0986729	0.0514871	0.04099	0.122001	0.0603243	0.0219836
Slope	1.17437	1.26365	1.11949	1.0567	0.914202	1.07543	1.03257
Intercept	-0.0781381	-0.0544109	0.0181092	0.0184981	0.0759924	0.0371228	0.00265326
R^2	0.740616	0.861835	0.955228	0.952296	0.635312	0.94819	0.984277
$\|\Delta E\|$	1.90773	1.74025	0.447709	0.447399	0.386855	0.206482	0.22106
ΔE	-1.24425	-0.957866	0.441212	0.440949	0.00615745	-0.0205926	0.0989957
E_{comp}/E_{exp}	0.674487	0.746724	1.1037	1.10365	1.00149	0.993425	1.02285

Table S6: f_{comp} vs f_{exp} /p-gauge/Improved Fit/

	CIS	HF	EOM-CCSD	LR	TDA-B3LYP	B3LYP
MAE	0.0841014	0.09799	0.0399071	0.0282986	0.0794786	0.0518471
Slope	0.638743	1.21241	0.86259	0.935483	0.44178	1.06175
Intercept	0.00313257	-0.0328891	-0.00488889	-0.00751536	0.0427395	0.0341303
R^2	0.799733	0.848669	0.949899	0.978309	0.564254	0.964913
$\|\Delta E\|$	1.43567	1.56284	0.567267	0.508896	1.10956	0.210964
ΔE	1.0322	-0.903858	0.560463	0.501974	1.06151	-0.0212969
E_{comp}/E_{exp}	1.23328	0.761059	1.13215	1.11735	1.24869	0.99323

Table S7: f_{comp} vs f_{exp} /xp-gauge/Improved Fit/

	CIS	HF	EOM-CCSD	LR	TDA-B3LYP	B3LYP
MAE	0.0752071	0.103016	0.0238643	0.0259557	0.0481329	0.0562329
Slope	1.02741	1.24716	1.04637	1.0371	0.834349	1.07764
Intercept	-0.0568984	-0.0352529	-0.015862	-0.00791798	-0.00500505	0.0348819
R^2	0.840791	0.847384	0.986629	0.977422	0.934833	0.960506
$\|\Delta E\|$	1.50348	1.56285	0.45246	0.45366	0.640977	0.210943
ΔE	-0.0464414	-0.903854	0.445905	0.44701	0.615189	-0.0213947
E_{comp}/E_{exp}	0.968179	0.76106	1.10368	1.10431	1.15148	0.993209

S2.1.2 Wave function methods vs. $(n \cdot f_{exp})$

Avg $nf_{exp} = 0.298506$

Table S8: f_{comp} vs nf_{exp} /x-gauge/Exact Band Limits/

	CIS	HF	EOM-CCSD	LR	TDA-B3LYP	B3LYP	GAS
MAE	0.20213	0.154428	0.107214	0.110339	0.0875343	0.035152	0.0716377
Slope	0.475656	0.854469	0.524738	0.498576	1.25311	0.915508	0.77605
Intercept	-0.00674633	-0.0721272	0.0557168	0.0528919	-0.0045502	0.017598	0.0063167
R^2	0.372771	0.73989	0.58322	0.573433	0.96476	0.979147	0.963813
$\|\Delta E\|$	2.29697	2.04943	0.987765	0.987842	0.366594	0.194237	0.226343
ΔE	-1.96982	-1.77584	-0.369483	-0.36936	-0.0226967	-0.0383761	0.0994301
E_{comp}/E_{exp}	0.490587	0.541302	0.930338	0.930367	0.998425	0.989187	1.02293

Table S9: f_{comp} vs nf_{exp} /p-gauge/Exact Band Limits/

	CIS	HF	EOM-CCSD	LR	TDA-B3LYP	B3LYP
MAE	0.256201	0.157454	0.178395	0.157698	0.245841	0.0354469
Slope	0.179256	0.806478	0.340916	0.380948	0.234669	0.884281
Intercept	-0.0112033	-0.0651644	0.0183459	0.0270932	-0.0173843	0.018065
R^2	0.320922	0.735417	0.565044	0.569188	0.797069	0.982221
$\|\Delta E\|$	2.29919	2.04936	0.991367	0.990235	0.357805	0.193666
ΔE	-1.96759	-1.77598	-0.366188	-0.367438	-0.0293323	-0.0396048
E_{comp}/E_{exp}	0.491051	0.541269	0.931054	0.930782	0.997096	0.988917

Table S10: f_{comp} vs nf_{exp} /xp-gauge/Exact Band Limits/

	CIS	HF	EOM-CCSD	LR	TDA-B3LYP	B3LYP
MAE	0.226915	0.155899	0.140275	0.129864	0.177221	0.0352389
Slope	0.287601	0.830117	0.423194	0.437415	0.523942	0.899681
Intercept	-0.0142593	-0.068661	0.0319053	0.0386747	-0.0351143	0.0177396
R^2	0.360711	0.737892	0.580842	0.573456	0.90672	0.980853
$\|\Delta E\|$	2.29856	2.04941	0.989803	0.989014	0.361076	0.193965
ΔE	-1.96823	-1.77592	-0.367502	-0.368387	-0.0231371	-0.0390178
E_{comp}/E_{exp}	0.490931	0.541283	0.93077	0.930576	0.998372	0.989044

Improving fit:

Table S11: f_{comp} vs nf_{exp} /x-gauge/Improved Fit/

	CIS	HF	EOM-CCSD	LR	TDA-B3LYP	B3LYP	GAS
MAE	0.129013	0.109366	0.0362503	0.0398846	0.0613286	0.0272994	0.0394785
Slope	1.17413	1.07467	0.988114	0.929664	1.06443	0.957696	0.865592
Intercept	-0.0767864	-0.0726422	-0.00400958	-0.00148763	0.0370552	0.00988454	0.00571613
R^2	0.848087	0.8633	0.981245	0.979275	0.949683	0.988671	0.975999
$\|\Delta E\|$	1.90394	1.74245	0.479557	0.478801	0.287564	0.176893	0.261766
ΔE	-1.05934	-1.1559	0.47306	0.47235	0.185994	-0.0174046	0.165961
E_{comp}/E_{exp}	0.725429	0.692744	1.10982	1.10972	1.04204	0.993299	1.03737

Table S12: f_{comp} vs nf_{exp} /p-gauge/Improved Fit/

	CIS	HF	EOM-CCSD	LR	TDA-B3LYP	B3LYP
MAE	0.0956406	0.119363	0.0893423	0.0734251	0.123598	0.0292554
Slope	0.524879	1.0227	0.629039	0.720725	0.313693	0.940066
Intercept	0.0708233	-0.0786023	0.0315107	0.0149362	0.0827093	0.013913
R^2	0.662673	0.828307	0.863544	0.915289	0.510134	0.9856
$\|\Delta E\|$	1.67797	1.73986	0.82887	0.66787	1.35296	0.18724
ΔE	1.67797	-1.16365	0.822066	0.660948	1.35176	-0.00882724
E_{comp}/E_{exp}	1.39869	0.691794	1.18969	1.15356	1.31962	0.995247

Table S13: f_{comp} vs nf_{exp} /xp-gauge/Improved Fit/

	CIS	HF	EOM-CCSD	LR	TDA-B3LYP	B3LYP
MAE	0.09862	0.10635	0.0610114	0.0541206	0.0901097	0.0270011
Slope	0.84272	1.05456	0.798407	0.85094	0.630628	0.938197
Intercept	-0.0406201	-0.0688048	0.00310759	-0.00288528	0.0285736	0.0106024
R^2	0.86647	0.863881	0.953888	0.958752	0.800771	0.989785
$\|\Delta E\|$	1.25044	1.7447	0.54626	0.53152	0.851003	0.176536
ΔE	0.829897	-1.15365	0.539706	0.52487	0.81993	-0.0180902
E_{comp}/E_{exp}	1.18972	0.693293	1.12583	1.12278	1.19621	0.993157

S2.1.3 Wave function methods vs. (f_{exp}/n)

Avg $f_{exp}/n = 0.155384$

Table S14: f_{comp} vs f_{exp}/n /x-gauge/Exact Band Limits/

	CIS	HF	EOM-CCSD	LR	TDA-B3LYP	B3LYP	GAS
MAE	0.149415	0.147663	0.113887	0.10583	0.217184	0.135498	0.0851869
Slope	0.922161	1.65328	1.04365	0.992072	2.44061	1.7812	1.50967
Intercept	-0.00804941	-0.0739563	0.0501879	0.0475675	-0.00972101	0.0141126	0.00339388
R^2	0.372109	0.735642	0.612711	0.602986	0.971934	0.984348	0.968671
$\|\Delta E\|$	2.29697	2.04943	0.987765	0.987842	0.366594	0.194237	0.226343
ΔE	-1.96982	-1.77584	-0.369483	-0.36936	-0.0226967	-0.0383761	0.0994301
E_{comp}/E_{exp}	0.490587	0.541302	0.930338	0.930367	0.998425	0.989187	1.02293

Table S15: f_{comp} vs f_{exp}/n /p-gauge/Exact Band Limits/

	CIS	HF	EOM-CCSD	LR	TDA-B3LYP	B3LYP
MAE	0.114324	0.141134	0.0596124	0.0654495	0.102719	0.126644
Slope	0.351243	1.55966	0.676782	0.7571	0.454548	1.71978
Intercept	-0.012272	-0.0667723	0.0149501	0.0231671	-0.0179639	0.0148014
R^2	0.327242	0.730481	0.591407	0.597079	0.79423	0.986678
$\|\Delta E\|$	2.29919	2.04936	0.991367	0.990235	0.357805	0.193666
ΔE	-1.96759	-1.77598	-0.366188	-0.367438	-0.0293323	-0.0396048
E_{comp}/E_{exp}	0.491051	0.541269	0.931054	0.930782	0.997096	0.988917

Table S16: f_{comp} vs f_{exp}/n /xp-gauge/Exact Band Limits/

	CIS	HF	EOM-CCSD	LR	TDA-B3LYP	B3LYP
MAE	0.117534	0.144306	0.074027	0.0816036	0.0493846	0.130916
Slope	0.560927	1.60577	0.840751	0.869918	1.01605	1.75007
Intercept	-0.0155678	-0.0703778	0.0275919	0.034074	-0.0365921	0.0143661
R^2	0.364411	0.733302	0.608856	0.602381	0.905596	0.985688
$\|\Delta E\|$	2.29856	2.04941	0.989803	0.989014	0.361076	0.193965
ΔE	-1.96823	-1.77592	-0.367502	-0.368387	-0.0231371	-0.0390178
E_{comp}/E_{exp}	0.490931	0.541283	0.93077	0.930576	0.998372	0.989044

Improving fit:

Table S17: f_{comp} vs f_{exp}/n /x-gauge/Improved Fit/

	CIS	HF	EOM-CCSD	LR	TDA-B3LYP	B3LYP	GAS
MAE	0.11702	0.101842	0.0890203	0.0829582	0.117172	0.0951031	0.0532096
Slope	0.604157	1.33398	0.601204	0.695335	0.879371	1.28434	1.15354
Intercept	-0.00310514	-0.0426717	0.0619337	0.0644272	0.00606513	0.0293796	0.0268892
R^2	0.421601	0.739079	0.480764	0.548505	0.434111	0.828696	0.925252
$\|\Delta E\|$	2.09624	1.86667	0.871208	0.68208	0.888192	0.417638	0.258943
ΔE	-1.47292	-1.26284	-0.0560664	0.185225	-0.243077	-0.0938946	0.129914
E_{comp}/E_{exp}	0.609986	0.665456	0.976074	1.04009	0.938365	0.977769	1.03032

Table S18: f_{comp} vs f_{exp}/n /p-gauge/Improved Fit/

	CIS	HF	EOM-CCSD	LR	TDA-B3LYP	B3LYP
MAE	0.0491843	0.103328	0.021934	0.0285255	0.0460672	0.0938898
Slope	0.866387	1.23252	1.04744	1.10664	0.598469	1.25275
Intercept	-0.0226087	-0.0444089	-0.0108612	0.000108919	0.0181644	0.0329729
R^2	0.851659	0.703707	0.97051	0.959659	0.746961	0.839007
$\|\Delta E\|$	1.31349	1.86505	0.4491	0.452378	1.04619	0.352537
ΔE	0.892945	-1.26453	0.442296	0.444816	1.02229	-0.159577
E_{comp}/E_{exp}	1.20448	0.665143	1.10424	1.10445	1.24677	0.96231

Table S19: f_{comp} vs f_{exp}/n /xp-gauge/Improved Fit/

	CIS	HF	EOM-CCSD	LR	TDA-B3LYP	B3LYP
MAE	0.073615	0.105763	0.045001	0.0540639	0.0259705	0.0913069
Slope	1.21144	1.26372	1.24134	1.28381	1.07037	1.25878
Intercept	-0.0360268	-0.0438996	0.000985969	-0.0029329	-0.0247108	0.0295628
R^2	0.830541	0.701409	0.964272	0.951245	0.97466	0.833461
$\|\Delta E\|$	1.62334	1.8566	0.449697	0.547341	0.451989	0.413593
ΔE	-0.434059	-1.27297	0.443143	0.276061	0.387591	-0.0979488
E_{comp}/E_{exp}	0.882663	0.663173	1.10409	1.0623	1.09196	0.976813

S2.1.4 Wave function methods vs. Cf_{exp} calculated from $C_0 = 1$

Comparing to Cf_{exp}, where C is a constant iteratively obtained to minimize MAE after improving fit

Table S20: f_{comp} vs Cf_{exp} PCM/x-gauge/Improved Fit/

	CIS	HF	EOM-CCSD	LR	TDA-B3LYP	B3LYP	GAS
MAE	0.1318	0.102217	0.036048	0.0350337	0.0337122	0.0247112	0.0196204
Slope	1.15894	1.0795	0.983802	0.979939	0.995839	0.99307	1.01201
Intercept	-0.0781381	-0.0464262	0.00312789	0.0047784	0.00354901	0.00447879	-0.00643766
R^2	0.740616	0.830139	0.966973	0.965577	0.982902	0.98993	0.989977
$\|\Delta E\|$	1.90773	1.74792	0.437711	0.437403	0.270197	0.193143	0.218258
ΔE	-1.24425	-1.17892	0.431215	0.430953	0.19215	-0.0370765	0.107736
E_{comp}/E_{exp}	0.674487	0.692328	1.10087	1.10082	1.0424	0.989359	1.02459

Table S21: f_{comp} vs Cf_{exp} PCM/p-gauge/Improved Fit/

	CIS	HF	EOM-CCSD	LR	TDA-B3LYP	B3LYP
MAE	0.0393294	0.0943804	0.0227623	0.022429	0.0159173	0.0227094
Slope	1.09235	1.07655	1.04687	1.05105	1.06318	0.987122
Intercept	-0.0248797	-0.0425177	-0.0174513	-0.0147843	-0.0106311	0.0080483
R^2	0.868206	0.834202	0.979339	0.985016	0.936753	0.991204
$\|\Delta E\|$	1.10038	1.74637	0.455857	0.447048	0.59334	0.186238
ΔE	0.873365	-1.18046	0.449053	0.440126	0.545645	-0.0321868
E_{comp}/E_{exp}	1.20793	0.692021	1.10584	1.10262	1.1336	0.990308

Table S22: f_{comp} vs Cf_{exp} PCM/xp-gauge/Improved Fit/

	CIS	HF	EOM-CCSD	LR	TDA-B3LYP	B3LYP
MAE	0.0693814	0.0982138	0.0236273	0.0277834	0.0256232	0.0235013
Slope	1.10292	1.07817	1.03268	1.01583	1.07033	0.99133
Intercept	-0.0437703	-0.0445097	-0.0160856	-0.00845828	-0.0246796	0.00550149
R^2	0.866093	0.83238	0.986603	0.974905	0.975818	0.990645
$\|\Delta E\|$	1.63403	1.74714	0.452388	0.462303	0.450387	0.192784
ΔE	-0.38421	-1.17969	0.445833	0.455653	0.383244	-0.037733
E_{comp}/E_{exp}	0.887027	0.692174	1.10368	1.10631	1.09085	0.989222

S2.1.5 Wave function methods vs. Cf_{exp} calculated from $C_0 = 1.4$

Table S23: f_{comp} vs Cf_{exp} PCM/x-gauge/Improved Fit/

	CIS	HF	EOM-CCSD	LR	TDA-B3LYP	B3LYP	GAS
MAE	0.12111	0.102217	0.0324788	0.0298228	0.0337122	0.0247112	0.0195129
Slope	1.10091	1.0795	1.00915	1.00458	0.994951	0.99307	1.01317
Intercept	-0.0727493	-0.0464262	-0.00605771	-0.00282775	0.00388526	0.00447879	-0.00707837
R^2	0.849754	0.830139	0.983096	0.982261	0.982868	0.98993	0.990116
$\|\Delta E\|$	1.90587	1.74792	0.478586	0.477338	0.278965	0.193143	0.218427
ΔE	-1.0574	-1.17892	0.47209	0.470888	0.200918	-0.0370765	0.107905
E_{comp}/E_{exp}	0.725945	0.692328	1.10959	1.10934	1.0445	0.989359	1.02462

Table S24: f_{comp} vs Cf_{exp} PCM/p-gauge/Improved Fit/

	CIS	HF	EOM-CCSD	LR	TDA-B3LYP	B3LYP
MAE	0.0393294	0.0943804	0.0227623	0.0247049	0.0159173	0.0227094
Slope	1.09235	1.07655	1.04687	1.02026	1.06318	0.987122
Intercept	-0.0248797	-0.0425177	-0.0174513	-0.0153712	-0.0106311	0.0080483
R^2	0.868206	0.834202	0.979339	0.98117	0.936753	0.991204
$\|\Delta E\|$	1.10038	1.74637	0.455857	0.438441	0.59334	0.186238
ΔE	0.873365	-1.18046	0.449053	0.431519	0.545645	-0.0321868
E_{comp}/E_{exp}	1.20793	0.692021	1.10584	1.10104	1.1336	0.990308

Table S25: f_{comp} vs Cf_{exp} PCM/xp-gauge/Improved Fit/

	CIS	HF	EOM-CCSD	LR	TDA-B3LYP	B3LYP
MAE	0.0693814	0.0982138	0.0236273	0.0252737	0.0256232	0.0235013
Slope	1.10292	1.07817	1.03268	1.02052	1.07033	0.99133
Intercept	-0.0437703	-0.0445097	-0.0160856	-0.0105958	-0.0246796	0.00550149
R^2	0.866093	0.83238	0.986603	0.984903	0.975818	0.990645
$\|\Delta E\|$	1.63403	1.74714	0.452388	0.472218	0.450387	0.192784
ΔE	-0.38421	-1.17969	0.445833	0.465568	0.383244	-0.037733
E_{comp}/E_{exp}	0.887027	0.692174	1.10368	1.10835	1.09085	0.989222

S2.1.6 Wave function methods vs. Cf_{exp} calculated from $C_0 = 0.7$

Table S26: f_{comp} vs Cf_{exp} PCM/x-gauge/Improved Fit/

	CIS	HF	EOM-CCSD	LR	TDA-B3LYP	B3LYP	GAS
MAE	0.0571588	0.102217	0.0423761	0.039258	0.109186	0.0247112	0.0196204
Slope	1.09977	1.0795	1.06436	1.06246	1.01415	0.99307	1.01201
Intercept	-0.0120947	-0.0464262	-0.00816144	-0.00742497	-0.00412333	0.00447879	-0.00643766
R^2	0.392517	0.830139	0.611693	0.615245	0.430735	0.98993	0.989977
$\|\Delta E\|$	2.22882	1.74792	1.21979	1.22021	0.983201	0.193143	0.218258
ΔE	-1.81175	-1.17892	-0.553079	-0.55266	-0.332365	-0.0370765	0.107736
E_{comp}/E_{exp}	0.528872	0.692328	0.872744	0.872869	0.915579	0.989359	1.02459

Table S27: f_{comp} vs Cf_{exp} PCM/p-gauge/Improved Fit/

	CIS	HF	EOM-CCSD	LR	TDA-B3LYP	B3LYP
MAE	0.0391587	0.0943804	0.0216088	0.0229879	0.0159173	0.0227094
Slope	1.09225	1.07655	1.05893	1.03336	1.06318	0.987122
Intercept	-0.0246561	-0.0425177	-0.0166677	-0.0144619	-0.0106311	0.0080483
R^2	0.868145	0.834202	0.982323	0.984985	0.936753	0.991204
$\|\Delta E\|$	1.10275	1.74637	0.46873	0.449282	0.59334	0.186238
ΔE	0.875736	-1.18046	0.461925	0.44236	0.545645	-0.0321868
E_{comp}/E_{exp}	1.20878	0.692021	1.10843	1.10305	1.1336	0.990308

Table S28: f_{comp} vs Cf_{exp} PCM/xp-gauge/Improved Fit/

	CIS	HF	EOM-CCSD	LR	TDA-B3LYP	B3LYP
MAE	0.0712785	0.0982138	0.0243459	0.0277834	0.0255321	0.0235013
Slope	1.07691	1.07817	1.02226	1.01583	1.06845	0.99133
Intercept	-0.0306914	-0.0445097	-0.006691	-0.00845828	-0.0239228	0.00550149
R^2	0.834009	0.83238	0.979289	0.974905	0.974957	0.990645
$\|\Delta E\|$	1.63118	1.74714	0.444079	0.462303	0.44872	0.192784
ΔE	-0.387069	-1.17969	0.437524	0.455653	0.384321	-0.037733
E_{comp}/E_{exp}	0.886471	0.692174	1.10193	1.10631	1.0912	0.989222

S2.2 Cavity Field Corrections

S2.2.1 $f_{exp}/n(\tilde{\nu})$

Avg. $f_{exp}/n(\tilde{\nu})$ =0.155384

Table S29: f_{comp} vs $C_{theoretical}f_{exp}$ /x-gauge/Exact Band Limits/

	LR	B3LYP	GAS
MAE	0.10583	0.135498	0.0851869
Slope	0.992072	1.7812	1.50967
Intercept	0.0475675	0.0141126	0.00339388
R^2	0.602986	0.984348	0.968671
$\|\Delta E\|$	0.987842	0.194237	0.226343
ΔE	-0.36936	-0.0383761	0.0994301
E_{comp}/E_{exp}	0.930367	0.989187	1.02293

Table S30: f_{comp} vs $C_{theoretical}f_{exp}$ /p-gauge/Exact Band Limits/

	LR	B3LYP
MAE	0.0654495	0.126644
Slope	0.7571	1.71978
Intercept	0.0231671	0.0148014
R^2	0.597079	0.986678
$\|\Delta E\|$	0.990235	0.193666
ΔE	-0.367438	-0.0396048
E_{comp}/E_{exp}	0.930782	0.988917

Table S31: f_{comp} vs $C_{theoretical}f_{exp}$ /xp-gauge/Exact Band Limits/

	LR	B3LYP
MAE	0.0816036	0.130916
Slope	0.869918	1.75007
Intercept	0.034074	0.0143661
R^2	0.602381	0.985688
$\|\Delta E\|$	0.989014	0.193965
ΔE	-0.368387	-0.0390178
E_{comp}/E_{exp}	0.930576	0.989044

Improving fit:

Table S32: f_{comp} vs $C_{theoretical}f_{exp}$ /x-gauge/Improved Fit/

	LR	B3LYP	GAS
MAE	0.0829582	0.0951031	0.0532096
Slope	0.695335	1.28434	1.15354
Intercept	0.0644272	0.0293796	0.0268892
R^2	0.548505	0.828696	0.925252
$\|\Delta E\|$	0.68208	0.417638	0.258943
ΔE	0.185225	-0.0938946	0.129914
E_{comp}/E_{exp}	1.04009	0.977769	1.03032

Table S33: f_{comp} vs $C_{theoretical} f_{exp}$ /p-gauge/Improved Fit/

	LR	B3LYP
MAE	0.0285255	0.0938898
Slope	1.10664	1.25275
Intercept	0.000108919	0.0329729
R^2	0.959659	0.839007
$\|\Delta E\|$	0.452378	0.352537
ΔE	0.444816	-0.159577
E_{comp}/E_{exp}	1.10445	0.96231

Table S34: f_{comp} vs $C_{theoretical} f_{exp}$ /xp-gauge/Improved Fit/

	LR	B3LYP
MAE	0.0540639	0.0913069
Slope	1.28381	1.25878
Intercept	-0.0029329	0.0295628
R^2	0.951245	0.833461
$\|\Delta E\|$	0.547341	0.413593
ΔE	0.276061	-0.0979488
E_{comp}/E_{exp}	1.0623	0.976813

S2.2.2 f_{exp}/n_D

Avg. f_{exp}/n_D =0.160009

Table S35: f_{comp} vs $C_{theoretical} f_{exp}$ /x-gauge/Exact Band Limits/

	LR	B3LYP	GAS
MAE	0.105519	0.130874	0.0809078
Slope	0.934833	1.70063	1.44185
Intercept	0.0521381	0.0187656	0.00726328
R^2	0.585795	0.981756	0.966743
$\|\Delta E\|$	0.987842	0.194237	0.226343
ΔE	-0.36936	-0.0383761	0.0994301
E_{comp}/E_{exp}	0.930367	0.989187	1.02293

Table S36: f_{comp} vs $C_{theoretical} f_{exp}$ /p-gauge/Exact Band Limits/

	LR	B3LYP
MAE	0.0664769	0.122019
Slope	0.71346	1.6425
Intercept	0.0266483	0.0192141
R^2	0.580126	0.984678
$\|\Delta E\|$	0.990235	0.193666
ΔE	-0.367438	-0.0396048
E_{comp}/E_{exp}	0.930782	0.988917

Table S37: f_{comp} vs $C_{theoretical} f_{exp}$ /xp-gauge/Exact Band Limits/

	LR	B3LYP
MAE	0.0817806	0.126291
Slope	0.819733	1.67117
Intercept	0.0380808	0.0188978
R^2	0.585217	0.983387
$\|\Delta E\|$	0.989014	0.193965
ΔE	-0.368387	-0.0390178
E_{comp}/E_{exp}	0.930576	0.989044

Improved fit:

Table S38: f_{comp} vs $C_{theoretical} f_{exp}$ /x-gauge/Improved Fit/

	LR	B3LYP	GAS
MAE	0.0740157	0.091431	0.0516686
Slope	1.00135	1.22149	1.11333
Intercept	0.0242087	0.0337696	0.0306164
R^2	0.754047	0.81993	0.924193
$\|\Delta E\|$	0.695218	0.414391	0.219813
ΔE	0.198363	-0.0971415	0.0911743
E_{comp}/E_{exp}	1.04234	0.977005	1.02119

Table S39: f_{comp} vs $C_{theoretical} f_{exp}$ /p-gauge/Improved Fit/

	LR	B3LYP
MAE	0.0276994	0.0906494
Slope	1.06319	1.20006
Intercept	0.00250004	0.0518851
R^2	0.956339	0.871454
$\|\Delta E\|$	0.453227	0.226365
ΔE	0.445664	-0.0276058
E_{comp}/E_{exp}	1.10464	0.992087

Table S40: f_{comp} vs $C_{theoretical} f_{exp}$ /xp-gauge/Improved Fit/

	LR	B3LYP
MAE	0.0504006	0.094495
Slope	1.22425	1.21692
Intercept	0.000885204	0.0369244
R^2	0.945514	0.825745
$\|\Delta E\|$	0.547731	0.352883
ΔE	0.27645	-0.15927
E_{comp}/E_{exp}	1.06238	0.962384

S2.2.3 $C_{Chako}(n) f_{exp}$

Avg. $C_{Chako}(n) f_{exp} = 0.174654$

Table S41: f_{comp} vs $C_{theoretical} f_{exp}$ /x-gauge/Exact Band Limits/

	LR	B3LYP	GAS
MAE	0.0987272	0.116443	0.0682787
Slope	0.881656	1.58386	1.34243
Intercept	0.047735	0.0142547	0.00351208
R^2	0.602251	0.984276	0.968619
$\|\Delta E\|$	0.987842	0.194237	0.226343
ΔE	-0.36936	-0.0383761	0.0994301
E_{comp}/E_{exp}	0.930367	0.989187	1.02293

Table S42: f_{comp} vs $C_{theoretical} f_{exp}$ /p-gauge/Exact Band Limits/

	LR	B3LYP
MAE	0.0651866	0.10757
Slope	0.672861	1.52927
Intercept	0.0232906	0.0149354
R^2	0.596395	0.98663
$\|\Delta E\|$	0.990235	0.193666
ΔE	-0.367438	-0.0396048
E_{comp}/E_{exp}	0.930782	0.988917

Table S43: f_{comp} vs $C_{theoretical}f_{exp}$ /xp-gauge/Exact Band Limits/

	LR	B3LYP
MAE	0.0777328	0.111809
Slope	0.77311	1.55619
Intercept	0.0342188	0.0145041
R^2	0.601665	0.985629
$\|\Delta E\|$	0.989014	0.193965
ΔE	-0.368387	-0.0390178
E_{comp}/E_{exp}	0.930576	0.989044

Improved fit:

Table S44: f_{comp} vs $C_{theoretical}f_{exp}$ /x-gauge/Improved Fit/

	LR	B3LYP	GAS
MAE	0.0637513	0.0887271	0.0451403
Slope	1.13812	1.1567	1.04236
Intercept	0.0201191	0.0578291	0.0257065
R^2	0.89176	0.884405	0.928242
$\|\Delta E\|$	0.635002	0.215897	0.22774
ΔE	0.138146	-0.0260182	0.0824733
E_{comp}/E_{exp}	1.02406	0.992451	1.0191

Table S45: f_{comp} vs $C_{theoretical}f_{exp}$ /p-gauge/Improved Fit/

	LR	B3LYP
MAE	0.0210626	0.080652
Slope	1.06023	1.12289
Intercept	-0.00687339	0.0512117
R^2	0.977345	0.890757
$\|\Delta E\|$	0.449713	0.221326
ΔE	0.442927	-0.0322065
E_{comp}/E_{exp}	1.1032	0.990887

Table S46: f_{comp} vs $C_{theoretical}f_{exp}$ /xp-gauge/Improved Fit/

	LR	B3LYP
MAE	0.0401261	0.0845596
Slope	1.14017	1.14482
Intercept	0.00736951	0.0517347
R^2	0.960256	0.884979
$\|\Delta E\|$	0.449314	0.221491
ΔE	0.442664	-0.0319365
E_{comp}/E_{exp}	1.10399	0.990945

S2.2.4 $C_{Chako}(n_D)f_{exp}$

Avg. $C_{Chako}(n_D)f_{exp} = 0.179524$

Table S47: f_{comp} vs $C_{theoretical}f_{exp}$ /x-gauge/Exact Band Limits/

	LR	B3LYP	GAS
MAE	0.0988565	0.111752	0.0637639
Slope	0.834222	1.51731	1.28639
Intercept	0.0519573	0.0184901	0.00703534
R^2	0.586066	0.981824	0.966763
$\|\Delta E\|$	0.987842	0.194237	0.226343
ΔE	-0.36936	-0.0383761	0.0994301
E_{comp}/E_{exp}	0.930367	0.989187	1.02293

Table S48: f_{comp} vs $C_{theoretical} f_{exp}$ /p-gauge/Exact Band Limits/

	LR	B3LYP
MAE	0.0673491	0.1029
Slope	0.636707	1.46542
Intercept	0.0265044	0.0189509
R^2	0.580454	0.984726
$\|\Delta E\|$	0.990235	0.193666
ΔE	-0.367438	-0.0396048
E_{comp}/E_{exp}	0.930782	0.988917

Table S49: f_{comp} vs $C_{theoretical} f_{exp}$ /xp-gauge/Exact Band Limits/

	LR	B3LYP
MAE	0.0785955	0.107118
Slope	0.731528	1.49101
Intercept	0.037919	0.0186285
R^2	0.585515	0.983445
$\|\Delta E\|$	0.989014	0.193965
ΔE	-0.368387	-0.0390178
E_{comp}/E_{exp}	0.930576	0.989044

Improved fit:

Table S50: f_{comp} vs $C_{theoretical} f_{exp}$ /x-gauge/Improved Fit/

	LR	B3LYP	GAS
MAE	0.0673213	0.0875011	0.0445578
Slope	1.26885	1.10308	0.9957
Intercept	-0.000706723	0.0618229	0.0290085
R^2	0.939478	0.874232	0.920635
$\|\Delta E\|$	0.629153	0.215897	0.227733
ΔE	0.132298	-0.0260182	0.0824656
E_{comp}/E_{exp}	1.02305	0.992451	1.0191

Table S51: f_{comp} vs $C_{theoretical} f_{exp}$ /p-gauge/Improved Fit/

	LR	B3LYP
MAE	0.0216526	0.0803926
Slope	1.04699	1.23123
Intercept	-0.00806552	0.0332852
R^2	0.979388	0.945233
$\|\Delta E\|$	0.461219	0.21618
ΔE	0.454297	-0.02706
E_{comp}/E_{exp}	1.1052	0.991783

Table S52: f_{comp} vs $C_{theoretical} f_{exp}$ /xp-gauge/Improved Fit/

	LR	B3LYP
MAE	0.0366717	0.0833725
Slope	1.09074	1.08162
Intercept	0.0106946	0.0612083
R^2	0.955212	0.880568
$\|\Delta E\|$	0.449372	0.215814
ΔE	0.442722	-0.0262593
E_{comp}/E_{exp}	1.10401	0.992397

S2.2.5 $C_{AbeO}(n) f_{exp}$

Avg. $C_{AbeO}(n) f_{exp} = 0.177222$

Table S53: f_{comp} vs $C_{theoretical} f_{exp}$ /x-gauge/Exact Band Limits/

	LR	B3LYP	GAS
MAE	0.0983849	0.113966	0.0659193
Slope	0.862934	1.55755	1.32015
Intercept	0.0487893	0.0148519	0.00401278
R^2	0.596119	0.983473	0.967874
$\|\Delta E\|$	0.987842	0.194237	0.226343
ΔE	-0.36936	-0.0383761	0.0994301
E_{comp}/E_{exp}	0.930367	0.989187	1.02293

Table S54: f_{comp} vs $C_{theoretical} f_{exp}$ /p-gauge/Exact Band Limits/

	LR	B3LYP
MAE	0.0662186	0.105164
Slope	0.658734	1.50398
Intercept	0.0240665	0.0154909
R^2	0.590612	0.985981
$\|\Delta E\|$	0.990235	0.193666
ΔE	-0.367438	-0.0396048
E_{comp}/E_{exp}	0.930782	0.988917

Table S55: f_{comp} vs $C_{theoretical} f_{exp}$ /xp-gauge/Exact Band Limits/

	LR	B3LYP
MAE	0.077775	0.109332
Slope	0.756774	1.5304
Intercept	0.035129	0.0150803
R^2	0.595666	0.984901
$\|\Delta E\|$	0.989014	0.193965
ΔE	-0.368387	-0.0390178
E_{comp}/E_{exp}	0.930576	0.989044

Improved fit:

Table S56: f_{comp} vs $C_{theoretical} f_{exp}$ /x-gauge/Improved Fit/

	LR	B3LYP	GAS
MAE	0.0629124	0.0876104	0.0442173
Slope	1.11718	1.13603	1.02412
Intercept	0.020908	0.0585226	0.0262633
R^2	0.887802	0.881429	0.925826
$\|\Delta E\|$	0.635002	0.215897	0.227733
ΔE	0.138146	-0.0260182	0.0824656
E_{comp}/E_{exp}	1.02406	0.992451	1.0191

Table S57: f_{comp} vs $C_{theoretical} f_{exp}$ /p-gauge/Improved Fit/

	LR	B3LYP
MAE	0.0219842	0.079635
Slope	1.04311	1.10292
Intercept	-0.0063704	0.0518674
R^2	0.976372	0.887917
$\|\Delta E\|$	0.449479	0.221326
ΔE	0.442557	-0.0322065
E_{comp}/E_{exp}	1.10306	0.990887

Table S58: f_{comp} vs $C_{theoretical} f_{exp}$ /xp-gauge/Improved Fit/

	LR	B3LYP
MAE	0.0381617	0.0834829
Slope	1.12071	1.1138
Intercept	0.0078943	0.0579959
R^2	0.9586	0.887603
$\|\Delta E\|$	0.449372	0.215814
ΔE	0.442722	-0.0262593
E_{comp}/E_{exp}	1.10401	0.992397

S2.2.6 $C_{AbeO}(n_D) f_{exp}$

Avg. $C_{AbeO}(n_D) f_{exp} = 0.180027$

Table S59: f_{comp} vs $C_{theoretical} f_{exp}$ /x-gauge/Exact Band Limits/

	LR	B3LYP	GAS
MAE	0.0985194	0.111266	0.0633241
Slope	0.835813	1.5192	1.28788
Intercept	0.0512509	0.0173845	0.00611886
R^2	0.586951	0.982019	0.966781
$\|\Delta E\|$	0.987842	0.194237	0.226343
ΔE	-0.36936	-0.0383761	0.0994301
E_{comp}/E_{exp}	0.930367	0.989187	1.02293

Table S60: f_{comp} vs $C_{theoretical} f_{exp}$ /p-gauge/Exact Band Limits/

	LR	B3LYP
MAE	0.0676287	0.102475
Slope	0.638047	1.4672
Intercept	0.0259426	0.0178933
R^2	0.581559	0.984845
$\|\Delta E\|$	0.990235	0.193666
ΔE	-0.367438	-0.0396048
E_{comp}/E_{exp}	0.930782	0.988917

Table S61: f_{comp} vs $C_{theoretical} f_{exp}$ /xp-gauge/Exact Band Limits/

	LR	B3LYP
MAE	0.078291	0.106643
Slope	0.73299	1.49284
Intercept	0.0372875	0.0175472
R^2	0.586507	0.983603
$\|\Delta E\|$	0.989014	0.193965
ΔE	-0.368387	-0.0390178
E_{comp}/E_{exp}	0.930576	0.989044

Improved fit:

Table S62: f_{comp} vs $C_{theoretical} f_{exp}$ /x-gauge/Improved Fit/

	LR	B3LYP	GAS
MAE	0.06695	0.0869285	0.0440813
Slope	1.27022	1.10516	0.997263
Intercept	-0.00159202	0.0608922	0.028226
R^2	0.939342	0.875522	0.921403
$\|\Delta E\|$	0.629153	0.215897	0.227733
ΔE	0.132298	-0.0260182	0.0824656
E_{comp}/E_{exp}	1.02305	0.992451	1.0191

Table S63: f_{comp} vs $C_{theoretical} f_{exp}$ /p-gauge/Improved Fit/

	LR	B3LYP
MAE	0.0225219	0.0800287
Slope	1.04914	1.23313
Intercept	-0.00946308	0.0323231
R^2	0.977509	0.945974
$\|\Delta E\|$	0.461178	0.21618
ΔE	0.454256	-0.02706
E_{comp}/E_{exp}	1.10515	0.991783

Table S64: f_{comp} vs $C_{theoretical} f_{exp}$ /xp-gauge/Improved Fit/

	LR	B3LYP
MAE	0.0362332	0.0828116
Slope	1.09223	1.08364
Intercept	0.0098765	0.0603007
R^2	0.955629	0.881822
$\|\Delta E\|$	0.449372	0.215814
ΔE	0.442722	-0.0262593
E_{comp}/E_{exp}	1.10401	0.992397

S2.2.7 f_{exp}

Avg. f_{exp} =0.215319

Table S65: f_{comp} vs $C_{theoretical} f_{exp}$ /x-gauge/Exact Band Limits/

	LR	B3LYP	GAS
MAE	0.09107	0.07839	0.0367631
Slope	0.703667	1.27771	1.08301
Intercept	0.0502075	0.0157681	0.00478047
R^2	0.588269	0.982224	0.966718
$\|\Delta E\|$	0.987842	0.194237	0.226343
ΔE	-0.36936	-0.0383761	0.0994301
E_{comp}/E_{exp}	0.930367	0.989187	1.02293

Table S66: f_{comp} vs $C_{theoretical} f_{exp}$ /p-gauge/Exact Band Limits/

	LR	B3LYP
MAE	0.0814786	0.0696729
Slope	0.537328	1.2339
Intercept	0.0251118	0.016348
R^2	0.583212	0.984933
$\|\Delta E\|$	0.990235	0.193666
ΔE	-0.367438	-0.0396048
E_{comp}/E_{exp}	0.930782	0.988917

Table S67: f_{comp} vs $C_{theoretical} f_{exp}$ /xp-gauge/Exact Band Limits/

	LR	B3LYP
MAE	0.0789586	0.0739043
Slope	0.617186	1.2555
Intercept	0.0363542	0.0159667
R^2	0.587986	0.983751
$\|\Delta E\|$	0.989014	0.193965
ΔE	-0.368387	-0.0390178
E_{comp}/E_{exp}	0.930576	0.989044

Improving fit:

Table S68: f_{comp} vs $C_{theoretical} f_{exp}$ /x-gauge/Improved Fit/

	LR	B3LYP	GAS
MAE	0.04099	0.0603243	0.0219836
Slope	1.0567	1.07543	1.03257
Intercept	0.0184981	0.0371228	0.00265326
R^2	0.952296	0.94819	0.984277
$\|\Delta E\|$	0.447399	0.206482	0.22106
ΔE	0.440949	-0.0205926	0.0989957
E_{comp}/E_{exp}	1.10365	0.993425	1.02285

Table S69: f_{comp} vs $C_{theoretical} f_{exp}$ /p-gauge/Improved Fit/

	LR	B3LYP
MAE	0.0282986	0.0518471
Slope	0.935483	1.06175
Intercept	-0.00751536	0.0341303
R^2	0.978309	0.964913
$\|\Delta E\|$	0.508896	0.210964
ΔE	0.501974	-0.0212969
E_{comp}/E_{exp}	1.11735	0.99323

Table S70: f_{comp} vs $C_{theoretical} f_{exp}$ /x-gauge/Improved Fit/

	LR	B3LYP
MAE	0.0259557	0.0562329
Slope	1.0371	1.07764
Intercept	-0.00791798	0.0348819
R^2	0.977422	0.960506
$\|\Delta E\|$	0.45366	0.210943
ΔE	0.44701	-0.0213947
E_{comp}/E_{exp}	1.10431	0.993209

S2.2.8 $C_{AbeL}(n_D) f_{exp}$

Avg. $C_{AbeL}(n_D) f_{exp} = 0.241583$

Table S71: f_{comp} vs $C_{theoretical} f_{exp}$ /x-gauge/Exact Band Limits/

	LR	B3LYP	GAS
MAE	0.0906924	0.0549551	0.0328518
Slope	0.627909	1.13992	0.966195
Intercept	0.0500277	0.0154965	0.00455597
R^2	0.588521	0.982254	0.9667
$\|\Delta E\|$	0.987842	0.194237	0.226343
ΔE	-0.36936	-0.0383761	0.0994301
E_{comp}/E_{exp}	0.930367	0.989187	1.02293

Table S72: f_{comp} vs $C_{theoretical} f_{exp}$ /p-gauge/Exact Band Limits/

	LR	B3LYP
MAE	0.100879	0.0461986
Slope	0.479504	1.10082
Intercept	0.0249684	0.0160885
R^2	0.583524	0.984942
$\|\Delta E\|$	0.990235	0.193666
ΔE	-0.367438	-0.0396048
E_{comp}/E_{exp}	0.930782	0.988917

Table S73: f_{comp} vs $C_{theoretical} f_{exp}$ /xp-gauge/Exact Band Limits/

	LR	B3LYP
MAE	0.0862442	0.0500606
Slope	0.550752	1.12011
Intercept	0.0361932	0.0157012
R^2	0.588267	0.983771
$\|\Delta E\|$	0.989014	0.193965
ΔE	-0.368387	-0.0390178
E_{comp}/E_{exp}	0.930576	0.989044

Improved fit:

Table S74: f_{comp} vs $C_{theoretical} f_{exp}$ /x-gauge/Improved Fit/

	LR	B3LYP	GAS
MAE	0.0299586	0.0458868	0.0195663
Slope	1.03768	1.1142	1.01276
Intercept	0.00552653	0.0116844	-0.0100183
R^2	0.974611	0.983777	0.990497
$\|\Delta E\|$	0.466396	0.204124	0.220714
ΔE	0.459946	-0.0147291	0.0784006
E_{comp}/E_{exp}	1.10721	0.994526	1.01686

Table S75: f_{comp} vs $C_{theoretical} f_{exp}$ /p-gauge/Improved Fit/

	LR	B3LYP
MAE	0.0423165	0.0383088
Slope	0.863201	1.0756
Intercept	-0.00226625	0.0125736
R^2	0.960844	0.986875
$\|\Delta E\|$	0.565087	0.205593
ΔE	0.558165	-0.0156515
E_{comp}/E_{exp}	1.13056	0.994328

Table S76: f_{comp} vs $C_{theoretical} f_{exp}$ /xp-gauge/Improved Fit/

	LR	B3LYP
MAE	0.0276748	0.0416713
Slope	1.0111	1.0945
Intercept	-0.0151611	0.0117655
R^2	0.982517	0.985214
$\|\Delta E\|$	0.467673	0.204304
ΔE	0.461024	-0.0145756
E_{comp}/E_{exp}	1.10717	0.994556

S2.2.9 $C_{AbeL}(n) f_{exp}$

Avg. $C_{AbeL}(n) f_{exp} = 0.292172$

Table S77: f_{comp} vs $C_{theoretical} f_{exp}$ /x-gauge/Exact Band Limits/

	LR	B3LYP	GAS
MAE	0.107647	0.0355799	0.0654132
Slope	0.50791	0.934391	0.792061
Intercept	0.0533226	0.0178795	0.00655394
R^2	0.570933	0.978525	0.963212
$\|\Delta E\|$	0.987842	0.194237	0.226343
ΔE	-0.36936	-0.0383761	0.0994301
E_{comp}/E_{exp}	0.930367	0.989187	1.02293

Table S78: f_{comp} vs $C_{theoretical} f_{exp}$ /p-gauge/Exact Band Limits/

	LR	B3LYP
MAE	0.151364	0.034201
Slope	0.388118	0.902547
Intercept	0.0274112	0.0183292
R^2	0.566817	0.981654
$\|\Delta E\|$	0.990235	0.193666
ΔE	-0.367438	-0.0396048
E_{comp}/E_{exp}	0.930782	0.988917

Table S79: f_{comp} vs $C_{theoretical} f_{exp}$ /xp-gauge/Exact Band Limits/

	LR	B3LYP
MAE	0.123886	0.034201
Slope	0.445623	0.918251
Intercept	0.039047	0.0180124
R^2	0.571004	0.980258
$\|\Delta E\|$	0.989014	0.193965
ΔE	-0.368387	-0.0390178
E_{comp}/E_{exp}	0.930576	0.989044

Improved fit:

Table S80: f_{comp} vs $C_{theoretical} f_{exp}$ /x-gauge/Improved Fit/

	LR	B3LYP	GAS
MAE	0.0384927	0.0276522	0.0376204
Slope	0.986858	0.973624	0.867072
Intercept	-0.0123211	0.00954815	0.00578697
R^2	0.978483	0.988465	0.981936
$\|\Delta E\|$	0.4662	0.175652	0.259482
ΔE	0.459749	-0.0190321	0.156373
E_{comp}/E_{exp}	1.10689	0.992986	1.0349

Table S81: f_{comp} vs $C_{theoretical} f_{exp}$ /p-gauge/Improved Fit/

	LR	B3LYP
MAE	0.0719332	0.0271178
Slope	0.728955	0.952067
Intercept	0.0128081	0.0143036
R^2	0.923883	0.986339
$\|\Delta E\|$	0.648802	0.186727
ΔE	0.64188	-0.00833536
E_{comp}/E_{exp}	1.14954	0.995361

Table S82: f_{comp} vs $C_{theoretical} f_{exp}$ /xp-gauge/Improved Fit/

	LR	B3LYP
MAE	0.0515868	0.0255904
Slope	0.861507	0.957689
Intercept	-0.00231722	0.0108212
R^2	0.963142	0.989576
$\|\Delta E\|$	0.534399	0.176579
ΔE	0.527749	-0.0181332
E_{comp}/E_{exp}	1.12317	0.993147

S2.2.10 $n_D f_{exp}$

Avg. $n_D f_{exp} = 0.289814$

Table S83: f_{comp} vs $C_{theoretical} f_{exp}$ /x-gauge/Exact Band Limits/

	LR	B3LYP	GAS
MAE	0.103771	0.0328761	0.0630505
Slope	0.529434	0.959522	0.813098
Intercept	0.0482827	0.0128003	0.00232536
R^2	0.590529	0.982269	0.966267
$\|\Delta E\|$	0.987842	0.194237	0.226343
ΔE	-0.36936	-0.0383761	0.0994301
E_{comp}/E_{exp}	0.930367	0.989187	1.02293

Table S84: f_{comp} vs $C_{theoretical} f_{exp}$ /p-gauge/Exact Band Limits/

	LR	B3LYP
MAE	0.149005	0.0320314
Slope	0.404507	0.926515
Intercept	0.0235769	0.0135118
R^2	0.586103	0.98476
$\|\Delta E\|$	0.990235	0.193666
ΔE	-0.367438	-0.0396048
E_{comp}/E_{exp}	0.930782	0.988917

Table S85: f_{comp} vs $C_{theoretical} f_{exp}$ /xp-gauge/Exact Band Limits/

	LR	B3LYP
MAE	0.121518	0.0318628
Slope	0.464486	0.942794
Intercept	0.0346312	0.0130653
R^2	0.59055	0.983689
$\|\Delta E\|$	0.989014	0.193965
ΔE	-0.368387	-0.0390178
E_{comp}/E_{exp}	0.930576	0.989044

Improved fit:

Table S86: f_{comp} vs $C_{theoretical} f_{exp}$ /x-gauge/Improved Fit/

	LR	B3LYP	GAS
MAE	0.0359499	0.0255297	0.0333985
Slope	0.973407	0.992991	0.89472
Intercept	-0.00608661	0.00165479	0.00153599
R^2	0.981332	0.989353	0.983971
$\|\Delta E\|$	0.479814	0.193422	0.262045
ΔE	0.473363	-0.0368017	0.134743
E_{comp}/E_{exp}	1.10993	0.989413	1.03003

Table S87: f_{comp} vs $C_{theoretical} f_{exp}$ /p-gauge/Improved Fit/

	LR	B3LYP
MAE	0.0687823	0.0255563
Slope	0.754542	0.974394
Intercept	0.0113061	0.00921313
R^2	0.920767	0.987874
$\|\Delta E\|$	0.675407	0.185337
ΔE	0.668485	-0.00970797
E_{comp}/E_{exp}	1.15496	0.995107

Table S88: f_{comp} vs $C_{theoretical} f_{exp}$ /xp-gauge/Improved Fit/

	LR	B3LYP
MAE	0.0503223	0.0250925
Slope	0.883941	0.975275
Intercept	-0.00643829	0.00608065
R^2	0.966607	0.990551
$\|\Delta E\|$	0.546543	0.175441
ΔE	0.539893	-0.0197626
E_{comp}/E_{exp}	1.12627	0.992834

S2.2.11 $n(\tilde{\nu}) f_{exp}$

Avg. $n(\tilde{\nu}) f_{exp} = 0.298506$

Table S89: f_{comp} vs $C_{theoretical} f_{exp}$ /x-gauge/Exact Band Limits/

	LR	B3LYP	GAS
MAE	0.110339	0.035152	0.0716377
Slope	0.498576	0.915508	0.77605
Intercept	0.0528919	0.017598	0.0063167
R^2	0.573433	0.979147	0.963813
$\|\Delta E\|$	0.987842	0.194237	0.226343
ΔE	-0.36936	-0.0383761	0.0994301
E_{comp}/E_{exp}	0.930367	0.989187	1.02293

Table S90: f_{comp} vs $C_{theoretical} f_{exp}$ /p-gauge/Exact Band Limits/

	LR	B3LYP
MAE	0.157698	0.0354469
Slope	0.380948	0.884281
Intercept	0.0270932	0.018065
R^2	0.569188	0.982221
$\|\Delta E\|$	0.990235	0.193666
ΔE	-0.367438	-0.0396048
E_{comp}/E_{exp}	0.930782	0.988917

Table S91: f_{comp} vs $C_{theoretical} f_{exp}$ /xp-gauge/Exact Band Limits/

	LR	B3LYP
MAE	0.129864	0.0352389
Slope	0.437415	0.899681
Intercept	0.0386747	0.0177396
R^2	0.573456	0.980853
$\|\Delta E\|$	0.989014	0.193965
ΔE	-0.368387	-0.0390178
E_{comp}/E_{exp}	0.930576	0.989044

Improved fit:

Table S92: f_{comp} vs $C_{theoretical} f_{exp}$ /x-gauge/Improved Fit/

	LR	B3LYP	GAS
MAE	0.0398846	0.0272994	0.0394785
Slope	0.929664	0.957696	0.865592
Intercept	-0.00148763	0.00988454	0.00571613
R^2	0.979275	0.988671	0.975999
$\|\Delta E\|$	0.478801	0.176893	0.261766
ΔE	0.47235	-0.0174046	0.165961
E_{comp}/E_{exp}	1.10972	0.993299	1.03737

Table S93: f_{comp} vs $C_{theoretical} f_{exp}$ /p-gauge/Improved Fit/

	LR	B3LYP
MAE	0.0734251	0.0292554
Slope	0.720725	0.940066
Intercept	0.0149362	0.013913
R^2	0.915289	0.9856
$\|\Delta E\|$	0.66787	0.18724
ΔE	0.660948	-0.00882724
E_{comp}/E_{exp}	1.15356	0.995247

Table S94: f_{comp} vs $C_{theoretical} f_{exp}$ /xp-gauge/Improved Fit/

	LR	B3LYP
MAE	0.0541206	0.0270011
Slope	0.85094	0.938197
Intercept	-0.00288528	0.0106024
R^2	0.958752	0.989785
$\|\Delta E\|$	0.53152	0.176536
ΔE	0.52487	-0.0180902
E_{comp}/E_{exp}	1.12278	0.993157

S2.2.12 $C_{Schuyer}(n) f_{exp}$

Avg. $C_{Schuyer}(n) f_{exp} = 0.299294$

Table S95: f_{comp} vs $C_{theoretical} f_{exp}$ /x-gauge/Exact Band Limits/

	LR	B3LYP	GAS
MAE	0.111357	0.0357268	0.0724258
Slope	0.495838	0.912141	0.773201
Intercept	0.0533188	0.0178847	0.00655812
R^2	0.570995	0.978545	0.963234
$\|\Delta E\|$	0.987842	0.194237	0.226343
ΔE	-0.36936	-0.0383761	0.0994301
E_{comp}/E_{exp}	0.930367	0.989187	1.02293

Table S96: f_{comp} vs $C_{theoretical} f_{exp}$ /p-gauge/Exact Band Limits/

	LR	B3LYP
MAE	0.158485	0.0360744
Slope	0.378892	0.881055
Intercept	0.0274085	0.0183343
R^2	0.566877	0.981674
$\|\Delta E\|$	0.990235	0.193666
ΔE	-0.367438	-0.0396048
E_{comp}/E_{exp}	0.930782	0.988917

Table S97: f_{comp} vs $C_{theoretical} f_{exp}$ /xp-gauge/Exact Band Limits/

	LR	B3LYP
MAE	0.130644	0.0357324
Slope	0.43503	0.896385
Intercept	0.0390438	0.0180175
R^2	0.571065	0.980278
$\|\Delta E\|$	0.989014	0.193965
ΔE	-0.368387	-0.0390178
E_{comp}/E_{exp}	0.930576	0.989044

Improved fit:

Table S98: f_{comp} vs $C_{theoretical} f_{exp}$ /x-gauge/Improved Fit/

	LR	B3LYP	GAS
MAE	0.0406528	0.0274478	0.0401832
Slope	0.963694	0.954359	0.862458
Intercept	-0.0124048	0.0101292	0.00597242
R^2	0.978442	0.988446	0.975511
$\|\Delta E\|$	0.465551	0.176893	0.261766
ΔE	0.459101	-0.0174046	0.165961
E_{comp}/E_{exp}	1.10676	0.993299	1.03737

Table S99: f_{comp} vs $C_{theoretical} f_{exp}$ /p-gauge/Improved Fit/

	LR	B3LYP
MAE	0.0737219	0.0297269
Slope	0.716525	0.938038
Intercept	0.0162685	0.0148367
R^2	0.914623	0.985159
$\|\Delta E\|$	0.703938	0.188021
ΔE	0.697017	-0.0106619
E_{comp}/E_{exp}	1.16131	0.994885

Table S100: f_{comp} vs $C_{theoretical} f_{exp}$ /xp-gauge/Improved Fit/

	LR	B3LYP
MAE	0.0536445	0.0272878
Slope	0.847176	0.935607
Intercept	-0.00150013	0.0110244
R^2	0.960602	0.989437
$\|\Delta E\|$	0.532087	0.177474
ΔE	0.525437	-0.0190282
E_{comp}/E_{exp}	1.12292	0.992937

S2.2.13 $C_{Schuyer}(n_D) f_{exp}$

Avg. $C_{Schuyer}(n_D) f_{exp} = 0.346714$

Table S101: f_{comp} vs $C_{theoretical} f_{exp}$ /x-gauge/Exact Band Limits/

	LR	B3LYP	GAS
MAE	0.147408	0.0599616	0.119125
Slope	0.447301	0.809347	0.685686
Intercept	0.0466345	0.0102707	0.000235276
R^2	0.592256	0.981938	0.965507
$\|\Delta E\|$	0.987842	0.194237	0.226343
ΔE	-0.36936	-0.0383761	0.0994301
E_{comp}/E_{exp}	0.930367	0.989187	1.02293

Table S102: f_{comp} vs $C_{theoretical} f_{exp}$ /p-gauge/Exact Band Limits/

	LR	B3LYP
MAE	0.205906	0.0679872
Slope	0.34192	0.78143
Intercept	0.02226	0.0110956
R^2	0.58839	0.984236
$\|\Delta E\|$	0.990235	0.193666
ΔE	-0.367438	-0.0396048
E_{comp}/E_{exp}	0.930782	0.988917

Table S103: f_{comp} vs $C_{theoretical} f_{exp}$ /xp-gauge/Exact Band Limits/

	LR	B3LYP
MAE	0.177468	0.0635434
Slope	0.392517	0.7952
Intercept	0.0331545	0.010593
R^2	0.592545	0.983263
$\|\Delta E\|$	0.989014	0.193965
ΔE	-0.368387	-0.0390178
E_{comp}/E_{exp}	0.930576	0.989044

Improved fit:

Table S104: f_{comp} vs $C_{theoretical} f_{exp}$ /x-gauge/Improved Fit/

	LR	B3LYP	GAS
MAE	0.0635483	0.0329489	0.0692102
Slope	0.886503	0.913054	0.793577
Intercept	-0.0142832	0.00891998	0.0111876
R^2	0.963587	0.982963	0.947104
$\|\Delta E\|$	0.489258	0.217707	0.312309
ΔE	0.481695	0.0532378	0.224807
E_{comp}/E_{exp}	1.11197	1.00991	1.04878

Table S105: f_{comp} vs $C_{theoretical} f_{exp}$ /p-gauge/Improved Fit/

	LR	B3LYP
MAE	0.0992907	0.0364454
Slope	0.631655	0.867926
Intercept	0.042062	0.0163578
R^2	0.873241	0.980353
$\|\Delta E\|$	0.935097	0.219604
ΔE	0.72718	0.0602628
E_{comp}/E_{exp}	1.16015	1.01151

Table S106: f_{comp} vs $C_{theoretical} f_{exp}$ /x-gauge/Improved Fit/

	LR	B3LYP
MAE	0.0820877	0.0341498
Slope	0.766447	0.883943
Intercept	0.00909909	0.0148559
R^2	0.934634	0.980412
$\|\Delta E\|$	0.631242	0.217386
ΔE	0.624592	0.0579021
E_{comp}/E_{exp}	1.14493	1.01089

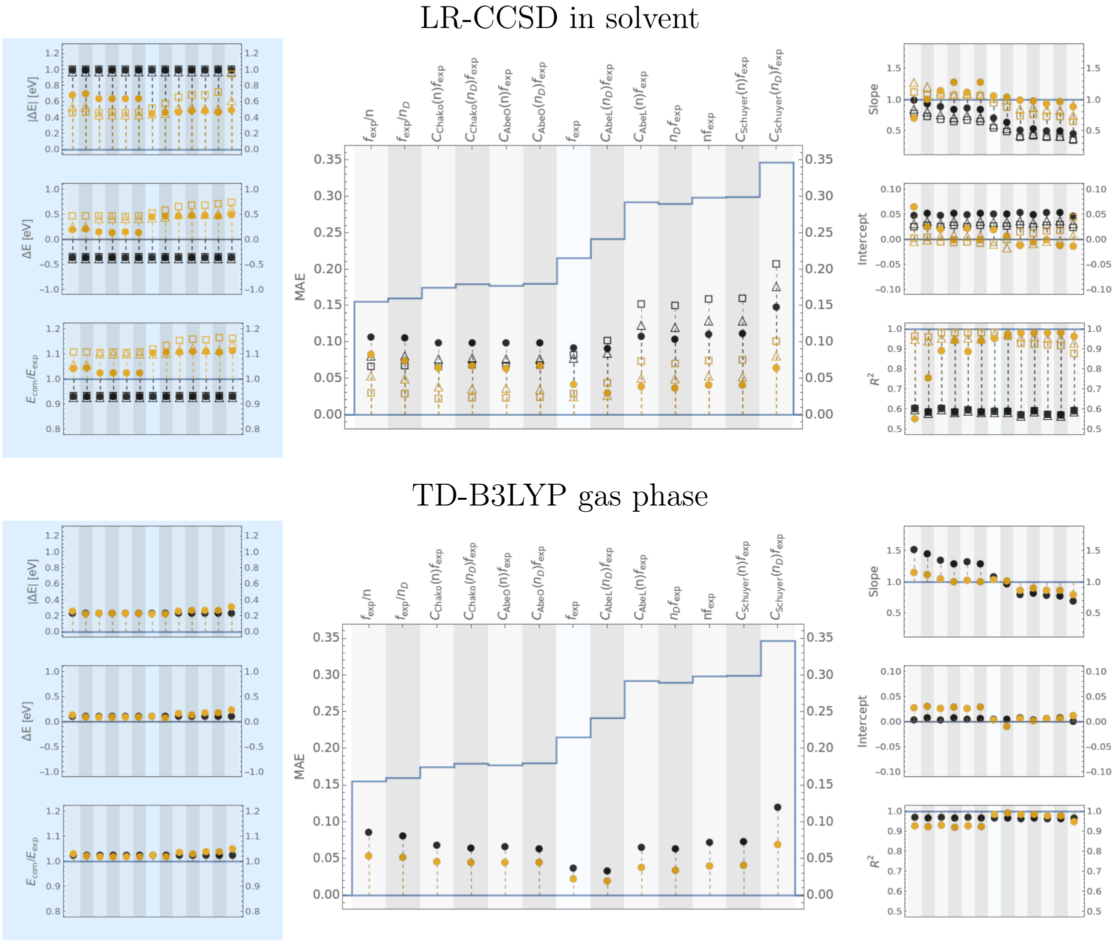

Figure S1: Comparison of f-values computed using LR-CCSD/aug-cc-pVDZ/PCM (top panel) and gas phase-RPA-TD-B3LYP/6-31+G* (bottom panel) for a subset of 35 experimental transitions multiplied by different cavity field corrections. The blue bar outline indicates the average values of f_{exp} multiplied by the respective cavity field factor. A full circle corresponds to the data obtained within the length gauge, an empty square corresponds to the velocity gauge, and an empty triangle corresponds to the mixed gauge. markers in black correspond to the Exact Band Limits comparison, while markers in yellow correspond to the Improved Fit comparison. The data displayed can be found in Tables S29 to S106.

S2.3 VHHM transitions for TD-B3LYP with different basis sets

S2.3.1 f_{exp}

Avg $f_{exp} = 0.307655$

Table S107: f_{comp} vs f_{exp} PCM/B3LYP/x-gauge/Exact Band Limits/

	STO-3G	3-21G	6-31G*	6-31++G**	6-311++G**	cc-pVDZ	Aug-CC-pVDZ	Aug-CC-pVTZ
MAE	0.232842	0.120941	0.115051	0.128638	0.123183	0.114964	0.123857	0.122698
Slope	0.434606	1.33882	1.35958	1.34644	1.34262	1.36031	1.3668	1.36219
Intercept	0.0389009	-0.0350706	-0.0197231	0.00750147	0.00257072	-0.0200312	-0.00445079	-0.00412828
R^2	0.142913	0.845121	0.887734	0.90369	0.904693	0.888938	0.903306	0.902779
$\|\Delta E\|$	1.64131	0.62728	0.339956	0.217404	0.206494	0.316317	0.205638	0.206899
ΔE	-1.20097	-0.349056	-0.106284	-0.105926	-0.0881267	-0.103626	-0.0936657	-0.0977201
E_{comp}/E_{exp}	0.736339	0.932553	0.978372	0.974433	0.978771	0.977097	0.977707	0.976976

Table S108: f_{comp} vs f_{exp} PCM/B3LYP/p-gauge/Exact Band Limits/

	STO-3G	3-21G	6-31G*	6-31++G**	6-311++G**	cc-pVDZ	Aug-CC-pVDZ	Aug-CC-pVTZ
MAE	0.237721	0.0824276	0.103318	0.108619	0.111934	0.103521	0.116279	0.121795
Slope	0.192575	0.956003	1.29708	1.26451	1.29656	1.30522	1.33746	1.35716
Intercept	0.0182723	-0.0276282	-0.0161218	0.0100928	0.00392091	-0.0165596	-0.00381143	-0.00360028
R^2	0.137336	0.833546	0.88208	0.898876	0.906879	0.88818	0.902508	0.902072
$\|\Delta E\|$	1.43767	0.618475	0.339433	0.219998	0.207702	0.274989	0.20564	0.206881
ΔE	-0.987693	-0.350883	-0.108898	-0.107852	-0.0897491	-0.0617384	-0.0936542	-0.0977202
E_{comp}/E_{exp}	0.804658	0.932235	0.977703	0.973916	0.97836	0.988587	0.977709	0.976977

Table S109: f_{comp} vs f_{exp} PCM/B3LYP/xp-gauge/Exact Band Limits/

	STO-3G	3-21G	6-31G*	6-31++G**	6-311++G**	cc-pVDZ	Aug-CC-pVDZ	Aug-CC-pVTZ
MAE	0.220985	0.0856582	0.108606	0.118309	0.117361	0.108764	0.120011	0.122242
Slope	0.286907	1.13024	1.32816	1.30491	1.3194	1.33227	1.35201	1.3597
Intercept	0.0243342	-0.0327748	-0.0183424	0.00858362	0.00309449	-0.0184853	-0.00413431	-0.00388071
R^2	0.142015	0.841202	0.885234	0.901533	0.905994	0.888599	0.902921	0.902425
$\|\Delta E\|$	1.39826	0.624643	0.339675	0.218808	0.207156	0.274611	0.205657	0.206902
ΔE	-0.946746	-0.34937	-0.107673	-0.107019	-0.0889781	-0.061058	-0.09368	-0.0977239
E_{comp}/E_{exp}	0.804601	0.932461	0.97801	0.974145	0.978553	0.988769	0.977704	0.976975

Improving fit:

Table S110: f_{comp} vs f_{exp} PCM/B3LYP/x-gauge/Improved Fit/

	STO-3G	3-21G	6-31G*	6-31++G**	6-311++G**	cc-pVDZ	Aug-CC-pVDZ	Aug-CC-pVTZ
MAE	0.0918524	0.0708429	0.0692688	0.0668935	0.0666347	0.0697924	0.0633606	0.0623488
Slope	1.13287	1.17607	1.16716	1.12424	1.12441	1.11326	1.12229	1.12249
Intercept	-0.0121185	-0.00597078	-0.00179619	0.0160586	0.0135823	0.0149779	0.0121398	0.00945525
R^2	0.790428	0.893164	0.902629	0.915356	0.913598	0.875359	0.914878	0.914621
$\|\Delta E\|$	0.9702	0.419987	0.33909	0.290802	0.277012	0.262736	0.272036	0.274715
ΔE	0.639609	0.0820845	0.0420937	-0.0356222	-0.0275795	0.0602807	-0.0276803	-0.0321343
E_{comp}/E_{exp}	1.13906	1.01793	1.00861	0.994036	0.996205	1.01221	0.996082	0.995329

Table S111: f_{comp} vs f_{exp} PCM/B3LYP/p-gauge/Improved Fit/

	STO-3G	3-21G	6-31G*	6-31++G**	6-311++G**	cc-pVDZ	Aug-CC-pVDZ	Aug-CC-pVTZ
MAE	0.0856688	0.0371676	0.0585559	0.0541018	0.0582112	0.0617971	0.0601088	0.0620147
Slope	0.750172	1.03683	1.11839	1.07937	1.09631	1.14465	1.09338	1.11198
Intercept	0.00260372	-0.0237966	0.000758339	0.0153582	0.0126148	-0.00162632	0.016629	0.0133351
R^2	0.720358	0.954429	0.910917	0.927972	0.924949	0.910926	0.915054	0.913471
$\|\Delta E\|$	1.3414	0.525759	0.352072	0.299517	0.278717	0.288521	0.268112	0.279309
ΔE	1.1974	0.0918475	0.0443443	-0.0300241	-0.0282637	0.0521431	-0.0448689	-0.0458105
E_{comp}/E_{exp}	1.25414	1.02079	1.00926	0.995246	0.995986	1.011	0.991867	0.99183

Table S112: f_{comp} vs f_{exp} PCM/B3LYP/xp-gauge/Improved Fit/

	STO-3G	3-21G	6-31G*	6-31++G**	6-311++G**	cc-pVDZ	Aug-CC-pVDZ	Aug-CC-pVTZ
MAE	0.0494806	0.0373371	0.0632782	0.0608688	0.0614041	0.0661112	0.0623076	0.0620747
Slope	1.01522	1.06463	1.13736	1.10266	1.1122	1.15564	1.1026	1.1135
Intercept	-0.029195	-0.0100892	0.00116144	0.0148368	0.0139091	0.000411057	0.0148941	0.0135109
R^2	0.908671	0.938231	0.90628	0.921741	0.922766	0.903174	0.911281	0.913902
$\|\Delta E\|$	1.13651	0.473442	0.344634	0.293912	0.27195	0.283298	0.279313	0.278186
ΔE	0.841452	0.0453283	0.059347	-0.0345827	-0.0215718	0.0664365	-0.0402439	-0.0446565
E_{comp}/E_{exp}	1.19084	1.00978	1.0131	0.99432	0.997225	1.0146	0.99324	0.992081

S2.3.2 nf_{exp}

Avg $nf_{exp} = 0.433273$

Table S113: f_{comp} vs nf_{exp} PCM/B3LYP/x-gauge/Exact Band Limits/

	STO-3G	3-21G	6-31G*	6-31++G**	6-311++G**	cc-pVDZ	Aug-CC-pVDZ	Aug-CC-pVTZ
MAE	0.30873	0.103209	0.0825775	0.0761589	0.0755225	0.0846947	0.0752227	0.0761439
Slope	0.289747	0.942511	0.954068	0.942522	0.939602	0.953446	0.957298	0.954059
Intercept	0.0470699	-0.0315398	-0.0148131	0.0133709	0.00852896	-0.014628	0.00128073	0.00158883
R^2	0.128488	0.847208	0.88425	0.895721	0.89625	0.88335	0.89632	0.895776
$\|\Delta E\|$	1.64131	0.62728	0.339956	0.217404	0.206494	0.316317	0.205638	0.206899
ΔE	-1.20097	-0.349056	-0.106284	-0.105926	-0.0881267	-0.103626	-0.0936657	-0.0977201
E_{comp}/E_{exp}	0.736339	0.932553	0.978372	0.974433	0.978771	0.977097	0.977707	0.976976

Table S114: f_{comp} vs nf_{exp} PCM/B3LYP/p-gauge/Exact Band Limits/

	STO-3G	3-21G	6-31G*	6-31++G**	6-311++G**	cc-pVDZ	Aug-CC-pVDZ	Aug-CC-pVTZ
MAE	0.357277	0.168366	0.0893846	0.0832754	0.0772834	0.0876615	0.0773742	0.0766331
Slope	0.12835	0.674736	0.910732	0.885253	0.907844	0.915247	0.93673	0.950514
Intercept	0.0219084	-0.0258542	-0.0116646	0.0155697	0.00946879	-0.011553	0.00180516	0.00210354
R^2	0.123401	0.839892	0.87963	0.891114	0.899357	0.883389	0.895493	0.895039
$\|\Delta E\|$	1.43767	0.618475	0.339433	0.219998	0.207702	0.274989	0.20564	0.206881
ΔE	-0.987693	-0.350883	-0.108898	-0.107852	-0.0897491	-0.0617384	-0.0936542	-0.0977202
E_{comp}/E_{exp}	0.804658	0.932235	0.977703	0.973916	0.97836	0.988587	0.977709	0.976977

Table S115: f_{comp} vs nf_{exp} PCM/B3LYP/xp-gauge/Exact Band Limits/

	STO-3G	3-21G	6-31G*	6-31++G**	6-311++G**	cc-pVDZ	Aug-CC-pVDZ	Aug-CC-pVTZ
MAE	0.332735	0.129794	0.0855018	0.0796898	0.0762686	0.0859514	0.0762519	0.0763813
Slope	0.191261	0.796726	0.932282	0.913491	0.923592	0.934008	0.946929	0.952307
Intercept	0.0297342	-0.0302501	-0.0136597	0.014255	0.00884541	-0.013285	0.00153912	0.00182993
R^2	0.127659	0.845511	0.882256	0.893659	0.898007	0.883413	0.895921	0.895407
$\|\Delta E\|$	1.39826	0.624643	0.339675	0.218808	0.207156	0.274611	0.205657	0.206902
ΔE	-0.946746	-0.34937	-0.107673	-0.107019	-0.0889781	-0.061058	-0.09368	-0.0977239
E_{comp}/E_{exp}	0.804601	0.932461	0.97801	0.974145	0.978553	0.988769	0.977704	0.976975

Improving fit:

Table S116: f_{comp} vs nf_{exp} PCM/B3LYP/x-gauge/Improved Fit/

	STO-3G	3-21G	6-31G*	6-31++G**	6-311++G**	cc-pVDZ	Aug-CC-pVDZ	Aug-CC-pVTZ
MAE	0.0764731	0.0508747	0.0483498	0.0404331	0.0391528	0.0489926	0.0422971	0.0448606
Slope	1.0268	1.05954	1.04029	0.989935	0.976065	0.99963	0.969688	0.967175
Intercept	-0.0318372	-0.0367691	-0.0285461	-0.00125085	-0.00054238	-0.0127713	-0.00132308	-0.00444017
R^2	0.898241	0.954949	0.957262	0.965158	0.968661	0.95021	0.965669	0.963342
$\|\Delta E\|$	1.07931	0.504525	0.280767	0.228552	0.224095	0.271506	0.231046	0.232109
ΔE	0.739521	0.0771543	0.123719	-0.0590833	-0.0238259	0.108696	-0.0347895	-0.0448323
E_{comp}/E_{exp}	1.16145	1.01664	1.02432	0.984355	0.995479	1.02156	0.993037	0.991244

Table S117: f_{comp} vs nf_{exp} PCM/B3LYP/p-gauge/Improved Fit/

	STO-3G	3-21G	6-31G*	6-31++G**	6-311++G**	cc-pVDZ	Aug-CC-pVDZ	Aug-CC-pVTZ
MAE	0.155638	0.0730255	0.0429804	0.0466229	0.0410876	0.0477716	0.0418347	0.0456865
Slope	0.612318	0.863335	1.02227	0.949203	0.958277	1.03118	0.957082	0.967268
Intercept	0.0168948	-0.00738767	-0.0196547	0.00609493	0.00452443	-0.0295985	0.0018002	-0.00360758
R^2	0.651987	0.92695	0.960235	0.958877	0.965987	0.955439	0.964956	0.962498
$\|\Delta E\|$	1.49936	0.613356	0.307438	0.226953	0.227143	0.285547	0.222116	0.230322
ΔE	1.386	0.429644	0.173025	-0.0513512	-0.00770753	0.126112	-0.0230686	-0.0402727
E_{comp}/E_{exp}	1.30537	1.09357	1.03484	0.985731	0.998739	1.02536	0.99578	0.992101

Table S118: f_{comp} vs nf_{exp} PCM/B3LYP/xp-gauge/Improved Fit/

	STO-3G	3-21G	6-31G*	6-31++G**	6-311++G**	cc-pVDZ	Aug-CC-pVDZ	Aug-CC-pVTZ
MAE	0.106642	0.0550253	0.0462714	0.0439822	0.0390712	0.0487142	0.0416947	0.0449159
Slope	0.798906	0.969048	1.03287	0.965462	0.965733	1.04044	0.966128	0.969752
Intercept	-0.00328089	-0.0293717	-0.0238241	0.00284	0.00195063	-0.0297765	0.00114753	-0.00368944
R^2	0.777821	0.949021	0.957984	0.962841	0.968159	0.954974	0.96516	0.963301
$\|\Delta E\|$	1.21924	0.61194	0.316552	0.223365	0.223578	0.273019	0.222792	0.22775
ΔE	1.07473	0.171049	0.158012	-0.0609394	-0.0141064	0.112832	-0.0241922	-0.038628
E_{comp}/E_{exp}	1.23147	1.03742	1.03165	0.98355	0.997273	1.02244	0.995493	0.992439

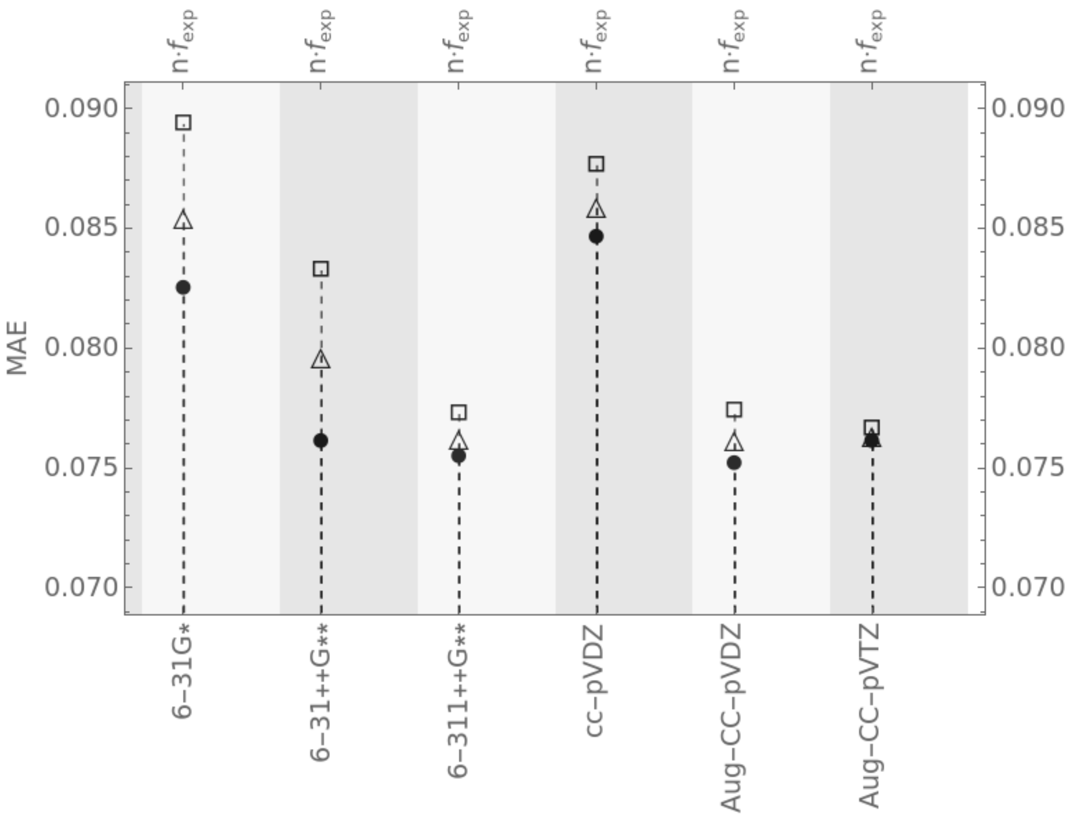

Figure S2: Comparison of f-values computed using TD-B3LYP/PCM and the basis sets 6-31G*, 6-31++G**, 6-311++G**, cc-pVDZ, Aug-CC-pVDZ, and Aug-CC-pVTZ to nf_{exp}. A full circle corresponds to the data obtained within the length gauge, an empty square corresponds to the velocity gauge, and an empty triangle corresponds to the mixed gauge. markers in black correspond to the Exact Band Limits comparison.

S2.4 VHHM transitions with the 9 functionals and 6-311++G**

S2.4.1 f_{exp}

Avg f_{exp} = 0.307655

Table S119: f_{comp} vs f_{exp} PCM/TD-DFT/6-311++G**/x-gauge/Exact Band Limits/

	B3P86	CAM-B3LYP	LC-wHPBE	M05	mPW1PW91	O3LYP	SVWN	wB97XD	B3LYP
MAE	0.125308	0.161828	0.189303	0.120583	0.129181	0.116584	0.128561	0.169114	0.123183
Slope	1.34349	1.4039	1.36558	1.3307	1.33424	1.31497	1.1614	1.44972	1.34262
Intercept	0.00297854	0.0132073	-0.0148119	0.00395011	0.0120865	-0.00213168	0.0116485	0.00627363	0.00257072
R^2	0.900338	0.883378	0.751487	0.901576	0.89466	0.882307	0.776069	0.886512	0.904693
$\|\Delta E\|$	0.267212	0.394401	0.840982	0.197738	0.236381	0.24126	0.371319	0.417156	0.206494
ΔE	-0.125991	-0.0148852	-0.302042	-0.0494465	-0.0522883	-0.160141	-0.171311	0.012057	-0.0881267
E_{comp}/E_{exp}	0.973611	1.00674	0.943726	0.989017	0.988398	0.961859	0.964925	1.01332	0.978771

Table S120: f_{comp} vs f_{exp} PCM/TD-DFT/6-311++G**/p-gauge/Exact Band Limits/

	B3P86	CAM-B3LYP	LC-wHPBE	M05	mPW1PW91	O3LYP	SVWN	wB97XD	B3LYP
MAE	0.113485	0.148263	0.176303	0.121249	0.117041	0.106569	0.117958	0.155722	0.111934
Slope	1.29427	1.34832	1.30941	1.32523	1.28458	1.27385	1.12769	1.39326	1.29656
Intercept	0.004653	0.0162971	-0.011011	0.00576729	0.0134527	-0.00114179	0.0100149	0.00953733	0.00392091
R^2	0.902434	0.885266	0.75161	0.903461	0.897842	0.885523	0.784998	0.887363	0.906879
$\|\Delta E\|$	0.268123	0.392473	0.840408	0.197986	0.236283	0.242676	0.408235	0.415278	0.207702
ΔE	-0.127555	-0.0160943	-0.303026	-0.0507995	-0.0539446	-0.159823	-0.216914	0.0107651	-0.0897491
E_{comp}/E_{exp}	0.973215	1.00642	0.943494	0.988662	0.987971	0.96193	0.952272	1.01298	0.97836

Table S121: f_{comp} vs f_{exp} PCM/TD-DFT/6-311++G**/Exact Band Limits/VH-H-M transitions/

	B3P86	CAM-B3LYP	LC-wHPBE	M05	mPW1PW91	O3LYP	SVWN	wB97XD	B3LYP
MAE	0.119176	0.154799	0.182549	0.120686	0.122905	0.111277	0.123106	0.162032	0.117361
Slope	1.31871	1.37595	1.33729	1.328	1.30923	1.29425	1.14437	1.42128	1.3194
Intercept	0.00364954	0.0145949	-0.0130561	0.00470057	0.0126095	-0.00178345	0.0106714	0.00774052	0.00309449
R^2	0.901638	0.884665	0.75192	0.902742	0.896506	0.884103	0.780784	0.887278	0.905994
$\|\Delta E\|$	0.267728	0.393432	0.840715	0.19778	0.236399	0.241812	0.408858	0.416206	0.207156
ΔE	-0.126832	-0.0155037	-0.302507	-0.0501597	-0.0531243	-0.159976	-0.217668	0.0114041	-0.0889781
E_{comp}/E_{exp}	0.973396	1.00657	0.943615	0.98883	0.988182	0.961896	0.952086	1.01315	0.978553

Improving fit:

Table S122: f_{comp} vs f_{exp} PCM/TD-DFT/6-311++G**/x-gauge/improved fit/ VH, H, and M transitions

	B3P86	CAM-B3LYP	LC-wHPBE	M05	mPW1PW91	O3LYP	SVWN	wB97XD	B3LYP
MAE	0.0683912	0.105142	0.126254	0.0748853	0.0721288	0.0559629	0.0495065	0.113471	0.0666347
Slope	1.11389	1.10046	1.1485	1.07972	1.12143	1.11554	1.04769	1.16556	1.12441
Intercept	0.0206683	0.0341067	0.0154848	0.0207848	0.0231636	0.00732262	-0.00595192	0.0233138	0.0135823
R^2	0.908498	0.801503	0.761944	0.853199	0.901028	0.923477	0.883053	0.823271	0.913598
$\|\Delta E\|$	0.282894	0.347361	0.781892	0.303688	0.304812	0.368004	0.461721	0.442827	0.277012
ΔE	0.00903122	0.121578	-0.0501622	-0.0962736	0.0306401	-0.15682	-0.245257	0.0720868	-0.0275795
E_{comp}/E_{exp}	1.00378	1.02521	0.986949	0.974725	1.01069	0.961608	0.945918	1.01885	0.996205

Table S123: f_{comp} vs f_{exp} PCM/TD-DFT/6-311++G**/p-gauge/improved fit/ VH, H, and M transitions

	B3P86	CAM-B3LYP	LC-wHPBE	M05	mPW1PW91	O3LYP	SVWN	wB97XD	B3LYP
MAE	0.0592935	0.0959747	0.114205	0.0725641	0.0639688	0.0550312	0.0422465	0.102416	0.0582112
Slope	1.07636	1.0533	1.10025	1.13038	1.10012	1.10701	1.02872	1.12024	1.09631
Intercept	0.0203401	0.0439876	0.018235	0.0159576	0.0203559	0.00513034	-0.00506245	0.0306936	0.0126148
R^2	0.917438	0.817717	0.773115	0.891946	0.915731	0.888577	0.89608	0.836373	0.924949
$\|\Delta E\|$	0.279915	0.312058	0.731059	0.231151	0.296213	0.360923	0.466548	0.442672	0.278717
ΔE	-0.00789901	0.164248	-0.0143982	-0.0174626	0.0119985	-0.161076	-0.23982	0.0899827	-0.0282637
E_{comp}/E_{exp}	0.999431	1.04087	0.997125	0.996909	1.00635	0.961372	0.947486	1.02386	0.995986

Table S124: f_{comp} vs f_{exp} PCM/TD-DFT/6-311++G**/xp-gauge/improved fit/ VH, H, and M transitions

	B3P86	CAM-B3LYP	LC-wHPBE	M05	mPW1PW91	O3LYP	SVWN	wB97XD	B3LYP
MAE	0.0635912	0.100895	0.119842	0.0737853	0.0721594	0.0579876	0.0457618	0.107349	0.0614041
Slope	1.09854	1.07345	1.1218	1.13157	1.16756	1.11915	1.04144	1.14013	1.1122
Intercept	0.0191743	0.0437681	0.0183711	0.017231	0.00548506	0.00569392	-0.0065831	0.031401	0.0139091
R^2	0.91403	0.812047	0.768025	0.888021	0.917397	0.882112	0.889138	0.833397	0.922766
$\|\Delta E\|$	0.283847	0.339022	0.739029	0.277394	0.303622	0.361169	0.461477	0.438095	0.27195
ΔE	-0.00437572	0.124018	0.00240534	-0.0695449	0.0233414	-0.156054	-0.240542	0.0964371	-0.0215718
E_{comp}/E_{exp}	1.00001	1.02649	1.00056	0.985047	1.00906	0.962289	0.947052	1.02497	0.997225

S2.4.2 nf_{exp}

Avg $nf_{exp} = 0.433273$

Table S125: f_{comp} vs nf_{exp} PCM/TD-DFT/6-311++G**/x-gauge/Experimental Band Limits/ VH, H, and M Transitions

	B3P86	CAM-B3LYP	LC-wHPBE	M05	mPW1PW91	O3LYP	SVWN	wB97XD	B3LYP
MAE	0.0769869	0.0848549	0.134026	0.0752613	0.0765608	0.0886112	0.130961	0.0860396	0.0755225
Slope	0.940019	0.981721	0.957231	0.931653	0.93303	0.918728	0.812492	1.01491	0.939602
Intercept	0.00902454	0.0197694	-0.00942818	0.00968458	0.018314	0.00436619	0.0169279	0.0125524	0.00852896
R^2	0.891569	0.873771	0.746908	0.89392	0.884969	0.871172	0.768279	0.878858	0.89625
$\|\Delta E\|$	0.267212	0.394401	0.840982	0.197738	0.236381	0.24126	0.371319	0.417156	0.206494
ΔE	-0.125991	-0.0148852	-0.302042	-0.0494465	-0.0522883	-0.160141	-0.171311	0.012057	-0.0881267
E_{comp}/E_{exp}	0.973611	1.00674	0.943726	0.989017	0.988398	0.961859	0.964925	1.01332	0.978771

Table S126: f_{comp} vs nf_{exp} PCM/TD-DFT/6-311++G**/p-gauge/Experimental Band Limits/ VH, H, and M Transitions

	B3P86	CAM-B3LYP	LC-wHPBE	M05	mPW1PW91	O3LYP	SVWN	wB97XD	B3LYP
MAE	0.0787046	0.0815547	0.132716	0.0755594	0.0773646	0.0888055	0.130646	0.0821269	0.0772834
Slope	0.906043	0.943266	0.918386	0.928298	0.898797	0.890434	0.789266	0.975822	0.907844
Intercept	0.0102785	0.0224226	-0.00607624	0.0112739	0.0192355	0.00496274	0.0149879	0.0153817	0.00946879
R^2	0.894551	0.876396	0.747886	0.896699	0.889089	0.87521	0.777816	0.880492	0.899357
$\|\Delta E\|$	0.268123	0.392473	0.840408	0.197986	0.236283	0.242676	0.408235	0.415278	0.207702
ΔE	-0.127555	-0.0160943	-0.303026	-0.0507995	-0.0539446	-0.159823	-0.216914	0.0107651	-0.0897491
E_{comp}/E_{exp}	0.973215	1.00642	0.943494	0.988662	0.987971	0.96193	0.952272	1.01298	0.97836

Table S127: f_{comp} vs nf_{exp} PCM/TD-DFT/6-311++G**/xp-gauge/Experimental Band Limits/ VH, H, and M Transitions

	B3P86	CAM-B3LYP	LC-wHPBE	M05	mPW1PW91	O3LYP	SVWN	wB97XD	B3LYP
MAE	0.0776201	0.082474	0.132792	0.0753364	0.0763622	0.088266	0.130683	0.0836928	0.0762686
Slope	0.922913	0.962388	0.93767	0.929997	0.915787	0.904474	0.800761	0.995226	0.923592
Intercept	0.00948321	0.0209364	-0.00789997	0.0103221	0.0186134	0.004516	0.0157956	0.0138003	0.00884541
R^2	0.893307	0.875421	0.747766	0.895526	0.887273	0.873374	0.773295	0.880009	0.898007
$\|\Delta E\|$	0.267728	0.393432	0.840715	0.19778	0.236399	0.241812	0.408858	0.416206	0.207156
ΔE	-0.126832	-0.0155037	-0.302507	-0.0501597	-0.0531243	-0.159976	-0.217668	0.0114041	-0.0889781
E_{comp}/E_{exp}	0.973396	1.00657	0.943615	0.98883	0.988182	0.961896	0.952086	1.01315	0.978553

improving fit:

Table S128: f_{comp} vs nf_{exp} PCM/TD-DFT/6-311++G**/x-gauge/Improved Fit/ VH, H, and M Transitions

	B3P86	CAM-B3LYP	LC-wHPBE	M05	mPW1PW91	O3LYP	SVWN	wB97XD	B3LYP
MAE	0.0413646	0.0513728	0.0784935	0.0439307	0.04167	0.0494782	0.0502368	0.0551441	0.0391528
Slope	0.982647	1.03505	1.05449	1.00309	1.01157	0.928555	0.929425	1.03138	0.976065
Intercept	-0.00204714	0.0122819	-0.00729061	-0.00551648	-0.00256715	0.00890369	0.00386762	0.0150352	-0.00054238
R^2	0.964821	0.944193	0.889362	0.958231	0.961125	0.948117	0.9202	0.937447	0.968661
$\|\Delta E\|$	0.224505	0.268943	0.49767	0.211299	0.216423	0.248828	0.383391	0.307001	0.224095
ΔE	0.0123462	0.233475	0.34986	-0.000715446	0.0542622	-0.11331	-0.21022	0.277441	-0.0238259
E_{comp}/E_{exp}	1.00355	1.05564	1.08526	0.999165	1.0112	0.970398	0.950891	1.06604	0.995479

Table S129: f_{comp} vs nf_{exp} PCM/TD-DFT/6-311++G**/p-gauge/Improved Fit/ VH, H, and M transitions

	B3P86	CAM-B3LYP	LC-wHPBE	M05	mPW1PW91	O3LYP	SVWN	wB97XD	B3LYP
MAE	0.0426192	0.0465752	0.0744542	0.0412502	0.0407121	0.0528156	0.0507693	0.0513992	0.0410876
Slope	1.01393	1.0157	1.03618	0.997124	0.984502	0.919918	0.914337	1.02525	0.958277
Intercept	-0.0173025	0.0104148	-0.0089672	-0.00366421	0.00107279	0.0119927	0.00598519	0.0113756	0.00452443
R^2	0.964273	0.951114	0.897991	0.960782	0.963724	0.924259	0.919203	0.946185	0.965987
$\|\Delta E\|$	0.231871	0.278836	0.607425	0.21693	0.215451	0.253107	0.381661	0.309465	0.227143
ΔE	0.0313932	0.246636	0.246617	0.0163878	0.0688027	-0.086868	-0.201638	0.280328	-0.00770753
E_{comp}/E_{exp}	1.00711	1.0579	1.06215	1.00381	1.01407	0.976783	0.952932	1.06609	0.998739

Table S130: f_{comp} vs nf_{exp} PCM/TD-DFT/6-311++G**/xp-gauge/Improved Fit/ VH, H, and M transitions

	B3P86	CAM-B3LYP	LC-wHPBE	M05	mPW1PW91	O3LYP	SVWN	wB97XD	B3LYP
MAE	0.040738	0.048254	0.0762086	0.0420199	0.0408001	0.0558434	0.0513639	0.0531827	0.0390712
Slope	0.967029	1.02895	1.04809	0.996396	0.99888	0.939012	0.920357	1.03083	0.965733
Intercept	-0.0003369	0.011841	-0.00895172	-0.00228191	-0.000661558	0.00511523	0.00354272	0.0110701	0.00195063
R^2	0.965121	0.949422	0.894179	0.959912	0.962921	0.920377	0.919723	0.94211	0.968159
$\|\Delta E\|$	0.23131	0.279927	0.561143	0.207272	0.218666	0.252188	0.385537	0.298525	0.223578
ΔE	0.0190046	0.247965	0.307695	0.00441583	0.0651715	-0.102854	-0.213525	0.264796	-0.0141064
E_{comp}/E_{exp}	1.00472	1.05831	1.07576	1.00052	1.01347	0.97337	0.950338	1.06252	0.997273

S2.4.3 Cf_{exp}

For the VH, H, and M subset:

Table S131: f_{comp} vs Cf_{exp} PCM/TD-DFT/6-311++G**/x-gauge/Improved Fit/

	B3P86	CAM-B3LYP	LC-wHPBE	M05	mPW1PW91	O3LYP	SVWN	wB97XD	B3LYP
MAE	0.0378776	0.0469059	0.0721389	0.0410794	0.038819	0.0437835	0.0357417	0.0483425	0.0346995
Slope	1.00996	1.00113	1.01393	1.01249	1.00673	1.01273	1.01197	1.0007	1.00838
Intercept	-0.00697017	-0.00245038	-0.011297	-0.009075	-0.00500206	-0.00896412	-0.00722108	-0.000580686	-0.00585291
R^2	0.968476	0.959099	0.90998	0.962116	0.967211	0.955963	0.930266	0.958415	0.971987
$\|\Delta E\|$	0.25967	0.28122	0.606304	0.21544	0.209003	0.25638	0.431879	0.317361	0.24869
ΔE	0.0152818	0.247192	0.257118	-0.00963995	0.0555093	-0.128588	-0.212971	0.289161	-0.0318023
E_{comp}/E_{exp}	1.00552	1.05888	1.06375	0.997622	1.01186	0.968389	0.952672	1.06817	0.995097

Table S132: f_{comp} vs Cf_{exp} PCM/TD-DFT/6-311++G**/p-gauge/Improved Fit/

	B3P86	CAM-B3LYP	LC-wHPBE	M05	mPW1PW91	O3LYP	SVWN	wB97XD	B3LYP
MAE	0.035389	0.0441699	0.0721313	0.0384171	0.0359406	0.0439364	0.0348478	0.0470636	0.0316732
Slope	1.00142	0.995775	1.02219	1.00891	1.00318	1.01007	1.01337	0.995964	1.00696
Intercept	-0.000954618	0.00329002	-0.0170795	-0.00640788	-0.00227225	-0.00657612	-0.00774399	0.00323103	-0.00473575
R^2	0.96824	0.960907	0.907945	0.964358	0.967992	0.931781	0.930849	0.959644	0.973144
$\|\Delta E\|$	0.250386	0.283259	0.574073	0.218465	0.211098	0.252796	0.483529	0.319376	0.233696
ΔE	0.00849815	0.246546	0.320621	0.00397407	0.0552332	-0.124264	-0.244423	0.287721	-0.0347352
E_{comp}/E_{exp}	1.00369	1.05776	1.07822	1.0016	1.0117	0.969884	0.946611	1.06716	0.99384

Table S133: f_{comp} vs Cf_{exp} PCM/TD-DFT/6-311++G**/xp-gauge/Improved Fit/

	B3P86	CAM-B3LYP	LC-wHPBE	M05	mPW1PW91	O3LYP	SVWN	wB97XD	B3LYP
MAE	0.03686	0.0448415	0.0733158	0.0390227	0.0370933	0.0473826	0.0347494	0.0473708	0.0331181
Slope	1.00422	0.998995	1.02303	1.00731	1.00458	1.01524	1.01541	1.00054	1.00656
Intercept	-0.00289392	0.000798306	-0.0185877	-0.00528881	-0.00333712	-0.0101018	-0.00914438	-0.00043978	-0.00450256
R^2	0.968196	0.96091	0.913087	0.963838	0.967638	0.927887	0.931731	0.96008	0.972161
$\|\Delta E\|$	0.253013	0.288212	0.62515	0.208252	0.210635	0.255504	0.424648	0.31438	0.250385
ΔE	0.00728703	0.256498	0.275955	-0.00694763	0.0558737	-0.121628	-0.219741	0.282805	-0.0310823
E_{comp}/E_{exp}	1.00361	1.06053	1.06795	0.998453	1.01184	0.970444	0.950755	1.0661	0.99516

S3 Other Results not presented in the manuscript

S3.1 All 164 Transitions with 9 functionals and 6-311++G**

S3.1.1 f_{exp}

Avg $f_{exp} = 0.329294$

Table S134: f_{comp} vs f_{exp} PCM/TD-DFT/6-311++G**/x-gauge/exact band limits/ All transitions

	B3P86	CAM-B3LYP	LC-wHPBE	M05	mPW1PW91	O3LYP	SVWN	wB97XD	B3LYP
MAE	0.130895	0.166517	0.194331	0.131648	0.135154	0.126357	0.134156	0.172234	0.128236
Slope	1.29279	1.33736	1.0287	1.30564	1.29563	1.21858	1.11695	1.35237	1.28604
Intercept	0.00402575	0.00246202	0.0194182	0.00358707	0.00685225	0.0105741	0.0244106	-0.00523267	0.00924303
R^2	0.877859	0.825022	0.574265	0.881501	0.879489	0.820641	0.770402	0.807458	0.890169
$\|\Delta E\|$	0.27672	0.577637	1.03818	0.221167	0.335024	0.24893	0.362163	0.572072	0.213816
ΔE	-0.156533	-0.264226	-0.626751	-0.101035	-0.18835	-0.186817	-0.222118	-0.223662	-0.122091
E_{comp}/E_{exp}	0.968097	0.95162	0.874569	0.980235	0.961292	0.960299	0.956838	0.961299	0.974292

Table S135: Table: f_{comp} vs f_{exp} PCM/TD-DFT/6-311++G**/p-gauge/exact band limits/ All transitions

	B3P86	CAM-B3LYP	LC-wHPBE	M05	mPW1PW91	O3LYP	SVWN	wB97XD	B3LYP
MAE	0.120303	0.155458	0.18337	0.133256	0.123984	0.117018	0.125212	0.16119	0.117901
Slope	1.257	1.29773	0.99196	1.31167	1.26087	1.19053	1.09181	1.31234	1.25307
Intercept	0.00342219	0.0028577	0.0199815	0.00313284	0.00594204	0.00919618	0.0211256	-0.00459791	0.00853882
R^2	0.880307	0.826777	0.581297	0.884751	0.883308	0.827377	0.777391	0.809465	0.893569
$\|\Delta E\|$	0.277263	0.576722	1.03757	0.221314	0.335193	0.249851	0.352443	0.571368	0.21487
ΔE	-0.157505	-0.26564	-0.627582	-0.101922	-0.189368	-0.187105	-0.217427	-0.225486	-0.123404
E_{comp}/E_{exp}	0.967857	0.951299	0.874382	0.980013	0.961038	0.960256	0.956161	0.960903	0.973982

Table S136: f_{comp} vs f_{exp} PCM/TD-DFT/6-311++G**/xp-gauge/exact band limits/ All transitions

	B3P86	CAM-B3LYP	LC-wHPBE	M05	mPW1PW91	O3LYP	SVWN	wB97XD	B3LYP
MAE	0.125381	0.16072	0.188597	0.132259	0.129335	0.121451	0.129346	0.16642	0.122886
Slope	1.27471	1.31743	1.01017	1.30866	1.27811	1.20443	1.10417	1.33221	1.26942
Intercept	0.00356884	0.00248394	0.0195539	0.00319342	0.00622484	0.00972141	0.0225957	-0.00508928	0.00872451
R^2	0.879288	0.826156	0.578044	0.883307	0.881631	0.824166	0.774053	0.808715	0.892074
$\|\Delta E\|$	0.277035	0.577181	1.03789	0.221221	0.335145	0.249323	0.352981	0.571778	0.214368
ΔE	-0.157046	-0.265012	-0.627177	-0.101509	-0.188856	-0.187005	-0.217754	-0.224607	-0.122755
E_{comp}/E_{exp}	0.967968	0.951444	0.874473	0.980116	0.961165	0.960269	0.956068	0.961094	0.974133

Improving fit:

Table S137: f_{comp} vs f_{exp} PCM/TD-DFT/6-311++G**/x-gauge/improved fit/ All transitions

	B3P86	CAM-B3LYP	LC-wHPBE	M05	mPW1PW91	O3LYP	SVWN	wB97XD	B3LYP
MAE	0.0633302	0.100321	0.119542	0.062596	0.067768	0.0494369	0.0495143	0.108374	0.0624869
Slope	1.06749	1.09297	1.02785	1.04593	1.08107	1.05574	0.993139	1.02022	1.08797
Intercept	0.0219101	0.0192859	0.0188102	0.0137836	0.0193723	0.00905147	0.0044957	0.0389784	0.0109999
R^2	0.904498	0.809798	0.745298	0.886594	0.893501	0.922608	0.900486	0.772836	0.907345
$\|\Delta E\|$	0.245964	0.381669	0.747289	0.251954	0.301359	0.301755	0.398692	0.453044	0.242175
ΔE	0.012912	0.0597698	-0.0939194	-0.0568199	-0.00660769	-0.130613	-0.189536	0.00528189	-0.0306837
E_{comp}/E_{exp}	1.00251	1.01266	0.972506	0.98565	0.999196	0.968887	0.959358	1.00181	0.993828

Table S138: f_{comp} vs f_{exp} PCM/TD-DFT/6-311++G**/p-gauge/improved fit/ All transitions

	B3P86	CAM-B3LYP	LC-wHPBE	M05	mPW1PW91	O3LYP	SVWN	wB97XD	B3LYP
MAE	0.055482	0.092604	0.108018	0.0612747	0.0599149	0.0478204	0.0486582	0.0948497	0.0566168
Slope	1.05521	1.04999	1.00409	1.06282	1.06186	1.05013	0.985271	1.03818	1.06585
Intercept	0.0172238	0.0330416	0.0181229	0.0141298	0.0170395	0.00729542	0.00414178	0.0358835	0.010663
R^2	0.919013	0.822973	0.765802	0.89975	0.904033	0.908514	0.891145	0.823215	0.917368
$\|\Delta E\|$	0.246437	0.380014	0.690119	0.21465	0.29839	0.303223	0.413604	0.452585	0.245921
ΔE	0.0081182	0.0541363	-0.00699036	-0.0138384	-0.0141052	-0.133988	-0.21881	0.0239864	-0.0295893
E_{comp}/E_{exp}	1.00101	1.01342	0.993656	0.997617	0.997367	0.968581	0.952596	1.00618	0.994041

Table S139: f_{comp} vs f_{exp} PCM/TD-DFT/6-311++G**/xp-gauge/improved fit/ All transitions

	B3P86	CAM-B3LYP	LC-wHPBE	M05	mPW1PW91	O3LYP	SVWN	wB97XD	B3LYP
MAE	0.060518	0.0960912	0.113877	0.0628838	0.0651564	0.0488088	0.0476716	0.100297	0.0595643
Slope	1.05326	1.07534	1.00663	1.06296	1.09391	1.05636	0.992662	1.04278	1.07542
Intercept	0.0180531	0.0230347	0.0203438	0.0141443	0.0105998	0.00796486	0.00252524	0.0348561	0.0113166
R^2	0.905017	0.816449	0.751927	0.895453	0.90102	0.904532	0.903137	0.806857	0.911217
$\|\Delta E\|$	0.24429	0.424533	0.726383	0.238616	0.302398	0.297691	0.400237	0.448701	0.239887
ΔE	0.00614005	0.000266323	-0.065206	-0.0407883	-0.0144657	-0.128887	-0.191489	0.0221432	-0.027858
E_{comp}/E_{exp}	1.00064	0.999594	0.979815	0.99145	0.997736	0.969493	0.959173	1.00571	0.994307

S3.1.2 nf_{exp}

Avg $nf_{exp} = 0.448314$

Table S140: f_{comp} vs nf_{exp} PCM/TD-DFT/6-311++G**/x-gauge/exact band limits/ All transitions

	B3P86	CAM-B3LYP	LC-wHPBE	M05	mPW1PW91	O3LYP	SVWN	wB97XD	B3LYP
MAE	0.0872549	0.111657	0.185675	0.0856068	0.0859785	0.101321	0.131649	0.119948	0.0832897
Slope	0.95622	0.99066	0.757602	0.965398	0.958335	0.90073	0.824476	1.00273	0.950419
Intercept	0.00104712	-0.00127999	0.0185172	0.000725883	0.00385863	0.00803445	0.0225896	-0.00944174	0.0066414
R^2	0.88321	0.832526	0.572799	0.886272	0.884884	0.824548	0.771952	0.816346	0.894078
$\|\Delta E\|$	0.27672	0.577637	1.03818	0.221167	0.335024	0.24893	0.362163	0.572072	0.213816
ΔE	-0.156533	-0.264226	-0.626751	-0.101035	-0.18835	-0.186817	-0.222118	-0.223662	-0.122091
E_{comp}/E_{exp}	0.968097	0.95162	0.874569	0.980235	0.961292	0.960299	0.956838	0.961299	0.974292

Table S141: f_{comp} vs nf_{exp} PCM/TD-DFT/6-311++G**/p-gauge/exact band limits/ All transitions

	B3P86	CAM-B3LYP	LC-wHPBE	M05	mPW1PW91	O3LYP	SVWN	wB97XD	B3LYP
MAE	0.0868591	0.107222	0.181746	0.0852724	0.0846097	0.10155	0.131709	0.115093	0.0830154
Slope	0.929829	0.961344	0.730421	0.969885	0.932689	0.880007	0.806013	0.973064	0.926095
Intercept	0.000490197	-0.000794176	0.0191691	0.000244598	0.00300053	0.00671188	0.0193061	-0.00869285	0.0059838
R^2	0.885825	0.834378	0.579614	0.889596	0.888847	0.83133	0.779125	0.818414	0.89758
$\|\Delta E\|$	0.277263	0.576722	1.03757	0.221314	0.335193	0.249851	0.352443	0.571368	0.21487
ΔE	-0.157505	-0.26564	-0.627582	-0.101922	-0.189368	-0.187105	-0.217427	-0.225486	-0.123404
E_{comp}/E_{exp}	0.967857	0.951299	0.874382	0.980013	0.961038	0.960256	0.956161	0.960903	0.973982

Table S142: f_{comp} vs nf_{exp} PCM/TD-DFT/6-311++G**/xp-gauge/exact band limits/ All transitions

	B3P86	CAM-B3LYP	LC-wHPBE	M05	mPW1PW91	O3LYP	SVWN	wB97XD	B3LYP
MAE	0.086724	0.109094	0.183284	0.0853571	0.0847923	0.101093	0.131677	0.117026	0.0827742
Slope	0.942886	0.975923	0.743899	0.967642	0.94541	0.890273	0.815094	0.987794	0.938157
Intercept	0.00061309	-0.00121431	0.0186964	0.000317955	0.00325714	0.00720946	0.0207745	-0.00924205	0.00614549
R^2	0.884726	0.833716	0.576474	0.888119	0.887101	0.828097	0.775699	0.81764	0.896038
$\|\Delta E\|$	0.277035	0.577181	1.03789	0.221221	0.335145	0.249323	0.352981	0.571778	0.214368
ΔE	-0.157046	-0.265012	-0.627177	-0.101509	-0.188856	-0.187005	-0.217754	-0.224607	-0.122755
E_{comp}/E_{exp}	0.967968	0.951444	0.874473	0.980116	0.961165	0.960269	0.956068	0.961094	0.974133

Improving fit:

Table S143: f_{comp} vs nf_{exp} PCM/TD-DFT/6-311++G**/x-gauge/Improved Fit/ All transitions

	B3P86	CAM-B3LYP	LC-wHPBE	M05	mPW1PW91	O3LYP	SVWN	wB97XD	B3LYP
MAE	0.0437347	0.0614552	0.0849002	0.0423548	0.0458368	0.0409392	0.0526068	0.0649375	0.0385821
Slope	1.00086	1.03787	1.05559	1.02401	1.0175	0.958167	0.936502	1.02386	1.00512
Intercept	-0.00404631	0.00482379	-0.0118491	-0.00782025	-0.00526992	-0.000745198	0.00374492	0.00860618	-0.00716841
R^2	0.96583	0.924294	0.88952	0.966903	0.944915	0.957349	0.916633	0.9166	0.970856
$\|\Delta E\|$	0.21147	0.352766	0.561354	0.209423	0.211392	0.24022	0.339734	0.352533	0.208875
ΔE	0.0140527	0.134611	0.19323	0.028144	0.0567836	-0.108146	-0.187186	0.186092	-0.0232959
E_{comp}/E_{exp}	1.0016	1.02944	1.04131	1.0044	1.01026	0.973402	0.957486	1.04124	0.994046

Table S144: f_{comp} vs nf_{exp} PCM/TD-DFT/6-311++G**/p-gauge/Improved Fit/ All transitions

	B3P86	CAM-B3LYP	LC-wHPBE	M05	mPW1PW91	O3LYP	SVWN	wB97XD	B3LYP
MAE	0.0443241	0.052994	0.0744792	0.0475563	0.0425668	0.0432514	0.0507779	0.0614205	0.038483
Slope	0.984397	1.05278	1.05202	1.01206	0.998188	0.950081	0.943137	1.03128	0.991112
Intercept	-0.00634738	-0.00268333	-0.0111932	-0.011192	-0.00489609	0.00161268	-0.00291987	-0.000267718	-0.00781488
R^2	0.945869	0.957052	0.916441	0.939963	0.946778	0.941202	0.920162	0.924068	0.969082
$\|\Delta E\|$	0.222667	0.307012	0.548824	0.218613	0.241507	0.235863	0.342123	0.347949	0.214465
ΔE	0.026867	0.214355	0.237396	0.0132013	0.0491595	-0.0761464	-0.159712	0.190297	-0.00784101
E_{comp}/E_{exp}	1.00438	1.04688	1.05023	1.00221	1.00802	0.980004	0.96338	1.04181	0.997136

Table S145: f_{comp} vs nf_{exp} PCM/TD-DFT/6-311++G**/xp-gauge/Improved Fit/ All transitions

	B3P86	CAM-B3LYP	LC-wHPBE	M05	mPW1PW91	O3LYP	SVWN	wB97XD	B3LYP
MAE	0.0479675	0.058963	0.0819284	0.0437484	0.0431477	0.0431885	0.0525643	0.0620255	0.0387715
Slope	0.987882	1.02443	1.04464	1.02134	1.0109	0.954884	0.945606	1.0261	0.997836
Intercept	-0.00882933	0.00537453	-0.0107415	-0.00928174	-0.00592759	0.00296171	-0.00241296	0.00620261	-0.00982963
R^2	0.939696	0.927245	0.892855	0.96341	0.946855	0.940654	0.917798	0.922409	0.970031
$\|\Delta E\|$	0.23211	0.304462	0.564972	0.206681	0.243075	0.233981	0.333754	0.324086	0.216965
ΔE	0.02646	0.197297	0.193716	0.0188587	0.0516765	-0.081651	-0.172982	0.218041	-0.0109143
E_{comp}/E_{exp}	1.00507	1.04356	1.0414	1.0029	1.00854	0.978933	0.960612	1.04802	0.997246

S3.2 VHHM ∩ pi-pi*

S3.2.1 f_{exp}

VHHM ∩ pi-pi* : 54 transitions

Avg f_{exp} =

Table S146: f_{comp} vs f_{exp} PCM/TD-DFT/6-311++G**/x-gauge/Exact Band Limits/VH, H, M intercept pi-pi*

	B3P86	CAM-B3LYP	LC-wHPBE	M05	mPW1PW91	O3LYP	SVWN	wB97XD	B3LYP
MAE	0.100927	0.130579	0.163345	0.0939417	0.106518	0.086188	0.121521	0.133123	0.0989935
Slope	1.41176	1.47238	1.41912	1.37708	1.44154	1.37853	1.29782	1.48454	1.40544
Intercept	-0.0137591	-0.00503458	-0.00730115	-0.00922154	-0.0121155	-0.0227919	-0.0252008	-0.00546652	-0.0132537
R^2	0.93003	0.913478	0.759937	0.936268	0.939102	0.925287	0.746156	0.912126	0.933529
$\|\Delta E\|$	0.219741	0.407177	0.789734	0.219445	0.283987	0.261099	0.444581	0.425629	0.225682
ΔE	-0.0464499	-0.0185297	-0.243711	-0.0313157	-0.0677079	-0.166564	-0.232729	0.00220187	-0.0779324
E_{comp}/E_{exp}	0.988352	1.00589	0.954641	0.993672	0.985912	0.959795	0.950238	1.01094	0.981141

Table S147: f_{comp} vs f_{exp} PCM/TD-DFT/6-311++G**/p-gauge/Exact Band Limits/VH, H, M intercept pi-pi*

	B3P86	CAM-B3LYP	LC-wHPBE	M05	mPW1PW91	O3LYP	SVWN	wB97XD	B3LYP
MAE	0.0920602	0.119488	0.151456	0.0945935	0.097238	0.0784231	0.111462	0.122442	0.0905491
Slope	1.33617	1.3877	1.3245	1.34816	1.36378	1.31381	1.24364	1.39865	1.33344
Intercept	-0.00592762	0.00461656	0.00426943	-0.00203404	-0.00433536	-0.0162388	-0.0221759	0.00443095	-0.00580801
R^2	0.922931	0.904713	0.746685	0.929792	0.932935	0.919874	0.748299	0.902155	0.927025
$\|\Delta E\|$	0.221166	0.4052	0.7896	0.219745	0.284176	0.263086	0.502541	0.423705	0.227178
ΔE	-0.0488841	-0.019694	-0.244628	-0.03332	-0.0702013	-0.16732	-0.30681	0.00104939	-0.0805529
E_{comp}/E_{exp}	0.987702	1.00553	0.954419	0.99313	0.985247	0.959596	0.929806	1.01059	0.980447

Table S148: f_{comp} vs f_{exp} PCM/TD-DFT/6-311++G**/xp-gauge/Exact Band Limits/VH, H, M intercept pi-pi*

	B3P86	CAM-B3LYP	LC-wHPBE	M05	mPW1PW91	O3LYP	SVWN	wB97XD	B3LYP
MAE	0.0963824	0.124831	0.157201	0.0941954	0.101756	0.0819361	0.116366	0.127369	0.094662
Slope	1.37359	1.42971	1.37129	1.36269	1.40226	1.3458	1.27036	1.44117	1.36909
Intercept	-0.00994116	-0.00032769	-0.00159729	-0.00577831	-0.00832888	-0.0195992	-0.0238009	-0.000622987	-0.00963525
R^2	0.926775	0.909601	0.75396	0.933281	0.936302	0.922756	0.747419	0.907651	0.930526
$\|\Delta E\|$	0.220536	0.40614	0.789672	0.219517	0.284146	0.261821	0.503573	0.42462	0.226485
ΔE	-0.0477665	-0.0191674	-0.244171	-0.0323745	-0.0689856	-0.16692	-0.30684	0.00159051	-0.0793117
E_{comp}/E_{exp}	0.988	1.00569	0.954529	0.993386	0.985571	0.959702	0.929773	1.01076	0.980775

Improving fit:

Table S149: f_{comp} vs f_{exp} PCM/TD-DFT/6-311++G**/x-gauge/Improved Fit/VH, H, M intercept pi-pi*

	B3P86	CAM-B3LYP	LC-wHPBE	M05	mPW1PW91	O3LYP	SVWN	wB97XD	B3LYP
MAE	0.0856398	0.110029	0.122994	0.0789028	0.0884528	0.0654324	0.0632472	0.112782	0.0802565
Slope	1.36034	1.42623	1.51258	1.31684	1.37517	1.25704	1.12446	1.44749	1.34294
Intercept	-0.0187207	-0.0120157	-0.0364825	-0.013876	-0.0148353	-0.0128876	-0.0158756	-0.0139921	-0.0178506
R^2	0.920952	0.906785	0.887292	0.916003	0.921042	0.909137	0.796462	0.912444	0.922956
$\|\Delta E\|$	0.276528	0.329472	0.666459	0.327466	0.322873	0.318985	0.542248	0.377678	0.26337
ΔE	-0.0119686	0.0992179	0.0426973	-0.0909117	-0.0249699	-0.138959	-0.378448	0.1294	-0.025397
E_{comp}/E_{exp}	0.998606	1.02721	1.00795	0.981211	0.997131	0.967293	0.915088	1.03529	0.995275

Table S150: f_{comp} vs f_{exp} PCM/TD-DFT/6-311++G**/p-gauge/Improved Fit/VH, H, M intercept pi-pi*

	B3P86	CAM-B3LYP	LC-wHPBE	M05	mPW1PW91	O3LYP	SVWN	wB97XD	B3LYP
MAE	0.0754935	0.0973343	0.108806	0.0770102	0.0783213	0.0588954	0.0567213	0.0996602	0.0714713
Slope	1.29473	1.34434	1.41537	1.29186	1.30914	1.19638	1.06734	1.36454	1.28093
Intercept	-0.0141201	-0.00398302	-0.0242824	-0.0114708	-0.0128029	-0.0081198	-0.00872629	-0.00585937	-0.0132523
R^2	0.921641	0.904779	0.887453	0.910895	0.919547	0.905991	0.796647	0.909484	0.923653
$\|\Delta E\|$	0.27714	0.335035	0.58772	0.256783	0.327977	0.316533	0.549621	0.375037	0.26253
ΔE	-0.0336655	0.112434	0.100008	-0.0106371	-0.0343351	-0.14768	-0.371651	0.152162	-0.0264185
E_{comp}/E_{exp}	0.992468	1.03095	1.02418	0.999509	0.99485	0.965321	0.916441	1.04066	0.99492

Table S151: f_{comp} vs f_{exp} PCM/TD-DFT/6-311++G**/xp-gauge/Improved Fit/VH, H, M intercept pi-pi*

	B3P86	CAM-B3LYP	LC-wHPBE	M05	mPW1PW91	O3LYP	SVWN	wB97XD	B3LYP
MAE	0.0808731	0.103473	0.116018	0.0783546	0.0836194	0.061688	0.0600472	0.105682	0.0761694
Slope	1.3314	1.38469	1.45906	1.30459	1.33943	1.22323	1.09708	1.40483	1.31532
Intercept	-0.017585	-0.00796676	-0.0291346	-0.0121346	-0.012992	-0.0105025	-0.0131495	-0.00962151	-0.0164833
R^2	0.923147	0.906063	0.885937	0.914477	0.919809	0.905545	0.796729	0.911645	0.925004
$\|\Delta E\|$	0.277058	0.329646	0.602011	0.329866	0.324421	0.314389	0.542361	0.374627	0.262543
ΔE	-0.0326705	0.0992596	0.114305	-0.0923249	-0.0294442	-0.14548	-0.378943	0.151768	-0.0256053
E_{comp}/E_{exp}	0.992744	1.02722	1.02745	0.980886	0.996057	0.965864	0.914975	1.04057	0.995162

S3.2.2 nf_{exp}

Avg $nf_{exp} = 0.348819$

Table S152: f_{comp} vs nf_{exp} PCM/TD-DFT/6-311++G**/x-gauge/Exact Band Limits

	B3P86	CAM-B3LYP	LC-wHPBE	M05	mPW1PW91	O3LYP	SVWN	wB97XD	B3LYP
MAE	0.0568372	0.0658265	0.108372	0.0544439	0.0531383	0.0629446	0.110604	0.0682302	0.0557106
Slope	0.999221	1.04185	1.00483	0.974405	1.021	0.975645	0.914281	1.05031	0.995142
Intercept	-0.00977729	-0.000784993	-0.00343907	-0.00524519	-0.00829487	-0.018885	-0.0200431	-0.00113301	-0.00942875
R^2	0.915067	0.898302	0.74831	0.920703	0.925266	0.910298	0.727306	0.896733	0.919245
$\|\Delta E\|$	0.219741	0.407177	0.789734	0.219445	0.283987	0.261099	0.444581	0.425629	0.225682
ΔE	-0.0464499	-0.0185297	-0.243711	-0.0313157	-0.0677079	-0.166564	-0.232729	0.00220187	-0.0779324
E_{comp}/E_{exp}	0.988352	1.00589	0.954641	0.993672	0.985912	0.959795	0.950238	1.01094	0.981141

Table S153: f_{comp} vs nf_{exp} PCM/TD-DFT/6-311++G**/p-gauge/Exact Band Limits/

	B3P86	CAM-B3LYP	LC-wHPBE	M05	mPW1PW91	O3LYP	SVWN	wB97XD	B3LYP
MAE	0.0597469	0.0655843	0.108982	0.0570806	0.0544357	0.0650406	0.1099	0.0670576	0.0583943
Slope	0.945507	0.981543	0.937281	0.953715	0.965791	0.929792	0.87622	0.98911	0.944008
Intercept	-0.0020873	0.00875568	0.00806754	0.00193866	-0.000673568	-0.0125	-0.0172722	0.00866426	-0.00212525
R^2	0.907687	0.888987	0.734391	0.913897	0.918932	0.904888	0.72958	0.886157	0.912543
$\|\Delta E\|$	0.221166	0.4052	0.7896	0.219745	0.284176	0.263086	0.502541	0.423705	0.227178
ΔE	-0.0488841	-0.019694	-0.244628	-0.03332	-0.0702013	-0.16732	-0.30681	0.00104939	-0.0805529
E_{comp}/E_{exp}	0.987702	1.00553	0.954419	0.99313	0.985247	0.959596	0.929806	1.01059	0.980447

Table S154: f_{comp} vs nf_{exp} PCM/TD-DFT/6-311++G**/xp-gauge/Exact Band Limits/

	B3P86	CAM-B3LYP	LC-wHPBE	M05	mPW1PW91	O3LYP	SVWN	wB97XD	B3LYP
MAE	0.0580191	0.0646306	0.107758	0.0556628	0.0530831	0.0637446	0.110066	0.0671069	0.0570294
Slope	0.972096	1.01146	0.970679	0.964109	0.993112	0.952457	0.894994	1.01941	0.969326
Intercept	-0.0060304	0.00386557	0.0022323	-0.00180353	-0.00458513	-0.0157724	-0.0187616	0.00365943	-0.00588096
R^2	0.911667	0.89415	0.741996	0.917548	0.922375	0.907767	0.72865	0.891955	0.916135
$\|\Delta E\|$	0.220536	0.40614	0.789672	0.219517	0.284149	0.261806	0.503593	0.42462	0.226485
ΔE	-0.0477665	-0.0191674	-0.244171	-0.0323745	-0.0689881	-0.166906	-0.30686	0.00159051	-0.0793117
E_{comp}/E_{exp}	0.988	1.00569	0.954529	0.993386	0.98557	0.959705	0.92977	1.01076	0.980775

Improving fit:

Table S155: f_{comp} vs nf_{exp} PCM/TD-DFT/6-311++G**/x-gauge/Improved Fit/

	B3P86	CAM-B3LYP	LC-wHPBE	M05	mPW1PW91	O3LYP	SVWN	wB97XD	B3LYP
MAE	0.0437943	0.0487531	0.0676206	0.0425606	0.0430743	0.0401998	0.0495194	0.0508443	0.0424513
Slope	1.02849	1.04615	1.08275	1.02122	1.03527	1.02772	0.946982	1.05875	1.01783
Intercept	-0.0143547	0.00851426	-0.00683407	-0.0124949	-0.0108865	-0.0164499	-0.00643319	0.00837947	-0.0121336
R^2	0.937403	0.940805	0.903446	0.94146	0.939574	0.941937	0.857785	0.94119	0.939797
$\|\Delta E\|$	0.213321	0.260741	0.478013	0.215636	0.218835	0.246008	0.428589	0.289651	0.212789
ΔE	-0.0133234	0.215257	0.265243	-0.000926914	0.045259	-0.118196	-0.281443	0.247937	-0.0477906
E_{comp}/E_{exp}	0.995484	1.05319	1.07003	1.00013	1.01037	0.969389	0.933641	1.0605	0.987621

Table S156: f_{comp} vs nf_{exp} PCM/TD-DFT/6-311++G**/p-gauge/Improved Fit/

	B3P86	CAM-B3LYP	LC-wHPBE	M05	mPW1PW91	O3LYP	SVWN	wB97XD	B3LYP
MAE	0.042818	0.0450765	0.0642917	0.0429698	0.0415331	0.0349213	0.0509272	0.0476769	0.0422313
Slope	0.99625	1.00817	1.02729	1.00695	0.996668	1.00024	0.934353	1.02446	1.00342
Intercept	-0.00842627	0.0123592	0.00174991	-0.00884146	-0.00247929	-0.0109689	-0.00642045	0.00829003	-0.00664507
R^2	0.935197	0.940732	0.905052	0.93787	0.938148	0.942333	0.857763	0.938412	0.937233
$\|\Delta E\|$	0.213388	0.26977	0.497327	0.228913	0.223368	0.255071	0.420538	0.29106	0.220702
ΔE	-0.000456574	0.22916	0.284536	0.0180306	0.0675067	-0.101844	-0.267307	0.249742	-0.0273672
E_{comp}/E_{exp}	0.997854	1.05542	1.07467	1.00535	1.01471	0.973284	0.936871	1.0604	0.991549

Table S157: f_{comp} vs nf_{exp} PCM/TD-DFT/6-311++G**/xp-gauge/Improved Fit/

	B3P86	CAM-B3LYP	LC-wHPBE	M05	mPW1PW91	O3LYP	SVWN	wB97XD	B3LYP
MAE	0.0428128	0.0459135	0.0642839	0.0425376	0.043112	0.037568	0.0512557	0.0489354	0.0416646
Slope	1.00513	1.03468	1.04776	1.00865	1.02205	1.02074	0.932871	1.0296	1.01101
Intercept	-0.0104855	0.00974154	-0.000223213	-0.00788748	-0.00872511	-0.0160788	-0.00731464	0.0113412	-0.00946148
R^2	0.935877	0.943836	0.90383	0.939114	0.939903	0.943127	0.856326	0.937861	0.939554
$\|\Delta E\|$	0.217854	0.27154	0.496269	0.214256	0.227422	0.243966	0.424493	0.279627	0.212553
ΔE	-0.00937138	0.23146	0.283484	-0.000277867	0.0545217	-0.112771	-0.27914	0.231229	-0.0345342
E_{comp}/E_{exp}	0.996064	1.0561	1.0744	1.00029	1.01216	0.970697	0.934409	1.05583	0.989993

S3.2.3 Cf_{exp}

Table S158: f_{comp} vs Cf_{exp} PCM/TD-DFT/6-311++G**/x-gauge/Improved Fit/

	B3P86	CAM-B3LYP	LC-wHPBE	M05	mPW1PW91	O3LYP	SVWN	wB97XD	B3LYP
MAE	0.041448	0.0430666	0.0607543	0.0395278	0.0393314	0.0379711	0.0463159	0.0427809	0.0396933
Slope	1.03217	1.00088	0.980419	1.02279	1.02488	1.03197	1.02195	1.00118	1.02629
Intercept	-0.0185737	-0.000551532	0.0127718	-0.012922	-0.0148176	-0.0177906	-0.0108437	-0.000751844	-0.0150241
R^2	0.947655	0.95546	0.928975	0.947746	0.952439	0.94683	0.857742	0.956963	0.94924
$\|\Delta E\|$	0.22398	0.267112	0.506289	0.223953	0.222758	0.25233	0.472323	0.299495	0.226596
ΔE	-0.0279138	0.221632	0.328926	-0.0154702	0.0479872	-0.145912	-0.306025	0.259151	-0.0616911
E_{comp}/E_{exp}	0.992582	1.05461	1.08297	0.997408	1.01127	0.963705	0.930353	1.06285	0.984892

Table S159: f_{comp} vs Cf_{exp} PCM/TD-DFT/6-311++G**/p-gauge/Improved Fit/

	B3P86	CAM-B3LYP	LC-wHPBE	M05	mPW1PW91	O3LYP	SVWN	wB97XD	B3LYP
MAE	0.0394926	0.042804	0.0632658	0.0387684	0.0386247	0.0357435	0.0429424	0.0440261	0.0367884
Slope	1.01519	0.985002	1.00439	1.0176	1.01188	1.02183	1.02818	0.976422	1.01897
Intercept	-0.00842079	0.00907898	-0.00267201	-0.00986562	-0.00673874	-0.0116886	-0.013173	0.0141435	-0.0103744
R^2	0.942771	0.952484	0.917206	0.944943	0.947045	0.943174	0.859022	0.947137	0.945791
$\|\Delta E\|$	0.21405	0.270222	0.503524	0.239768	0.221769	0.251456	0.47402	0.28223	0.22481
ΔE	-0.0180279	0.222507	0.290732	0.0014354	0.0463552	-0.144886	-0.300837	0.234229	-0.0467488
E_{comp}/E_{exp}	0.99448	1.05338	1.07583	1.00216	1.01069	0.963822	0.930767	1.05664	0.988481

Table S160: f_{comp} vs Cf_{exp} PCM/TD-DFT/6-311++G**/xp-gauge/Improved Fit/

	B3P86	CAM-B3LYP	LC-wHPBE	M05	mPW1PW91	O3LYP	SVWN	wB97XD	B3LYP
MAE	0.0398773	0.0420005	0.0657946	0.0390559	0.038921	0.0365509	0.0439636	0.0428197	0.0377532
Slope	1.02896	0.993755	0.993801	1.01449	1.01777	1.02671	1.0318	0.984648	1.02408
Intercept	-0.0163561	0.00385392	0.00402034	-0.00816371	-0.0102924	-0.0145987	-0.0153245	0.00942355	-0.0134187
R^2	0.946733	0.955691	0.925226	0.94557	0.949548	0.945196	0.860354	0.95115	0.947972
$\|\Delta E\|$	0.247003	0.273598	0.518271	0.222747	0.222056	0.256919	0.485916	0.285455	0.247856
ΔE	0.00133671	0.233908	0.323759	-0.0146979	0.0476455	-0.146491	-0.283618	0.237448	-0.0367251
E_{comp}/E_{exp}	1.00054	1.05682	1.0812	0.997563	1.01103	0.963533	0.935029	1.05734	0.991857

S3.3 VHHM ∩ Mixed

VHHM intercept Mixed: 21 transitions

S3.3.1 f_{exp}

Avg f_{exp} = 0.405048

Table S161: f_{comp} vs f_{exp} PCM/TD-DFT/6-311++G**/x-gauge/Exact Band Limits/

	B3P86	CAM-B3LYP	LC-wHPBE	M05	mPW1PW91	O3LYP	SVWN	wB97XD	B3LYP
MAE	0.125133	0.17509	0.199257	0.127248	0.123848	0.124614	0.111819	0.190105	0.12571
Slope	1.25896	1.30326	1.38309	1.27086	1.23158	1.19073	1.06715	1.37125	1.26685
Intercept	0.0113959	0.0432255	-0.0860306	0.00724021	0.0216569	0.0247329	0.056805	0.030921	0.00854703
R^2	0.977028	0.964288	0.856523	0.974254	0.975951	0.936395	0.891183	0.967325	0.980748
$\|\Delta E\|$	0.157219	0.199275	0.7161	0.156657	0.144376	0.203646	0.238903	0.219813	0.156571
ΔE	-0.0989224	0.183424	-0.281399	-0.0939799	-0.0343315	-0.165981	-0.0873975	0.212997	-0.11641
E_{comp}/E_{exp}	0.978287	1.03982	0.936921	0.980171	0.992174	0.964636	0.983929	1.046	0.974769

Table S162: f_{comp} vs f_{exp} PCM/TD-DFT/6-311++G**/p-gauge/Exact Band Limits/

	B3P86	CAM-B3LYP	LC-wHPBE	M05	mPW1PW91	O3LYP	SVWN	wB97XD	B3LYP
MAE	0.110824	0.160229	0.187129	0.130248	0.109614	0.113843	0.100052	0.174971	0.111938
Slope	1.23562	1.27551	1.35744	1.2883	1.20918	1.17376	1.0509	1.34295	1.24636
Intercept	0.00591384	0.0389259	-0.0876527	0.0026293	0.0158909	0.0191386	0.0489335	0.0265996	0.00255569
R^2	0.978718	0.96772	0.859008	0.975804	0.978509	0.939382	0.902141	0.970497	0.982756
$\|\Delta E\|$	0.157989	0.197125	0.71585	0.157482	0.143224	0.205666	0.241008	0.217911	0.1584
ΔE	-0.100221	0.182036	-0.281291	-0.0948372	-0.0353945	-0.164463	-0.083653	0.211481	-0.117679
E_{comp}/E_{exp}	0.97801	1.03953	0.936957	0.979977	0.991942	0.964937	0.984697	1.04569	0.974496

Table S163: f_{comp} vs f_{exp} PCM/TD-DFT/6-311++G**/xp-gauge/Exact Band Limits/

	B3P86	CAM-B3LYP	LC-wHPBE	M05	mPW1PW91	O3LYP	SVWN	wB97XD	B3LYP
MAE	0.117467	0.167267	0.192743	0.128043	0.116276	0.119067	0.105705	0.182133	0.118381
Slope	1.24744	1.28944	1.37035	1.27966	1.22046	1.18239	1.05909	1.35715	1.25667
Intercept	0.00833635	0.0408298	-0.0870705	0.00471806	0.018495	0.0216473	0.052595	0.0284619	0.00528381
R^2	0.97811	0.96625	0.857906	0.97527	0.977504	0.938083	0.897074	0.969162	0.981961
$\|\Delta E\|$	0.157624	0.198312	0.716002	0.156916	0.143914	0.204734	0.239956	0.218936	0.157542
ΔE	-0.0995254	0.182824	-0.281273	-0.0943984	-0.0347981	-0.165242	-0.085445	0.212299	-0.117021
E_{comp}/E_{exp}	0.978159	1.0397	0.936952	0.980077	0.992072	0.964782	0.984329	1.04586	0.974638

Improving fit:

Table S164: f_{comp} vs f_{exp} PCM/TD-DFT/6-311++G**/x-gauge/Improved Fit/

	B3P86	CAM-B3LYP	LC-wHPBE	M05	mPW1PW91	O3LYP	SVWN	wB97XD	B3LYP
MAE	0.037719	0.0716857	0.103529	0.0344524	0.0393333	0.0302619	0.0164429	0.0980429	0.0452238
Slope	0.989582	0.981925	1.01898	1.00017	1.0006	1.03695	1.03122	1.06898	1.03321
Intercept	0.0306007	0.0248831	0.00880086	0.0178818	0.0268567	0.00879426	-0.0106689	0.020423	0.00846205
R^2	0.977005	0.909474	0.861723	0.973115	0.971111	0.98659	0.995797	0.906482	0.969329
$\|\Delta E\|$	0.199781	0.281561	0.635217	0.150895	0.15916	0.477533	0.292	0.265625	0.220186
ΔE	-0.0816213	0.21199	0.0951674	-0.0477923	0.00122921	-0.22784	-0.0902904	0.199724	-0.155856
E_{comp}/E_{exp}	0.98138	1.04326	1.01785	0.990881	1.00046	0.946519	0.981502	1.04127	0.967436

Table S165: f_{comp} vs f_{exp} PCM/TD-DFT/6-311++G**/p-gauge/Improved Fit/

	B3P86	CAM-B3LYP	LC-wHPBE	M05	mPW1PW91	O3LYP	SVWN	wB97XD	B3LYP
MAE	0.0299524	0.071081	0.0970286	0.0334952	0.0309286	0.023981	0.0120714	0.0920476	0.0354476
Slope	0.970495	0.926168	1.0024	1.01678	1.00189	1.02843	1.02546	1.02766	1.02761
Intercept	0.0261889	0.0665104	0.00504299	0.0113061	0.0181571	0.00349739	-0.0116967	0.0497871	0.00541641
R^2	0.98357	0.929973	0.871693	0.980059	0.981542	0.9922	0.998283	0.925122	0.978195
$\|\Delta E\|$	0.185634	0.247897	0.632075	0.154349	0.16467	0.469512	0.279754	0.272799	0.229042
ΔE	-0.0949147	0.222024	0.0926961	-0.0500256	0.00652542	-0.238631	-0.105602	0.214639	-0.156233
E_{comp}/E_{exp}	0.979345	1.04843	1.01734	0.990396	1.00177	0.944674	0.978691	1.04796	0.967323

Table S166: f_{comp} vs f_{exp} PCM/TD-DFT/6-311++G**/xp-gauge/Improved Fit/

	B3P86	CAM-B3LYP	LC-wHPBE	M05	mPW1PW91	O3LYP	SVWN	wB97XD	B3LYP
MAE	0.0328286	0.0730143	0.098419	0.033381	0.049981	0.0271524	0.013719	0.0940333	0.0362714
Slope	0.984688	0.934475	1.00693	1.0089	1.12725	1.03037	1.03311	1.03106	1.02659
Intercept	0.0259592	0.0691025	0.0113759	0.0135106	-0.0134119	0.00668488	-0.0115576	0.0565045	0.0142993
R^2	0.980455	0.923064	0.868523	0.976631	0.968272	0.989369	0.997671	0.923115	0.979401
$\|\Delta E\|$	0.201857	0.247377	0.627481	0.153419	0.159314	0.474459	0.283176	0.25482	0.201546
ΔE	-0.0830782	0.221772	0.123841	-0.0509091	0.00711965	-0.221305	-0.0820123	0.241271	-0.131282
E_{comp}/E_{exp}	0.981033	1.04839	1.0228	0.990239	1.00146	0.947761	0.982828	1.05259	0.971731

S3.3.2 nf_{exp}

Avg $nf_{exp} = 0.582223$

Table S167: f_{comp} vs nf_{exp} PCM/TD-DFT/6-311++G**/x-gauge/Exact Band Limits/

	B3P86	CAM-B3LYP	LC-wHPBE	M05	mPW1PW91	O3LYP	SVWN	wB97XD	B3LYP
MAE	0.0879214	0.0840738	0.15048	0.0887214	0.0905119	0.12066	0.155724	0.0779643	0.0853833
Slope	0.87067	0.899526	0.959799	0.879223	0.850141	0.822372	0.736718	0.94835	0.875963
Intercept	0.014409	0.0473848	-0.0846316	0.0100959	0.0255327	0.0282289	0.060118	0.0341913	0.011675
R^2	0.975031	0.958506	0.860649	0.972968	0.970313	0.931963	0.886222	0.965388	0.978375
$\|\Delta E\|$	0.157219	0.199275	0.7161	0.156657	0.144376	0.203646	0.238903	0.219813	0.156571
ΔE	-0.0989224	0.183424	-0.281399	-0.0939799	-0.0343315	-0.165981	-0.0873975	0.212997	-0.11641
E_{comp}/E_{exp}	0.978287	1.03982	0.936921	0.980171	0.992174	0.964636	0.983929	1.046	0.974769

Table S168: f_{comp} vs nf_{exp} PCM/TD-DFT/6-311++G**/p-gauge/Exact Band Limits/

	B3P86	CAM-B3LYP	LC-wHPBE	M05	mPW1PW91	O3LYP	SVWN	wB97XD	B3LYP
MAE	0.0921881	0.0803862	0.153528	0.0841167	0.0956338	0.123114	0.158281	0.0743948	0.0890348
Slope	0.854932	0.880733	0.942439	0.891656	0.835053	0.810978	0.725879	0.929182	0.8622
Intercept	0.00863887	0.0427831	-0.0865336	0.00530955	0.0194794	0.0223964	0.0519763	0.0295658	0.00539741
R^2	0.977629	0.962718	0.863945	0.975324	0.973724	0.935683	0.898053	0.969402	0.981299
$\|\Delta E\|$	0.157989	0.197125	0.71585	0.157482	0.143224	0.205666	0.241008	0.217911	0.1584
ΔE	-0.100221	0.182036	-0.281291	-0.0948372	-0.0353945	-0.164463	-0.083653	0.211481	-0.117679
E_{comp}/E_{exp}	0.97801	1.03953	0.936957	0.979977	0.991942	0.964937	0.984697	1.04569	0.974496

Table S169: f_{comp} vs nf_{exp} PCM/TD-DFT/6-311++G**/xp-gauge/Exact Band Limits/

	B3P86	CAM-B3LYP	LC-wHPBE	M05	mPW1PW91	O3LYP	SVWN	wB97XD	B3LYP
MAE	0.0901119	0.0821071	0.15209	0.08645	0.0925024	0.120815	0.157	0.076049	0.0872357
Slope	0.86291	0.890172	0.95118	0.885496	0.842654	0.816781	0.731342	0.938803	0.869133
Intercept	0.0112033	0.0448356	-0.0858137	0.00748634	0.0222255	0.0250225	0.0557716	0.0315782	0.00826571
R^2	0.976571	0.960861	0.862441	0.974389	0.972294	0.934022	0.892548	0.967647	0.980053
$\|\Delta E\|$	0.157624	0.198312	0.716002	0.156916	0.143914	0.204734	0.239956	0.218936	0.157542
ΔE	-0.0995254	0.182824	-0.281273	-0.0943984	-0.0347981	-0.165242	-0.085445	0.212299	-0.117021
E_{comp}/E_{exp}	0.978159	1.0397	0.936952	0.980077	0.992072	0.964782	0.984329	1.04586	0.974638

Improving fit:

Table S170: f_{comp} vs nf_{exp} PCM/TD-DFT/6-311++G**/x-gauge/Improved Fit/

	B3P86	CAM-B3LYP	LC-wHPBE	M05	mPW1PW91	O3LYP	SVWN	wB97XD	B3LYP
MAE	0.0379252	0.03047	0.0827424	0.0369214	0.03029	0.072991	0.0564481	0.0332138	0.0367671
Slope	0.956444	1.00126	1.03969	0.981259	0.987125	0.853899	0.891815	1.0009	0.951366
Intercept	-0.00197375	0.0008424	-0.0658531	-0.0126787	-0.00883204	0.0210355	0.0315928	0.00765069	0.00132123
R^2	0.989224	0.989997	0.918007	0.991661	0.993504	0.975264	0.961556	0.988601	0.992238
$\|\Delta E\|$	0.157528	0.24801	0.463161	0.152337	0.162587	0.180385	0.282156	0.254377	0.155506
ΔE	-0.0339099	0.224308	0.412007	-0.0267662	0.0401677	-0.151234	-0.142612	0.241994	-0.0556311
E_{comp}/E_{exp}	0.99068	1.04745	1.08383	0.992952	1.00651	0.967343	0.971027	1.05155	0.986429

Table S171: f_{comp} vs nf_{exp} PCM/TD-DFT/6-311++G**/p-gauge/Improved Fit/

	B3P86	CAM-B3LYP	LC-wHPBE	M05	mPW1PW91	O3LYP	SVWN	wB97XD	B3LYP
MAE	0.04393	0.0271433	0.0817348	0.0336052	0.0341776	0.07945	0.0556824	0.0296748	0.0419043
Slope	1.05057	1.00039	1.02652	0.977211	0.97403	0.836559	0.875642	0.993203	0.937261
Intercept	-0.0455248	-0.00524317	-0.0670448	-0.0126501	-0.00818421	0.0264308	0.0386853	0.00326266	0.00112846
R^2	0.983491	0.99282	0.919097	0.994255	0.993677	0.973558	0.960857	0.991739	0.99036
$\|\Delta E\|$	0.190755	0.262199	0.682167	0.149043	0.158554	0.1949	0.270442	0.273929	0.150936
ΔE	0.00524469	0.239186	0.196477	-0.0194603	0.0521812	-0.105624	-0.119621	0.262237	-0.0495172
E_{comp}/E_{exp}	0.997761	1.05006	1.03509	0.994583	1.00902	0.97608	0.976296	1.05529	0.987956

Table S172: f_{comp} vs nf_{exp} PCM/TD-DFT/6-311++G**/xp-gauge/Improved Fit/

	B3P86	CAM-B3LYP	LC-wHPBE	M05	mPW1PW91	O3LYP	SVWN	wB97XD	B3LYP
MAE	0.0395471	0.0288148	0.0823748	0.0364119	0.03007	0.0845119	0.0569271	0.0307395	0.0394443
Slope	0.946606	1.00152	1.03069	0.974031	0.97697	0.861348	0.883941	1.00445	0.942915
Intercept	-0.00152678	6.89607e-05	-0.0682896	-0.0114701	-0.00534312	0.00727911	0.0344253	-0.0001499	0.000188885
R^2	0.989392	0.991606	0.919045	0.993471	0.994195	0.967709	0.961387	0.990533	0.992107
$\|\Delta E\|$	0.1733	0.261946	0.49679	0.147569	0.149774	0.215531	0.273655	0.252219	0.153492
ΔE	-0.017618	0.238537	0.445636	-0.0204298	0.060212	-0.141851	-0.133891	0.240145	-0.0530004
E_{comp}/E_{exp}	0.993774	1.04996	1.09067	0.994415	1.01097	0.96921	0.972895	1.05121	0.986981

S3.4 VHHM ∩ pi-pi* (CT)

VHHM ∩ pi-pi* (CT): 7 transitions. Due to small sample size, this led to large intercepts and poor R^2.

S3.4.1 f_{exp}

Avg f_{exp} = 0.543771

Table S173: f_{comp} vs f_{exp} PCM/TD-DFT/6-311++G**/x-gauge/Exact Band Limits/

	B3P86	CAM-B3LYP	LC-wHPBE	M05	mPW1PW91	O3LYP	SVWN	wB97XD	B3LYP
MAE	0.333729	0.4049	0.412186	0.327129	0.339643	0.352529	0.2608	0.427329	0.329771
Slope	1.24492	2.05188	0.500721	1.17631	1.15468	1.97772	-0.136324	2.24671	1.24412
Intercept	0.124762	-0.291241	0.488236	0.150756	0.172119	-0.256654	0.703615	-0.377357	0.124524
R^2	0.0896081	0.175225	0.00875713	0.0781326	0.0714278	0.215006	0.00165317	0.194574	0.0909935
$\|\Delta E\|$	0.188529	0.148736	0.467514	0.182228	0.162787	0.228078	0.319857	0.206567	0.204634
ΔE	-0.132022	0.142571	0.467514	-0.125531	-0.0830979	-0.185952	-0.0237013	0.206567	-0.15648
E_{comp}/E_{exp}	0.961429	1.04108	1.1321	0.96403	0.97506	0.946835	1.00519	1.06044	0.954245

Table S174: f_{comp} vs f_{exp} PCM/TD-DFT/6-311++G**/p-gauge/Exact Band Limits/

	B3P86	CAM-B3LYP	LC-wHPBE	M05	mPW1PW91	O3LYP	SVWN	wB97XD	B3LYP
MAE	0.301871	0.369443	0.382386	0.319057	0.306757	0.323429	0.244886	0.391414	0.299829
Slope	1.23644	2.02928	0.526266	1.19378	1.14756	1.9597	-0.0349255	2.2191	1.23697
Intercept	0.0962303	-0.314634	0.43966	0.13367	0.141588	-0.276699	0.627049	-0.398281	0.0970718
R^2	0.0983314	0.189753	0.010784	0.0839036	0.0783939	0.231576	0.000118314	0.209345	0.099258
$\|\Delta E\|$	0.186678	0.147826	0.463362	0.180498	0.163562	0.224286	0.31641	0.205051	0.202385
ΔE	-0.127947	0.141841	0.463362	-0.123871	-0.0808098	-0.180414	-0.0180611	0.205051	-0.152052
E_{comp}/E_{exp}	0.96255	1.0409	1.1311	0.964494	0.97569	0.948411	1.00673	1.06007	0.955442

Table S175: f_{comp} vs f_{exp} PCM/TD-DFT/6-311++G**/xp-gauge/Exact Band Limits/

	B3P86	CAM-B3LYP	LC-wHPBE	M05	mPW1PW91	O3LYP	SVWN	wB97XD	B3LYP
MAE	0.317529	0.386929	0.3971	0.323014	0.322986	0.337714	0.252714	0.409114	0.314557
Slope	1.24236	2.04089	0.514025	1.18693	1.15296	1.97053	-0.083225	2.23353	1.24198
Intercept	0.109184	-0.303376	0.463374	0.141008	0.155508	-0.268018	0.663713	-0.38843	0.109648
R^2	0.0941027	0.182444	0.00974345	0.0811985	0.0750556	0.223532	0.000643336	0.201977	0.0952241
$\|\Delta E\|$	0.187648	0.148255	0.465553	0.181444	0.163137	0.226289	0.318139	0.20581	0.203617
ΔE	-0.130069	0.142177	0.465553	-0.12473	-0.0819772	-0.183293	-0.0209322	0.20581	-0.154297
E_{comp}/E_{exp}	0.961966	1.04098	1.13163	0.964254	0.975369	0.947591	1.00594	1.06026	0.954833

Improving fit:

Table S176: f_{comp} vs f_{exp} PCM/TD-DFT/6-311++G**/x-gauge/Improved Fit/

	B3P86	CAM-B3LYP	LC-wHPBE	M05	mPW1PW91	O3LYP	SVWN	wB97XD	B3LYP
MAE	0.0449857	0.199857	0.254629	0.175414	0.0634857	0.0785571	0.0616857	0.197943	0.0403571
Slope	0.917399	-0.256019	-1.2961	-0.206691	1.09825	1.12773	0.570469	0.381912	0.758182
Intercept	0.0808306	0.679944	1.23481	0.645664	-0.0186552	-0.0136689	0.217395	0.339599	0.164579
R^2	0.4516	0.00574759	0.128008	0.00427202	0.433798	0.338607	0.204791	0.0116198	0.392268
$\|\Delta E\|$	0.566699	0.71199	0.931289	0.629968	0.62008	0.405287	0.429143	0.768831	0.590346
ΔE	0.310982	-0.0447036	0.174595	-0.340372	0.458192	-0.140961	0.0958164	0.0121371	0.3028
E_{comp}/E_{exp}	1.08487	0.94161	1.00043	0.861093	1.12841	0.944297	1.02889	0.957518	1.08115

Table S177: f_{comp} vs f_{exp} PCM/TD-DFT/6-311++G**/p-gauge/Improved Fit/

	B3P86	CAM-B3LYP	LC-wHPBE	M05	mPW1PW91	O3LYP	SVWN	wB97XD	B3LYP
MAE	0.0362143	0.1877	0.238043	0.166786	0.0674143	0.1363	0.0372429	0.182671	0.0339714
Slope	0.857284	-0.116093	-1.19137	0.640079	0.136712	1.46587	0.823701	0.414246	0.711301
Intercept	0.0967622	0.5859	1.15636	0.340572	0.52476	-0.149413	0.0849233	0.300688	0.173586
R^2	0.491916	0.00128367	0.120928	0.0583604	0.026192	0.16295	0.484197	0.0149618	0.435195
$\|\Delta E\|$	0.568867	0.341026	0.930872	0.286718	0.466089	0.36241	0.468258	0.767062	0.591392
ΔE	0.313262	0.341026	0.174178	0.00140758	0.294514	-0.0928619	0.153956	0.0103683	0.303923
E_{comp}/E_{exp}	1.08567	1.08729	1.00033	0.990234	1.09059	0.962213	1.04563	0.957091	1.08165

Table S178: f_{comp} vs f_{exp} PCM/TD-DFT/6-311++G**/xp-gauge/Improved Fit/

	B3P86	CAM-B3LYP	LC-wHPBE	M05	mPW1PW91	O3LYP	SVWN	wB97XD	B3LYP
MAE	0.0382286	0.194571	0.2463	0.171786	0.0568429	0.141271	0.0494429	0.190271	0.0346143
Slope	0.888885	-0.199481	-1.2423	0.615542	1.06978	1.41932	0.698519	0.399614	0.737072
Intercept	0.0877925	0.639201	1.1946	0.358372	-0.0121021	-0.117327	0.149508	0.319073	0.16773
R^2	0.472236	0.00364848	0.124336	0.0516156	0.451698	0.142956	0.277581	0.0133074	0.414573
$\|\Delta E\|$	0.568273	0.711767	0.931184	0.287179	0.620066	0.367183	0.4524	0.767946	0.591395
ΔE	0.312556	-0.0449263	0.17449	0.00186866	0.458178	-0.100957	0.13253	0.0112526	0.303849
E_{comp}/E_{exp}	1.08542	0.941589	1.0004	0.99035	1.12847	0.959865	1.03962	0.957304	1.08156

S3.4.2 nf_{exp}

Avg $nf_{exp} = 0.751171$

Table S179: f_{comp} vs nf_{exp} PCM/TD-DFT/6-311++G**/x-gauge/Exact Band Limits/

	B3P86	CAM-B3LYP	LC-wHPBE	M05	mPW1PW91	O3LYP	SVWN	wB97XD	B3LYP
MAE	0.217029	0.255303	0.325686	0.218657	0.234343	0.20796	0.250557	0.272531	0.2201
Slope	1.19336	1.90009	0.543505	1.15614	1.17069	1.80423	0.169297	2.06415	1.19914
Intercept	-0.0947024	-0.602775	0.352249	-0.078056	-0.0793881	-0.536513	0.502314	-0.706189	-0.0997165
R^2	0.129411	0.236157	0.0162159	0.118623	0.115397	0.281235	0.00400719	0.258129	0.132858
$\|\Delta E\|$	0.188529	0.148736	0.467514	0.182228	0.162787	0.228078	0.319857	0.206567	0.204634
ΔE	-0.132022	0.142571	0.467514	-0.125531	-0.0830979	-0.185952	-0.0237013	0.206567	-0.15648
E_{comp}/E_{exp}	0.961429	1.04108	1.1321	0.96403	0.97506	0.946835	1.00519	1.06044	0.954245

Table S180: f_{comp} vs nf_{exp} PCM/TD-DFT/6-311++G**/p-gauge/Exact Band Limits/

	B3P86	CAM-B3LYP	LC-wHPBE	M05	mPW1PW91	O3LYP	SVWN	wB97XD	B3LYP
MAE	0.203011	0.227989	0.295886	0.2139	0.218971	0.185831	0.242957	0.244817	0.20874
Slope	1.18144	1.87325	0.558135	1.17356	1.15998	1.78348	0.235052	2.03253	1.1892
Intercept	-0.118893	-0.618301	0.306574	-0.0987282	-0.105747	-0.550774	0.431492	-0.718378	-0.12359
R^2	0.141102	0.254133	0.019064	0.127439	0.125892	0.301451	0.00842254	0.276025	0.144185
$\|\Delta E\|$	0.186678	0.147826	0.463362	0.180498	0.163562	0.224286	0.31641	0.205051	0.202385
ΔE	-0.127947	0.141841	0.463362	-0.123871	-0.0808098	-0.180414	-0.0180611	0.205051	-0.152052
E_{comp}/E_{exp}	0.96255	1.0409	1.1311	0.964494	0.97569	0.948411	1.00673	1.06007	0.955442

Table S181: f_{comp} vs nf_{exp} PCM/TD-DFT/6-311++G**/xp-gauge/Exact Band Limits/

	B3P86	CAM-B3LYP	LC-wHPBE	M05	mPW1PW91	O3LYP	SVWN	wB97XD	B3LYP
MAE	0.209229	0.241417	0.3106	0.216229	0.226514	0.19666	0.246729	0.25846	0.212886
Slope	1.1886	1.88678	0.55126	1.16629	1.16673	1.7952	0.203956	2.04873	1.19526
Intercept	-0.108101	-0.610891	0.328795	-0.0896549	-0.0939536	-0.545006	0.465251	-0.712844	-0.112849
R^2	0.135377	0.245073	0.0176125	0.123217	0.120796	0.291585	0.00607251	0.267085	0.138615
$\|\Delta E\|$	0.187648	0.148255	0.465553	0.181444	0.163137	0.226289	0.318139	0.20581	0.203617
ΔE	-0.130069	0.142177	0.465553	-0.12473	-0.0819772	-0.183293	-0.0209322	0.20581	-0.154297
E_{comp}/E_{exp}	0.961966	1.04098	1.13163	0.964254	0.975369	0.947591	1.00594	1.06026	0.954833

Improving fit:

Table S182: f_{comp} vs nf_{exp} PCM/TD-DFT/6-311++G**/x-gauge/Improved Fit/

	B3P86	CAM-B3LYP	LC-wHPBE	M05	mPW1PW91	O3LYP	SVWN	wB97XD	B3LYP
MAE	0.0397886	0.145443	0.174029	0.0852857	0.0722286	0.0609257	0.0516886	0.168597	0.0296171
Slope	1.16059	1.58941	0.748807	1.0047	0.908798	1.2163	1.33543	1.30544	1.02827
Intercept	-0.131589	-0.31468	0.335889	0.0469827	0.125651	-0.180738	-0.282852	-0.136766	-0.0335064
R^2	0.822018	0.37007	0.0944181	0.309342	0.334013	0.646157	0.857447	0.210586	0.891909
$\|\Delta E\|$	0.496412	0.368287	0.706412	0.389236	0.341474	0.418525	0.365617	0.544005	0.515308
ΔE	0.242913	0.359587	0.706412	0.030134	0.0888377	-0.17444	-0.0908283	0.544005	0.203736
E_{comp}/E_{exp}	1.08327	1.09351	1.19247	0.999088	1.0148	0.942113	0.973905	1.14303	1.071

Table S183: f_{comp} vs nf_{exp} PCM/TD-DFT/6-311++G**/p-gauge/Improved Fit/

	B3P86	CAM-B3LYP	LC-wHPBE	M05	mPW1PW91	O3LYP	SVWN	wB97XD	B3LYP
MAE	0.0449686	0.126057	0.151157	0.0576771	0.0603314	0.120637	0.0511114	0.158174	0.0390171
Slope	0.963304	1.57904	1.40431	1.09703	0.893742	1.47953	1.22199	1.31519	0.862761
Intercept	0.00365104	-0.338532	-0.15255	-0.0161704	0.104375	-0.338396	-0.215035	-0.102376	0.0783759
R^2	0.724858	0.419345	0.368984	0.433379	0.379815	0.325903	0.797967	0.31309	0.826101
$\|\Delta E\|$	0.486049	0.376585	0.68433	0.36512	0.340965	0.359989	0.409602	0.527598	0.500802
ΔE	0.257868	0.367909	0.68433	0.0697101	0.0919022	-0.113363	-0.125628	0.527598	0.21939
E_{comp}/E_{exp}	1.08699	1.09599	1.18573	1.0103	1.01562	0.963995	0.965808	1.13791	1.07485

Table S184: f_{comp} vs nf_{exp} PCM/TD-DFT/6-311++G**/xp-gauge/Improved Fit/

	B3P86	CAM-B3LYP	LC-wHPBE	M05	mPW1PW91	O3LYP	SVWN	wB97XD	B3LYP
MAE	0.0351143	0.1347	0.170657	0.0630371	0.0623629	0.125371	0.0513914	0.1663	0.02604
Slope	1.14858	1.59318	1.42649	1.07755	0.910218	1.47837	1.22025	1.95382	1.02281
Intercept	-0.13488	-0.331424	-0.149709	0.00260453	0.10747	-0.325765	-0.202873	-0.602015	-0.0398914
R^2	0.840618	0.401193	0.353023	0.406873	0.360384	0.305223	0.806263	0.46682	0.917252
$\|\Delta E\|$	0.496966	0.376746	0.686101	0.365329	0.341435	0.366316	0.41529	0.524982	0.512507
ΔE	0.244609	0.368055	0.686101	0.0684647	0.0902583	-0.120798	-0.13748	0.524982	0.207225
E_{comp}/E_{exp}	1.08375	1.096	1.18615	1.00996	1.01518	0.961844	0.963212	1.13729	1.07195

S3.5 VH

S3.5.1 f_{exp}

Avg f_{exp} = 0.277068.

Table S185: f_{comp} vs f_{exp} PCM/TD-DFT/6-311++G**/x-gauge/Experimental Band Limits/ VH Transitions

	B3P86	CAM-B3LYP	LC-wHPBE	M05	mPW1PW91	O3LYP	SVWN	wB97XD	B3LYP
MAE	0.110239	0.136561	0.149668	0.104432	0.118482	0.0929321	0.108232	0.139296	0.104975
Slope	1.38467	1.38239	1.41862	1.315	1.39111	1.38263	1.47142	1.38964	1.36437
Intercept	0.00365916	0.0306125	0.0220455	0.0171553	0.0101178	-0.0130821	-0.025783	0.0313399	0.00401969
R^2	0.988178	0.988142	0.970726	0.985553	0.988098	0.986863	0.97511	0.987687	0.986635

Table S186: f_{comp} vs f_{exp} PCM/TD-DFT/6-311++G**/p-gauge/Experimental Band Limits/ VH Transitions

	B3P86	CAM-B3LYP	LC-wHPBE	M05	mPW1PW91	O3LYP	SVWN	wB97XD	B3LYP
MAE	0.0945107	0.119861	0.130082	0.101689	0.102239	0.0796036	0.092625	0.122375	0.090375
Slope	1.29813	1.28766	1.31393	1.276	1.30298	1.30782	1.39356	1.29388	1.28243
Intercept	0.0119097	0.0401596	0.0314671	0.0252178	0.0182919	-0.00568382	-0.0184457	0.0409506	0.0121223
R^2	0.987385	0.987383	0.967357	0.984732	0.987285	0.985965	0.971627	0.986892	0.985605

Table S187: f_{comp} vs f_{exp} PCM/TD-DFT/6-311++G**/xp-gauge/Experimental Band Limits/ VH Transitions

	B3P86	CAM-B3LYP	LC-wHPBE	M05	mPW1PW91	O3LYP	SVWN	wB97XD	B3LYP
MAE	0.102211	0.128025	0.139632	0.102946	0.110175	0.0861107	0.100196	0.130618	0.0974964
Slope	1.34089	1.33444	1.36551	1.29549	1.34654	1.3447	1.43197	1.34113	1.32302
Intercept	0.0077608	0.0353632	0.026724	0.021075	0.0141603	-0.00939437	-0.0222185	0.036103	0.00799764
R^2	0.987879	0.987907	0.96935	0.985215	0.987786	0.986476	0.973499	0.98745	0.986184

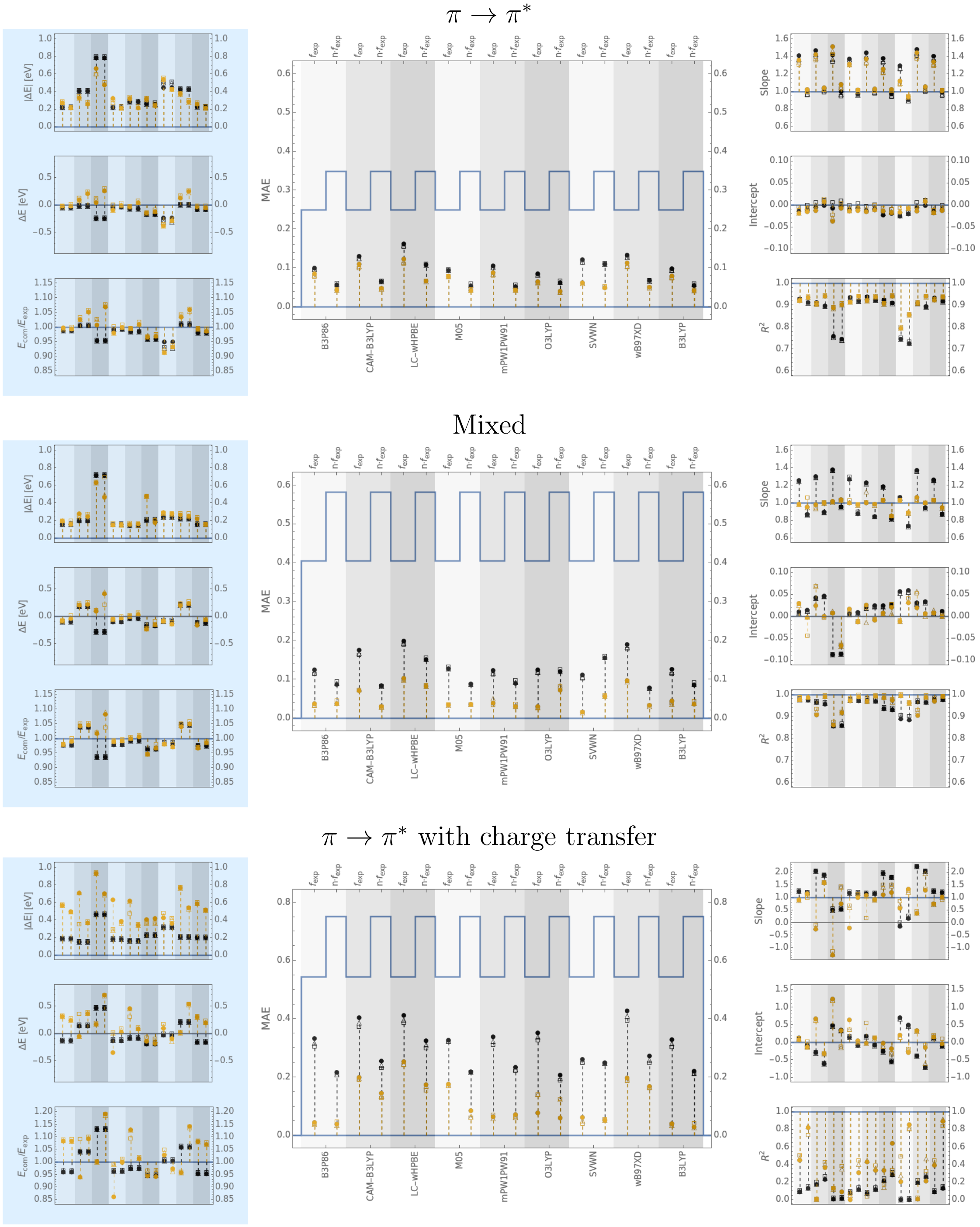

Figure S3: Separating the VHHM transitions into three subsets ($\pi \rightarrow \pi^*$, Mixed, and $\pi \rightarrow \pi^*$ with charge transfer) according to the character of the orbitals involved

Maybe the improved fit for f_{comp} vs f_{exp} doesn't makes sense since the original slope is about 1.3 with R^2 mainly above.98, but here it is:

Table S188: f_{comp} vs f_{exp} PCM/TD-DFT/6-311++G**/xp-gauge/Improved Fit/ VH Transitions

	B3P86	CAM-B3LYP	LC-wHPBE	M05	mPW1PW91	O3LYP	SVWN	wB97XD	B3LYP
MAE	0.0999179	0.128211	0.136489	0.092875	0.109739	0.066275	0.0510464	0.130175	0.096975
Slope	1.34437	1.40354	1.44029	1.28386	1.36088	1.20361	1.25953	1.40973	1.33604
Intercept	0.00450312	0.00683025	-0.00468042	0.0142278	0.00975106	0.0042903	-0.0245546	0.00706543	0.00386896
R^2	0.989274	0.977856	0.961887	0.985017	0.988584	0.955104	0.981011	0.976279	0.988863
$\|\Delta E\|$	0.225548	0.304448	0.433111	0.247603	0.257541	0.213588	0.315318	0.313619	0.210567
ΔE	0.0939251	0.286054	0.433111	0.100289	0.157104	-0.0221623	-0.215437	0.306197	0.0601536
E_{comp}/E_{exp}	1.02242	1.06952	1.10485	1.02527	1.03771	0.994471	0.947881	1.07431	1.01446

Table S189: f_{comp} vs f_{exp} PCM/TD-DFT/6-311++G**/p-gauge/Improved Fit/ VH Transitions

	B3P86	CAM-B3LYP	LC-wHPBE	M05	mPW1PW91	O3LYP	SVWN	wB97XD	B3LYP
MAE	0.0836821	0.110561	0.115946	0.0892607	0.0929036	0.0570607	0.0404821	0.112332	0.0817321
Slope	1.25719	1.30939	1.33827	1.24342	1.27173	1.13328	1.18101	1.3148	1.25267
Intercept	0.0124231	0.0152953	0.00310046	0.021816	0.0176155	0.010613	-0.0165406	0.0155529	0.0117258
R^2	0.989209	0.976192	0.959571	0.98459	0.988388	0.954184	0.980614	0.97459	0.988618
$\|\Delta E\|$	0.225548	0.304448	0.433111	0.247603	0.257541	0.213587	0.315597	0.313619	0.210567
ΔE	0.0939251	0.286054	0.433111	0.100289	0.157104	-0.0221622	-0.215715	0.306197	0.0601536
E_{comp}/E_{exp}	1.02242	1.06952	1.10485	1.02527	1.03771	0.994471	0.947826	1.07431	1.01446

Table S190: f_{comp} vs f_{exp} PCM/TD-DFT/6-311++G**/xp-gauge/Improved Fit/ VH Transitions

	B3P86	CAM-B3LYP	LC-wHPBE	M05	mPW1PW91	O3LYP	SVWN	wB97XD	B3LYP
MAE	0.0916607	0.119246	0.126018	0.0910036	0.101168	0.0615179	0.0455179	0.121068	0.089225
Slope	1.30031	1.35593	1.38852	1.26365	1.31585	1.16797	1.21984	1.36166	1.29388
Intercept	0.00845488	0.0110569	-0.000778198	0.0179534	0.0136568	0.00746385	-0.0205634	0.0112922	0.00779927
R^2	0.989318	0.977156	0.960972	0.984873	0.988569	0.954646	0.980873	0.975603	0.988794
$\|\Delta E\|$	0.225548	0.304448	0.433111	0.247603	0.257541	0.213579	0.31546	0.313619	0.210567
ΔE	0.0939251	0.286054	0.433111	0.100289	0.157104	-0.0221535	-0.215578	0.306197	0.0601536
E_{comp}/E_{exp}	1.02242	1.06952	1.10485	1.02527	1.03771	0.994473	0.947853	1.07431	1.01446

S3.5.2 nf_{exp}

Avg $nf_{exp} = 0.383316$

Table S191: f_{comp} vs nf_{exp} PCM/TD-DFT/6-311++G**/x-gauge/Experimental Band Limits/ VH Transitions

	B3P86	CAM-B3LYP	LC-wHPBE	M05	mPW1PW91	O3LYP	SVWN	wB97XD	B3LYP
MAE	0.0274164	0.0421721	0.0584379	0.0314293	0.0306279	0.0282707	0.0457679	0.0439221	0.0268793
Slope	1.00289	1.0009	1.02665	0.952365	1.00748	1.00186	1.06543	1.00612	0.995906
Intercept	0.00288462	0.0299653	0.0215688	0.0164428	0.00936711	-0.0140301	-0.0264958	0.0307026	0.00221733
R^2	0.985629	0.984945	0.966664	0.982886	0.985409	0.985212	0.972069	0.984423	0.985398
$\|\Delta E\|$	0.225281	0.298495	0.732789	0.250147	0.256987	0.214786	0.253911	0.30804	0.213229
ΔE	0.0923963	0.279749	0.0834121	0.0997158	0.155404	-0.0186461	-0.13636	0.301505	0.0587922
E_{comp}/E_{exp}	1.02214	1.06795	1.02864	1.0252	1.03738	0.99538	0.966188	1.07317	1.01422

Table S192: f_{comp} vs nf_{exp} PCM/TD-DFT/6-311++G**/p-gauge/Experimental Band Limits/ VH Transitions

	B3P86	CAM-B3LYP	LC-wHPBE	M05	mPW1PW91	O3LYP	SVWN	wB97XD	B3LYP
MAE	0.0307393	0.0403807	0.0550607	0.0342093	0.0304764	0.0315879	0.0466421	0.0408093	0.031345
Slope	0.940265	0.932366	0.950899	0.924178	0.943723	0.947734	1.00894	0.936837	0.936067
Intercept	0.0111594	0.0395374	0.031019	0.0245046	0.0175626	-0.00661068	-0.0190807	0.040338	0.0104471
R^2	0.98497	0.984295	0.963344	0.982188	0.98474	0.98448	0.968397	0.983737	0.984666
$\|\Delta E\|$	0.225182	0.29818	0.732461	0.25079	0.256864	0.215365	0.253649	0.30786	0.213853
ΔE	0.0918806	0.279384	0.0830848	0.0992941	0.154949	-0.0186307	-0.13484	0.301105	0.0583499
E_{comp}/E_{exp}	1.02203	1.06787	1.02856	1.0251	1.03728	0.995395	0.96667	1.07308	1.01412

Table S193: f_{comp} vs nf_{exp} PCM/TD-DFT/6-311++G**/xp-gauge/Experimental Band Limits/ VH Transitions

	B3P86	CAM-B3LYP	LC-wHPBE	M05	mPW1PW91	O3LYP	SVWN	wB97XD	B3LYP
MAE	0.0283536	0.039825	0.0543336	0.032595	0.0284864	0.0282264	0.0455279	0.041475	0.0289893
Slope	0.971207	0.96621	0.988221	0.938261	0.975228	0.974416	1.03682	0.971017	0.965626
Intercept	0.00699896	0.0347286	0.0262628	0.0203634	0.0134219	-0.0103309	-0.0228927	0.035479	0.00631695
R^2	0.985392	0.984763	0.965306	0.982602	0.985159	0.984903	0.970368	0.984235	0.985088
$\|\Delta E\|$	0.22524	0.298327	0.732621	0.250436	0.25694	0.215073	0.253713	0.307946	0.21352
ΔE	0.0921485	0.279557	0.0832447	0.0995208	0.155205	-0.018666	-0.135744	0.301326	0.058578
E_{comp}/E_{exp}	1.02209	1.06791	1.0286	1.02515	1.03734	0.995381	0.966394	1.07313	1.01417

Improving fit

Table S194: f_{comp} vs nf_{exp} PCM/TD-DFT/6-311++G**/x-gauge/Improved Fit/ VH Transitions

	B3P86	CAM-B3LYP	LC-wHPBE	M05	mPW1PW91	O3LYP	SVWN	wB97XD	B3LYP
MAE	0.0196307	0.0371507	0.0473793	0.0274636	0.0255479	0.0218021	0.0160307	0.0391721	0.0189679
Slope	0.981965	0.987035	0.982577	0.940967	0.98476	1.01876	1.00958	0.992296	0.976285
Intercept	0.0104826	0.0310747	0.0478049	0.017462	0.0111326	-0.00365072	-0.0117304	0.0314224	0.00983102
R^2	0.99109	0.987616	0.986447	0.985766	0.987926	0.991536	0.994886	0.986876	0.991303
$\|\Delta E\|$	0.197832	0.300024	0.457839	0.244064	0.25902	0.213653	0.250804	0.309654	0.182048
ΔE	0.120104	0.281682	0.457839	0.103777	0.155611	0.0247584	-0.0787657	0.302231	0.0889121
E_{comp}/E_{exp}	1.02768	1.06837	1.11091	1.02602	1.03741	1.00466	0.981188	1.07334	1.02032

Table S195: f_{comp} vs nf_{exp} PCM/TD-DFT/6-311++G**/p-gauge/Improved Fit/ VH Transitions

	B3P86	CAM-B3LYP	LC-wHPBE	M05	mPW1PW91	O3LYP	SVWN	wB97XD	B3LYP
MAE	0.0264021	0.0362164	0.0441521	0.0313721	0.0265179	0.0198793	0.0224107	0.0367736	0.0285421
Slope	0.921644	0.922864	0.910001	0.912155	0.91954	0.990603	0.958609	0.927333	0.959217
Intercept	0.0183616	0.0393583	0.0556959	0.0255201	0.0260968	-0.00112151	-0.00307902	0.0399595	0.021895
R^2	0.990819	0.987701	0.986479	0.985927	0.98992	0.991939	0.99606	0.986968	0.981923
$\|\Delta E\|$	0.197854	0.300374	0.456259	0.243276	0.245818	0.21083	0.249787	0.310658	0.21171
ΔE	0.118989	0.281303	0.456259	0.104565	0.184129	0.028738	-0.0686398	0.302378	0.111592
E_{comp}/E_{exp}	1.0275	1.06831	1.1106	1.02618	1.04311	1.00561	0.983931	1.07338	1.02543

Table S196: f_{comp} vs nf_{exp} PCM/TD-DFT/6-311++G**/xp-gauge/Improved Fit/ VH Transitions

	B3P86	CAM-B3LYP	LC-wHPBE	M05	mPW1PW91	O3LYP	SVWN	wB97XD	B3LYP
MAE	0.0201379	0.0358321	0.0435521	0.0291793	0.0265921	0.0198436	0.0188536	0.036595	0.0219221
Slope	0.951607	0.957	0.944027	0.926533	0.952083	1.01691	0.981129	0.956876	0.94618
Intercept	0.0143763	0.0347805	0.0519963	0.0214017	0.0151154	-0.00441087	-0.00790427	0.0360421	0.0132851
R^2	0.991703	0.987861	0.98658	0.985896	0.988117	0.992685	0.994285	0.98708	0.991411
$\|\Delta E\|$	0.200115	0.300009	0.456705	0.24369	0.259384	0.20931	0.249101	0.309855	0.182867
ΔE	0.122454	0.281681	0.456705	0.104151	0.155281	0.0291105	-0.0802661	0.301899	0.0881085
E_{comp}/E_{exp}	1.02829	1.06838	1.11068	1.0261	1.03734	1.00567	0.980805	1.07327	1.02016

S3.6 Spectrophotometers

S3.6.1 f_{exp}

Looking at specific spectrophotometers:

Table S197: f_{comp} vs f_{exp} PCM/TD-DFT/6-311++G**/x-gauge/Exact Band Limits/ 78 transitions from spectrometer 1: Zeiss PMQ II, MM12

	B3P86	CAM-B3LYP	LC-wHPBE	M05	mPW1PW91	O3LYP	SVWN	wB97XD	B3LYP
MAE	0.126101	0.158213	0.180758	0.129135	0.132423	0.111294	0.114291	0.159227	0.121463
Slope	1.25712	1.28493	0.839823	1.27965	1.26548	1.18367	1.13335	1.29071	1.25876
Intercept	-0.00595285	0.00607392	0.0470267	-0.0067493	-0.00506101	-0.00429323	0.0195252	-0.00542697	0.00157861
R^2	0.906253	0.858926	0.602163	0.917643	0.904718	0.89977	0.882584	0.842312	0.930839

Table S198: f_{comp} vs f_{exp} PCM/TD-DFT/6-311++G**/p-gauge/Exact Band Limits/ 78 transitions from spectrometer 1: Zeiss PMQ II, MM12

	B3P86	CAM-B3LYP	LC-wHPBE	M05	mPW1PW91	O3LYP	SVWN	wB97XD	B3LYP
MAE	0.118176	0.151063	0.173906	0.132312	0.123469	0.103183	0.106592	0.151814	0.112967
Slope	1.22557	1.24818	0.807964	1.28834	1.23518	1.15965	1.11024	1.25387	1.2296
Intercept	-0.00404029	0.0100286	0.0503615	-0.00527037	-0.003149	-0.00341394	0.0177808	-0.00109272	0.00331239
R^2	0.904612	0.854209	0.606385	0.917288	0.904567	0.902642	0.884727	0.838311	0.9308

Table S199: f_{comp} vs f_{exp} PCM/TD-DFT/6-311++G**/x-gauge/Exact Band Limits/ 22 transitions from spectrometer 9 Perkin Elmer 4000 A

	B3P86	CAM-B3LYP	LC-wHPBE	M05	mPW1PW91	O3LYP	SVWN	wB97XD	B3LYP
MAE	0.109307	0.14683	0.176975	0.10052	0.11522	0.113302	0.13403	0.148143	0.112816
Slope	1.4608	1.60272	1.62004	1.45385	1.49775	1.44562	1.37649	1.61792	1.47188
Intercept	-0.018757	-0.058693	-0.111962	-0.0229868	-0.0182383	-0.0254728	-0.0216179	-0.0606311	-0.0176259
R^2	0.890286	0.809906	0.656166	0.896195	0.898006	0.826289	0.659282	0.805625	0.891931

Table S200: f_{comp} vs f_{exp} PCM/TD-DFT/6-311++G**/x-gauge/Exact Band Limits/ 20 transitions from spectrometer 6: Unicam SP 500

	B3P86	CAM-B3LYP	LC-wHPBE	M05	mPW1PW91	O3LYP	SVWN	wB97XD	B3LYP
MAE	0.1053	0.160035	0.182525	0.122775	0.11307	0.096975	0.112915	0.169835	0.107875
Slope	1.28693	1.35739	1.33582	1.3095	1.24325	1.25125	1.11372	1.46875	1.28129
Intercept	0.00766277	0.0355078	-0.0256546	0.0167164	0.0260174	0.0103319	0.0124682	0.0130262	0.0119042
R^2	0.977002	0.938794	0.77674	0.959147	0.960522	0.965787	0.877285	0.957317	0.975159

S3.7 Different Solvents

S3.7.1 f_{exp}

Table S201: f_{comp} vs f_{exp} PCM/TD-DFT/6-311++G**/x-gauge/Exact Band Limits/ 39 transitions Solvent:Ethanol

	B3P86	CAM-B3LYP	LC-wHPBE	M05	mPW1PW91	O3LYP	SVWN	wB97XD	B3LYP
MAE	0.140031	0.163423	0.208541	0.129369	0.1361	0.124969	0.140587	0.179069	0.132941
Slope	1.45875	1.52733	1.40362	1.44458	1.48349	1.40914	1.21697	1.49259	1.4476
Intercept	-0.00971734	-0.0524821	-0.0874668	-0.011928	-0.0190076	-0.0118464	0.0091142	-0.041116	-0.00876146
R^2	0.813185	0.786029	0.559276	0.807385	0.862488	0.802	0.689093	0.723355	0.822403
$\|\Delta E\|$	0.430727	0.794173	1.3345	0.19271	0.293291	0.194586	0.332429	0.810176	0.171923
ΔE	-0.291698	-0.388626	-0.878495	0.00773746	-0.0803244	-0.136673	-0.223781	-0.370684	-0.052695
E_{comp}/E_{exp}	0.943337	0.938819	0.84332	1.00266	0.984201	0.969459	0.948215	0.943402	0.988415

Table S202: f_{comp} vs f_{exp} PCM/TD-DFT/6-311++G**/x-gauge/Exact Band Limits/ Ethanol intersect VHHM: 23 transitions

	B3P86	CAM-B3LYP	LC-wHPBE	M05	mPW1PW91	O3LYP	SVWN	wB97XD	B3LYP
MAE	0.146922	0.175752	0.20727	0.130022	0.147483	0.137748	0.125983	0.1833	0.143187
Slope	1.57245	1.64516	1.68779	1.52426	1.56394	1.58281	1.39591	1.67601	1.55711
Intercept	-0.0294584	-0.0284136	-0.105852	-0.0279985	-0.0212747	-0.040778	-0.00727236	-0.0298908	-0.0249405
R^2	0.869608	0.885529	0.732714	0.861464	0.873429	0.868516	0.896503	0.882536	0.868525
$\|\Delta E\|$	0.422449	0.49751	1.16555	0.195095	0.192174	0.19933	0.421324	0.523723	0.177829
ΔE	-0.238452	-0.001938	-0.559594	0.0428703	0.0813551	-0.120024	-0.292878	0.0269775	-0.023548
E_{comp}/E_{exp}	0.95739	1.01686	0.903043	1.00986	1.01842	0.970133	0.93111	1.0244	0.99356

Table S203: f_{comp} vs f_{exp} PCM/TD-DFT/6-311++G**/x-gauge/Exact Band Limits/ Solvent:Methanol (23 transitions)

	B3P86	CAM-B3LYP	LC-wHPBE	M05	mPW1PW91	O3LYP	SVWN	wB97XD	B3LYP
MAE	0.147052	0.164413	0.187713	0.142387	0.145743	0.166787	0.157674	0.167826	0.144635
Slope	1.25076	1.1291	1.08315	1.2407	1.26045	1.18503	1.03429	1.09206	1.25971
Intercept	-0.0100558	0.0473699	-0.00432243	-0.00909969	-0.00519858	0.00107364	0.0369878	0.0224667	-0.00609458
R^2	0.89951	0.774884	0.711443	0.878504	0.8873	0.713488	0.728573	0.717813	0.908534
$\|\Delta E\|$	0.19819	0.336412	0.793536	0.190482	0.177254	0.228734	0.254715	0.172995	0.186548
ΔE	-0.147366	-0.101079	-0.337957	-0.151357	-0.09411	-0.19013	-0.0656508	0.0941256	-0.152502
E_{comp}/E_{exp}	0.963961	0.980333	0.918072	0.964621	0.97706	0.952936	0.986588	1.02686	0.961587

Table S204: f_{comp} vs f_{exp} PCM/TD-DFT/6-311++G**/x-gauge/Exact Band Limits/ Methanol intercept VHHM: 9 transitions

	B3P86	CAM-B3LYP	LC-wHPBE	M05	mPW1PW91	O3LYP	SVWN	wB97XD	B3LYP
MAE	0.121828	0.162017	0.164494	0.115072	0.126728	0.0926278	0.139761	0.166928	0.125006
Slope	1.2401	1.31776	1.11548	1.23242	1.2645	1.19281	1.13015	1.32703	1.25201
Intercept	0.0208792	0.0284196	0.11594	0.0163526	0.0147001	0.0104625	-0.0155686	0.0294309	0.0190523
R^2	0.987372	0.990201	0.976224	0.988835	0.983443	0.987279	0.880956	0.989891	0.987767
$\|\Delta E\|$	0.184566	0.147463	0.355954	0.197365	0.18175	0.240091	0.31362	0.168382	0.199737
ΔE	-0.101613	0.0871334	0.355954	-0.123697	-0.0612328	-0.219773	0.00119542	0.129816	-0.137387
E_{comp}/E_{exp}	0.970917	1.02663	1.09792	0.967426	0.9814	0.941141	1.00546	1.038	0.961951

Table S205: f_{comp} vs f_{exp} PCM/TD-DFT/6-311++G**/x-gauge/Exact Band Limits/ Solvent:Heptane (29 transitions)

	B3P86	CAM-B3LYP	LC-wHPBE	M05	mPW1PW91	O3LYP	SVWN	wB97XD	B3LYP
MAE	0.154317	0.200459	0.203783	0.163276	0.160645	0.129083	0.123003	0.211545	0.144428
Slope	1.46038	1.62741	0.650983	1.47275	1.46174	1.27254	1.22204	1.66438	1.39345
Intercept	-0.0327812	-0.0600406	0.0852594	-0.0354541	-0.0320961	0.00386999	0.0263812	-0.0625476	-0.0167258
R^2	0.968627	0.973795	0.417207	0.971539	0.971006	0.940399	0.946139	0.972859	0.962535
$\|\Delta E\|$	0.144019	0.44799	0.88629	0.20126	0.293381	0.176711	0.232633	0.468872	0.150852
ΔE	0.0199258	-0.190924	-0.540134	-0.0819385	-0.118703	-0.0877992	-0.197633	-0.163933	-0.0304949
E_{comp}/E_{exp}	1.00475	0.951344	0.880968	0.987665	0.973789	0.985803	0.965252	0.955992	0.996122

Table S206: f_{comp} vs f_{exp} PCM/TD-DFT/6-311++G**/x-gauge/Exact Band Limits/ Heptane intersect VHHM: 10 transitions

	B3P86	CAM-B3LYP	LC-wHPBE	M05	mPW1PW91	O3LYP	SVWN	wB97XD	B3LYP
MAE	0.091665	0.109055	0.136125	0.097435	0.102775	0.088875	0.101125	0.111495	0.085275
Slope	1.35286	1.42382	1.49311	1.38919	1.40327	1.29671	1.18524	1.43687	1.35491
Intercept	-0.0196181	-0.0236809	-0.0410143	-0.0279171	-0.0223721	-0.00266596	0.0410196	-0.0238522	-0.0241543
R^2	0.952114	0.939077	0.908381	0.943069	0.947415	0.911308	0.857683	0.941806	0.963201
$\|\Delta E\|$	0.171582	0.19948	0.73845	0.228121	0.180891	0.20091	0.216005	0.215848	0.182645
ΔE	0.00571585	0.15072	-0.335296	-0.0710219	0.0616577	-0.0610852	-0.161404	0.176798	-0.0267883
E_{comp}/E_{exp}	1.00282	1.03085	0.925104	0.989321	1.0136	0.990573	0.969445	1.03575	0.996831

Table S207: f_{comp} vs f_{exp} PCM/TD-DFT/6-311++G**/x-gauge/Exact Band Limits/ Solvent:Hexane (12 transitions)

	B3P86	CAM-B3LYP	LC-wHPBE	M05	mPW1PW91	O3LYP	SVWN	wB97XD	B3LYP
MAE	0.08845	0.119742	0.148233	0.086475	0.0897333	0.0938667	0.149083	0.120567	0.0964833
Slope	1.34553	1.40295	1.51447	1.31334	1.3554	1.25446	0.919649	1.40816	1.31436
Intercept	-0.0458333	-0.0471081	-0.0536284	-0.0317304	-0.0353451	-0.0189759	0.0182458	-0.0476088	-0.0283809
R^2	0.832791	0.771979	0.788277	0.836534	0.837643	0.791574	0.37461	0.773588	0.813227
$\|\Delta E\|$	0.752446	1.10662	1.18048	0.747872	0.712212	0.819217	1.19706	1.12278	0.760955
ΔE	-0.737299	-0.950278	-0.828584	-0.702236	-0.655156	-0.802468	-1.0751	-0.947826	-0.742743
E_{comp}/E_{exp}	0.865076	0.832715	0.860363	0.871578	0.881789	0.850829	0.808929	0.833089	0.863598

Table S208: f_{comp} vs f_{exp} PCM/TD-DFT/6-311++G**/x-gauge/Exact Band Limits/ Hexane intercept VHHM: 7 transitions

	B3P86	CAM-B3LYP	LC-wHPBE	M05	mPW1PW91	O3LYP	SVWN	wB97XD	B3LYP
MAE	0.115857	0.164743	0.203357	0.106371	0.112943	0.106086	0.206114	0.1645	0.112643
Slope	1.34277	1.42433	1.39679	1.30107	1.33486	1.30121	0.902362	1.44004	1.32848
Intercept	-0.0426067	-0.0556115	0.0030005	-0.0257185	-0.0246555	-0.043197	0.0226555	-0.0613763	-0.0382265
R^2	0.776111	0.697697	0.684585	0.790975	0.785476	0.759557	0.267529	0.70404	0.784181
$\|\Delta E\|$	0.32215	0.865079	0.246992	0.281778	0.25001	0.442369	0.97167	0.886424	0.344911
ΔE	-0.319727	-0.844485	0.106035	-0.281778	-0.243186	-0.442369	-0.762596	-0.85052	-0.344911
E_{comp}/E_{exp}	0.928095	0.84241	1.02578	0.935904	0.944737	0.901374	0.858004	0.841079	0.922822

Table S209: f_{comp} vs f_{exp} PCM/TD-DFT/6-311++G**/x-gauge/Exact Band Limits/Cyclohexane intercept VHHM: 6 transitions

	B3P86	CAM-B3LYP	LC-wHPBE	M05	mPW1PW91	O3LYP	SVWN	wB97XD	B3LYP
MAE	0.120317	0.19485	0.212033	0.11075	0.13745	0.156683	0.135017	0.196517	0.126933
Slope	1.49016	1.56334	2.03591	1.36728	1.53966	1.75495	1.03621	1.61773	1.59461
Intercept	-0.0784532	-0.0271988	-0.325239	-0.0340195	-0.0752665	-0.202942	-0.0584912	-0.0469707	-0.116977
R^2	0.927723	0.925804	0.602993	0.94467	0.959258	0.666776	0.306605	0.874164	0.975621
$\|\Delta E\|$	0.222401	0.206209	0.331918	0.170826	0.162994	0.247507	0.209882	0.232036	0.21698
ΔE	-0.217843	0.174324	0.187305	-0.140082	-0.13159	-0.225382	0.118813	0.20927	-0.211898
E_{comp}/E_{exp}	0.954847	1.03946	1.04807	0.972539	0.97373	0.955104	1.0238	1.0473	0.956757

Table S210: f_{comp} vs f_{exp} PCM/TD-DFT/6-311++G**/x-gauge/Exact Band Limits/ 15 transitions Solvent:water

	B3P86	CAM-B3LYP	LC-wHPBE	M05	mPW1PW91	O3LYP	SVWN	wB97XD	B3LYP
MAE	0.0795567	0.111297	0.121143	0.0765433	0.09185	0.0694567	0.08337	0.0907567	0.0745633
Slope	0.901601	0.918895	0.825984	0.94624	0.921495	0.922744	0.802811	0.951534	0.95277
Intercept	0.0234664	0.0235661	0.0418406	0.0113989	0.0163955	0.0126823	0.0602681	0.020176	0.0121458
R^2	0.832191	0.745468	0.748927	0.849291	0.823767	0.873348	0.883348	0.852176	0.857202
$\|\Delta E\|$	0.172781	1.10326	1.25306	0.144019	0.740882	0.202988	0.264332	1.14189	0.159964
ΔE	0.0192412	-0.818945	-1.02446	-0.00661347	-0.657471	-0.123681	-0.140452	-0.843521	-0.062025
E_{comp}/E_{exp}	0.999942	0.831561	0.747516	0.996631	0.867072	0.970453	0.968194	0.829118	0.98446

Table S211: f_{comp} vs f_{exp} PCM/TD-DFT/6-311++G**/x-gauge/Exact Band Limits/Water intersect VHHM: 11 transitions

	B3P86	CAM-B3LYP	LC-wHPBE	M05	mPW1PW91	O3LYP	SVWN	wB97XD	B3LYP
MAE	0.0577	0.0833545	0.109791	0.0515182	0.0663364	0.0483455	0.0915091	0.0726273	0.0521818
Slope	0.950134	0.977111	0.869806	0.999626	0.958323	0.96221	0.77311	0.976919	1.00151
Intercept	0.0628068	0.080093	0.0686665	0.0479082	0.0661715	0.0480264	0.0603583	0.0686847	0.0505727
R^2	0.966375	0.947501	0.845879	0.977809	0.961495	0.97376	0.899684	0.958006	0.979735
$\|\Delta E\|$	0.17281	0.974301	1.6432	0.153736	0.504686	0.243305	0.309067	0.9967	0.182956
ΔE	-0.0211899	-0.676538	-1.33247	-0.0203467	-0.431058	-0.143958	-0.196145	-0.654139	-0.0766945
E_{comp}/E_{exp}	0.991996	0.853526	0.667496	0.993418	0.90643	0.964186	0.956198	0.859023	0.979959

Table S212: f_{comp} vs f_{exp} PCM/TD-DFT/6-311++G**/x-gauge/Exact Band Limits/ Water intersect VHHM: 6 transitions

	B3P86	CAM-B3LYP	LC-wHPBE	M05	mPW1PW91	O3LYP	SVWN	wB97XD	B3LYP
MAE	0.0667333	0.0824	0.110317	0.0638833	0.0701667	0.0549	0.0770167	0.0849167	0.06535
Slope	1.41308	1.57413	1.89325	1.38328	1.56911	1.32045	1.28889	1.5833	1.39969
Intercept	0.00535625	-0.00836275	-0.0741995	0.00763084	-0.0198599	0.00338471	-0.0248329	-0.00719291	0.00540507
R^2	0.953489	0.972822	0.781707	0.975296	0.969031	0.943123	0.602378	0.967627	0.953466
$\|\Delta E\|$	0.195305	0.953541	1.55229	0.189286	0.851148	0.26677	0.369284	0.989565	0.212038
ΔE	0.00133385	-0.598205	-1.21195	0.00673976	-0.771604	-0.139115	-0.285101	-0.562181	-0.0489976
E_{comp}/E_{exp}	0.997855	0.877277	0.70393	0.999947	0.834239	0.963896	0.927438	0.885994	0.985811

Table S213: f_{comp} vs f_{exp} PCM/TD-DFT/6-311++G**/x-gauge/Exact Band Limits/ 22 transitions Solvent:ions in aq solution

	B3P86	CAM-B3LYP	LC-wHPBE	M05	mPW1PW91	O3LYP	SVWN	wB97XD	B3LYP
MAE	0.110348	0.161298	0.201016	0.12572	0.127466	0.12142	0.110925	0.156457	0.118675
Slope	1.14129	1.15819	0.91345	1.21028	1.14882	1.10901	1.26399	1.17674	1.17952
Intercept	0.0171425	0.0425844	0.0722053	0.0285872	0.0248562	0.00316707	-0.0385665	0.0227026	0.0332215
R^2	0.84228	0.748162	0.532508	0.869852	0.815662	0.77858	0.867047	0.7422	0.874661
$\|\Delta E\|$	0.163912	0.161063	0.539721	0.144829	0.154954	0.193259	0.194145	0.173825	0.161787
ΔE	-0.0242406	0.136281	-0.0613602	-0.0240523	0.0281614	-0.106325	0.0067149	0.155901	-0.0405451
E_{comp}/E_{exp}	0.99435	1.03298	1.01056	0.996056	1.00686	0.977366	1.00532	1.0375	0.990454

Table S214: f_{comp} vs f_{exp} PCM/TD-DFT/6-311++G**/x-gauge/Exact Band Limits/ Ions intersection VHHM: 12 transitions

	B3P86	CAM-B3LYP	LC-wHPBE	M05	mPW1PW91	O3LYP	SVWN	wB97XD	B3LYP
MAE	0.121479	0.165896	0.191704	0.124946	0.130871	0.114163	0.101404	0.168021	0.119479
Slope	1.39465	1.33259	1.35981	1.3567	1.39678	1.39244	1.37882	1.33547	1.37973
Intercept	-0.0122114	0.0250059	0.0408099	5.15502e-05	-0.00480814	-0.0308473	-0.0639186	0.0250214	-0.00908976
R^2	0.76144	0.617179	0.588882	0.745015	0.736555	0.768377	0.80143	0.610444	0.76922
$\|\Delta E\|$	0.201187	0.193792	0.32543	0.170349	0.190855	0.210777	0.199408	0.20269	0.197667
ΔE	0.000737716	0.164294	0.32543	0.0170964	0.0536581	-0.0595749	0.120267	0.185762	-0.0212486
E_{comp}/E_{exp}	1.00102	1.04528	1.08713	1.00666	1.01535	0.985952	1.03152	1.05072	0.995274

S3.7.2 Solvent: Non-polar

S3.7.3 f_{exp}

Avg $f_{exp} = 0.324309$

Table S215: f_{comp} vs f_{exp} PCM/TD-DFT/6-311++G**/x-gauge/Exact Band Limits/

	B3P86	CAM-B3LYP	LC-wHPBE	M05	mPW1PW91	O3LYP	SVWN	wB97XD	B3LYP
MAE	0.118794	0.153209	0.183472	0.118378	0.122209	0.113425	0.147003	0.161647	0.116991
Slope	1.317	1.35206	1.50505	1.31827	1.27479	1.25206	1.04743	1.43565	1.32169
Intercept	-0.00400884	0.0115628	-0.0628981	-0.00328599	0.0176696	0.00273118	0.0453042	-0.00696823	-0.00680721
R^2	0.932877	0.907576	0.869443	0.931138	0.919661	0.896593	0.669023	0.918454	0.938955
$\|\Delta E\|$	0.229685	0.372221	0.84226	0.217618	0.197282	0.282329	0.418599	0.391706	0.235558
ΔE	-0.122735	-0.036986	-0.435654	-0.124148	-0.0494986	-0.210208	-0.223062	-0.0191544	-0.146298
E_{comp}/E_{exp}	0.972429	0.997088	0.903752	0.973568	0.988291	0.954157	0.960248	1.00049	0.967822

Table S216: f_{comp} vs f_{exp} PCM/TD-DFT/6-311++G**/p-gauge/Exact Band Limits/

	B3P86	CAM-B3LYP	LC-wHPBE	M05	mPW1PW91	O3LYP	SVWN	wB97XD	B3LYP
MAE	0.108091	0.137662	0.166331	0.120203	0.111491	0.105347	0.135056	0.146594	0.107103
Slope	1.28436	1.31382	1.46484	1.32781	1.24246	1.22591	1.03082	1.39677	1.29174
Intercept	-0.00814286	0.00792913	-0.0668026	-0.0071532	0.0130509	-0.0010972	0.037608	-0.0105688	-0.0108
R^2	0.932479	0.911518	0.875039	0.931247	0.921858	0.897261	0.686336	0.920766	0.93908
$\|\Delta E\|$	0.229689	0.370976	0.841509	0.217746	0.196642	0.283463	0.41964	0.390344	0.235843
ΔE	-0.123149	-0.0377508	-0.436148	-0.124174	-0.0497216	-0.209041	-0.22204	-0.019994	-0.146091
E_{comp}/E_{exp}	0.972353	0.996936	0.903663	0.973561	0.98825	0.954392	0.960498	1.00032	0.967867

Table S217: f_{comp} vs f_{exp} PCM/TD-DFT/6-311++G**/xp-gauge/Exact Band Limits/

	B3P86	CAM-B3LYP	LC-wHPBE	M05	mPW1PW91	O3LYP	SVWN	wB97XD	B3LYP
MAE	0.113106	0.145084	0.174506	0.118922	0.116534	0.108987	0.140797	0.153413	0.111756
Slope	1.30067	1.33291	1.48491	1.32305	1.25854	1.23896	1.03919	1.41613	1.30666
Intercept	-0.00633252	0.00954446	-0.0650709	-0.00539417	0.015122	0.000598173	0.0411603	-0.00900549	-0.00902437
R^2	0.933042	0.910029	0.872645	0.931547	0.921164	0.897207	0.678177	0.920079	0.93934
$\|\Delta E\|$	0.229728	0.371675	0.841902	0.217577	0.197046	0.282927	0.419116	0.391073	0.235747
ΔE	-0.122947	-0.0373139	-0.435854	-0.124159	-0.0495925	-0.209635	-0.222497	-0.0195292	-0.146191
E_{comp}/E_{exp}	0.972389	0.997022	0.903716	0.973565	0.988275	0.95427	0.960385	1.00042	0.967843

Improving fit

Table S218: f_{comp} vs f_{exp} PCM/TD-DFT/6-311++G**/x-gauge/Improving Fit/

	B3P86	CAM-B3LYP	LC-wHPBE	M05	mPW1PW91	O3LYP	SVWN	wB97XD	B3LYP
MAE	0.06185	0.0954187	0.126541	0.0686031	0.0664594	0.0438344	0.046625	0.104744	0.0643781
Slope	1.07428	1.06427	1.1537	1.08077	1.075	1.05591	0.91849	1.1974	1.11532
Intercept	0.0156009	0.0371226	0.00830941	0.016454	0.022159	0.00485771	0.00530307	0.00414857	0.00226121
R^2	0.922901	0.855674	0.800588	0.918373	0.916972	0.940426	0.823778	0.896591	0.932073
$\|\Delta E\|$	0.291619	0.248284	0.737204	0.223535	0.251555	0.461811	0.480869	0.273125	0.292016
ΔE	-0.0845064	0.143672	-0.186188	-0.120182	0.0129639	-0.235093	-0.266441	0.135939	-0.131549
E_{comp}/E_{exp}	0.981387	1.02856	0.950751	0.974412	1.00201	0.946022	0.948397	1.0272	0.972282

Table S219: f_{comp} vs f_{exp} PCM/TD-DFT/6-311++G**/p-gauge/Improving Fit/

	B3P86	CAM-B3LYP	LC-wHPBE	M05	mPW1PW91	O3LYP	SVWN	wB97XD	B3LYP
MAE	0.0543156	0.0829719	0.110981	0.0663813	0.0612875	0.0392875	0.045475	0.0921156	0.0570094
Slope	1.03921	1.02304	1.1207	1.08087	1.07128	1.04411	0.925034	1.15789	1.0896
Intercept	0.0133819	0.0534946	0.00609089	0.0144081	0.0198627	-0.000962262	-0.000478345	0.0194334	-0.00165927
R^2	0.928036	0.891414	0.824391	0.928545	0.931725	0.947311	0.829929	0.923168	0.937702
$\|\Delta E\|$	0.283625	0.241087	0.629022	0.223327	0.216244	0.452958	0.471658	0.262055	0.294253
ΔE	-0.0959961	0.170716	-0.0643109	-0.119692	-0.0247976	-0.238486	-0.275708	0.160954	-0.13327
E_{comp}/E_{exp}	0.979431	1.03589	0.983848	0.974489	0.993238	0.945559	0.94676	1.0342	0.971906

Table S220: f_{comp} vs f_{exp} PCM/TD-DFT/6-311++G**/xp-gauge/Improving Fit/

	B3P86	CAM-B3LYP	LC-wHPBE	M05	mPW1PW91	O3LYP	SVWN	wB97XD	B3LYP
MAE	0.0570688	0.089	0.117619	0.0671188	0.0646188	0.0414187	0.0453219	0.0970094	0.0583156
Slope	1.06023	1.03772	1.13182	1.07945	1.07752	1.0473	0.92867	1.16853	1.09855
Intercept	0.0134124	0.0558831	0.0114667	0.0161946	0.0131491	0.00264809	0.00113287	0.0243998	0.0031041
R^2	0.926856	0.882657	0.814473	0.924021	0.92289	0.94372	0.827845	0.92019	0.93867
$\|\Delta E\|$	0.291733	0.240755	0.623756	0.222376	0.245561	0.459962	0.474343	0.250206	0.277338
ΔE	-0.0849327	0.170464	-0.0461233	-0.119654	-0.00287655	-0.23097	-0.26075	0.178759	-0.117321
E_{comp}/E_{exp}	0.98128	1.03584	0.987	0.974507	0.99875	0.9468	0.949355	1.0373	0.974693

S3.7.4 nf_{exp}

Avg $nf_{exp} = 0.473265$

Table S221: f_{comp} vs nf_{exp} PCM/TD-DFT/6-311++G**/x-gauge/Exact Band Limits/

	B3P86	CAM-B3LYP	LC-wHPBE	M05	mPW1PW91	O3LYP	SVWN	wB97XD	B3LYP
MAE	0.0866322	0.0898709	0.109303	0.0883272	0.0878791	0.108295	0.172642	0.0842959	0.0842459
Slope	0.891276	0.914474	1.01748	0.892011	0.861822	0.845654	0.707775	0.971862	0.894421
Intercept	0.00129665	0.0172616	-0.0563354	0.00208329	0.023227	0.00856606	0.0500319	-0.00132282	-0.00146963
R^2	0.930181	0.903899	0.865134	0.928189	0.915109	0.890479	0.66507	0.916349	0.936183
$\|\Delta E\|$	0.229685	0.372221	0.84226	0.217618	0.197282	0.282329	0.418599	0.391706	0.235558
ΔE	-0.122735	-0.036986	-0.435654	-0.124148	-0.0494986	-0.210208	-0.223062	-0.0191544	-0.146298
E_{comp}/E_{exp}	0.972429	0.997088	0.903752	0.973568	0.988291	0.954157	0.960248	1.00049	0.967822

Table S222: f_{comp} vs nf_{exp} PCM/TD-DFT/6-311++G**/p-gauge/Exact Band Limits/

	B3P86	CAM-B3LYP	LC-wHPBE	M05	mPW1PW91	O3LYP	SVWN	wB97XD	B3LYP
MAE	0.0920009	0.0889522	0.11227	0.0880697	0.0931197	0.112313	0.171576	0.0835241	0.0895797
Slope	0.869552	0.889011	0.99084	0.898846	0.840354	0.828298	0.696774	0.945957	0.874512
Intercept	-0.00314087	0.0132751	-0.0606704	-0.00192329	0.018284	0.00447387	0.0421541	-0.00527254	-0.00575062
R^2	0.930562	0.908654	0.871651	0.929076	0.918142	0.891787	0.682722	0.919462	0.937065
$\|\Delta E\|$	0.229689	0.370976	0.841509	0.217746	0.196642	0.283463	0.41964	0.390344	0.235843
ΔE	-0.123149	-0.0377508	-0.436148	-0.124174	-0.0497216	-0.209041	-0.22204	-0.019994	-0.146091
E_{comp}/E_{exp}	0.972353	0.996936	0.903663	0.973561	0.98825	0.954392	0.960498	1.00032	0.967867

Table S223: f_{comp} vs nf_{exp} PCM/TD-DFT/6-311++G**/xp-gauge/Exact Band Limits/

	B3P86	CAM-B3LYP	LC-wHPBE	M05	mPW1PW91	O3LYP	SVWN	wB97XD	B3LYP
MAE	0.0894009	0.0893022	0.11045	0.0882103	0.0905916	0.109632	0.172067	0.0837928	0.0869928
Slope	0.880413	0.901724	1.00414	0.895438	0.851032	0.836955	0.702313	0.958865	0.884433
Intercept	-0.00118092	0.0150645	-0.058726	-9.46933e-05	0.0205209	0.00630815	0.0458108	-0.00353748	-0.00383349
R^2	0.930739	0.906759	0.868796	0.928988	0.917033	0.89142	0.674403	0.918378	0.936952
$\|\Delta E\|$	0.229728	0.371675	0.841902	0.217577	0.19705	0.282903	0.419148	0.391073	0.235747
ΔE	-0.122947	-0.0373139	-0.435854	-0.124159	-0.0495967	-0.209611	-0.222529	-0.0195292	-0.146191
E_{comp}/E_{exp}	0.972389	0.997022	0.903716	0.973565	0.988274	0.954275	0.960379	1.00042	0.967843

Improving Fit:

Table S224: f_{comp} vs nf_{exp} PCM/TD-DFT/6-311++G**/x-gauge/Improved Fit/

	B3P86	CAM-B3LYP	LC-wHPBE	M05	mPW1PW91	O3LYP	SVWN	wB97XD	B3LYP
MAE	0.0487178	0.0430016	0.0685034	0.0480122	0.0444178	0.0604659	0.0773147	0.0465034	0.0486691
Slope	0.983074	0.997515	1.03828	0.982833	0.98429	0.88168	0.81043	1.00253	0.961739
Intercept	-0.0192041	0.000464425	-0.0305703	-0.0145372	-0.0132577	0.0109507	0.0236678	-0.00244408	-0.0116135
R^2	0.962004	0.973115	0.922107	0.964136	0.968015	0.939007	0.851087	0.968739	0.962985
$\|\Delta E\|$	0.211497	0.234909	0.425515	0.198533	0.211054	0.229034	0.472348	0.248405	0.213695
ΔE	-0.0558111	0.17187	0.361111	-0.0616733	0.00587029	-0.138061	-0.279935	0.1815	-0.0864837
E_{comp}/E_{exp}	0.984938	1.03511	1.07471	0.985049	0.998641	0.967836	0.943644	1.03712	0.979067

Table S225: f_{comp} vs nf_{exp} PCM/TD-DFT/6-311++G**/p-gauge/Improved Fit/

	B3P86	CAM-B3LYP	LC-wHPBE	M05	mPW1PW91	O3LYP	SVWN	wB97XD	B3LYP
MAE	0.0500041	0.0408847	0.0681234	0.0467541	0.0451772	0.0617116	0.0803472	0.0470816	0.0504022
Slope	0.979332	0.991911	1.02098	0.98501	0.973028	0.869244	0.812475	1.00109	0.956966
Intercept	-0.0172364	-0.00576476	-0.0368807	-0.0172484	-0.0127371	0.0108676	0.024356	-0.00311903	-0.0105075
R^2	0.954499	0.974387	0.928075	0.964071	0.964696	0.934796	0.84232	0.96789	0.955802
$\|\Delta E\|$	0.217005	0.251636	0.666411	0.192269	0.201292	0.240876	0.482077	0.267695	0.206756
ΔE	-0.0207342	0.190655	0.036267	-0.0562438	0.0357328	-0.108312	-0.280882	0.201938	-0.0624085
E_{comp}/E_{exp}	0.991531	1.03837	1.00242	0.986301	1.00416	0.973654	0.943106	1.04066	0.983589

Table S226: f_{comp} vs nf_{exp} PCM/TD-DFT/6-311++G**/xp-gauge/Improved Fit/

	B3P86	CAM-B3LYP	LC-wHPBE	M05	mPW1PW91	O3LYP	SVWN	wB97XD	B3LYP
MAE	0.0477472	0.0427441	0.0675309	0.0477203	0.0456947	0.0594691	0.0793084	0.0472616	0.0480866
Slope	0.971443	0.998997	1.0353	0.98439	0.981707	0.882239	0.807995	0.993704	0.961955
Intercept	-0.0171966	-0.0020464	-0.0395944	-0.0175896	-0.0158634	0.00916767	0.024198	-0.0060571	-0.00894374
R^2	0.959735	0.97416	0.927158	0.965239	0.967031	0.935754	0.848008	0.968928	0.958549
$\|\Delta E\|$	0.22766	0.248295	0.544476	0.195641	0.221645	0.236937	0.487016	0.248896	0.206046
ΔE	-0.0372803	0.187469	0.199495	-0.060582	0.019656	-0.115438	-0.294145	0.182079	-0.0651941
E_{comp}/E_{exp}	0.988258	1.03783	1.0388	0.985392	1.00095	0.972392	0.940462	1.0372	0.983025

S3.7.5 Solvent: Polar

S3.7.6 f_{exp}

Avg f_{exp} = 0.279478

Table S227: f_{comp} vs f_{exp} PCM/TD-DFT/6-311++G**/x-gauge/Exact Band Limits/

	B3P86	CAM-B3LYP	LC-wHPBE	M05	mPW1PW91	O3LYP	SVWN	wB97XD	B3LYP
MAE	0.126676	0.159712	0.1842	0.118176	0.130482	0.114024	0.116688	0.16488	0.124884
Slope	1.37955	1.43547	1.37075	1.34892	1.38945	1.35898	1.26551	1.45188	1.37836
Intercept	0.00508867	0.0141768	0.00577638	0.00714888	0.00725851	-0.00485635	-0.0108654	0.0144625	0.00575591
R^2	0.881302	0.86364	0.746671	0.878591	0.879427	0.872816	0.854722	0.859638	0.885785
$\|\Delta E\|$	0.29927	0.416333	0.864604	0.188867	0.269058	0.217507	0.342432	0.438615	0.190638
ΔE	-0.127641	-0.0175615	-0.260673	0.0023668	-0.0533131	-0.125762	-0.139857	0.0128977	-0.0465412
E_{comp}/E_{exp}	0.975152	1.00869	0.958409	1.00026	0.988916	0.967962	0.96815	1.01656	0.987352

Table S228: f_{comp} vs f_{exp} PCM/TD-DFT/6-311++G**/p-gauge/Exact Band Limits/

	B3P86	CAM-B3LYP	LC-wHPBE	M05	mPW1PW91	O3LYP	SVWN	wB97XD	B3LYP
MAE	0.115286	0.148638	0.17344	0.118502	0.118594	0.103888	0.106292	0.153814	0.113854
Slope	1.32023	1.36984	1.30174	1.33503	1.32917	1.30927	1.22092	1.38529	1.32238
Intercept	0.0100497	0.021005	0.0141276	0.0121161	0.0120142	-0.00119392	-0.00898365	0.0215303	0.010142
R^2	0.884394	0.86396	0.744196	0.881285	0.882423	0.877078	0.858651	0.859351	0.888736
$\|\Delta E\|$	0.300776	0.41384	0.86407	0.189213	0.269309	0.219224	0.404556	0.436291	0.192469
ΔE	-0.129995	-0.0191387	-0.262067	0.000104663	-0.0559574	-0.126005	-0.218126	0.0112354	-0.0493895
E_{comp}/E_{exp}	0.974541	1.00823	0.958061	0.99967	0.988224	0.967921	0.946454	1.01608	0.986636

Table S229: f_{comp} vs f_{exp} PCM/TD-DFT/6-311++G**/xp-gauge/Exact Band Limits/

	B3P86	CAM-B3LYP	LC-wHPBE	M05	mPW1PW91	O3LYP	SVWN	wB97XD	B3LYP
MAE	0.120836	0.153996	0.178644	0.11819	0.1244	0.108716	0.11138	0.159156	0.119232
Slope	1.34962	1.40241	1.3359	1.34201	1.35907	1.33388	1.24292	1.41827	1.35008
Intercept	0.00745329	0.0174593	0.00985252	0.00948435	0.00951652	-0.00313545	-0.0100234	0.0178738	0.00783699
R^2	0.883006	0.864068	0.745767	0.880062	0.881087	0.875057	0.856762	0.859761	0.887371
$\|\Delta E\|$	0.3001	0.41503	0.864362	0.188967	0.26924	0.218097	0.405914	0.437403	0.191624
ΔE	-0.128916	-0.0184082	-0.261353	0.00117382	-0.0546574	-0.125882	-0.219047	0.0120256	-0.0480369
E_{comp}/E_{exp}	0.974819	1.00844	0.958238	0.99995	0.988563	0.967944	0.946228	1.01631	0.986974

Improving fit

Table S230: f_{comp} vs f_{exp} PCM/TD-DFT/6-311++G**/x-gauge/Improving Fit/

	B3P86	CAM-B3LYP	LC-wHPBE	M05	mPW1PW91	O3LYP	SVWN	wB97XD	B3LYP
MAE	0.074976	0.112134	0.12171	0.08262	0.079498	0.065468	0.052262	0.118894	0.070806
Slope	1.16859	1.13093	1.1699	1.09761	1.18808	1.18118	1.15879	1.14946	1.15934
Intercept	0.020792	0.0313073	0.0162752	0.0217611	0.0203634	0.00591126	-0.015709	0.0338526	0.0173989
R^2	0.906544	0.76527	0.747597	0.810582	0.901766	0.923477	0.935255	0.768627	0.906201
$\|\Delta E\|$	0.283654	0.413266	0.824923	0.36476	0.349406	0.310488	0.450575	0.55549	0.271694
ΔE	0.0800658	0.0963861	0.00140131	-0.0865677	0.0439441	-0.0965755	-0.236521	0.0130311	0.0496419
E_{comp}/E_{exp}	1.02145	1.01922	0.99992	0.973568	1.01702	0.974981	0.94371	1.00804	1.0149

Table S231: f_{comp} vs f_{exp} PCM/TD-DFT/6-311++G**/p-gauge/Improved Fit/

	B3P86	CAM-B3LYP	LC-wHPBE	M05	mPW1PW91	O3LYP	SVWN	wB97XD	B3LYP
MAE	0.065132	0.105738	0.112168	0.080266	0.069096	0.067044	0.041876	0.109936	0.061902
Slope	1.12786	1.07594	1.11159	1.19725	1.14825	1.17606	1.11536	1.09865	1.12977
Intercept	0.0215325	0.0380953	0.0219559	0.0135957	0.017895	0.00558228	-0.0099757	0.0370701	0.0179456
R^2	0.917803	0.769791	0.751671	0.880984	0.910805	0.86891	0.947056	0.773399	0.922177
$\|\Delta E\|$	0.284438	0.357539	0.807745	0.241356	0.354941	0.303521	0.460418	0.562312	0.273886
ΔE	0.0579081	0.151301	-0.0157997	0.0468746	0.0339369	-0.101042	-0.22587	0.0274445	0.0488563
E_{comp}/E_{exp}	1.0151	1.04105	0.996039	1.01117	1.01456	0.975053	0.946059	1.01208	1.01455

Table S232: f_{comp} vs f_{exp} PCM/TD-DFT/6-311++G**/xp-gauge/Improving Fit/

	B3P86	CAM-B3LYP	LC-wHPBE	M05	mPW1PW91	O3LYP	SVWN	wB97XD	B3LYP
MAE	0.070238	0.109532	0.117016	0.081744	0.080528	0.070592	0.047956	0.114322	0.066236
Slope	1.15013	1.10092	1.13928	1.2003	1.27329	1.19821	1.13516	1.12366	1.14925
Intercept	0.0197472	0.0362203	0.0191452	0.0145515	-0.00337047	0.0040216	-0.013499	0.0354269	0.0173415
R^2	0.912023	0.767025	0.749177	0.877651	0.935666	0.864424	0.937132	0.771117	0.917296
$\|\Delta E\|$	0.285928	0.403833	0.824663	0.320663	0.351202	0.299245	0.449735	0.562113	0.273205
ΔE	0.0568223	0.083311	0.00112533	-0.0416061	0.0416592	-0.0971037	-0.237068	0.0270217	0.0500275
E_{comp}/E_{exp}	1.0149	1.01671	0.999857	0.991016	1.01633	0.975889	0.943539	1.01199	1.01488

S3.7.7 nf_{exp}

Avg $nf_{exp} = 0.384494$

Table S233: f_{comp} vs nf_{exp} PCM/TD-DFT/6-311++G**/x-gauge/Exact Band Limits/

	B3P86	CAM-B3LYP	LC-wHPBE	M05	mPW1PW91	O3LYP	SVWN	wB97XD	B3LYP
MAE	0.0695	0.0825984	0.132832	0.0681356	0.0707416	0.0761528	0.0994984	0.0863624	0.0682356
Slope	0.993818	1.03444	0.984254	0.971808	1.00134	0.979045	0.913943	1.04631	0.993217
Intercept	0.00852491	0.0176203	0.0104324	0.0104877	0.0105689	-0.00148874	-0.00858952	0.0179318	0.00909008
R^2	0.876993	0.859989	0.738169	0.874395	0.875812	0.86863	0.854801	0.856061	0.881913
$\|\Delta E\|$	0.29927	0.416333	0.864604	0.188867	0.269058	0.217507	0.342432	0.438615	0.190638
ΔE	-0.127641	-0.0175615	-0.260673	0.0023668	-0.0533131	-0.125762	-0.139857	0.0128977	-0.0465412
E_{comp}/E_{exp}	0.975152	1.00869	0.958409	1.00026	0.988916	0.967962	0.96815	1.01656	0.987352

Table S234: f_{comp} vs nf_{exp} PCM/TD-DFT/6-311++G**/p-gauge/Exact Band Limits/

	B3P86	CAM-B3LYP	LC-wHPBE	M05	mPW1PW91	O3LYP	SVWN	wB97XD	B3LYP
MAE	0.068594	0.079112	0.129077	0.0687252	0.0677408	0.0747476	0.099624	0.0822708	0.0674496
Slope	0.951283	0.987356	0.934908	0.962014	0.958147	0.943454	0.881864	0.998514	0.953074
Intercept	0.0132635	0.0242114	0.0184695	0.0153392	0.0150864	0.00196549	-0.00683523	0.0247653	0.0132667
R^2	0.88043	0.860669	0.736048	0.877461	0.879248	0.87328	0.85897	0.856111	0.885209
$\|\Delta E\|$	0.300776	0.41384	0.86407	0.189213	0.269309	0.219224	0.404556	0.436291	0.192469
ΔE	-0.129995	-0.0191387	-0.262067	0.000104663	-0.0559574	-0.126005	-0.218126	0.0112354	-0.0493895
E_{comp}/E_{exp}	0.974541	1.00823	0.958061	0.99967	0.988224	0.967921	0.946454	1.01608	0.986636

Table S235: f_{comp} vs nf_{exp} PCM/TD-DFT/6-311++G**/xp-gauge/Exact Band Limits/

	B3P86	CAM-B3LYP	LC-wHPBE	M05	mPW1PW91	O3LYP	SVWN	wB97XD	B3LYP
MAE	0.0686084	0.0799468	0.130189	0.0683	0.068422	0.075136	0.09938	0.0837388	0.067562
Slope	0.972357	1.01073	0.959335	0.96694	0.979571	0.961074	0.897687	1.02219	0.972943
Intercept	0.0107767	0.0207836	0.0143494	0.0127655	0.0127067	0.000126788	-0.00780737	0.0212249	0.0110652
R^2	0.878869	0.860592	0.737438	0.87605	0.877689	0.871064	0.856972	0.856349	0.883669
$\|\Delta E\|$	0.3001	0.41503	0.864362	0.188967	0.26924	0.218097	0.405915	0.437403	0.191624
ΔE	-0.128916	-0.0184082	-0.261353	0.00117382	-0.0546574	-0.125882	-0.219048	0.0120256	-0.0480369
E_{comp}/E_{exp}	0.974819	1.00844	0.958238	0.99995	0.988563	0.967944	0.946228	1.01631	0.986974

Improving fit

Table S236: f_{comp} vs nf_{exp} PCM/TD-DFT/6-311++G**/x-gauge/Improved Fit/

	B3P86	CAM-B3LYP	LC-wHPBE	M05	mPW1PW91	O3LYP	SVWN	wB97XD	B3LYP
MAE	0.0383176	0.0572712	0.0861036	0.0433268	0.0406324	0.0448224	0.0349724	0.0609496	0.0345788
Slope	0.98422	1.0762	1.09533	1.02651	1.05081	0.965289	1.02284	1.08451	0.992486
Intercept	0.00655535	0.0153128	0.00038989	-0.00278536	-0.000654662	0.00528018	-0.0127439	0.0185846	0.00385318
R^2	0.966163	0.930895	0.873323	0.954162	0.961122	0.95627	0.981658	0.928647	0.973165
$\|\Delta E\|$	0.238258	0.291472	0.535438	0.222564	0.224039	0.26651	0.334769	0.341024	0.23658
ΔE	0.0560533	0.27152	0.32538	0.0414584	0.0906403	-0.0978213	-0.163524	0.333591	0.0171133
E_{comp}/E_{exp}	1.01544	1.06763	1.08597	1.00905	1.02054	0.972142	0.956824	1.0821	1.00632

Table S237: f_{comp} vs nf_{exp} PCM/TD-DFT/6-311++G**/p-gauge/Improved Fit/

	B3P86	CAM-B3LYP	LC-wHPBE	M05	mPW1PW91	O3LYP	SVWN	wB97XD	B3LYP
MAE	0.0394456	0.0507768	0.0793416	0.0397832	0.0378888	0.0497968	0.034116	0.0542116	0.0356204
Slope	1.05062	1.04793	1.05466	1.01503	1.00662	0.960925	0.993715	1.05324	0.962511
Intercept	-0.0203314	0.0161775	0.00428166	0.00175245	0.00571009	0.00957005	-0.00890758	0.0168691	0.0118202
R^2	0.972425	0.939896	0.879282	0.958591	0.965075	0.920377	0.980467	0.934619	0.973382
$\|\Delta E\|$	0.246801	0.297333	0.569586	0.236056	0.225646	0.264004	0.326474	0.335437	0.248034
ΔE	0.0653791	0.281621	0.359505	0.0669523	0.0927489	-0.0758467	-0.149201	0.327988	0.0269453
E_{comp}/E_{exp}	1.01709	1.06932	1.09349	1.01611	1.02088	0.978101	0.960395	1.0807	1.00838

Table S238: f_{comp} vs nf_{exp} PCM/TD-DFT/6-311++G**/xp-gauge/Improved Fit/

	B3P86	CAM-B3LYP	LC-wHPBE	M05	mPW1PW91	O3LYP	SVWN	wB97XD	B3LYP
MAE	0.0381196	0.0524588	0.0811172	0.0403632	0.0379604	0.056074	0.0356896	0.0571624	0.0342652
Slope	0.965484	1.06578	1.06658	1.01467	1.02615	0.984707	1.00727	1.07114	0.973538
Intercept	0.00833131	0.0161508	0.00580491	0.0039786	0.00429515	-0.000159983	-0.0130125	0.0170146	0.00687837
R^2	0.968021	0.937935	0.874834	0.956756	0.963479	0.913087	0.979863	0.93259	0.974164
$\|\Delta E\|$	0.23942	0.301541	0.570482	0.217534	0.218187	0.26937	0.329667	0.328967	0.240553
ΔE	0.0553757	0.286134	0.360408	0.0494422	0.0969576	-0.0969084	-0.160685	0.314389	0.0198976
E_{comp}/E_{exp}	1.01526	1.07041	1.09378	1.01112	1.02193	0.973585	0.957743	1.07686	1.00681

S3.8 Different Point Groups of symmetry

S3.8.1 Point Group: $C_1 \cap$ VHHM

S3.8.2 f_{exp}

Avg $f_{exp} = 0.382314$

Table S239: f_{comp} vs f_{exp} PCM/TD-DFT/6-311++G**/x-gauge/Exact Band Limits/

	B3P86	CAM-B3LYP	LC-wHPBE	M05	mPW1PW91	O3LYP	SVWN	wB97XD	B3LYP
MAE	0.161526	0.204921	0.235593	0.155214	0.165756	0.156074	0.143302	0.215037	0.159333
Slope	1.35379	1.42062	1.38447	1.32929	1.34333	1.32847	1.15344	1.46481	1.35399
Intercept	0.0042689	0.011525	-0.0592383	0.00895066	0.014574	0.0021626	0.0254661	0.00438749	0.00403813
R^2	0.862557	0.844278	0.674559	0.86102	0.853614	0.842593	0.788982	0.847286	0.868082
$\|\Delta E\|$	0.307458	0.343927	0.926685	0.174558	0.169701	0.2086	0.231924	0.372846	0.178517
ΔE	-0.17023	0.065618	-0.315226	-0.0260204	0.0247343	-0.126191	-0.0413114	0.101031	-0.0637696
E_{comp}/E_{exp}	0.966124	1.02613	0.956415	0.993323	1.00462	0.968275	0.993445	1.03525	0.983357

Table S240: f_{comp} vs f_{exp} PCM/TD-DFT/6-311++G**/p-gauge/Exact Band Limits/

	B3P86	CAM-B3LYP	LC-wHPBE	M05	mPW1PW91	O3LYP	SVWN	wB97XD	B3LYP
MAE	0.144137	0.18677	0.22047	0.154014	0.147874	0.14073	0.13043	0.196791	0.142647
Slope	1.3081	1.36895	1.33274	1.32701	1.29676	1.29042	1.12092	1.41234	1.31175
Intercept	0.00361211	0.0124518	-0.0559032	0.00867031	0.0137253	0.000891961	0.0224459	0.00550074	0.00279083
R^2	0.869509	0.851042	0.678492	0.8679	0.861204	0.851025	0.800348	0.853359	0.874858
$\|\Delta E\|$	0.307263	0.342609	0.925937	0.174378	0.16911	0.208461	0.230579	0.371581	0.178818
ΔE	-0.170074	0.0653072	-0.3158	-0.0261856	0.0245126	-0.124489	-0.0371588	0.10049	-0.0637427
E_{comp}/E_{exp}	0.96621	1.02605	0.956269	0.993307	1.0046	0.968712	0.994386	1.03511	0.983425

Table S241: f_{comp} vs f_{exp} PCM/TD-DFT/6-311++G**/xp-gauge/Exact Band Limits/

	B3P86	CAM-B3LYP	LC-wHPBE	M05	mPW1PW91	O3LYP	SVWN	wB97XD	B3LYP
MAE	0.152474	0.195551	0.227721	0.154233	0.156512	0.148102	0.136691	0.205616	0.150691
Slope	1.33082	1.39467	1.35843	1.3282	1.31995	1.30938	1.13703	1.43845	1.33273
Intercept	0.00372804	0.0118035	-0.0577136	0.00863114	0.0139378	0.00132354	0.0237642	0.00473899	0.00323665
R^2	0.866256	0.84792	0.67673	0.864653	0.857628	0.847002	0.794891	0.850579	0.871641
$\|\Delta E\|$	0.307369	0.343292	0.926349	0.174425	0.169469	0.208571	0.231231	0.372236	0.178701
ΔE	-0.170159	0.0654847	-0.315453	-0.02612	0.0246693	-0.12535	-0.0392944	0.100788	-0.0637408
E_{comp}/E_{exp}	0.966163	1.02609	0.956354	0.993311	1.00462	0.96849	0.993894	1.03519	0.983392

Improving Fit:

Table S242: f_{comp} vs f_{exp} PCM/TD-DFT/6-311++G**/x-gauge/Improved Fit/

	B3P86	CAM-B3LYP	LC-wHPBE	M05	mPW1PW91	O3LYP	SVWN	wB97XD	B3LYP
MAE	0.0756279	0.126598	0.153979	0.0907233	0.079893	0.0659791	0.0450488	0.136481	0.0699372
Slope	1.10601	1.10699	1.1228	1.07286	1.11935	1.14516	1.10643	1.14153	1.09448
Intercept	0.0225058	0.0213978	0.0173793	0.022262	0.0218022	-0.000320527	-0.0228842	0.0217949	0.0212196
R^2	0.886563	0.740637	0.690926	0.798589	0.877558	0.912153	0.929247	0.753025	0.889074
$\|\Delta E\|$	0.278576	0.352918	0.780658	0.259294	0.253031	0.301236	0.298785	0.498655	0.262296
ΔE	0.014622	0.192085	0.128799	-0.0578848	0.119968	-0.100516	-0.0588555	0.0505315	-0.0162855
E_{comp}/E_{exp}	1.00525	1.03734	1.03489	0.977684	1.03125	0.971229	0.98553	1.01473	0.997998

Table S243: f_{comp} vs f_{exp} PCM/TD-DFT/6-311++G**/p-gauge/Improved Fit/

	B3P86	CAM-B3LYP	LC-wHPBE	M05	mPW1PW91	O3LYP	SVWN	wB97XD	B3LYP
MAE	0.0635116	0.116984	0.141153	0.0868233	0.0703884	0.0674023	0.034207	0.123342	0.0591953
Slope	1.07447	1.04577	1.08312	1.14444	1.09384	1.14405	1.08392	1.08295	1.07462
Intercept	0.0213535	0.0453157	0.0172743	0.0186092	0.0237718	-0.00117615	-0.0193336	0.0422429	0.0181216
R^2	0.901062	0.754171	0.702217	0.862233	0.895258	0.858771	0.94754	0.766777	0.907124
$\|\Delta E\|$	0.276568	0.276463	0.778915	0.206937	0.232482	0.295182	0.31578	0.481713	0.265038
ΔE	0.017711	0.259894	0.127384	-0.00481127	0.0919414	-0.0932589	-0.0459059	0.0826202	-0.014533
E_{comp}/E_{exp}	1.00603	1.06363	1.0346	0.998138	1.02495	0.973949	0.989	1.02229	0.998429

Table S244: f_{comp} vs f_{exp} PCM/TD-DFT/6-311++G**/x-gauge/Improved Fit/

	B3P86	CAM-B3LYP	LC-wHPBE	M05	mPW1PW91	O3LYP	SVWN	wB97XD	B3LYP
MAE	0.0693326	0.122774	0.147349	0.0886349	0.0831488	0.0716349	0.0394395	0.129598	0.0639698
Slope	1.09235	1.0632	1.10308	1.14504	1.20238	1.15783	1.10073	1.10199	1.08985
Intercept	0.0210298	0.0457126	0.0178347	0.0193341	-0.0130531	-0.000906956	-0.0234398	0.0426018	0.0191475
R^2	0.894716	0.747139	0.697421	0.855782	0.903052	0.850359	0.937338	0.761783	0.901571
$\|\Delta E\|$	0.278288	0.336521	0.78071	0.206433	0.251002	0.29355	0.300713	0.481582	0.263743
ΔE	0.0154166	0.196994	0.129031	-0.00506523	0.107748	-0.0928683	-0.052206	0.0826588	-0.0144976
E_{comp}/E_{exp}	1.00551	1.0399	1.03492	0.998093	1.02865	0.974	0.98734	1.02229	0.998449

S3.8.3 nf_{exp}

Avg $nf_{exp} = 0.536484$

Table S245: f_{comp} vs nf_{exp} PCM/TD-DFT/6-311++G**/x-gauge/Exact Band Limits/

	B3P86	CAM-B3LYP	LC-wHPBE	M05	mPW1PW91	O3LYP	SVWN	wB97XD	B3LYP
MAE	0.0964912	0.106816	0.189934	0.094566	0.0964577	0.110373	0.144536	0.110225	0.0950219
Slope	0.956306	1.00291	0.978011	0.938879	0.948553	0.936522	0.817095	1.03543	0.956644
Intercept	0.00879924	0.0166003	-0.054622	0.0134623	0.0192656	0.00762487	0.0280834	0.00891523	0.00846451
R^2	0.854562	0.835454	0.668348	0.852825	0.84505	0.831415	0.786119	0.840564	0.860386
$\|\Delta E\|$	0.307458	0.343927	0.926685	0.174558	0.169701	0.2086	0.231924	0.372846	0.178517
ΔE	-0.17023	0.065618	-0.315226	-0.0260204	0.0247343	-0.126191	-0.0413114	0.101031	-0.0637696
E_{comp}/E_{exp}	0.966124	1.02613	0.956415	0.993323	1.00462	0.968275	0.993445	1.03525	0.983357

Table S246: f_{comp} vs nf_{exp} PCM/TD-DFT/6-311++G**/p-gauge/Exact Band Limits/

	B3P86	CAM-B3LYP	LC-wHPBE	M05	mPW1PW91	O3LYP	SVWN	wB97XD	B3LYP
MAE	0.0973381	0.0992405	0.185784	0.0928233	0.0961312	0.108747	0.145313	0.101238	0.0961265
Slope	0.924609	0.967034	0.942253	0.937846	0.916285	0.910232	0.794393	0.998977	0.927403
Intercept	0.00767877	0.0170208	-0.0518804	0.0128653	0.0179211	0.00591251	0.0248118	0.00952339	0.00675632
R^2	0.862529	0.843193	0.673367	0.860696	0.853721	0.840717	0.79811	0.847673	0.868228
$\|\Delta E\|$	0.307263	0.342609	0.925937	0.174378	0.16911	0.208461	0.230579	0.371581	0.178818
ΔE	-0.170074	0.0653072	-0.3158	-0.0261856	0.0245126	-0.124489	-0.0371588	0.10049	-0.0637427
E_{comp}/E_{exp}	0.96621	1.02605	0.956269	0.993307	1.0046	0.968712	0.994386	1.03511	0.983425

Table S247: f_{comp} vs nf_{exp} PCM/TD-DFT/6-311++G**/xp-gauge/Exact Band Limits/

	B3P86	CAM-B3LYP	LC-wHPBE	M05	mPW1PW91	O3LYP	SVWN	wB97XD	B3LYP
MAE	0.0964805	0.101712	0.1871	0.0936195	0.0954079	0.108687	0.144955	0.105084	0.0953158
Slope	0.940375	0.984904	0.960017	0.938396	0.932356	0.923333	0.805634	1.01711	0.941926
Intercept	0.00802504	0.0166222	-0.0533987	0.0129866	0.0183805	0.00656304	0.0262556	0.00901231	0.00742998
R^2	0.858759	0.839578	0.671059	0.856943	0.849592	0.836252	0.792332	0.844366	0.864473
$\|\Delta E\|$	0.307369	0.343292	0.926349	0.174425	0.169469	0.208571	0.231231	0.372236	0.178701
ΔE	-0.170159	0.0654847	-0.315453	-0.02612	0.0246693	-0.12535	-0.0392944	0.100788	-0.0637408
E_{comp}/E_{exp}	0.966163	1.02609	0.956354	0.993311	1.00462	0.96849	0.993894	1.03519	0.983392

Improved fit:

Table S248: f_{comp} vs nf_{exp} PCM/TD-DFT/6-311++G**/x-gauge/Improved Fit/

	B3P86	CAM-B3LYP	LC-wHPBE	M05	mPW1PW91	O3LYP	SVWN	wB97XD	B3LYP
MAE	0.0428488	0.0628228	0.0988284	0.0486116	0.0444381	0.0560479	0.0461698	0.0683749	0.0384642
Slope	0.985846	1.05952	1.07716	1.01426	1.02756	0.936917	0.981816	1.05087	0.982204
Intercept	-0.00061136	0.00765582	-0.0173883	-0.00568077	-0.00309025	0.0068637	-0.0114027	0.0139568	0.000517239
R^2	0.965152	0.923448	0.853446	0.951964	0.956423	0.946875	0.964951	0.912999	0.971466
$\|\Delta E\|$	0.236749	0.296005	0.510823	0.213662	0.220478	0.242278	0.291376	0.358595	0.232464
ΔE	0.0657132	0.282966	0.485841	0.0347691	0.0970717	-0.0833379	-0.131404	0.356679	0.0299795
E_{comp}/E_{exp}	1.01781	1.06844	1.11705	1.00629	1.02011	0.975769	0.965447	1.08589	1.00956

Table S249: f_{comp} vs nf_{exp} PCM/TD-DFT/6-311++G**/p-gauge/Improved Fit/

	B3P86	CAM-B3LYP	LC-wHPBE	M05	mPW1PW91	O3LYP	SVWN	wB97XD	B3LYP
MAE	0.0461005	0.0552526	0.0909567	0.0424158	0.0440526	0.0652516	0.04506	0.061253	0.042634
Slope	1.04605	1.04166	1.06685	1.00539	0.997205	0.933958	0.954097	1.04588	0.955474
Intercept	-0.033907	0.00425799	-0.0194375	-5.05767e-05	-0.00028443	0.0128563	-0.000643535	0.0122249	0.00509436
R^2	0.969473	0.936418	0.868316	0.958405	0.962938	0.907152	0.965324	0.930522	0.97235
$\|\Delta E\|$	0.250044	0.314202	0.650264	0.207397	0.219111	0.239614	0.296488	0.360378	0.233372
ΔE	0.0901964	0.306486	0.416481	0.045628	0.104917	-0.0410917	-0.117504	0.358137	0.0415852
E_{comp}/E_{exp}	1.02262	1.0729	1.10107	1.00923	1.022	0.986161	0.968738	1.08571	1.01217

Table S250: f_{comp} vs nf_{exp} PCM/TD-DFT/6-311++G**/xp-gauge/Improved Fit/

	B3P86	CAM-B3LYP	LC-wHPBE	M05	mPW1PW91	O3LYP	SVWN	wB97XD	B3LYP
MAE	0.0428256	0.0574819	0.0960535	0.0440274	0.04278	0.0693809	0.0467051	0.0645047	0.0394098
Slope	0.969727	1.05191	1.07693	1.00646	1.00919	0.957648	0.967634	1.0612	0.966547
Intercept	0.000761668	0.00849541	-0.0209073	-4.4347e-05	0.00155148	0.00162838	-0.0068806	0.0050604	0.00326081
R^2	0.967117	0.932232	0.861607	0.95632	0.960266	0.901192	0.965246	0.924133	0.972732
$\|\Delta E\|$	0.244893	0.314526	0.634563	0.204983	0.21693	0.248897	0.298696	0.353334	0.236042
ΔE	0.0741363	0.306772	0.40078	0.0468827	0.112547	-0.0611518	-0.136461	0.351295	0.0379702
E_{comp}/E_{exp}	1.01946	1.07295	1.09796	1.00941	1.02372	0.982028	0.964304	1.08448	1.01121

S3.8.4 Point Group: $C_s \cap$ VHHM

S3.8.5 f_{exp}

Avg f_{exp} = 0.177228

Table S251: f_{comp} vs f_{exp} PCM/TD-DFT/6-311++G**/x-gauge/Exact Band Limits/

	B3P86	CAM-B3LYP	LC-wHPBE	M05	mPW1PW91	O3LYP	SVWN	wB97XD	B3LYP
MAE	0.0663283	0.0901317	0.120235	0.0618617	0.0736183	0.052825	0.103338	0.091495	0.0652417
Slope	1.30356	1.3495	1.44111	1.2547	1.35306	1.26417	1.15372	1.36163	1.3035
Intercept	0.00573286	0.0224871	0.00972481	0.00984853	0.00381888	-0.00505946	0.00774229	0.021867	0.00523639
R^2	0.962877	0.954101	0.869901	0.959293	0.965342	0.960887	0.660601	0.956564	0.965079
$\|\Delta E\|$	0.202217	0.37058	0.789852	0.206739	0.330125	0.250577	0.405012	0.387886	0.210117
ΔE	-0.0682508	0.00581506	-0.33158	-0.0569065	-0.16382	-0.188939	-0.173781	0.020649	-0.0981863
E_{comp}/E_{exp}	0.982838	1.00599	0.918528	0.987812	0.96425	0.954696	0.959517	1.00916	0.976116

Table S252: f_{comp} vs f_{exp} PCM/TD-DFT/6-311++G**/p-gauge/Exact Band Limits/

	B3P86	CAM-B3LYP	LC-wHPBE	M05	mPW1PW91	O3LYP	SVWN	wB97XD	B3LYP
MAE	0.060065	0.083755	0.112115	0.0628317	0.0671517	0.048625	0.095095	0.0850583	0.0594517
Slope	1.23108	1.26654	1.34275	1.22493	1.28107	1.20558	1.10774	1.27569	1.23567
Intercept	0.012091	0.0305262	0.0193897	0.0163207	0.00973882	-0.000126532	0.0082733	0.030301	0.0113008
R^2	0.960478	0.948033	0.854586	0.955454	0.962484	0.958947	0.667788	0.949593	0.963174
$\|\Delta E\|$	0.204999	0.367279	0.789183	0.207545	0.330582	0.255087	0.510969	0.384664	0.212827
ΔE	-0.0727279	0.00263987	-0.333658	-0.0603496	-0.168083	-0.190787	-0.307444	0.0175778	-0.10254
E_{comp}/E_{exp}	0.981619	1.00513	0.918047	0.986858	0.963085	0.954197	0.922628	1.00834	0.974911

Table S253: f_{comp} vs f_{exp} PCM/TD-DFT/6-311++G**/xp-gauge/Exact Band Limits/

	B3P86	CAM-B3LYP	LC-wHPBE	M05	mPW1PW91	O3LYP	SVWN	wB97XD	B3LYP
MAE	0.063085	0.0867317	0.115975	0.0622517	0.0702417	0.050335	0.0990983	0.0880217	0.0622317
Slope	1.26715	1.30791	1.39145	1.24006	1.31671	1.2345	1.1304	1.31839	1.26938
Intercept	0.00876771	0.0263306	0.014425	0.0129056	0.00665114	-0.00268518	0.00786051	0.0259306	0.00813385
R^2	0.962209	0.952028	0.863482	0.957898	0.96448	0.960221	0.664447	0.954064	0.964617
$\|\Delta E\|$	0.203752	0.368879	0.78952	0.206955	0.330442	0.252313	0.51211	0.386216	0.211566
ΔE	-0.0706325	0.0041585	-0.33263	-0.0586909	-0.166026	-0.189837	-0.307129	0.0190498	-0.100475
E_{comp}/E_{exp}	0.982189	1.00554	0.918285	0.987317	0.963648	0.954455	0.922683	1.00873	0.975483

Improving Fit:

Table S254: f_{comp} vs f_{exp} PCM/TD-DFT/6-311++G**/x-gauge/Improved Fit/

	B3P86	CAM-B3LYP	LC-wHPBE	M05	mPW1PW91	O3LYP	SVWN	wB97XD	B3LYP
MAE	0.0572917	0.0771417	0.0870117	0.0528283	0.0627217	0.0389817	0.0359817	0.0816183	0.0577217
Slope	1.27949	1.33269	1.42804	1.22476	1.30367	1.09642	1.15586	1.343	1.28185
Intercept	0.00216401	0.00809912	-0.0226755	0.00545769	0.00247875	0.0102933	-0.00684387	0.0043632	-0.00571954
R^2	0.97403	0.946248	0.893873	0.968586	0.971975	0.95341	0.951355	0.934703	0.961857
$\|\Delta E\|$	0.268809	0.358875	0.808615	0.245758	0.385395	0.341408	0.565208	0.38091	0.281516
ΔE	-0.00317614	-0.0238454	-0.277476	-0.0197487	-0.112155	-0.132937	-0.416594	-0.001874	-0.0452519
E_{comp}/E_{exp}	1.00108	0.997604	0.923349	0.998378	0.978966	0.970061	0.905147	1.00297	0.993367

Table S255: f_{comp} vs f_{exp} PCM/TD-DFT/6-311++G**/p-gauge/Improved Fit/

	B3P86	CAM-B3LYP	LC-wHPBE	M05	mPW1PW91	O3LYP	SVWN	wB97XD	B3LYP
MAE	0.049745	0.0694283	0.0783417	0.0522517	0.0552817	0.0357583	0.031175	0.0745883	0.051345
Slope	1.21323	1.25047	1.3225	1.19466	1.23209	1.04732	1.09796	1.26203	1.21684
Intercept	0.00523395	0.0151154	-0.0107904	0.00794576	0.003889	0.012591	-0.00207214	0.00882203	-0.00101468
R^2	0.974134	0.949545	0.894408	0.96288	0.966102	0.953279	0.957019	0.928547	0.962263
$\|\Delta E\|$	0.27182	0.368742	0.692831	0.254518	0.39166	0.334318	0.562219	0.404963	0.281338
ΔE	-0.0445402	0.000168638	-0.148372	-0.0250964	-0.123529	-0.145793	-0.413575	0.0030171	-0.0504967
E_{comp}/E_{exp}	0.989423	1.00434	0.958491	0.997066	0.976011	0.96716	0.905629	1.00637	0.991947

Table S256: f_{comp} vs f_{exp} PCM/TD-DFT/6-311++G**/xp-gauge/Improved Fit/

	B3P86	CAM-B3LYP	LC-wHPBE	M05	mPW1PW91	O3LYP	SVWN	wB97XD	B3LYP
MAE	0.0536717	0.0730617	0.0814183	0.0529417	0.0590383	0.0370717	0.0336483	0.0772417	0.0524917
Slope	1.24831	1.29095	1.36688	1.21034	1.26799	1.07198	1.12654	1.30041	1.24441
Intercept	0.0024149	0.0116862	-0.0134141	0.00832072	0.00467284	0.011245	-0.00447526	0.00754006	-0.000661128
R^2	0.973299	0.948394	0.897683	0.969414	0.972895	0.953537	0.954397	0.935539	0.971476
$\|\Delta E\|$	0.271572	0.358907	0.687247	0.247185	0.385376	0.333032	0.562275	0.392338	0.264478
ΔE	-0.0425836	-0.0238133	-0.128716	-0.0194735	-0.114803	-0.144507	-0.41363	0.0214328	-0.0312531
E_{comp}/E_{exp}	0.989962	0.997593	0.96189	0.998421	0.978236	0.967497	0.90562	1.00956	0.995503

S3.8.6 nf_{exp}

Avg $nf_{exp} = 0.249499$

Table S257: f_{comp} vs nf_{exp} PCM/TD-DFT/6-311++G**/x-gauge/Exact Band Limits/

	B3P86	CAM-B3LYP	LC-wHPBE	M05	mPW1PW91	O3LYP	SVWN	wB97XD	B3LYP
MAE	0.04189	0.0448473	0.0772193	0.0427933	0.0382307	0.0478273	0.103071	0.0446773	0.04147
Slope	0.904027	0.936311	1.0002	0.870067	0.939221	0.876881	0.79218	0.944664	0.904277
Intercept	0.0112059	0.0280477	0.0155798	0.0151355	0.00928492	0.000205453	0.014565	0.0274935	0.0106367
R^2	0.953396	0.94556	0.862687	0.949681	0.957592	0.951797	0.641194	0.947872	0.956191
$\|\Delta E\|$	0.202217	0.37058	0.789852	0.206739	0.330125	0.250577	0.405012	0.387886	0.210117
ΔE	-0.0682508	0.00581506	-0.33158	-0.0569065	-0.16382	-0.188939	-0.173781	0.020649	-0.0981863
E_{comp}/E_{exp}	0.982838	1.00599	0.918528	0.987812	0.96425	0.954696	0.959517	1.00916	0.976116

Table S258: f_{comp} vs nf_{exp} PCM/TD-DFT/6-311++G**/p-gauge/Exact Band Limits/

	B3P86	CAM-B3LYP	LC-wHPBE	M05	mPW1PW91	O3LYP	SVWN	wB97XD	B3LYP
MAE	0.0445927	0.0460747	0.078354	0.0453127	0.0403227	0.050312	0.0995573	0.0461967	0.0440227
Slope	0.853934	0.878761	0.931666	0.849529	0.88949	0.836539	0.760724	0.884982	0.857437
Intercept	0.0172174	0.0357432	0.0249134	0.0214563	0.0148528	0.00482072	0.0147965	0.0355875	0.0163668
R^2	0.951399	0.939561	0.847001	0.946112	0.955284	0.950547	0.648357	0.940836	0.954779
$\|\Delta E\|$	0.204999	0.367279	0.789183	0.207545	0.330582	0.255087	0.510969	0.384664	0.212827
ΔE	-0.0727279	0.00263987	-0.333658	-0.0603496	-0.168083	-0.190787	-0.307444	0.0175778	-0.10254
E_{comp}/E_{exp}	0.981619	1.00513	0.918047	0.986858	0.963085	0.954197	0.922628	1.00834	0.974911

Table S259: f_{comp} vs nf_{exp} PCM/TD-DFT/6-311++G**/xp-gauge/Exact Band Limits/

	B3P86	CAM-B3LYP	LC-wHPBE	M05	mPW1PW91	O3LYP	SVWN	wB97XD	B3LYP
MAE	0.043216	0.0452673	0.077226	0.0439327	0.038826	0.0490593	0.100914	0.0452573	0.0427227
Slope	0.878871	0.907469	0.965608	0.859974	0.914141	0.856464	0.776269	0.914641	0.880717
Intercept	0.0140657	0.0317171	0.0201115	0.018117	0.0119392	0.00242278	0.0145414	0.0313843	0.0133651
R^2	0.952928	0.943527	0.856085	0.948423	0.957007	0.951489	0.645094	0.945346	0.955974
$\|\Delta E\|$	0.203752	0.368879	0.78952	0.206955	0.330447	0.252288	0.512146	0.386216	0.211566
ΔE	-0.0706325	0.0041585	-0.33263	-0.0586909	-0.166031	-0.189811	-0.307165	0.0190498	-0.100475
E_{comp}/E_{exp}	0.982189	1.00554	0.918285	0.987317	0.963647	0.95446	0.922676	1.00873	0.975483

Improving Fit:

Table S260: f_{comp} vs nf_{exp} PCM/TD-DFT/6-311++G**/x-gauge/Improved Fit/

	B3P86	CAM-B3LYP	LC-wHPBE	M05	mPW1PW91	O3LYP	SVWN	wB97XD	B3LYP
MAE	0.0312627	0.0356033	0.053286	0.0301713	0.028918	0.0275387	0.0338147	0.0364033	0.031236
Slope	0.969122	0.973249	1.0033	0.992647	0.982895	1.04923	0.968031	0.980321	0.967555
Intercept	-0.000692021	0.0201349	0.00932488	-0.00359809	0.00205163	-0.0149484	0.000260142	0.0188371	-0.0011476
R^2	0.972005	0.965604	0.914133	0.96828	0.974542	0.979929	0.962852	0.964541	0.971458
$\|\Delta E\|$	0.193104	0.247735	0.518635	0.188457	0.195215	0.232693	0.350564	0.26034	0.196787
ΔE	-0.0345336	0.203778	0.168539	-0.00911549	0.0319075	-0.130529	-0.162697	0.213394	-0.0688664
E_{comp}/E_{exp}	0.989751	1.04866	1.04594	0.997517	1.00642	0.965701	0.959054	1.05097	0.982317

Table S261: f_{comp} vs nf_{exp} PCM/TD-DFT/6-311++G**/p-gauge/Improved Fit/

	B3P86	CAM-B3LYP	LC-wHPBE	M05	mPW1PW91	O3LYP	SVWN	wB97XD	B3LYP
MAE	0.0307573	0.0331387	0.0532653	0.03075	0.0275307	0.0228627	0.0362	0.035592	0.03072
Slope	0.995406	0.958768	0.95261	0.990025	0.963047	1.00909	0.94989	0.985241	1.04863
Intercept	-0.00462649	0.0192647	0.0130678	-0.00499391	0.00484044	-0.00771807	-0.00256023	0.0112165	-0.00903819
R^2	0.970948	0.967577	0.91912	0.968789	0.974298	0.984378	0.956232	0.96399	0.974122
$\|\Delta E\|$	0.195458	0.249169	0.519726	0.212477	0.200333	0.248149	0.334483	0.263471	0.203291
ΔE	-0.0148312	0.207484	0.169591	0.0248028	0.0454725	-0.115678	-0.159558	0.218236	-0.0390867
E_{comp}/E_{exp}	0.993094	1.04868	1.0459	1.00668	1.00853	0.968991	0.959552	1.05111	0.987972

Table S262: f_{comp} vs nf_{exp} PCM/TD-DFT/6-311++G**/xp-gauge/Improved Fit/

	B3P86	CAM-B3LYP	LC-wHPBE	M05	mPW1PW91	O3LYP	SVWN	wB97XD	B3LYP
MAE	0.0302713	0.03481	0.051406	0.030884	0.0288187	0.0259693	0.03515	0.036008	0.0302813
Slope	0.958706	0.984589	0.981911	0.98306	0.983263	1.02888	0.957244	0.952701	1.007
Intercept	0.00125011	0.0164223	0.0111306	-0.00100279	0.00164318	-0.013431	-0.00343829	0.0218016	-0.00550633
R^2	0.972469	0.968158	0.918727	0.967149	0.974407	0.98036	0.955866	0.964746	0.971621
$\|\Delta E\|$	0.201196	0.252135	0.521516	0.188631	0.206103	0.232433	0.342598	0.243851	0.189641
ΔE	-0.027372	0.211005	0.171394	-0.0104353	0.0403729	-0.132415	-0.166057	0.185393	-0.052275
E_{comp}/E_{exp}	0.990762	1.04979	1.04645	0.997202	1.00763	0.965208	0.958613	1.043	0.985136

S3.8.7 Point Group: Higher than C_s (D_{2h}, C_{2h}, D_2, C_{2v}, and C_2) $\cap$ VHHM

S3.8.8 f_{exp}

Avg f_{exp} = 0.366192

Table S263: f_{comp} vs f_{exp} PCM/TD-DFT/6-311++G**/x-gauge/Exact Band Limits/

	B3P86	CAM-B3LYP	LC-wHPBE	M05	mPW1PW91	O3LYP	SVWN	wB97XD	B3LYP
MAE	0.142975	0.18665	0.1961	0.143292	0.137025	0.134475	0.138792	0.1986	0.1385
Slope	1.3092	1.38596	1.50732	1.35306	1.24473	1.2247	1.15451	1.47243	1.30343
Intercept	0.00760511	0.00379731	-0.0282165	-0.00130364	0.0358151	0.0268817	-0.0112625	-0.0159157	0.00674495
R^2	0.91463	0.89082	0.936294	0.929751	0.910183	0.877997	0.695285	0.900478	0.920176
$\|\Delta E\|$	0.285486	0.634822	0.661705	0.258301	0.24096	0.335	0.786585	0.649104	0.297685
ΔE	-0.111817	-0.355105	-0.180957	-0.11474	-0.0494572	-0.209802	-0.63097	-0.328246	-0.150257
E_{comp}/E_{exp}	0.977373	0.939147	0.961255	0.976603	0.990627	0.956779	0.87625	0.945174	0.968972

Table S264: f_{comp} vs f_{exp} PCM/TD-DFT/6-311++G**/p-gauge/Exact Band Limits/

	B3P86	CAM-B3LYP	LC-wHPBE	M05	mPW1PW91	O3LYP	SVWN	wB97XD	B3LYP
MAE	0.1372	0.17155	0.178508	0.149883	0.131275	0.129017	0.130425	0.185217	0.133083
Slope	1.27313	1.34261	1.4623	1.36146	1.20929	1.19495	1.12853	1.42878	1.27009
Intercept	0.00658939	0.00457186	-0.0288741	-0.00228749	0.03421	0.0254681	-0.0123667	-0.0149745	0.005369
R^2	0.904791	0.88436	0.935334	0.921512	0.902615	0.867797	0.700607	0.892217	0.911126
$\|\Delta E\|$	0.285685	0.634137	0.661992	0.258685	0.241238	0.334253	0.787999	0.648395	0.298391
ΔE	-0.112259	-0.354619	-0.18067	-0.115124	-0.0497375	-0.209031	-0.634712	-0.327782	-0.150963
E_{comp}/E_{exp}	0.977307	0.939276	0.961334	0.976531	0.99058	0.956957	0.87547	0.945298	0.968831

Table S265: f_{comp} vs f_{exp} PCM/TD-DFT/6-311++G**/xp-gauge/Exact Band Limits/

	B3P86	CAM-B3LYP	LC-wHPBE	M05	mPW1PW91	O3LYP	SVWN	wB97XD	B3LYP
MAE	0.140083	0.178942	0.187117	0.146567	0.134125	0.131692	0.134467	0.190883	0.13575
Slope	1.29097	1.36384	1.48449	1.35715	1.22668	1.2097	1.14143	1.45019	1.28651
Intercept	0.00697571	0.00418989	-0.0286089	-0.00189293	0.0349428	0.0260444	-0.0119325	-0.01549	0.00592569
R^2	0.909887	0.887894	0.936309	0.925758	0.906653	0.873016	0.698234	0.896641	0.915803
$\|\Delta E\|$	0.285626	0.634485	0.661848	0.258535	0.241114	0.334739	0.787135	0.648737	0.298096
ΔE	-0.112079	-0.354868	-0.180814	-0.114974	-0.0496191	-0.209463	-0.6331	-0.328002	-0.150668
E_{comp}/E_{exp}	0.977332	0.93921	0.961294	0.976558	0.9906	0.956858	0.875801	0.945238	0.968889

Improving Fit:

Table S266: f_{comp} vs f_{exp} PCM/TD-DFT/6-311++G**/x-gauge/Improved Fit/

	B3P86	CAM-B3LYP	LC-wHPBE	M05	mPW1PW91	O3LYP	SVWN	wB97XD	B3LYP
MAE	0.0702083	0.0982583	0.125008	0.073275	0.067825	0.062525	0.0992917	0.11065	0.0770833
Slope	1.0615	0.967686	1.0652	1.03962	1.03764	1.01662	0.843594	1.1814	1.14952
Intercept	0.0169448	0.0814333	0.0459479	0.0136148	0.0325402	0.0313872	0.00329971	0.0246301	-0.00347061
R^2	0.876365	0.824278	0.793279	0.870086	0.868334	0.882472	0.635042	0.893904	0.905164
$\|\Delta E\|$	0.333578	0.298666	0.719506	0.607595	0.288906	0.673742	0.786854	0.397567	0.31848
ΔE	0.019516	0.232488	-0.123155	-0.425146	0.0675348	-0.418288	-0.484854	0.334229	-0.0238684
E_{comp}/E_{exp}	1.00527	1.05079	0.974166	0.904989	1.01634	0.905998	0.905906	1.07327	0.996877

Table S267: f_{comp} vs f_{exp} PCM/TD-DFT/6-311++G**/p-gauge/Improved Fit/

	B3P86	CAM-B3LYP	LC-wHPBE	M05	mPW1PW91	O3LYP	SVWN	wB97XD	B3LYP
MAE	0.06805	0.0870583	0.1073	0.07225	0.0626833	0.0588833	0.0987333	0.097	0.07185
Slope	1.01596	0.938477	1.0314	1.03342	1.04532	0.996922	0.853186	1.15105	1.1185
Intercept	0.0185317	0.0764126	0.0444499	0.0142438	0.0194859	0.0250937	-0.00341279	0.0209703	-0.00485125
R^2	0.876219	0.838379	0.819936	0.87612	0.890115	0.884585	0.64264	0.900257	0.907135
$\|\Delta E\|$	0.312147	0.297895	0.655147	0.259501	0.285967	0.663011	0.767624	0.397048	0.321185
ΔE	-0.00806536	0.231717	-0.187515	-0.043712	0.0643544	-0.442295	-0.500291	0.333779	-0.021883
E_{comp}/E_{exp}	1.0008	1.05062	0.95944	0.992113	1.01551	0.901836	0.90337	1.07317	0.997329

Table S268: f_{comp} vs f_{exp} PCM/TD-DFT/6-311++G**/xp-gauge/Improved Fit/

	B3P86	CAM-B3LYP	LC-wHPBE	M05	mPW1PW91	O3LYP	SVWN	wB97XD	B3LYP
MAE	0.0678167	0.092075	0.117333	0.0726833	0.065575	0.0613917	0.098725	0.102892	0.0744917
Slope	1.04707	0.952721	1.04625	1.03503	1.06055	0.993014	0.849469	1.16589	1.13384
Intercept	0.0154044	0.0788798	0.0444813	0.0139724	0.0209372	0.029558	-0.000126803	0.0227025	-0.00432921
R^2	0.879481	0.831765	0.804774	0.872585	0.88689	0.878357	0.638894	0.89747	0.906359
$\|\Delta E\|$	0.334457	0.298274	0.719128	0.607198	0.287791	0.673877	0.785628	0.396659	0.32004
ΔE	0.0202213	0.232096	-0.123534	-0.425775	0.0662508	-0.411401	-0.482646	0.33332	-0.0227182
E_{comp}/E_{exp}	1.00542	1.05071	0.974079	0.904868	1.01594	0.907289	0.906271	1.07307	0.99714

S3.8.9 nf_{exp}

Avg nf_{exp} = 0.522867

Table S269: f_{comp} vs nf_{exp} PCM/TD-DFT/6-311++G**/x-gauge/Exact Band Limits/

	B3P86	CAM-B3LYP	LC-wHPBE	M05	mPW1PW91	O3LYP	SVWN	wB97XD	B3LYP
MAE	0.0948392	0.106181	0.0757008	0.0872558	0.101089	0.112589	0.152042	0.102781	0.0907808
Slope	0.88316	0.933193	1.01756	0.91509	0.837935	0.825791	0.777348	0.99316	0.879789
Intercept	0.0252491	0.0233888	-0.0082992	0.0157042	0.0534961	0.0435788	0.0050582	0.00398382	0.0240369
R^2	0.902034	0.875274	0.924779	0.921669	0.893949	0.865136	0.683148	0.887887	0.908587
$\|\Delta E\|$	0.285486	0.634822	0.661705	0.258301	0.24096	0.335	0.786585	0.649104	0.297685
ΔE	-0.111817	-0.355105	-0.180957	-0.11474	-0.0494572	-0.209802	-0.63097	-0.328246	-0.150257
E_{comp}/E_{exp}	0.977373	0.939147	0.961255	0.976603	0.990627	0.956779	0.87625	0.945174	0.968972

Table S270: f_{comp} vs nf_{exp} PCM/TD-DFT/6-311++G**/p-gauge/Exact Band Limits/

	B3P86	CAM-B3LYP	LC-wHPBE	M05	mPW1PW91	O3LYP	SVWN	wB97XD	B3LYP
MAE	0.0972142	0.106881	0.0784592	0.0893142	0.102723	0.113581	0.155809	0.103472	0.0929142
Slope	0.859355	0.904403	0.987864	0.921336	0.814614	0.806246	0.760511	0.964163	0.857804
Intercept	0.0234714	0.0233423	-0.00991372	0.0145302	0.0511067	0.0414902	0.00324527	0.00410362	0.0219488
R^2	0.893428	0.869692	0.925128	0.914624	0.887685	0.85618	0.689561	0.88055	0.900735
$\|\Delta E\|$	0.285685	0.634137	0.661992	0.258685	0.241238	0.334253	0.787999	0.648395	0.298391
ΔE	-0.112259	-0.354619	-0.18067	-0.115124	-0.0497375	-0.209031	-0.634712	-0.327782	-0.150963
E_{comp}/E_{exp}	0.977307	0.939276	0.961334	0.976531	0.99058	0.956957	0.87547	0.945298	0.968831

Table S271: f_{comp} vs nf_{exp} PCM/TD-DFT/6-311++G**/xp-gauge/Exact Band Limits/

	B3P86	CAM-B3LYP	LC-wHPBE	M05	mPW1PW91	O3LYP	SVWN	wB97XD	B3LYP
MAE	0.0960475	0.106556	0.0771008	0.0883308	0.101956	0.113106	0.153968	0.103131	0.0918808
Slope	0.871127	0.918497	1.00251	0.918137	0.826054	0.815931	0.76888	0.978386	0.868627
Intercept	0.0242325	0.0233647	-0.00917747	0.0150194	0.052225	0.0424012	0.00402757	0.00399197	0.0228565
R^2	0.897914	0.872777	0.925444	0.918272	0.891069	0.860777	0.686646	0.884508	0.904814
$\|\Delta E\|$	0.285626	0.634485	0.661848	0.258535	0.241114	0.334739	0.787135	0.648737	0.298096
ΔE	-0.112079	-0.354868	-0.180814	-0.114974	-0.0496191	-0.209463	-0.6331	-0.328002	-0.150668
E_{comp}/E_{exp}	0.977332	0.93921	0.961294	0.976558	0.9906	0.956858	0.875801	0.945238	0.968889

Improved Fit:

Table S272: f_{comp} vs nf_{exp} PCM/TD-DFT/6-311++G**/x-gauge/Improved Fit/

	B3P86	CAM-B3LYP	LC-wHPBE	M05	mPW1PW91	O3LYP	SVWN	wB97XD	B3LYP
MAE	0.0613008	0.0497675	0.0686458	0.0615558	0.0636308	0.0807858	0.105866	0.0545858	0.0614125
Slope	0.973709	0.969669	1.03869	0.969109	0.968349	0.819473	0.737907	0.967124	0.949029
Intercept	-0.00362089	0.0349251	-0.0160318	-0.00692382	-0.00249346	0.0415323	0.0429472	0.0373641	0.00481704
R^2	0.929411	0.970848	0.931508	0.940906	0.94031	0.902663	0.699405	0.965331	0.931179
$\|\Delta E\|$	0.259136	0.224992	0.398124	0.259936	0.254915	0.312639	0.795179	0.238775	0.262379
ΔE	-0.0616862	0.130371	0.315899	-0.106868	-0.0432515	-0.177661	-0.611455	0.153623	-0.104027
E_{comp}/E_{exp}	0.986974	1.02725	1.06967	0.977744	0.991223	0.962893	0.878323	1.03262	0.977919

Table S273: f_{comp} vs nf_{exp} PCM/TD-DFT/6-311++G**/p-gauge/Improved Fit/

	B3P86	CAM-B3LYP	LC-wHPBE	M05	mPW1PW91	O3LYP	SVWN	wB97XD	B3LYP
MAE	0.0597992	0.0490725	0.0682925	0.0633242	0.0616958	0.0831358	0.107651	0.0556075	0.0614658
Slope	0.955819	0.950909	1.00864	0.958702	0.951189	0.800945	0.742929	0.9411	0.927742
Intercept	-0.00929168	0.029817	-0.0189765	-0.00465768	0.00289575	0.0377701	0.0421134	0.0380377	0.00138904
R^2	0.920448	0.963351	0.931819	0.933426	0.930382	0.89533	0.692482	0.957265	0.922229
$\|\Delta E\|$	0.257782	0.226272	0.673164	0.262218	0.240134	0.313853	0.804809	0.242015	0.264453
ΔE	-0.0637572	0.130057	-0.169497	-0.109427	-0.0022827	-0.178875	-0.608321	0.156741	-0.105892
E_{comp}/E_{exp}	0.986573	1.02718	0.963325	0.977189	0.999534	0.962657	0.879743	1.03327	0.977545

Table S274: f_{comp} vs nf_{exp} PCM/TD-DFT/6-311++G**/xp-gauge/Improved Fit/

	B3P86	CAM-B3LYP	LC-wHPBE	M05	mPW1PW91	O3LYP	SVWN	wB97XD	B3LYP
MAE	0.0594242	0.0487975	0.0671042	0.0626658	0.0636592	0.0820192	0.108592	0.0555492	0.0598325
Slope	0.962186	0.957849	1.02278	0.962167	0.967835	0.810194	0.730322	0.953845	0.938281
Intercept	-0.00570389	0.0330716	-0.0172429	-0.00662767	-0.00819941	0.039451	0.0438134	0.0363906	0.00294483
R^2	0.924997	0.967452	0.932028	0.937598	0.936723	0.899184	0.698622	0.961398	0.926851
$\|\Delta E\|$	0.25792	0.225429	0.397121	0.262083	0.256292	0.313365	0.804064	0.238813	0.263757
ΔE	-0.0626092	0.129642	0.314896	-0.110629	-0.0425938	-0.178388	-0.608343	0.153348	-0.105293
E_{comp}/E_{exp}	0.98679	1.02712	1.06947	0.976965	0.991321	0.96275	0.879602	1.03257	0.977665

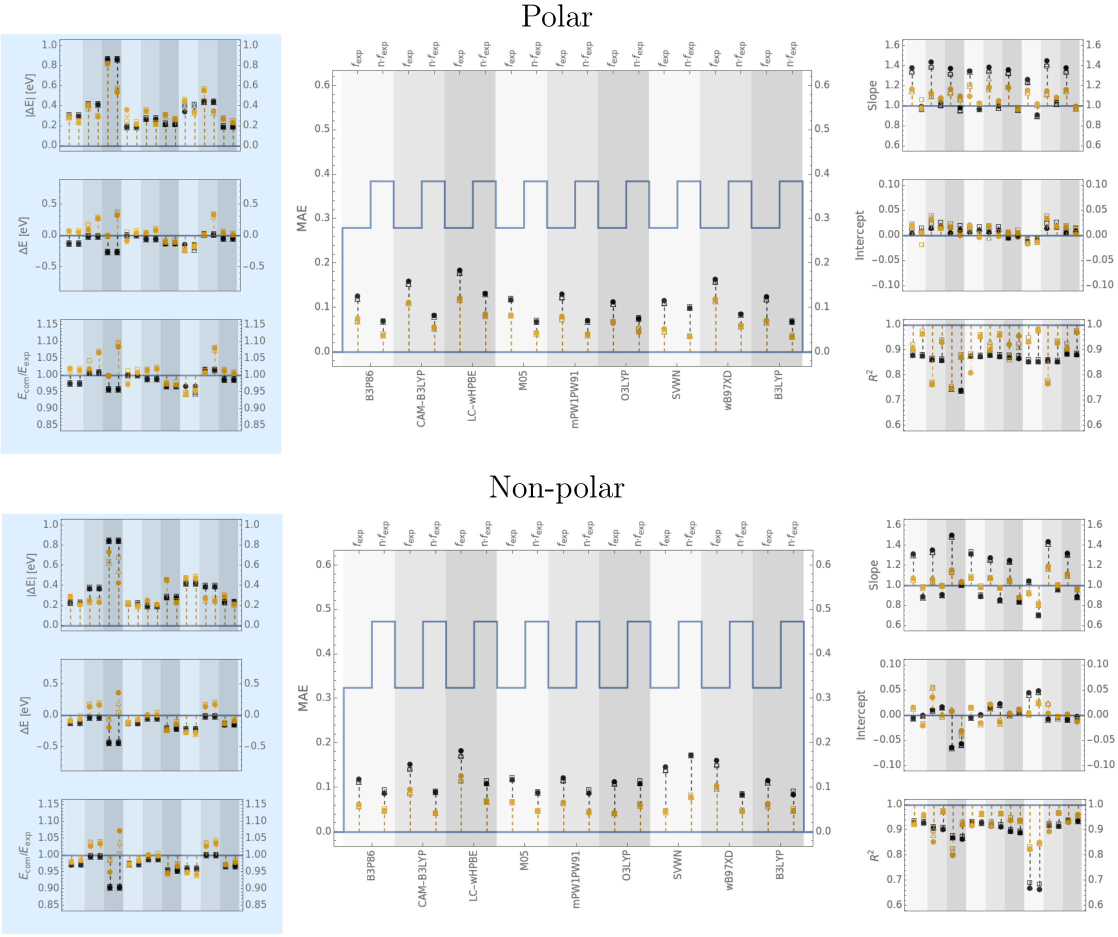

Figure S4: Separating the VHHM transitions into two subsets (Polar or Non Polar) according to the solvent in which the experimental data was obtained

S3.9 TDA/TD-DFT/PCM: VHHM transitions

S3.9.1 f_{exp} VHHM transitions

Avg $f_{exp} = 0.307655$

Table S275: f_{comp} vs f_{exp} PCM/TD-DFT/TDA/6-311++G**/x-gauge/Exact Band Limits/

	B3P86	CAM-B3LYP	LC-wHPBE	M05	mPW1PW91	O3LYP	SVWN	wB97XD	B3LYP
MAE	0.218451	0.239776	0.247064	0.214183	0.221829	0.20629	0.229084	0.238431	0.211997
Slope	1.68061	1.53675	1.09806	1.68385	1.62585	1.67634	1.72739	1.42812	1.6541
Intercept	-0.00973909	0.0222703	0.0335478	-0.0127576	0.00247059	-0.0102551	-0.0221611	0.044657	-0.0044078
R^2	0.816383	0.762884	0.419882	0.852388	0.824796	0.823599	0.754733	0.69971	0.819391
$\|\Delta E\|$	0.444154	0.813286	1.03648	0.258337	0.426524	0.268282	0.315795	0.882524	0.318475
ΔE	-0.170306	-0.288467	-0.401749	0.0235285	-0.126664	-0.0882073	-0.169291	-0.322294	-0.0774471
E_{comp}/E_{exp}	0.970747	0.957073	0.927911	1.01034	0.978677	0.981346	0.956776	0.950503	0.987842

Table S276: f_{comp} vs f_{exp} PCM/TD-DFT/TDA/6-311++G**/p-gauge/Exact Band Limits/

	B3P86	CAM-B3LYP	LC-wHPBE	M05	mPW1PW91	O3LYP	SVWN	wB97XD	B3LYP
MAE	0.190311	0.162922	0.194882	0.177137	0.186678	0.19243	0.206174	0.166052	0.187146
Slope	0.454037	0.563659	0.458364	0.523951	0.462616	0.435642	0.382756	0.520995	0.468827
Intercept	-0.022343	-0.0234018	-0.0143859	-0.0297137	-0.0207979	-0.0186062	-0.0155627	-0.0105876	-0.0236381
R^2	0.681769	0.62677	0.343092	0.723787	0.702114	0.71572	0.676575	0.524769	0.712736
$\|\Delta E\|$	0.448349	0.821724	1.04118	0.267434	0.439691	0.270958	0.384474	0.889672	0.32008
ΔE	-0.167588	-0.282164	-0.409769	0.0208778	-0.125374	-0.0648285	-0.1853	-0.316189	-0.0692313
E_{comp}/E_{exp}	0.971472	0.958235	0.926498	1.00984	0.978667	0.98703	0.955991	0.951657	0.989808

Table S277: f_{comp} vs f_{exp} PCM/TD-DFT/TDA/6-311++G**/xp-gauge/Exact Band Limits/

	B3P86	CAM-B3LYP	LC-wHPBE	M05	mPW1PW91	O3LYP	SVWN	wB97XD	B3LYP
MAE	0.112892	0.109682	0.170643	0.104465	0.107282	0.11251	0.124295	0.120431	0.110482
Slope	0.840941	0.922366	0.714055	0.912795	0.841769	0.818713	0.755149	0.853846	0.851755
Intercept	-0.0385771	-0.031788	-0.0179505	-0.0475587	-0.034986	-0.0366514	-0.0347888	-0.014244	-0.0393147
R^2	0.751668	0.699247	0.398148	0.796792	0.77446	0.785155	0.758927	0.607521	0.774312
$\|\Delta E\|$	0.448512	0.824957	1.09737	0.268418	0.435859	0.26819	0.319213	0.892991	0.320474
ΔE	-0.164216	-0.277584	-0.462588	0.0249518	-0.122789	-0.0777981	-0.147347	-0.312227	-0.0685225
E_{comp}/E_{exp}	0.972241	0.959483	0.916798	1.01077	0.979515	0.983843	0.962737	0.952767	0.989955

Improving Fit:

Table S278: f_{comp} vs f_{exp} PCM/TD-DFT/TDA/6-311++G**/x-gauge/ImprovedFit/

	B3P86	CAM-B3LYP	LC-wHPBE	M05	mPW1PW91	O3LYP	SVWN	wB97XD	B3LYP
MAE	0.143261	0.157043	0.172644	0.135566	0.145485	0.107138	0.0872535	0.161925	0.146519
Slope	1.21069	1.15701	1.3132	1.18151	1.30555	1.24503	1.18944	1.20216	1.22088
Intercept	0.0139534	0.0157899	-0.0236578	0.0188397	-0.00749265	-0.01955	-0.000124222	0.0057357	0.0322017
R^2	0.670386	0.626709	0.692431	0.682585	0.723147	0.793766	0.798802	0.647563	0.62217
$\|\Delta E\|$	0.371592	0.807803	1.1645	0.330347	0.391449	0.435965	0.509919	0.812166	0.311139
ΔE	-0.00371874	-0.0123282	-0.215287	0.0396611	0.0660931	0.0212666	-0.156357	-0.025587	0.0402942
E_{comp}/E_{exp}	1.00069	1.00505	0.953121	1.00601	1.01892	1.00455	0.961919	1.00603	1.01105

Table S279: f_{comp} vs f_{exp} PCM/TD-DFT/TDA/6-311++G**/p-gauge/ImprovedFit/

	B3P86	CAM-B3LYP	LC-wHPBE	M05	mPW1PW91	O3LYP	SVWN	wB97XD	B3LYP
MAE	0.0875394	0.0659853	0.07959	0.0841265	0.0819629	0.117421	0.120403	0.0613124	0.0954865
Slope	0.661248	0.784011	0.851699	0.690323	0.691031	0.536868	0.493061	0.812954	0.629457
Intercept	0.0192662	0.00882714	-0.00803974	0.0164506	0.0158941	0.0265159	0.0385935	0.00888504	0.0210965
R^2	0.717965	0.796878	0.749299	0.728423	0.724266	0.631538	0.573193	0.813945	0.675513
$\|\Delta E\|$	0.925739	1.03643	1.13437	0.805573	0.885481	0.688814	0.701207	0.986629	0.797853
ΔE	0.878681	0.80603	0.984853	0.785885	0.856624	0.599698	0.541642	0.975771	0.740863
E_{comp}/E_{exp}	1.20494	1.19336	1.2328	1.18633	1.20258	1.14135	1.125	1.22968	1.17368

Table S280: f_{comp} vs f_{exp} PCM/TD-DFT/TDA/6-311++G**/xp-gauge/ImprovedFit/

	B3P86	CAM-B3LYP	LC-wHPBE	M05	mPW1PW91	O3LYP	SVWN	wB97XD	B3LYP
MAE	0.05327	0.0573688	0.0747441	0.0520076	0.0524865	0.05571	0.0586159	0.0589253	0.0533359
Slope	0.994067	1.07556	1.07158	0.987565	0.996862	0.848163	0.861053	1.0519	1.00007
Intercept	-0.0352766	-0.0274351	-0.0459483	-0.0249715	-0.0289317	-0.00287078	-0.00923768	-0.0293834	-0.0273812
R^2	0.916355	0.888209	0.819084	0.906883	0.908092	0.875818	0.862832	0.871685	0.888148
$\|\Delta E\|$	0.456568	0.567766	0.731986	0.461771	0.551607	0.435608	0.442865	0.594209	0.457616
ΔE	0.382432	0.564647	0.601549	0.404962	0.33205	0.295857	0.178184	0.589761	0.373018
E_{comp}/E_{exp}	1.08681	1.13309	1.14127	1.09504	1.07326	1.07069	1.03903	1.1383	1.09025

S3.9.2 nf_{exp} VHHM transitions

Looking only at VH, H, and M transitions: Avg nf_{exp} = 0.433273

Table S281: f_{comp} vs nf_{exp} PCM/TD-DFT/TDA/6-311++G**/x-gauge/Exact Band Limits/

	B3P86	CAM-B3LYP	LC-wHPBE	M05	mPW1PW91	O3LYP	SVWN	wB97XD	B3LYP
MAE	0.141487	0.172694	0.237349	0.126182	0.146742	0.134378	0.161424	0.183201	0.137499
Slope	1.16561	1.06733	0.747076	1.17158	1.12644	1.16141	1.19604	0.985384	1.14622
Intercept	0.00228423	0.0326134	0.047683	-0.00232917	0.014618	0.00227207	-0.00893529	0.0570834	0.00785839
R^2	0.794339	0.744379	0.393143	0.834685	0.800831	0.799661	0.731901	0.673824	0.795879
$\|\Delta E\|$	0.444154	0.813286	1.03648	0.258337	0.426524	0.268282	0.315795	0.882524	0.318475
ΔE	-0.170306	-0.288467	-0.401749	0.0235285	-0.126664	-0.0882073	-0.169291	-0.322294	-0.0774471
E_{comp}/E_{exp}	0.970747	0.957073	0.927911	1.01034	0.978677	0.981346	0.956776	0.950503	0.987842

Table S282: f_{comp} vs nf_{exp} PCM/TD-DFT/TDA/6-311++G**/p-gauge/Exact Band Limits/

	B3P86	CAM-B3LYP	LC-wHPBE	M05	mPW1PW91	O3LYP	SVWN	wB97XD	B3LYP
MAE	0.315929	0.283262	0.30991	0.30179	0.311745	0.317852	0.331079	0.283928	0.312674
Slope	0.319846	0.39392	0.312057	0.369941	0.325407	0.307766	0.27218	0.359852	0.330559
Intercept	-0.021237	-0.0206644	-0.00857384	-0.028803	-0.0194618	-0.0179254	-0.0157341	-0.00621509	-0.0226234
R^2	0.684353	0.619208	0.321663	0.729857	0.70269	0.72255	0.692035	0.506399	0.716712
$\|\Delta E\|$	0.448349	0.821724	1.04118	0.267434	0.439691	0.270958	0.384474	0.889672	0.32008
ΔE	-0.167588	-0.282164	-0.409769	0.0208778	-0.125374	-0.0648285	-0.1853	-0.316189	-0.0692313
E_{comp}/E_{exp}	0.971472	0.958235	0.926498	1.00984	0.978667	0.98703	0.955991	0.951657	0.989808

Table S283: f_{comp} vs nf_{exp} PCM/TD-DFT/TDA/6-311++G**/xp-gauge/Exact Band Limits/

	B3P86	CAM-B3LYP	LC-wHPBE	M05	mPW1PW91	O3LYP	SVWN	wB97XD	B3LYP
MAE	0.218	0.192331	0.24933	0.20661	0.209691	0.221342	0.237007	0.198714	0.215348
Slope	0.587505	0.642262	0.486232	0.639595	0.587429	0.573084	0.530659	0.588984	0.595368
Intercept	-0.0344076	-0.0262921	-0.00893948	-0.043852	-0.0305288	-0.0330721	-0.0323835	-0.00674505	-0.035225
R^2	0.7421	0.685791	0.373434	0.791321	0.762901	0.778166	0.75807	0.584728	0.765246
$\|\Delta E\|$	0.448512	0.824957	1.09737	0.268418	0.435859	0.26819	0.319213	0.892991	0.320474
ΔE	-0.164216	-0.277584	-0.462588	0.0249518	-0.122789	-0.0777981	-0.147347	-0.312227	-0.0685225
E_{comp}/E_{exp}	0.972241	0.959483	0.916798	1.01077	0.979515	0.983843	0.962737	0.952767	0.989955

Improving fit:

Table S284: f_{comp} vs nf_{exp} PCM/TD-DFT/TDA/6-311++G**/x-gauge/Improved Fit/

	B3P86	CAM-B3LYP	LC-wHPBE	M05	mPW1PW91	O3LYP	SVWN	wB97XD	B3LYP
MAE	0.0834288	0.104081	0.1325	0.0850364	0.0868225	0.0734818	0.0640869	0.109101	0.0864081
Slope	1.0395	1.09414	1.08409	1.09079	1.12549	1.06802	1.04031	1.0934	1.11894
Intercept	0.0175446	0.0225157	-0.00872496	0.0146225	0.00943193	0.0107326	0.00418526	0.0269341	0.0100225
R^2	0.876942	0.862089	0.800097	0.886333	0.892098	0.888722	0.914478	0.86401	0.841399
$\|\Delta E\|$	0.340493	0.575801	0.833519	0.232813	0.341853	0.243373	0.28817	0.557933	0.275458
ΔE	0.0902485	0.155027	0.164702	0.127809	0.156454	0.00458007	-0.077895	0.263008	0.120997
E_{comp}/E_{exp}	1.02466	1.03619	1.04122	1.03058	1.03834	0.99937	0.981948	1.06093	1.02906

Table S285: f_{comp} vs nf_{exp} PCM/TD-DFT/TDA/6-311++G**/p-gauge/Improved Fit/

	B3P86	CAM-B3LYP	LC-wHPBE	M05	mPW1PW91	O3LYP	SVWN	wB97XD	B3LYP
MAE	0.165186	0.133913	0.113916	0.156908	0.166655	0.223242	0.224806	0.123355	0.178016
Slope	0.490888	0.602924	0.682292	0.518477	0.505831	0.343906	0.351774	0.630044	0.446534
Intercept	0.0589632	0.0439694	0.032293	0.0524745	0.0488043	0.061968	0.0561531	0.0448825	0.0625126
R^2	0.564691	0.673317	0.692832	0.600817	0.60911	0.48556	0.502834	0.680261	0.594789
$\|\Delta E\|$	1.13365	1.1783	1.427	0.965784	1.01124	0.79734	0.772621	1.14724	0.976975
ΔE	1.00906	1.05567	1.20366	0.927326	0.992686	0.712813	0.672018	1.13323	0.941388
E_{comp}/E_{exp}	1.23518	1.25143	1.28512	1.22059	1.23275	1.16602	1.15433	1.26266	1.22186

Table S286: f_{comp} vs nf_{exp} PCM/TD-DFT/TDA/6-311++G**/x-gauge/Improved Fit/

	B3P86	CAM-B3LYP	LC-wHPBE	M05	mPW1PW91	O3LYP	SVWN	wB97XD	B3LYP
MAE	0.101864	0.0767293	0.101087	0.0895971	0.0943844	0.122034	0.133014	0.0733959	0.104973
Slope	0.807898	0.867747	0.932278	0.773892	0.79094	0.654309	0.634532	0.889079	0.773205
Intercept	-0.0033369	0.00161701	-0.0439227	0.0164349	0.0023671	0.0345433	0.0276756	-0.00295727	0.00906291
R^2	0.834417	0.884189	0.848622	0.836554	0.852558	0.738113	0.71711	0.889846	0.799022
$\|\Delta E\|$	0.707895	0.78285	0.95672	0.714385	0.691279	0.613447	0.576337	0.869495	0.660366
ΔE	0.553173	0.781169	0.63348	0.573549	0.666096	0.516454	0.375715	0.782326	0.605538
E_{comp}/E_{exp}	1.1265	1.18148	1.147	1.13464	1.15463	1.11967	1.08664	1.18259	1.14072

S3.9.3 f_{exp}/n VHHM transitions

Avg $f_{exp}/n = 0.218714$

Table S287: f_{comp} vs f_{exp}/n PCM/TD-DFT/TDA/6-311++G**/x-gauge/Exact Band Limits/

	B3P86	CAM-B3LYP	LC-wHPBE	M05	mPW1PW91	O3LYP	SVWN	wB97XD	B3LYP
MAE	0.297643	0.308342	0.271104	0.295325	0.296686	0.289686	0.301448	0.298201	0.293926
Slope	2.40561	2.19685	1.60101	2.40277	2.32942	2.402	2.47674	2.05442	2.36968
Intercept	-0.018832	0.0145762	0.0212064	-0.0202345	-0.00680501	-0.0198723	-0.0324199	0.0346918	-0.0138008
R^2	0.833952	0.777293	0.445038	0.865344	0.844132	0.843079	0.773581	0.721943	0.838459
$\|\Delta E\|$	0.444154	0.813286	1.03648	0.258337	0.426524	0.268282	0.315795	0.882524	0.318475
ΔE	-0.170306	-0.288467	-0.401749	0.0235285	-0.126664	-0.0882073	-0.169291	-0.322294	-0.0774471
E_{comp}/E_{exp}	0.970747	0.957073	0.927911	1.01034	0.978677	0.981346	0.956776	0.950503	0.987842

Table S288: f_{comp} vs f_{exp}/n PCM/TD-DFT/TDA/6-311++G**/p-gauge/Exact Band Limits/

	B3P86	CAM-B3LYP	LC-wHPBE	M05	mPW1PW91	O3LYP	SVWN	wB97XD	B3LYP
MAE	0.106897	0.0946251	0.133301	0.0968737	0.102667	0.107151	0.120551	0.10008	0.104202
Slope	0.639909	0.800842	0.667955	0.736802	0.652919	0.612197	0.534336	0.748674	0.660179
Intercept	-0.0226137	-0.0251448	-0.0194595	-0.0296668	-0.0212744	-0.0184749	-0.0146728	-0.0140469	-0.0237918
R^2	0.675185	0.630812	0.363259	0.713613	0.697293	0.704688	0.657403	0.54028	0.704625
$\|\Delta E\|$	0.448349	0.821724	1.04118	0.267434	0.439691	0.270958	0.384474	0.889672	0.32008
ΔE	-0.167588	-0.282164	-0.409769	0.0208778	-0.125374	-0.0648285	-0.1853	-0.316189	-0.0692313
E_{comp}/E_{exp}	0.971472	0.958235	0.926498	1.00984	0.978667	0.98703	0.955991	0.951657	0.989808

Table S289: f_{comp} vs f_{exp}/n PCM/TD-DFT/TDA/6-311++G**/xp-gauge/Exact Band Limits/

	B3P86	CAM-B3LYP	LC-wHPBE	M05	mPW1PW91	O3LYP	SVWN	wB97XD	B3LYP
MAE	0.084075	0.112462	0.15241	0.0840538	0.0841272	0.0753293	0.0811156	0.120503	0.0799835
Slope	1.19506	1.31521	1.04032	1.29345	1.19747	1.16122	1.06691	1.22856	1.20984
Intercept	-0.0412345	-0.0356736	-0.0258018	-0.0496283	-0.0379154	-0.0387452	-0.0358129	-0.0202575	-0.0418765
R^2	0.756844	0.70884	0.421357	0.797678	0.7814	0.7875	0.755309	0.627084	0.77888
$\|\Delta E\|$	0.448512	0.824957	1.09737	0.268418	0.435859	0.26819	0.319213	0.892991	0.320474
ΔE	-0.164216	-0.277584	-0.462588	0.0249518	-0.122789	-0.0777981	-0.147347	-0.312227	-0.0685225
E_{comp}/E_{exp}	0.972241	0.959483	0.916798	1.01077	0.979515	0.983843	0.962737	0.952767	0.989955

Improving fit:

Table S290: f_{comp} vs f_{exp}/n PCM/TD-DFT/TDA/6-311++G**/x-gauge/Improved Fit/

	B3P86	CAM-B3LYP	LC-wHPBE	M05	mPW1PW91	O3LYP	SVWN	wB97XD	B3LYP
MAE	0.137759	0.153438	0.177167	0.143207	0.144876	0.105264	0.0895263	0.144949	0.146236
Slope	1.26311	1.15604	1.2039	1.24289	1.45833	1.18976	1.30895	1.1863	1.42143
Intercept	-0.0365701	-0.0342512	-0.0605632	-0.0377876	-0.0600664	-0.053832	-0.0210969	-0.0468624	-0.0475174
R^2	0.472643	0.425238	0.40576	0.476093	0.535885	0.550455	0.652197	0.454878	0.467242
$\|\Delta E\|$	0.737981	1.08929	1.29072	0.558975	0.722897	0.653525	0.705797	1.13615	0.690708
ΔE	-0.134705	-0.391928	-0.546684	0.00657244	-0.0961958	0.0380842	-0.0554464	-0.374448	-0.00983794
E_{comp}/E_{exp}	0.978909	0.921452	0.876896	1.0009	0.98613	1.00934	0.980981	0.928585	1.00138

Table S291: f_{comp} vs f_{exp}/n PCM/TD-DFT/TDA/6-311++G**/p-gauge/Improved Fit/

	B3P86	CAM-B3LYP	LC-wHPBE	M05	mPW1PW91	O3LYP	SVWN	wB97XD	B3LYP
MAE	0.0417017	0.0423197	0.0589504	0.0436445	0.0418045	0.0546592	0.0592584	0.0471499	0.0449216
Slope	0.858831	0.942728	0.987674	0.842506	0.848458	0.72085	0.678525	0.987951	0.814953
Intercept	-0.00433739	-0.00345514	-0.0246937	-0.00352323	-0.00480395	0.00916818	0.0129416	-0.0144071	0.00116051
R^2	0.838679	0.831462	0.770739	0.819872	0.818683	0.7453	0.750139	0.825442	0.809684
$\|\Delta E\|$	0.766959	0.867749	0.982053	0.638884	0.697048	0.654758	0.608636	0.843579	0.67634
ΔE	0.686263	0.744284	0.609847	0.582067	0.657182	0.52956	0.406839	0.695082	0.609314
E_{comp}/E_{exp}	1.16147	1.17624	1.14859	1.14003	1.1547	1.12624	1.09565	1.16914	1.14542

Table S292: f_{comp} vs f_{exp}/n PCM/TD-DFT/TDA/6-311++G**/xp-gauge/Improved Fit/

	B3P86	CAM-B3LYP	LC-wHPBE	M05	mPW1PW91	O3LYP	SVWN	wB97XD	B3LYP
MAE	0.0446959	0.0670632	0.095343	0.0491721	0.0493977	0.0373124	0.0392388	0.0772832	0.0470584
Slope	1.16141	1.18915	1.2617	1.17725	1.18974	1.08056	1.04539	1.19232	1.17339
Intercept	-0.0307094	-0.0261666	-0.0288949	-0.026563	-0.0288839	-0.0243591	-0.0279669	-0.0256936	-0.0310823
R^2	0.889591	0.783652	0.731542	0.867097	0.874368	0.898032	0.893637	0.750377	0.84142
$\|\Delta E\|$	0.422232	0.534289	0.763674	0.347179	0.390159	0.410342	0.337192	0.604461	0.348274
ΔE	0.178819	0.382186	0.382723	0.247064	0.307413	0.0334829	0.0366285	0.315689	0.220322
E_{comp}/E_{exp}	1.04547	1.09241	1.09829	1.06056	1.07383	1.00996	1.00698	1.08037	1.05295

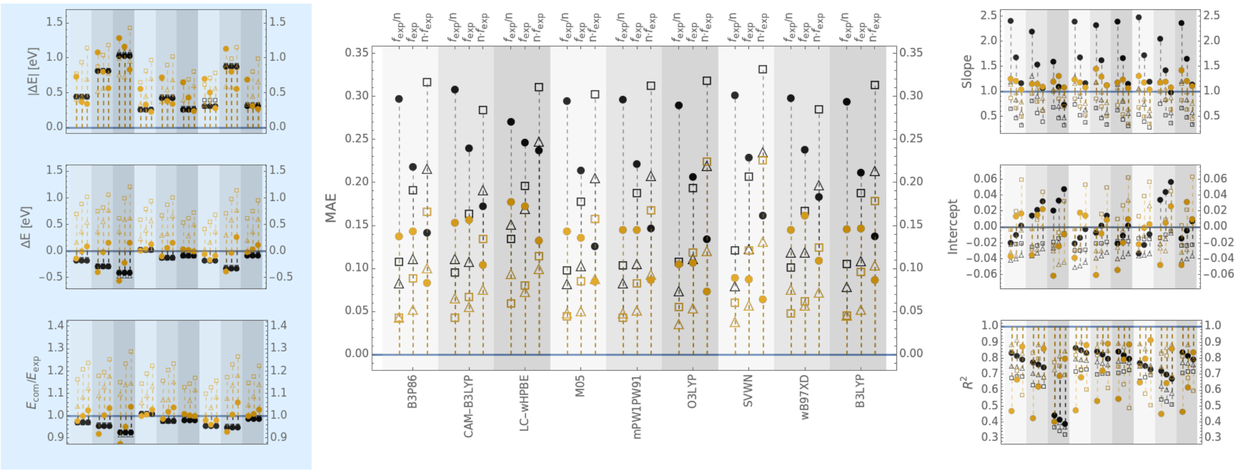

Figure S5: Comparison of f-values computed using TDA to a subset of 85 experimental transitions. For each method the f_{comp} values are compared to f_{exp}/n (left) f_{exp} (center) and $n \cdot f_{exp}$ (right). For reference, the average value of the experimental f-values are $\langle f_{exp}/n \rangle = 0.218714$, $\langle f_{exp} \rangle = 0.307655$, and $\langle n \cdot f_{exp} \rangle = 0.433273$. A full circle corresponds to the data obtained within the length gauge, an empty square to the velocity gauge, and an empty triangle to the mixed gauge. markers in black correspond to the Exact Band Limits comparison, while markers in yellow correspond to the Improved Fit comparison. Blue markers are used for the iterative comparison to $C \cdot f_{exp}$ The data displayed can be found in Tables of the supporting information.

S3.9.4 $C_0 = 1.0$ Cf_{exp} VHHM transitions

Table S293: f_{comp} vs Cf_{exp} PCM/TD-DFT/TDA/6-311++G**/x-gauge/Improved Fit/

	B3P86	CAM-B3LYP	LC-wHPBE	M05	mPW1PW91	O3LYP	SVWN	wB97XD	B3LYP
MAE	0.0598327	0.0564293	0.111494	0.0551262	0.0574787	0.0619222	0.0579612	0.0589331	0.0653885
Slope	1.02551	1.01977	1.03839	1.02577	1.02523	0.991407	1.00688	1.01743	1.02751
Intercept	-0.0240542	-0.0201849	-0.0342419	-0.0248959	-0.0233038	0.00720341	-0.00572185	-0.0174704	-0.0271704
R^2	0.951097	0.962145	0.856209	0.958699	0.956577	0.918814	0.935723	0.958664	0.928599
$\|\Delta E\|$	0.32007	0.553959	0.771878	0.284806	0.350304	0.24611	0.261729	0.610055	0.273412
ΔE	0.105333	0.325095	0.268198	0.214277	0.186618	0.0353669	-0.0737233	0.271008	0.152281
E_{comp}/E_{exp}	1.02405	1.07878	1.0711	1.04851	1.04365	1.00657	0.981431	1.06705	1.03381

Table S294: f_{comp} vs Cf_{exp} PCM/TD-DFT/TDA/6-311++G**/p-gauge/Improved Fit/

	B3P86	CAM-B3LYP	LC-wHPBE	M05	mPW1PW91	O3LYP	SVWN	wB97XD	B3LYP
MAE	0.0278564	0.0417492	0.0541006	0.0320615	0.0315725	0.0231447	0.0208624	0.0433795	0.0275452
Slope	1.0774	1.05064	1.08031	1.04675	1.04728	1.03498	1.08101	1.06248	1.05789
Intercept	-0.0187158	-0.0145923	-0.0255827	-0.0113643	-0.0112563	-0.00748798	-0.0136194	-0.0183939	-0.0123269
R^2	0.885079	0.791688	0.784159	0.846825	0.850997	0.841186	0.862435	0.799514	0.848826
$\|\Delta E\|$	0.556373	0.72798	0.934239	0.451221	0.46574	0.497424	0.474798	0.724041	0.410097
ΔE	0.337178	0.550896	0.576529	0.365346	0.416546	0.0792522	-0.000656572	0.577501	0.298822
E_{comp}/E_{exp}	1.08339	1.13485	1.14038	1.08967	1.09798	1.01984	1.00041	1.14143	1.07192

Table S295: f_{comp} vs Cf_{exp} PCM/TD-DFT/TDA/6-311++G**/xp-gauge/Improved Fit/

	B3P86	CAM-B3LYP	LC-wHPBE	M05	mPW1PW91	O3LYP	SVWN	wB97XD	B3LYP
MAE	0.0580148	0.0655674	0.0789666	0.0618087	0.0599329	0.0549351	0.0524991	0.070308	0.0623522
Slope	1.11847	1.07585	1.09716	1.09525	1.0989	1.11456	1.11918	1.07451	1.10808
Intercept	-0.0498539	-0.0402669	-0.0482754	-0.0415209	-0.0410756	-0.0407606	-0.0390525	-0.0353018	-0.0451916
R^2	0.891389	0.884168	0.808793	0.872956	0.876702	0.847446	0.838927	0.840384	0.847379
$\|\Delta E\|$	0.813329	0.618406	0.873473	0.436735	0.712067	0.761431	7.40681e+13	0.68663	0.527769
ΔE	0.615553	0.409344	0.379967	0.358499	0.538805	0.487663	-7.40681e+13	0.303132	0.401784
E_{comp}/E_{exp}	1.15875	1.09631	1.09749	1.08822	1.14093	1.12812	-1.69134e+13	1.07949	1.10131

S3.9.5 $C_0 = 1.4\ Cf_{exp}$ VHHM transitions

Table S296: f_{comp} vs Cf_{exp} PCM/TD-DFT/TDA/6-311++G**/x-gauge/Improved Fit/

The data in this table is identical to the data in corresponding table for C=1.0 (Table S293)

Table S297: f_{comp} vs Cf_{exp} PCM/TD-DFT/TDA/6-311++G**/p-gauge/Improved Fit/

The data in this table is identical to the data in corresponding table for C=1.0 (Table S294)

Table S298: f_{comp} vs Cf_{exp} PCM/TD-DFT/TDA/6-311++G**/xp-gauge/Improved Fit/

	B3P86	CAM-B3LYP	LC-wHPBE	M05	mPW1PW91	O3LYP	SVWN	wB97XD	B3LYP
MAE	0.0401772	0.0565743	0.0765463	0.0495177	0.0477132	0.0386088	0.0377217	0.0609039	0.0490107
Slope	1.07714	1.04332	1.08974	1.06311	1.05662	1.06107	1.0754	1.06478	1.06064
Intercept	-0.0349045	-0.0244479	-0.0447728	-0.0283268	-0.027443	-0.0233056	-0.0263072	-0.0369462	-0.0272854
R^2	0.923963	0.895946	0.808824	0.896679	0.903191	0.897962	0.902815	0.896853	0.871834
$\|\Delta E\|$	0.416774	0.612312	0.83697	0.350456	0.435149	0.333674	0.346145	0.688918	0.370304
ΔE	0.333445	0.609526	0.466491	0.258043	0.385547	0.12542	0.0514135	0.561131	0.261051
E_{comp}/E_{exp}	1.07737	1.14323	1.1148	1.06097	1.09016	1.02974	1.01165	1.13355	1.06124

S3.9.6 $C_0 = 0.7\ Cf_{exp}$ VHHM transitions

Table S299: f_{comp} vs Cf_{exp} PCM/TD-DFT/TDA/6-311++G**/x-gauge/Improved Fit/

	B3P86	CAM-B3LYP	LC-wHPBE	M05	mPW1PW91	O3LYP	SVWN	wB97XD	B3LYP
MAE	0.0598327	0.160429	0.183684	0.0551261	0.0573194	0.0619681	0.0579247	0.159122	0.0654814
Slope	1.02551	1.06537	1.13527	1.02577	1.02665	0.991501	1.0068	1.10713	1.02639
Intercept	-0.0240542	-0.0251224	-0.0558455	-0.0248959	-0.0233024	0.00711868	-0.00564567	-0.0434405	-0.0269033
R^2	0.951097	0.391046	0.430241	0.958699	0.956572	0.918821	0.935716	0.443422	0.928558
$\|\Delta E\|$	0.32007	1.03915	1.30675	0.284806	0.350371	0.24644	0.262038	1.05889	0.272923
ΔE	0.105333	-0.361194	-0.510652	0.214277	0.186685	0.035037	-0.0734142	-0.357628	0.152534
E_{comp}/E_{exp}	1.02405	0.930373	0.884361	1.04851	1.04367	1.00651	0.981493	0.931927	1.0339

Table S300: f_{comp} vs Cf_{exp} PCM/TD-DFT/TDA/6-311++G**/p-gauge/Improved Fit/

The data in this table is identical to the data in corresponding table for C=1.0 (Table S294)

Table S301: f_{comp} vs Cf_{exp} PCM/TD-DFT/TDA/6-311++G**/xp-gauge/Improved Fit/

	B3P86	CAM-B3LYP	LC-wHPBE	M05	mPW1PW91	O3LYP	SVWN	wB97XD	B3LYP
MAE	0.0401766	0.0549982	0.0765463	0.0487118	0.0484805	0.0386088	0.037484	0.0589614	0.0490184
Slope	1.07573	1.04417	1.08974	1.05919	1.06005	1.06107	1.07553	1.05667	1.06082
Intercept	-0.0348692	-0.0244495	-0.0447728	-0.0265407	-0.0291237	-0.0233056	-0.0263608	-0.0294374	-0.0272099
R^2	0.923868	0.896136	0.808824	0.896709	0.903721	0.897962	0.903032	0.871337	0.871875
$\|\Delta E\|$	0.415854	0.609832	0.83697	0.369183	0.450207	0.333674	0.353434	0.592581	0.378574
ΔE	0.332591	0.606767	0.466491	0.293762	0.316449	0.12542	0.0732707	0.588134	0.272651
E_{comp}/E_{exp}	1.07717	1.14256	1.1148	1.07012	1.0716	1.02974	1.01772	1.13789	1.06404

Table S302: C values obtained according to Algorithm 1 for the nine functionals considered, under the TDA

**Method**	**X-gauge**	**P-gauge**	**XP-gauge**
B3P86	1.80	0.45	0.86
CAM-B3LYP	1.95	0.53	1.07
LC-wHPBE	1.71	0.62	0.95
M05	1.85	0.46	0.86
mPW1PW91	1.85	0.46	0.87
O3LYP	1.60	0.37	0.73
SVWN	1.59	0.32	0.68
wB97XD	1.92	0.56	1.09
B3LYP	1.89	0.40	0.85

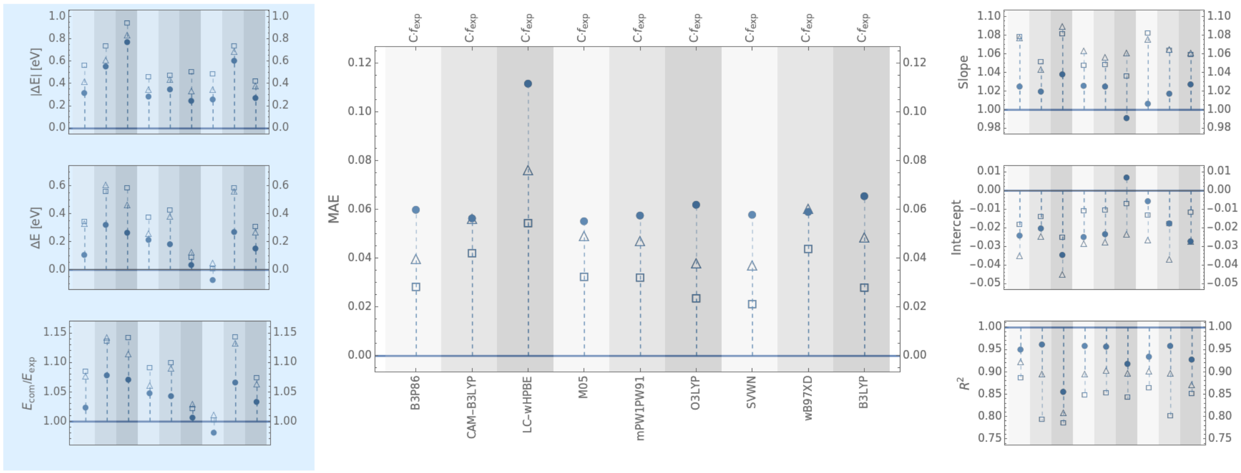

Figure S6: Comparison of f-values computed using TD-DFT with 6-311++G** under TDA, to a subset of 85 VHHM transitions. For each method, the f_{comp} values are compared to Cf_{exp} . A full circle corresponds to the data obtained within the length gauge, an empty square corresponds to the velocity gauge, while an empty triangle corresponds to the mixed gauge. The data displayed can be found in Tables S293 to S301.
